# Estimating the Incidence of Symptomatic Rotavirus Infections: A Systematic Review and Meta-Analysis

**DOI:** 10.1371/journal.pone.0006060

**Published:** 2009-06-26

**Authors:** Joke Bilcke, Pierre Van Damme, Marc Van Ranst, Niel Hens, Marc Aerts, Philippe Beutels

**Affiliations:** 1 Centre for Health Economics Research & Modeling of Infectious Diseases (CHERMID), Centre for the Evaluation of Vaccinations (CEV), Vaccine and Infectious Disease Institute (VAXINFECTIO), University of Antwerp, Wilrijk, Antwerp, Belgium; 2 Laboratory of Clinical and Epidemiological Virology, Department of Microbiology and Immunology, Rega Institute for Medical Research, Catholic University of Leuven, Leuven, Belgium; 3 Interuniversitary Institute for Biostatistics and Statistical Bioinformatics (I-BioStat), Hasselt University, Diepenbeek, Belgium; 4 School of Public Health, University of Sydney, Sydney, New South Wales, Australia; The Cochrane Collaboration, Germany

## Abstract

**Background:**

We conducted for the first time a systematic review, including a meta-analysis, of the incidence of symptomatic rotavirus (RV) infections, because (1) it was shown to be an influential factor in estimating the cost-effectiveness of RV vaccination, (2) multiple community-based studies assessed it prospectively, (3) previous studies indicated, inconclusively, it might be similar around the world.

**Methodology:**

Pubmed (which includes Medline) was searched for surveys assessing prospectively symptomatic (diarrheal) episodes in a general population and situation, which also reported on the number of the episodes being tested RV+ and on the persons and the time period observed. A bias assessment tool was developed and used according to Cochrane guidelines by 4 researchers with different backgrounds. Heterogeneity was explored graphically and by comparing fits of study-homogenous ‘fixed effects’ and -heterogeneous ‘random effects’ models. Data were synthesized using these models. Sensitivity analysis for uncertainty regarding data abstraction, bias assessment and included studies was performed.

**Principal Findings:**

Variability between the incidences obtained from 20 studies is unlikely to be due to study groups living in different environments (tropical versus temperate climate, slums versus middle-class suburban populations), nor due to the year the study was conducted (from 1967 to 2003). A random effects model was used to incorporate unexplained heterogeneity and resulted in a global incidence estimate of 0.31 [0.19; 0.50] symptomatic RV infections per personyear of observation for children below 2 years of age, and of 0.24 [0.17; 0.34] when excluding the extreme high value of 0.84 reported for Mayan Indians in Guatemala. Apart from the inclusion/exclusion of the latter study, results were robust.

**Conclusions/Significance:**

Rather than assumptions based on an ad-hoc selection of one or two studies, these pooled estimates (together with the measure for variability between populations) should be used as an input in future cost-effectiveness analyses of RV vaccination.

## Introduction

Meta-analyses of Randomized Controlled Trials (RCT's) are frequently undertaken as part of systematic reviews to estimate clinical effect sizes. Effect size estimates based on meta-analyses are even regarded as superior to estimates from a single RCT, particularly when they serve as an input in Health Technology Assessments(HTA's) [1], [2]. But clinical effect sizes are only one of the many input variables needed for a HTA (others include estimates on incidence of morbidity and mortality, costs and utilities), and meta-analysis for estimating these other types of variables have been used to a far lesser extent [1]. However, in some cases (e.g. when data is available from different sources for similar study groups, but not for the specific study group under analysis), meta-analysis could be as strong a tool for good estimation of these other types of variables, as it is for clinical effect sizes.

This seems for instance the case for the estimation of the incidence of symptomatic rotavirus (RV) infections, including those that do not present to a physician or a hospital. Despite common believe, several HTA studies showed this estimate to be influential on the cost-effectiveness of RV vaccination [3]–[8]. However, many other studies considered only RV cases presenting to physicians and hospitals [9]–[16], some of them justifying this by claiming a lack of information on RV cases not presenting to a physician or hospital. Nonetheless, a number of published studies recorded prospectively the incidence of symptomatic RV infections. Moreover, De Zoysa and Feachem [17] already pointed out that the incidence of symptomatic RV infections in a study group from Canada [18] was similar to one from Bangladesh [19]. This suggests that the incidence of RV diarrhoea may generally be similar between developing and developed countries, and that basic improvements in water supply, sanitation, hygiene, nutrition and education do not necessarily reduce this incidence.

In short, because (1) the incidence of all symptomatic RV infections can be an influential factor in estimating the cost-effectiveness of RV vaccination, (2) multiple community-based studies exist that assessed prospectively the incidence of symptomatic RV infections, and (3) it would be important for global public health policy to verify whether the incidence of symptomatic RV infections is similar around the world, we conducted a systematic review, including a meta-analysis, of community-based studies on the incidence of symptomatic RV infections. To our knowledge this is the first meta-analysis on this subject. The main aim of this meta-analysis is to produce reliable estimates of the incidence of symptomatic RV infections, which can then be used for HTA. Since this incidence was shown to be highly age-dependent [17], age is taken into account.

## Methods

The strategy used and the reporting hereafter is based on the QUORUM [20] and MOOSE statement [21] and follows Cochrane guidelines [22]. JB, who determined the strategy, is a Biologist (BSc, MSc), Biostatistician (MSc), and current PhD student. The strategy was implemented after conferring with PVD: vaccinologist and infectious disease epidemiologist (PhD); MVR: virologist and epidemiologist (PhD) and PB: infectious disease modeller and economist (PhD). All these authors have worked and published on RV before.

### Searching

Pubmed, the main database for health sciences that fully includes Medline, was searched using EndNote 9 (title, abstract and keywords of each paper were searched). A first search of PubMed to identify relevant papers for our meta-analysis was conducted on September 6^th^ 2007 using search string ‘rotavirus’ AND ‘infection’ NOT ‘review’ NOT ‘letter’ NOT ‘clinical trial’. But as not all relevant papers were detected through this search, a second, more exhaustive search of PubMed was performed on January 16^th^ 2008 using the search terms ‘rotavirus AND diarrh*’ OR ‘rotavirus AND symptom*’ OR ‘rotavirus AND gastroenteritis’ OR ‘rotavirus AND gastro-enteritis’. This search was updated up to January 12^th^ 2009 to yield our final set of papers for review. All papers in foreign languages (i.e. not familiar to any of the authors), but of possible interest based on an English title and/or abstract were translated by people ignorant of the purpose of our study. Papers published prior to 1985 were excluded since relevant studies up to 1985 were taken from the review on incidence of symptomatic RV infections published in 1985 (de Zoysa & Feachem [17]).

### Selection

All papers identified through the search were screened manually for eligibility by JB, based on titles, abstracts and full text if necessary. Only one person (JB) did the screening because of the heavy workload involved. However, to assure high reliability of the assessment of study eligibility, a detailed and complete list of eligibility criteria was defined by multiple researchers (JB and PB, advised by MVR and PVD), prior to scanning the references (as recommended by Cochrane guidelines [22]):

- **Original papers,** i.e. excluding review papers, letters and editorial comments.
- **Descriptive studies (community surveys),** i.e. not comparative, hence excluding controlled trials and case-control studies, because they often use a strictly controlled study design (e.g. strict recruitment criteria), and because all participants usually received some sort of intervention (placebo or treatment of interest).
- **Prospective,** because RV testing is required to discern RV from other infections as a cause of diarrhea, and such tests are not standard practice. Therefore, RV cases should be recorded prospectively, with RV testing included in the study protocol.
- In a **‘general population, and general situation’,** because we want to estimate the incidence of *all* symptomatic RV cases, and not restrict to cases for whom medical care is sought, nor to cases occurring in a particular setting such as day care centres, refugee camps and homes for the elderly. Reports of outbreaks are also excluded, because they focus on a particular period of time during which incidence is high.
- **On humans,** i.e. excluding studies of RV in animals.
- **Reporting incidence of symptomatic laboratory-confirmed RV infections per person-year directly OR reporting number/percentage of RV+ tests and number/percentage of diarrheal/symptomatic infections (implying the number of symptomatic RV+ cases can be derived), and the person-time of observation (or the number of persons under observation and the time period during which they were observed).** Studies assessing incidence of symptomatic RV infection using serological assays and not stool tests are not included, because it is very difficult to link symptoms with blood tests indicating RV.

### Validity assessment

In the absence of standard tools for validating studies that measure non-comparative outcomes, a simple and transparent risk of bias assessment tool was developed (for details see Text S1), based on existing validation tools for studies on comparative outcomes [22]. In a similar way as the ‘Cochrane collaboration's tool for assessing risk of bias’ [22], the tool consists of key sources of bias (selection bias, confounders age and season, drop-outs and detection bias) and is used to evaluate all studies that passed the first two selection hurdles described above (database search and eligibility criteria). Four assessors gave for each study a description and judgement of each of these sources of bias (see Table S1). All four assessors (JB, MVR, PB, PVD) had an adequate understanding of the methods used for assessing the incidence of symptomatic RV infections (see before), and MVR is an expert in laboratory techniques used to identify rotaviruses. Although the use of multiple assessors may limit bias, minimise errors and improve reliability, it could give rise to disagreements [22]. Therefore, as generally recommended [22], we adhered to the following procedure:

1. Compare the individual assessments in group and try to come to an agreement
2. If disagreement on a topic persists because of lack of knowledge on this topic: gather the information to get agreement.
3. If impossible to come to an agreement: report different opinions.

Based on a pilot study (for details see Text S1), it was decided that assessors should not be blinded from the results section nor author names, institutions and journal names because (1) the results section includes sometimes information on drop-outs and on how potential confounders were dealt with, and (2) converting the papers to a standardised anonymous format is labour intensive at the arguably low benefit of excluding journal names for assessors. We realise this could induce prejudice in interpretation but we believe we minimized such prejudice by having four independent assessors use a tool with specific questions. The papers to evaluate were given to each of the four assessors in a random order. Methodological papers from the reference lists were provided to the assessors upon request. Although attempts to assess risk of bias may be hampered by incomplete reporting, no attempt was made to obtain missing information from authors.

### Data abstraction

From each eligible paper, JB derived independently the incidence of laboratory-confirmed symptomatic RV infections as the number of such infections on the person-years of observation. The abstraction method is made completely transparent by reporting for each study in detail how the data were abstracted (Table S2).

### Study characteristics

All studies included are community surveys assessing prospectively symptomatic (diarrheal) episodes in a general population and situation (not restricted to e.g. day care centres), which also reported on the number of the episodes being tested RV+, and on the persons and the time period observed. Variation in study characteristics and their possible impact on the study outcomes was explored by plotting incidence per study according to a range of study characteristics, such as person-years of observation, risk of bias, Human Development Index (HDI, United Nations Development Programme's Human Development Report 2007/2008, compiled on the basis of 2005 data), socioeconomic status of study group (see further), and year in which the study was conducted (for multi-year studies, the midpoint was taken). Note that the data are too scarce to allow for a specific analysis with the aim of identifying explanatory variables.

### Quantitative data synthesis

*Principal measure* – Incidence of laboratory confirmed symptomatic RV infections, obtained as the number of such infections on the personyears of observation. 95% credible intervals for the incidence values reported in each study were obtained by taken the 95% interval of a gamma distribution with parameters *α*, the number of symptomatic RV+ episodes, and *β*, the inverse of the personyears of observation. The gamma distribution conforms with the gamma we assumed as a prior distribution in the models (see below).

#### Statistical heterogeneity

Statistical heterogeneity was explored graphically with a forest plot and by comparing the fit of a fixed-effects (study-homogeneous) model with a random-effects (study-heterogeneous) model [23]. The use of a random effects model allows for variation between the studies and can be used when the source of variation cannot be identified [22], [23]. A test for statistical heterogeneity (e.g. by calculating I^2^) was not performed, as we believe a pooled incidence estimate for symptomatic RV infections makes sense, even in case of (some) heterogeneity. This is because previous studies already indicated incidence might be similar around the world as RV is so contagious that basic improvements in water supply, sanitation, hygiene, nutrition and education do not necessarily decrease incidence. Note that incidence values taking into account possible heterogeneity will also be estimated (see further).

#### Combining results

A pooled estimate for the incidence of symptomatic RV infections was obtained by fitting the following models to the data:

Study-homogeneous (‘fixed effects’) model: This model assumes that the number of symptomatic RV infections, Y, as recorded in each study can be sampled from a Poisson distribution with a common mean (μt_i_).



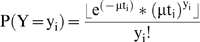



with

y_i_ = the number of symptomatic RV infections for each study i

μ = mean incidence rate (number of symptomatic RV infections/person-years of observation)

t_i_ = person-years of observation for each study i

Study-heterogeneous (‘random effects’) model [24]: This model allows the number of symptomatic RV infections recorded in each study to deviate from the common mean μt_i_ by a factor μ_i_, with μ_i_ having a gamma distribution defined by parameter v ( = shape parameter and scale parameter, i.e. a gamma distribution with common mean one and unknown variance). As μ_i_ works multiplicative on the mean μt_i_ of the Poisson, a positive gamma distribution with mean one is a logical choice to reflect the heterogeneity of the studies compared to the global incidence. With the above model, three types of incidences can be estimated:

- Global incidence μ, a common incidence estimate not taking into account heterogeneity between the studies.
- Incidence estimates for the study populations included in this meta-analysis μ_i_μ, taking into account the heterogeneity between the studies.
- Incidence estimates for study populations not included in this meta-analysis. This relates to the situation in which one wants to estimate the incidence for a specific population (in HTA this population will often be all children <2 years of age of one country) in the absence of empirical incidence estimates for this specific population. If the study population is then not directly comparable to any of the populations covered in our meta-analysis, the uncertainty stemming from this incomparability should be accounted for. This can be done by sampling from the gamma distribution (Gamma(v,v)), conditional on the posterior distribution for v to get an estimate for the deviation from the global incidence μ_u_, where μ_u_μ represents the incidence estimate. Note that the above procedure will increase the 95% credible interval around the global incidence estimate as more uncertainty is taken into account.

Models were fitted in Winbugs14, using uninformative gamma priors for μ and v (i.e. shape = scale parameter = 1E-03), implying results are not carried by the priors, but by the data. If possible, age would be added as a covariate to the models (continuous or ordinal, depending on the width of age intervals reported). In what we termed hereafter ‘base case analysis’, the models were fitted on the outcomes from the nine studies without high risk of bias.

#### Publication bias

Unlike for RCT's and observational studies on effects, for studies on incidence there is no reason to not publish results (i.e. no publication bias) as it does not involve a significance test for a certain treatment or intervention (and hence, there is no possible bias to false-positive results).

#### Sensitivity analysis

To investigate whether the conclusions rely on the inclusion/exclusion of any particular study, the meta-analysis was repeated systematically excluding each individual study in turn. Data were also re-analysed where uncertainties concerning the values extracted existed. Because there is little consensus concerning the optimal way of dealing with study validity in meta-analysis, the impact of excluding/including outcomes of studies with (possible) high risk of bias was investigated [22], [23].

## Results

### Flow of included studies (Figure 1)

**Figure 1:**
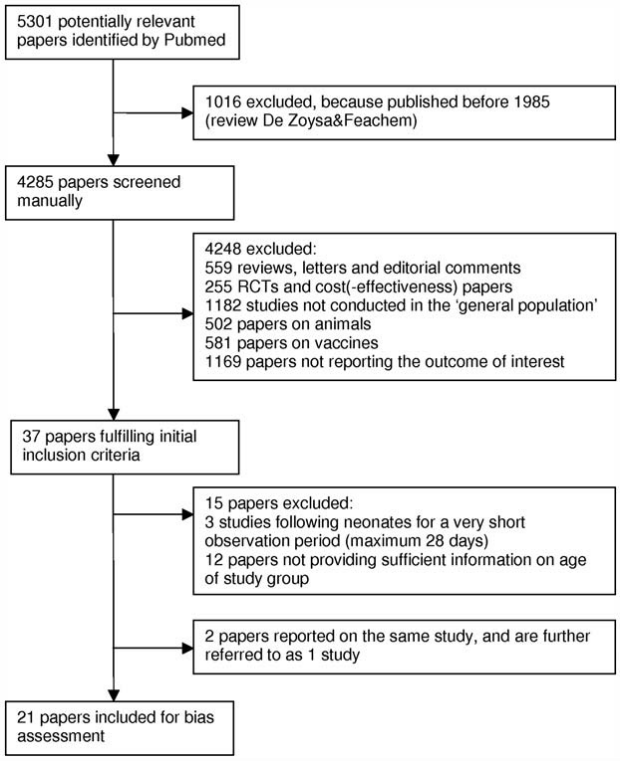
Flow diagram of the studies reviewed.

The literature search in Pubmed identified 5301 papers, 1016 of which were published before 1985. Manual screening resulted in 37 papers fulfilling the predefined inclusion criteria. Another 16 papers were excluded due to the following reasons:

- Three papers [25]–[27] followed neonates for a very short observation period (maximum 28 days).
- Twelve papers [28]–[39] did not provide sufficient information on age of the study group.
- Two papers [40], [41] reported on the same study, and are further referred to as one study.

Incidence estimates were obtained from the remaining 21 studies, and for all these studies the risk of bias in the reported outcomes was assessed. A separate reference list for the 5279 excluded studies, as recommended by the MOOSE statement [21], can be found in the Reference List S1.

### Study characteristics

The main characteristics of each of these 21 studies are presented in Table 1. Papers were assigned to different categories according to the socioeconomic status (SES) of the study group: low (study group living in poor, crowded area), probably low (studies performed in developing countries), not low (studies performed in developed countries) or unclear (Table 1).

**Table 1:** Background characteristics, incidence of symptomatic rotavirus infections per personyear of observation for children <2 years and bias assessment of 21 prospective studies included in the meta-analysis.

ref	country (location)	inc	year of study (midpoint)	study group/situation	ses	bias
[18]	Canada (Winnipeg, Manitoba)	0.24	1977	mothers in early postpartum period recruited at Health Sciences Centre; not biased toward high socioeconomic class	NL	P
[41]	Mexico (San Pedro Martir, around the southwestern outskirts of Mexico City)	0.38	1988	low income periurban area	L	N
[42], [67]	Nigeria (Ibadan)	1	NR	children born at a General Hospital; lower socioeconomic class	L	H
[43]	Chile (Población Carlos Condell slum, City of Santiago)	0.19	1981	all families with children <7years age living in slum	L	P
[44]	Mexico (rural village appr 180 km southwest of Mexico City)	0.33	1983	all children from the village	U	P
[45]	Guinea-Bissau (suburban districts Bandim II and Bele of Bissau)	0.24	1997	houses were randomly selected	PL	N
[46]	Central African Republic (Bangui)	0.14	1985	children in maternity ward (living in neighbourhood of the ward)	PL	H
[47]	Argentina (Avellaneda District, a suburb of Buenos Aires)	0.13	1984	families recruited when seeing pediatrician at Primary Health Care Center; low socioeconomic level, shantytowns, unstable and overcrowded houses	L	H
[48]	Guatemala (Cauqué)	0.84	1967	Mayan Indians; crowded	L	N
[49]	Bangladesh (Mirpur, Dhaka)	0.25	2003	urban slum	L	P
[50]	US (Northern Virginia)	0.22	1978	patients from a group pediatric practice; middle-class suburban population	NL	H
[51]	Palestina (Gaza: Jebaliya)	0.08	1985	crowded, poor	L	P
[52]	Costa Rica (Puriscal)	0.06	1983	children born in Hospital San Juan de Dios in San José (97% of babies from Puriscal are born here)	NL	N
[53]	China (suburb of Hong Kong)	0.06	NR	low- to low-middle income families	U	H
[54]	Nicaragua (Léon)	0.23	1992	children born in university hospital of whom mothers lived in one of the 3 health areas in the city	NL	N
[55]	Bangladesh (10 villages in Mirzapur, rural area appr 60 km from Dhaka)	0.21	1994	door-to-door census; crowded, poor	L	N
[56]	Egypt (2 villages in the vicinity of a rural district appr 40 km from Alexandria)	0.29	1995	house-to-house census	U	N
[57]	Bangladesh (Enayet Nagar and Sepai Kandi)	0.41	1978	Matlab field research area	L	H
[58]	Egypt (Epidemiology Study Center Field research area near Bilbeis)	0.21	1982	8 villages were selected in the Epidemiology Study Center field research area	U	N
[59]	Gambia (Bakau)	0.51	1982	representative 55% sample of locally born children	PL	H
[60]	Brazil (peripheral area of Belem)	0.22	1984	poor housing, low socioeconomic level	L	N

Inc: incidence; SocioEconomic Status (ses): low (L: study group living in poor, crowded area), probably low (PL: studies performed in developing countries), not low (NL: studies performed in developed countries) or unclear (U). NR: not reported, N: no high risk of bias, P: possible high risk of bias and H: high risk of bias in the reported incidences.

1 children were followed for the first year of life only.

### Data abstraction

From the 21 papers, the number of symptomatic RV infections and person-years of observation were obtained for children under 2 years of age from all but one paper [42] (only data until age 1 available). Data on older children or adults were rare and often not separately presented. Outcomes for yearly, 6-monthly and 3-monthly age intervals could be obtained from 18, 15 and 3 papers respectively.

For most studies, outcomes of interest were reported or could be calculated directly using information provided by the study. For instance, if the number of RV infections and/or person-years of observation in the first and second year were reported separately, they were summed to get the number of RV infections for the first 2 years. In case person-months or -days were given instead of person-years of observation [18], [41]–[53], the numbers were divided by 12, respectively 365. For Espinoza et al [54], the person-years of observation for age intervals 0–6 months and 6–12 months could be obtained by dividing the person-years of observation in the first year by two, because no drop-outs occurred during that period. For Hasan et al [55], the number of symptomatic RV infections was obtained by multiplying the number of diarrhea cases by the proportion due to RV.

When number of symptomatic RV infections, and/or person-years of observation was/were not reported, or could not be calculated directly, we approximated it (for details see Table S2).

The number of symptomatic RV infections and person-years of observation did not always match: during the observation period, stool samples were not always taken from all diarrhea episodes, and/or not all stool samples were tested for RV. When no adjustment is made for this, the incidence of symptomatic RV infections is likely to be underestimated. Therefore the incidence was adjusted by decreasing the person-years of observation by multiplying it by the proportion of diarrheal episodes tested for RV, when such information was given. For three of the papers evaluated not to have high risk of bias in their reported outcomes (and used in meta-analysis, see further) [41], [48], [56], the proportion of diarrheal episodes tested for RV was uncertain, however minimum and maximum proportions could be derived and/or assumed (for details, see Table S2). In base case analysis, midpoints of those minimum-maximum ranges were used for adjustment. When the proportion of diarrheal episodes tested for RV was given for only one age interval, we assumed the same proportion for other age intervals [42], [45], [54], [55], [57]–[59], for details see Table S2. Adjustment (when necessary) was achieved for 15 of the 21 studies, including all the studies without high risk of bias (see below).

Gurwith et al [18] provided outcomes per season so that we could adjust for the oversampling in winter (for details see Table S2).

### Assessment of risk of bias in the reported outcomes

The assessors came to agreement on the risk of bias for all studies. Nine studies were evaluated to be without high risk of bias in the reported incidence of symptomatic RV infections and 7 studies to be with high risk of bias (the reasons are presented in Table S3). Another 5 studies were evaluated as having a possible high risk of bias in their reported outcomes. In 3 of them, information was lacking so that validity assessment was not possible. The two other studies presented the appropriate information, and based on this they were regarded to be ‘borderline’, i.e. with risk of bias, but unclear if this risk is high.

The nine ‘without high risk of bias’ studies all used ELISA for RV testing, followed children at least for one complete year and visited the child at least once a week, with the exception of one study, in which the child was visited each time (s)he had diarrhea [60].

### Exploratory analysis

**Figure 2:**
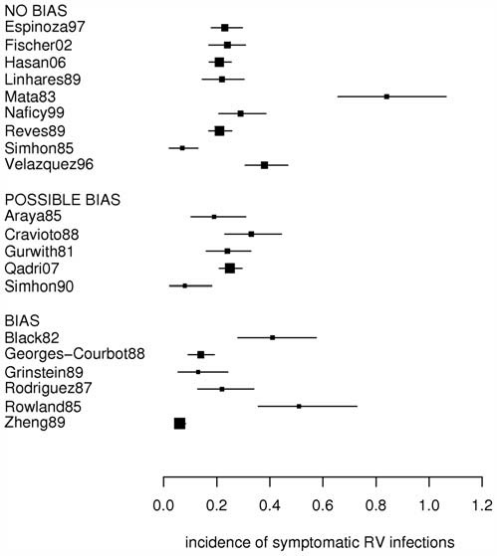
Incidence of symptomatic RV infections in children below age 2 for the 20 eligible studies. Forest plot showing incidence (number of symptomatic RV infections per personyear of observation; boxes) and 95% credible intervals (horizontal lines), obtained from studies which were evaluated to be at respectively no high risk of bias (‘no bias’), possible high risk of bias (‘possible bias’), and high risk of bias (‘bias’). The size of each box is proportionate to the person-years of observation from each study.

Incidence in children below 2 years of age varied between 0.06 and 0.84 symptomatic infections per person-year of observation (Table 1, Figure 2). When only considering studies without high risk of bias, the range stays the same, but the majority of studies shows an incidence between 0.20 and 0.25 (Table 1, Figure 2).

**Figure 3:**
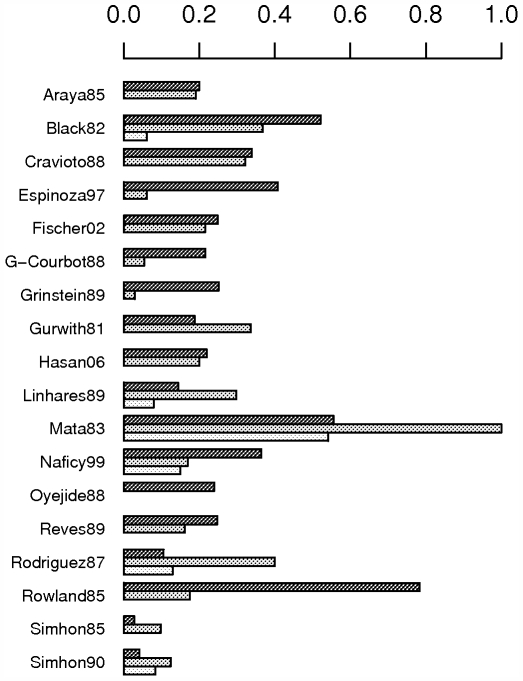
Incidence of symptomatic RV infections by age (in years) obtained from different studies. Incidence is number of symptomatic RV infections per personyear of observation. Black, grey and white bars represent incidences for children in their first, second and third year of life, respectively.

Figure 2 indicates heterogeneity, but variation between the studies seems not to be explained by differences in HDI, socioeconomic status of the study group or the year when the study was conducted, also not when only considering papers without high risk of bias (Table 1 and Figure S1). Age-specific incidences for yearly and 6-monthly intervals seem to vary even more between the studies, i.e. some papers report the peak incidence in the first year of life, whereas others found the incidence to peak in the second year of life (Figure 3). A peak incidence in the first year of life is due to a high incidence in the first 6 months of life (e.g. [46], [54]), or between 6 and 12 months of age (e.g. [56], [58], [59]). Only 2 papers [48], [52] with peak incidence in the second year of life had information on incidence in 6 monthly intervals, and report a high incidence between 12 and 18 months of age. The six papers from which 6-monthly data until 2 years of age could be derived [44]–[46], [48], [52], [55], all demonstrate a decrease in incidence for children aged 18–24 months.

Note that the variation in incidence between the studies without high risk of bias is very unlikely to be explained by differences in surveillance methods and/or RV tests, as these aspects were taken into account in the bias assessment: all studies without high risk of bias are considered to have used appropriate (i.e. unlikely leading to over- or underestimation) methodology to assess symptomatic RV episodes.

### Quantitative data synthesis

As only few studies provided detailed information on incidence of symptomatic RV infections by age, quantifying the variation by age (by adding age as a covariate in the models) was not considered feasible. Hence, all analyses are done on incidence values for children below 2 years of age.

### Base case analysis (9 studies without high risk of bias)

1. Global incidence estimate

The mean [95% credible interval] number of symptomatic RV infections per person-year of observation is estimated at 0.26 [0.24; 0.29] (median 0.26) by the study-homogeneous ‘fixed effects’ model and at 0.31 [0.19; 0.50] (median 0.30) by the study-heterogeneous ‘random effects’ model. The DIC value of 160.0 (study-homogeneous model) compared to 67.4 (study-heterogeneous model) suggests the model taking into account heterogeneity would best predict a replicate set of data of the same structure as the data included in the meta-analysis.

2. Incidence estimates for the studies included in the meta-analysis

The study-specific incidences estimated by the study-heterogeneous model and their 95% credible intervals are very close to the reported ones (Table 2). The smallest (Simhon et al [52]) and largest (Mata et al [48]) estimated values are most modified towards the pooled incidence estimate.

**Table 2:** Reported and estimated (study-heterogeneous meta-analysis model) incidence values of symptomatic RV infections in children <2 years.

	REPORTED			ESTIMATED		
Study	mean	LL	UL	mean	LL	UL
Espinoza et al [54]	**0.23**	0.18	0.30	**0.24**	0.18	0.30
Fischer et al [45]	**0.24**	0.17	0.31	**0.24**	0.18	0.31
Hasan et al [55]	**0.21**	0.17	0.26	**0.21**	0.17	0.26
Linhares et al [60]	**0.22**	0.15	0.31	**0.23**	0.16	0.31
Mata et al [48]	**0.84**	0.66	1.06	**0.79**	0.61	0.99
Naficy et al [56]	**0.29**	0.21	0.38	**0.29**	0.21	0.38
Reves et al [58]	**0.21**	0.17	0.26	**0.21**	0.17	0.26
Simhon et al [52]	**0.06**	0.02	0.13	**0.09**	0.03	0.17
Velazquez et al [41]	**0.38**	0.31	0.47	**0.38**	0.31	0.46
Pooled estimate				**0.31**	0.19	0.50

LL: lower 95% credible limit, UL: upper 95% credible limit.

3. Incidence estimate for study populations not included in the meta-analysis

The 95% credible interval increases substantially due to the extra source of uncertainty involved: 0.31 [0.05; 0.72].

### Sensitivity analysis

1. Meta-analysis on studies without high risk of bias: impact of excluding any particular study

**Figure 4:**
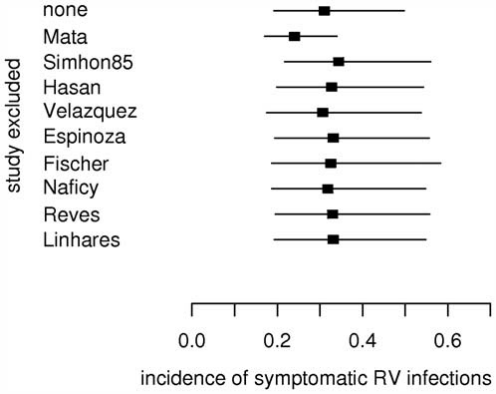
Impact of excluding any particular study from meta-analysis. Impact on the pooled estimate for the incidence of symptomatic RV infections in children below 2 years of age. Squares and horizontal lines represent means and 95% credible intervals of the pooled estimates. Only studies without high risk of bias are considered.

The exclusion of any single study from the meta-analysis does not have a remarkable impact, with the exception of excluding the Mata et al paper [48]. The latter substantially reduces both the pooled incidence estimate and its credible interval (Figure 4).

2. Meta-analysis on studies without high risk of bias: re-analysis of data where uncertainties concerning values extracted exist.

From the papers of Mata et al [48], Naficy et al [56] and Velazquez et al [41] it is unclear if all diarrheal episodes are tested for RV (see Table S2). Impact of this uncertainty is investigated by comparing the pooled incidence estimate when 100% testing of diarrhea episodes is assumed for Mata et al [48] and Naficy et al [56], and 100% testing of stool samples is assumed for Velazquez et al [41], with the pooled incidence estimate when 85.5% and 80% testing of diarrhea episodes is assumed for Mata et al [48] and Naficy et al [56], respectively and 80% testing of stool samples is assumed for Velazquez et al [41].

The pooled incidence estimate varies then between 0.30 [0.19; 0.46] (100% testing assumed) and 0.33 [0.20; 0.56] (85.5/80% testing assumed).

3. Impact of including studies with possible high risk of bias and high risk of bias

**Figure 5:**
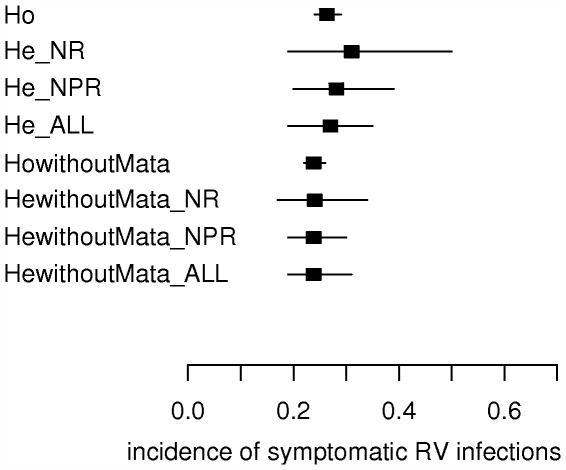
Impact of including/excluding studies from meta-analysis with possible high risk of bias, high risk of bias and the Mata et al paper. Impact on the pooled estimate for incidence of symptomatic RV infections for children below 2 years of age. Squares and horizontal lines represent means and 95% credible intervals of the pooled estimates. Ho = study-homogeneous model, He = study-heterogeneous model, NR = only studies with no high risk of bias included, NPR = studies with no or possible high risk of bias included, ALL = studies with no, possible or high risk of bias included, n = number of studies included in meta-analysis.

Including the studies with possible high risk of bias renders a slightly smaller pooled incidence estimate with a smaller credible interval: 0.28 [0.20; 0.39]. The same occurs when including the studies with high risk of bias: 0.27 [0.19; 0.35] (Figure 5).

4. Impact of the Mata et al paper [48]

When excluding the Mata et al paper [48], some heterogeneity between the studies remains (DIC study-heterogeneous model is 60.7 compared to 82.4 for the study-homogeneous model). The pooled incidence estimate lies then between 0.23 [0.17; 0.32] (100% testing assumed for Naficy et al [56]and Velazquez et al [41]) and 0.25 [0.17; 0.37] (80% testing assumed). When excluding the Mata et al paper [48], the credible interval for study populations not included in this meta-analysis becomes smaller: 0.24 [0.09; 0.45]. Including the studies with possible high risk of bias does not change the pooled ‘global’ incidence estimate, but the credible interval decreases slightly: 0.24 [0.19; 0.30]. The same occurs when including the studies with high risk of bias: 0.24 [0.18; 0.31] (Figure 5).

## Discussion

We estimated the number of laboratory-confirmed symptomatic RV infections per person-year of observation for children below 2 years of age, to be 0.31 [0.19; 0.50]. Excluding the high value (0.84) reported by Mata et al [48], reduces this to 0.24 [0.17; 0.34]. Mata et al's [48] estimate was based on a prospective follow-up study in Mayan Indians in Guatemala, living in poor, crowded areas. Other studies conducted in similar poor and crowded areas reported much lower values for the incidence of symptomatic RV infections (e.g. [41], [45], [55], [60]). This could be due to the local or ethnic pathogenicity of RV infections (e.g.: 39% versus 66% of RV infections were symptomatic in Velazquez et al [41] and Mata et al [48], respectively), or to a lower incidence of symptomatic and asymptomatic RV infections combined (e.g. 0.6 versus 1.1 RV infections per person-year for children <2 years of age in Fischer et al [45] and Mata et al [48], respectively). Also, the Mata et al [48] study was performed at least 10 years earlier than the other studies included, and RV tests were performed on faecal samples that were frozen for 13 to 18 years. However, it remains unclear whether these aspects explain the high incidence value.

The reported values of incidence of symptomatic RV infections apply to children below two years of age, and are based on data from different regions of the world (all continents, except for Australia and Europe), with different climates (from tropical to temperate), from populations with different socioeconomic status (from the slums of the City of Santiago in Chile to a middle-class suburban population in US), and at different times (1967–2003). Although it has already been shown that the proportion of gastroenteritis due to RV is seasonal, and that the extent of this seasonality depends on region [61], the meta analysis indicates region not to be an important determinant of the incidence. Incidence seems also independent of socioeconomic status and year of study, but one has to be aware of some limitations of the study:

### Limitations

The number of studies involved in this review is restricted, and additional information is needed to confirm results, especially with respect to possible explanatory factors for observed differences in incidences. For instance, only four studies in developed countries and/or study groups not having a low socioeconomic status were admissible for meta-analysis [18], [50], [52], [54]. Therefore, it could be of value to investigate the incidence of symptomatic RV infections in the placebo groups of RCTs on RV vaccination, as many RCTs were performed in developed countries (e.g. [62]–[65]). Other factors possibly influencing the incidence of symptomatic RV infection are the effect of breastfeeding and maternal antibodies, virulence of the circulating strain, co-infections and co-morbidity, etc.. Breastfeeding is believed to postpone symptomatic RV infection to a later age, which could explain why in some papers the incidence was found to peak in the second year of life, and not in the first year of life as in the other studies. However, studies on the protective effect of breastfeeding against RV infection have yielded variable results [66].

Another limitation of this review is the nature of the outcome of interest: there is no such thing as a ‘universally accepted’ method to assess the number of symptomatic RV infections per person-year of observation. Consequently, a large variety of methodologies are described by the papers, which could also (partially) explain the variability in the reported outcomes between the studies, i.e. the different papers did not necessarily measure the same events. However, by conducting for each paper a very exhaustive assessment of risk of bias in the reported outcome (with respect to our outcome of interest), and by carefully extracting and adjusting (if necessary) the outcomes of interest, we believe our pooled estimates for the number of laboratory-confirmed symptomatic RV infections per person-year of observation are reliable. Furthermore, sensitivity analysis showed that these estimates were robust for different selections of studies included in the meta-analysis, with the exception of the study by Mata et al [48].

When one wants to estimate the incidence of symptomatic RV+ infections for a specific population for which no prospective studies exist, and for which the resemblance with any of the populations included in our meta-analysis is uncertain, one should use the incidence estimates incorporating this extra source of uncertainty (i.e. 0.31[0.05; 0.72] with and 0.24 [0.09; 0.45] without inclusion of the Mata et al paper). The credible intervals reflect the different sources of uncertainties involved. Therefore, despite being relatively large these intervals are preferable to incidence estimates derived from an ad-hoc selection of one or two prospective studies. Clearly, once information is available on the incidence of symptomatic RV+ infections for the specific population of interest, incidence estimates and their credible intervals should be updated using this new information.

### Conclusion

In conclusion, given the currently available information, meta-analysis was shown to be a useful tool in getting an estimate for the incidence of symptomatic RV infections. Our study provides relevant information for disease burden estimates of RV. Furthermore the pooled (global) estimates, in combination with an extra measure of uncertainty to account for variability between studies (populations), should be used as an input in future CE analyses of RV vaccination rather than an ad-hoc selection of one or two of the studies we identified. As such, our meta-analysis fills one of the most important gaps for CE analyses on this subject and thus helps to reduce the uncertainty in the estimates they produce.

## Supporting Information

Text S1 Detailed description of developing the tool for assessing risk of bias.
(0.03 MB DOC)

Table S1 Tool for assessing risk of bias in each of the papers included in meta-analysis.
(0.04 MB DOC)

Table S2 Details on the way incidence of symptomatic RV infections in children <2 years of age were obtained from studies included in the meta-analysis.
(0.11 MB DOC)

Table S3 Assessment of risk of bias in the reported outcomes of the 21 prospective studies included in the meta-analysis.
(0.10 MB DOC)

Reference List S1 Studies excluded from bias assessment and meta-analysis.
(1.43 MB DOC)

Figure S1 Incidence of symptomatic RV infections by study characteristics. Incidence of symptomatic RV infections per personyear of observation in children below age 2 by Human Development Index (HDI) of the country where each study was conducted, socioeconomic status of the study group (0 = low; 1 = probably low; 2 = not low; 4 = unclear), and year in which the study was conducted (for multi-year studies, midpoint was taken). Black, grey and white circles represent incidences that were evaluated to be at respectively no high risk of bias, possible high risk of bias, and high risk of bias. The size of the circle is proportionate to the personyears of observation.
(0.01 MB TIF)
