# Supplementary material for: Estimating the Incidence of Symptomatic Rotavirus Infections: A Systematic Review and Meta-Analysis
Source: PLoS One. 2009 Jun 26;4(6):e6060. doi: 10.1371/journal.pone.0006060 (PMC2699052; doi:10.1371/journal.pone.0006060)
Supplement: Text S1 — Detailed description of developing the tool for assessing risk of bias. (0.03 MB DOC) [file pone.0006060.s001.doc]

**Text S1:** Detailed description of developing the tool for assessing risk of bias.

As recommended by the Cochrane collaboration[22], our method to assess risk of bias was tested first on a subset of six papers (named hereafter ‘pilot study’), which exhibited a large variety in methodologies and hence were vulnerable to a variety of risks of bias.

## Pilot study

Four assessors were individually required to record for each paper if it had in their opinion a high, medium, or low risk of bias in the reported outcome, and to explain in detail the reasons for their choice. Only the methods section of each paper was presented to the assessors. It was converted to a standard format and blinded to the names of the authors, institutions and journals (except for JB because she selected the papers).

## Final method for assessment of risk of bias

During the evaluation of the pilot study, it became apparent that the choice of score (low, medium, high) for risk of bias for each study was largely based on if and how the study took some key aspects into account. These key aspects/sources of bias were:

- Selection bias: the selected study participants should be representative for the population group in which RV incidence is measured.
- Confounder age: as the incidence of rotavirus infection is highly age-dependent[17], the age of the study group should be appropriately described and accounted for in the results (i.e. age-specific incidence).
- Confounder season: the incidence of rotavirus infection can be highly seasonal, depending on the country[61]. Hence, when children are only recruited over part of the year, the reported incidence may not be representative for the full year. So studies should have equal recruitment over all seasons, or present incidence estimates for seasons separately.
- Drop-outs: characteristics of participants withdrawn from the study and reasons for this should be mentioned and discussed in relation to the results.
- Detection bias: our outcome of interest – the incidence of symptomatic RV infections - is difficult to measure directly. Therefore most studies assessed it indirectly using a range of methods (e.g. first identifying episodes of diarrhea, next taking stool samples and testing them for RV). It is important to investigate these methods to determine whether the outcome reported by each study fits our purpose.

In a similar way as the ‘Cochrane collaboration’s tool for assessing risk of bias’[22], we made a tool consisting of these key sources of bias to evaluate all studies, i.e. the assessors gave for each study a description and judgement of each of these sources of bias (see Table S1b).
