# Supplementary material for: Estimating the Incidence of Symptomatic Rotavirus Infections: A Systematic Review and Meta-Analysis
Source: PLoS One. 2009 Jun 26;4(6):e6060. doi: 10.1371/journal.pone.0006060 (PMC2699052; doi:10.1371/journal.pone.0006060)
Supplement: Table S1 — Tool for assessing risk of bias in each of the papers included in meta-analysis. (0.04 MB DOC) [file pone.0006060.s002.doc]

**Table S1**: Tool for assessing risk of bias in each of the papers included in meta-analysis.

| sources of bias | ANSWER: detailed description (and/or mark text in the paper) | ANSWER: yes (high risk of bias) OR no (no high risk of bias) OR unclear |
| --- | --- | --- |
| 1.selection bias (study group) | Describe the study group that was selected. | Is the selected study group such that it could put the reported outcome (incidence of symptomatic RV episodes, or number of symptomatic RV infections and person-years of observation) at high risk of bias (i.e. high risk of under- or overestimation compared to the ‘true’ incidence of symptomatic RV infections)? |
| 2.confounder: age | Describe the age of the participants, and if and how it was controlled for in the outcome. | Is the age of the study participants appropriately controlled for in the outcome (incidence of symptomatic RV episodes, or number of symptomatic RV infections and person-years of observation), or could it put the reported outcome at high risk of bias (i.e. high risk of under- or overestimation compared to the ‘true’ incidence of symptomatic RV infections)? |
| 3.confounder: season | Describe the duration (and season(s)) of recruitment, and if and how it was controlled for in the outcome. | Is the duration (and season(s)) of recruitment appropriate, or is it appropriately controlled for in the outcome (incidence of symptomatic RV episodes, or number of symptomatic RV infections and person-years of observation), or could it put the reported outcome at high risk of bias (i.e. high risk of under- or overestimation compared to the ‘true’ incidence of symptomatic RV episodes)? |
| 4.drop-outs | Describe drop-outs from the analysis. State whether drop-outs were reported and whether reasons for drop-outs where reported. | Are the drop-outs and reasons for drop-out such that it could put the reported outcome (incidence of symptomatic RV infections, or number of symptomatic RV infections and person-years of observation) at high risk of bias (i.e. high risk of under- or overestimation compared to the ‘true’ incidence of symptomatic RV episodes)? |
| 5.detection bias | Describe in detail what was meant with a 'symptomatic RV infection' (e.g. definition of diarrhea, how episodes of diarrhea were assessed, how samples were taken, which RV test was used, ...). And describe how the total number of persons under study was determined, how the total follow-up time was determined, and if and how the incidence of symptomatic RV episodes was determined. | Does the study measures what we really want to measure, or is there a high risk of bias in the reported outcome compared to the outcome we are interested in (i.e. number of symptomatic RV infections per person-year of observation)? |
| 6.other | State any important concerns about bias, other than the ones stated above. | Had the study problems, other than the ones described above, that could put the reported outcome (incidence of symptomatic RV infections, or number of symptomatic RV infections and person-years of observation) at high risk of bias (i.e. high risk of under- or overestimation compared to the ‘true’ incidence of symptomatic RV infections)? |
| **OVERALL** | *do not write something here* | **Had the study problems that could put the reported outcome (incidence of symptomatic RV infections, or number of symptomatic RV infections and person-years of observation) at high risk of bias (i.e. high risk of under- or overestimation compared to the ‘true’ number of symptomatic RV infections and person-years of observation, or 'true' incidence of symptomatic RV infections)?** |
