# Supplementary material for: Estimating the Incidence of Symptomatic Rotavirus Infections: A Systematic Review and Meta-Analysis
Source: PLoS One. 2009 Jun 26;4(6):e6060. doi: 10.1371/journal.pone.0006060 (PMC2699052; doi:10.1371/journal.pone.0006060)
Supplement: Table S2 — Details on the way incidence of symptomatic RV infections in children <2 years of age were obtained from studies included in the meta-analysis. (0.11 MB DOC) [file pone.0006060.s003.doc]

**Table S2**: Details on the way incidence of symptomatic RV infections in children <2 years of age were obtained from studies included in the meta-analysis.

| ref | inc | n | person-years | how outcomes were obtained (calculated or approximated) and if they were adjusted for the % of diarrheal episodes tested for RV |
| --- | --- | --- | --- | --- |
| [18] | 0.24 | 28 | 117 | approximated: we decreased person-years of observation during winter so that it became equal as for summer, and decreased the number of symptomatic RV episodes accordingly; not adjusted: no info, but unlikely that 100% of diarrhea episodes was tested, as parents were asked to notify cases of gastrointestinal illness |
| [41] | 0.38 | 89 | 232 | calculated; adjustment for % stool samples taken, but no info on % stool samples tested: we assumed for this 80-100% |
| [42,67] | NR | NR | NR | outcomes for children <1 year of age and narrower age intervals: calculated; adjusted |
| [43] | 0.19 | 13 | 67 | calculated; adjusted |
| [44] | 0.33 | 37 | 112 | calculated; we assumed 100% for adjustment as the annual number of RV episodes and the number of samples tested are very similar:on average 56 children*3 diarrheal episodes=168 episodes per year; in 1st resp. 2nd year 170 resp. 202 samples tested |
| [45] | 0.24 | 46 | 195 | calculated; adjusted |
| [46] | 0.14 | 30 | 222 | calculated; not adjusted because no info |
| [47] | 0.13 | 7 | 53 | calculated; reported incidences were already adjusted by augmenting the observed number of episodes, we redid adjustment by decreasing the person-years of observation |
| [48] | 0.84 | 70 | 83 | calculated; we assumed between 85.5% and 100% for adjustment: Mata et al report that 85.5% of all weeks samples were taken and tested, but they also report: “2 RV+ weeks separated by 1 or 2 weeks without samples was considered as the same RV period”, hence proportion diarrheal episodes tested for RV is unclear |
| [49] | 0.25 | 121 | 486 | calculated; adjustment only for % stool samples tested; no info about % stool samples taken from diarrhea episodes |
| [50] | 0.22 | 17 | 78 | calculated; not adjusted: no info, but unlikely that 100% of diarrhea episodes was tested, as parents were contacted every 2 weeks and asked to take stool sample themselves in case of enteric illness. |
| [51] | 0.08 | 4 | 48 | calculated; not adjusted: no info, but unlikely that 100% of diarrhea episodes was tested as parents were asked to bring their children to clinic in case of diarrhea, to take stool sample there |
| [52] | 0.06 | 5 | 77 | calculated; assumed 100% for adjustment: as they report ‘Weekly testing for RV were achieved’ |
| [53] | 0.06 | 40 | 643 | as reported; not adjusted because no info |
| [54] | 0.23 | 54 | 230 | calculated; adjusted |
| [55] | 0.21 | 89 | 424 | approximated: incidence for children between 0-2 years of age could be obtained directly. For narrower age-intervals the person-years of observation were approximated as follows: 244 children followed for 2 years=488 person-years; + 8 children lost to follow up earlier (followed for a total of 1669 days): divide these days equally, i.e. followed up for about 7 months each, hence include this 8 children for 7 months each (i.e. birth to 7 months of age); adjusted |
| [56] | 0.29 | 41 | 142 | ^1^calculated; we assumed between 80% and 100% for adjustment: they report that from 80% of diarrhea episodes at least 1 stool specimen tested, but from all diarrhea episodes a rectal swap was taken. |
| [57] | 0.41 | 31 | 75 | approximated: annual incidences are derived from a plot (and hence approximations as the plot was not very detailed) and together with reported person-years of observation, they were used to calculate the annual number of RV infections. No data for the first 2 months of life, so we assumed no cases occurred during that period; adjusted |
| [58] | 0.21 | 98 | 463 | calculated; adjusted |
| [59] | 0.51 | 33 | 64 | approximated: they report an annual attrition rate of 20%, so we assumed that follow up for the first year was complete for all children, and that follow up for the second year decreased by 20%; adjusted |
| [60] | 0.22 | 30 | 136 | approximated: monthly number of cases were available, but not by age. As children were enrolled during 3.5 months, at each month there are children who can differ maximum 3.5 months in age. Hence, the number of infections for the 1st, 2nd and 3rd year resp., represents infections of children aged between 0-13, 9.5-23 and 21.5-37 months resp. To obtain the number of infections per year, we counted the number of infections per calendar year because the proportion used for adjustment was only recorded per calendar year; adjusted |

RV: rotavirus; inc: incidence; n: number of symptomatic RV infections; person-years: person-years of observation; calculated: (one of) the outcomes was not reported in the study, but could be calculated directly based on information provided by the study; approximated: outcomes could not be obtained directly; adjusted: outcomes are adjusted for the percentage of diarrheal episodes that were tested for RV during the observation period; NR: not reported

^1^Naficy et al(62) report that the number of person-years at risk = total person-years of follow-up minus the duration of diarrheal episodes after the first day of each episode. (Although in a table (which we used to obtain person-years of observation) they report ‘days of follow-up’). This will slightly overestimate incidence compared to other studies.
