# Supplementary material for: Estimating the Incidence of Symptomatic Rotavirus Infections: A Systematic Review and Meta-Analysis
Source: PLoS One. 2009 Jun 26;4(6):e6060. doi: 10.1371/journal.pone.0006060 (PMC2699052; doi:10.1371/journal.pone.0006060)
Supplement: Table S3 — Assessment of risk of bias in the reported outcomes of the 21 prospective studies included in the meta-analysis. (0.10 MB DOC) [file pone.0006060.s004.doc]

**Table S3**: Assessment of risk of bias in the reported outcomes of the 21 prospective studies included in the meta-analysis.

| ref | high risk of bias? | possible important sources of bias |
| --- | --- | --- |
| [18] | possible | drop-outs: not reported; detection: reliability of electron microscopy and seroconversion for RV detection at that time is unclear |
| [41] | no |  |
| [42,67] | yes | season: 35 dry and 96 wet season babies, not accounted for in the results; drop-outs: 27% |
| [43] | possible | season: only 9 months follow up; detection: gel electrophoresis can lead to underestimation |
| [44] | possible | detection: reliability of electron microscopy for RV detection at that time is unclear |
| [45] | no |  |
| [46] | yes | season: 20 months recruitment; drop-outs: 50% of initially recruited children excluded from analysis (4 died and 108 moved away or withdrawn due to poor follow-up) |
| [47] | yes | season: 18 months recruitment; selection: at least one child had to go to pediatrician; drop-outs: 19 of the 49 families (5 died, 7 withdrawn, 4 refused, 3 moved away); detection: changed protocol after 15 months of study, but do not report result for the 2 periods separately |
| [48] | no |  |
| [49] | possible | selection: method not described; drop-outs: 21% (55 outmigration or refusal by parents, 12 deaths of which 6 from diarrhea) |
| [50] | yes | detection: parents were called once in 2 weeks to ask about diarrheal episodes, reliance on mothers for diarrhea detection: they were asked to call and collect stool in case of diarrhea, this can lead to underestimation |
| [51] | possible | drop-outs: 20% (5 died, 21 excluded because of non-compliance); detection: reliance on parents to bring their children with diarrhea to clinic, but authors report 'parents were very cooperative', in Discussion authors state: lower incidence, because of less intensive surveillance? |
| [52] | no |  |
| [53] | yes | selection, season, drop-outs, detection: unclear |
| [54] | no |  |
| [55] | no |  |
| [56] | no |  |
| [57] | yes | detection: rectal swabs can lead to underestimation |
| [58] | no |  |
| [59] | yes | drop-outs: only from 48% of the recorded diarrhea episodes, a stool sample was tested; detection: reliance on mother to bring stool, can lead to underestimation (as acknowledged by the authors in Discussion) |
| [60] | no |  |
