# Supplementary material for: Estimating the Incidence of Symptomatic Rotavirus Infections: A Systematic Review and Meta-Analysis
Source: PLoS One. 2009 Jun 26;4(6):e6060. doi: 10.1371/journal.pone.0006060 (PMC2699052; doi:10.1371/journal.pone.0006060)
Supplement: Reference List S1 — Studies excluded from bias assessment and meta-analysis. (1.43 MB DOC) [file pone.0006060.s005.doc]

**Reference List S1:** Studies excluded from bias assessment and meta-analysis.

1. (1976) Rotavirus infections. Vet Rec 99: 363-364.

2. (1977) Rotavirus gastroenteritis. Br Med J 2: 784.

3. (1977) [Rotavirus infections]. Pediatria (Napoli) 85: VI-XIX contd.

4. (1978) Panel report on the Colloquium on Selected Diarrheal Diseases of the Young. J Am Vet Med Assoc 173: 515-518.

5. (1978) Panel report on the colloquium on selected diarrheal diseases of the young. J Am Vet Med Assoc 173: 315-318.

6. (1978) [Viruses and children's diarrhoea]. S Afr Med J 53: 777-778.

7. (1979) The rotavirus syndrome. Lancet 2: 186.

8. (1979) [Rotaviruses, the etiological agent of gastroenteritis]. Vopr Virusol: 86-87.

9. (1980) Rotavirus and other viral diarrhoeas: WHO scientific working group. Bull World Health Organ 58: 183-198.

10. (1980) Rotavirus infections in infancy. Br Med J 281: 1162-1163.

11. (1981) The how of breast milk and infection. Lancet 1: 1192-1193.

12. (1981) Towards a rotavirus vaccine. Lancet 2: 619-620.

13. (1982) Utilization of nutrients during and after diarrhoea. Glimpse 4: 2-4.

14. (1983) Clinical range of neonatal rotavirus gastroenteritis. Br Med J (Clin Res Ed) 286: 1745.

15. (1983) Diarrheal diseases control program: rotavirus diarrhea. MMWR Morb Mortal Wkly Rep 32: 311-312, 317.

16. (1983) [Symposium on gastroenteritis-inducing viruses in man and animal. Potsdam, December 1981]. Arch Exp Veterinarmed 37: 7-183.

17. (1985) Travelers' diarrhea. NIH Consensus Development Conference. Jama 253: 2700-2704.

18. (1987) [Rotaviruses and rotavirus gastroenteritis]. Vopr Virusol 32: 120-121.

19. (1989) Rotavirus vaccines. Indian J Pediatr 56: 548-550.

20. (1990) Puzzling diversity of rotaviruses. Lancet 335: 573-575.

21. (1991) Diarrhoeal Diseases Control Programme. Global activities. Wkly Epidemiol Rec 66: 209-213.

22. (1991) From the Centers for Disease Control. Rotavirus surveillance--United States, 1989-1990. Jama 265: 959-960.

23. (1991) Research priorities for diarrhoeal disease vaccines: memorandum from a WHO meeting. Bull World Health Organ 69: 667-676.

24. (1991) Rotavirus surveillance. CDR (Lond Engl Wkly) 1: 89.

25. (1991) Rotavirus surveillance--United States, 1989-1990. MMWR Morb Mortal Wkly Rep 40: 80-81, 87.

26. (1992) New children's vaccine initiative launched. Bull Pan Am Health Organ 26: 95-96.

27. (1992) Persistent diarrhoea still a serious and difficult problem. Glimpse 14: 1, 4.

28. (1994) ICDDR,B and ORS: the history of a miracle discovery. Glimpse 16: 3-4.

29. (1996) The threat of emerging infections. Glob Issues 1: 31-34.

30. (1997) A global vaccine for a global disease -- and end to rotavirus diarrhoea? CVI Forum: 2-6.

31. (1997) Laboratory-based surveillance for rotavirus -- United States, July 1996-June 1997. MMWR Morb Mortal Wkly Rep 46: 1092-1094.

32. (1997) New vaccine against diarrhea shown effective, but immediate impact in developing world unclear. Monday Dev 15: 7.

33. (1997) Scientific achievements of the Centre, 1991-1995, in research on child survival. Glimpse 19: 2-5.

34. (1997) Vaccine research and development. Rotavirus vaccines for developing countries. Wkly Epidemiol Rec 72: 35-40.

35. (1998) FDA licenses rotavirus vaccine for infants. Am J Health Syst Pharm 55: 2226, 2228.

36. (1998) Laboratory-based surveillance for rotavirus--United States, July 1997-June 1998. MMWR Morb Mortal Wkly Rep 47: 978-980.

37. (1998) Prevention of rotavirus disease: guidelines for use of rotavirus vaccine. American Academy of Pediatrics. Pediatrics 102: 1483-1491.

38. (1999) From the Centers for Disease Control and Prevention. Withdrawal of rotavirus vaccine recommendation. Jama 282: 2113-2114.

39. (1999) Rotavirus Gastroenteritis in Europe. Proceedings of the Workshop on Rotavirus Surveillance in Europe. Geneva, 28-29 October 1996. Acta Paediatr Suppl 88: 1-76.

40. (1999) Rotavirus vaccine for the prevention of rotavirus gastroenteritis among children. Recommendations of the Advisory Committee on Immunization Practices (ACIP). MMWR Recomm Rep 48: 1-20.

41. (1999) Rotavirus vaccines. Wkly Epidemiol Rec 74: 33-38.

42. (1999) Withdrawal of rotavirus vaccine recommendation. MMWR Morb Mortal Wkly Rep 48: 1007.

43. (2000) Foodborne outbreak of Group A rotavirus gastroenteritis among college students--District of Columbia, March-April 2000. MMWR Morb Mortal Wkly Rep 49: 1131-1133.

44. (2001) From the Centers for Disease Control and Prevention. Foodborne outbreak of group A rotavirus gastroenteritis among college students--District of Columbia, March-April 2000. Jama 285: 405-406.

45. (2003) FactSheet: Viral gastroenteritis. N S W Public Health Bull 14: 206-207.

46. (2003) Outbreak of severe rotavirus gastroenteritis among children--Jamaica, 2003. MMWR Morb Mortal Wkly Rep 52: 1103-1105.

47. (2004) [Diarrhea and rotavirus]. Rev Saude Publica 38: 844-845.

48. (2005) Hopes and fears for rotavirus vaccines. Lancet 365: 190.

49. (2006) [500,000 cases per year in Germany. There is now a vaccine for control of rotavirus infection]. MMW Fortschr Med 148: 6-7.

50. (2006) ACIP recommends new vaccine to prevent rotavirus. An update from CDC. Mich Med 105: 20.

51. (2006) [Approval of the first vaccine against rotavirus infections in Germany]. Kinderkrankenschwester 25: 289-290.

52. (2006) CDC's advisory committee recommends new vaccine to prevent rotavirus. Indian J Med Sci 60: 89-90.

53. (2006) Cost effectiveness of rotavirus vaccines and other interventions for diarrhoeal diseases: meeting report 2006. Wkly Epidemiol Rec 81: 350-353.

54. (2006) New rotavirus vaccine is likely on its way. Child Health Alert 24: 1.

55. (2006) New vaccine for infants. FDA Consum 40: 3.

56. (2006) The paediatric burden of rotavirus disease in Europe. Epidemiol Infect 134: 908-916.

57. (2006) RotaTeq: a new oral rotavirus vaccine. Med Lett Drugs Ther 48: 61-63.

58. (2006) [Vaccine against rotavirus]. Rev Saude Publica 40: 355-358.

59. (2007) Postmarketing monitoring of intussusception after RotaTeq vaccination--United States, February 1, 2006-February 15, 2007. MMWR Morb Mortal Wkly Rep 56: 218-222.

60. (2007) Prevention of rotavirus disease: guidelines for use of rotavirus vaccine. Pediatrics 119: 171-182.

61. (2007) Rotavirus vaccines: new drug. Rotavirus infection: one case of severe diarrhoea prevented per 100 vaccinated infants. Prescrire Int 16: 3-6.

62. (2008) Global networks for surveillance of rotavirus gastroenteritis, 2001-200. Wkly Epidemiol Rec 83: 421-425.

63. (2008) A new rotavirus vaccine. Med Lett Drugs Ther 50: 66-67.

64. (2008) Rotavirus surveillance--worldwide, 2001-2008. MMWR Morb Mortal Wkly Rep 57: 1255-1257.

65. (2008) Rotavirus vaccination coverage and adherence to the Advisory Committee on Immunization Practices (ACIP)-recommended vaccination schedule--United States, February 2006-May 2007. MMWR Morb Mortal Wkly Rep 57: 398-401.

66. (2008) [Rotaviruses "target" infants, noroviruses for seniors]. Kinderkrankenschwester 27: 342.

67. (2008) Statement on the recommended use of pentavalent human-bovine reassortant rotavirus vaccine. An Advisory Committee Statement (ACS). Can Commun Dis Rep 34: 1-33.

68. Abad FX, Villena C, Guix S, Caballero S, Pinto RM, et al. (2001) Potential role of fomites in the vehicular transmission of human astroviruses. Appl Environ Microbiol 67: 3904-3907.

69. Abbas AM, Denton MD (1987) An outbreak of rotavirus infection in a geriatric hospital. J Hosp Infect 9: 76-80.

70. Abbott MR, Cloonan MJ, Montgomery J, Smith DD (1984) Pathogens detected in the faeces of children with diarrhoea in a Sydney hospital. Med J Aust 141: 26-28.

71. Abdel-Haq NM, Thomas RA, Asmar BI, Zacharova V, Lyman WD (2003) Increased prevalence of G1P[4] genotype among children with rotavirus-associated gastroenteritis in metropolitan Detroit. J Clin Microbiol 41: 2680-2682.

72. Abdelmalek B (1987) [Total protein, urea and blood sugar in healthy calves and calves with diarrhea]. Vet Med Nauki 24: 55-58.

73. Abe T, Kobayashi M, Araki K, Kodama H, Fujita Y, et al. (2000) Infantile convulsions with mild gastroenteritis. Brain Dev 22: 301-306.

74. Abe Y, Inouye S (1979) Complement-fixing immunoglobulin M antibody response in patients with infantile gastroenteritis. J Clin Microbiol 9: 284-287.

75. Abid I, Guix S, Aouni M, Pinto R, Bosch A (2007) Detection and characterization of human group C rotavirus in the pediatric population of Barcelona, Spain. J Clin Virol 38: 78-82.

76. Abiodun PO, Ihongbe JC, Ogbimi A (1985) Asymptomatic rotavirus infection in Nigerian day-care centres. Ann Trop Paediatr 5: 163-165.

77. Abiodun PO, Omoigberale A (1994) Prevalence of nosocomial rotavirus infection in hospitalized children in Benin City, Nigeria. Ann Trop Paediatr 14: 85-88.

78. Abraham G, Roeder PL, Zewdu R (1992) Agents associated with neonatal diarrhoea in Ethiopian dairy calves. Trop Anim Health Prod 24: 74-80.

79. Abraham R, Ogra PL (1994) Mucosal microenvironment and mucosal response. Am J Trop Med Hyg 50: 3-9.

80. Abrahamsen TG, Kjeldsberg E, Mortensson-Egnund K, Anestad G (1989) [Rotavirus in stools from hospitalized children]. Tidsskr Nor Laegeforen 109: 3419-3420.

81. Abrams SA, Hilmers DC (2007) Improving rehydration solutions with human milk proteins: are the benefits worth the challenges? J Pediatr Gastroenterol Nutr 44: 298-299.

82. Abu-Elamreen FH, Abed AA, Sharif FA (2008) Viral, bacterial and parasitic etiology of pediatric diarrhea in Gaza, Palestine. Med Princ Pract 17: 296-301.

83. Acosta-Martinez F, Gyles CL, Butler DG (1980) Escherichia coli heat-stable enterotoxin in feces and intestines of calves with diarrhea. Am J Vet Res 41: 1143-1149.

84. Acres SD, Babiuk LA (1978) Studies on rotaviral antibody in bovine serum and lacteal secretions, using radioimmunoassay. J Am Vet Med Assoc 173: 555-559.

85. Adah MI, Jaji Z, Agwazim BF, el-Yuguda AD, Mani AU (2002) Detection of human rotavirus in faeces from diarrhoeic calves in north-east Nigeria. Trop Anim Health Prod 34: 1-6.

86. Adah MI, Nagashima S, Wakuda M, Taniguchi K (2003) Close relationship between G8-serotype bovine and human rotaviruses isolated in Nigeria. J Clin Microbiol 41: 3945-3950.

87. Adah MI, Rohwedder A, Olaleye OD, Durojaiye OA, Werchau H (1997) Further characterization of field strains of rotavirus from Nigeria VP4 genotype P6 most frequently identified among symptomatically infected children. J Trop Pediatr 43: 267-274.

88. Adah MI, Rohwedder A, Olaleye OD, Durojaiye OA, Werchau H (1997) Serotype of Nigerian rotavirus strains. Trop Med Int Health 2: 363-370.

89. Adah MI, Rohwedder A, Olaleye OD, Werchau H (1996) Sequence analysis of VP7 gene of two Nigerian rotavirus strains. Acta Virol 40: 187-193.

90. Adah MI, Wade A, Oseto M, Kuzuya M, Taniguchi K (2002) Detection of human group C rotaviruses in Nigeria and sequence analysis of their genes encoding VP4, VP6, and VP7 proteins. J Med Virol 66: 269-275.

91. Adah MI, Wade A, Taniguchi K (2001) Molecular epidemiology of rotaviruses in Nigeria: detection of unusual strains with G2P[6] and G8P[1] specificities. J Clin Microbiol 39: 3969-3975.

92. Adesiyun AA, Kaminjolo JS, Ngeleka M, Mutani A, Borde G, et al. (2001) A longitudinal study on enteropathogenic infections of livestock in Trinidad. Rev Soc Bras Med Trop 34: 29-35.

93. Adhikari M, Coovadia Y, Hewitt J (1985) Enteropathogenic Escherichia coli (EPEC) and enterotoxigenic (ETEC) related diarrhoeal disease in a neonatal unit. Ann Trop Paediatr 5: 19-22.

94. Adhikary AK, Zhou Y, Kakizawa J, Numaga J, Akihara S, et al. (1998) Distribution of rotavirus VP4 genotype and VP7 serotype among Chinese children. Acta Paediatr Jpn 40: 641-643.

95. Adkins H, Merrell B, O'Rourke T, Echeverria P (1990) Travelers' diarrhea among U.S. Navy and Marine Corps personnel during a Western Pacific deployment. Mil Med 155: 111-116.

96. Adkins HJ, Escamilla J, Echeverria P, Santiago LT, Ranoa CP, et al. (1989) Symptoms associated with diarrhoeal illness at San Lazaro Hospital, Manila in 1983 and 1984. Southeast Asian J Trop Med Public Health 20: 207-214.

97. Adkins HJ, Escamilla J, Santiago LT, Ranoa C, Echeverria P, et al. (1987) Two-year survey of etiologic agents of diarrheal disease at San Lazaro Hospital, Manila, Republic of the Philippines. J Clin Microbiol 25: 1143-1147.

98. Adler A, Wollach B, Kilman A, Gottesman G (2005) Enteric gram-negative sepsis complicating rotavirus gastroenteritis in previously healthy infants. Clin Pediatr (Phila) 44: 351-354.

99. Adler M, Schulz S, Fischer R, Niemeyer CM (2005) Detection of Rotavirus from stool samples using a standardized immuno-PCR ("Imperacer") method with end-point and real-time detection. Biochem Biophys Res Commun 333: 1289-1294.

100. Adrian T, Wigand R, Richter J (1987) Gastroenteritis in infants, associated with a genome type of adenovirus 31 and with combined rotavirus and adenovirus 31 infection. Eur J Pediatr 146: 38-40.

101. Afset JE, Bergh K, Bevanger L (2003) High prevalence of atypical enteropathogenic Escherichia coli (EPEC) in Norwegian children with diarrhoea. J Med Microbiol 52: 1015-1019.

102. Afshar A, Tadayon RA (1979) Rotavirus in diarrhoeic calves in Iran. Vet Rec 105: 400.

103. Agboatwalla M, Isomura S, Akram DS, Isihara Y, Sakae K, et al. (1995) Enteric viral infections in pre-school children in Karachi, Pakistan. Indian J Pediatr 62: 345-351.

104. Aggarwal P, Singh M, Guha DK (1988) Prevalence of bacterial pathogens and rotavirus in hospitalised children with acute diarrhoea in Delhi, India. J Diarrhoeal Dis Res 6: 37-38.

105. Aggarwal P, Srivastav VK, Singh M, Khanna KK (1988) Rotavirus shown to be the main cause of acute childhood diarrhoea in a New Delhi hospital with a high prevalence in winter. J Diarrhoeal Dis Res 6: 39-40.

106. Agnello D, Herve CA, Lavaux A, Darniot M, Guillon P, et al. (2006) Intrarectal immunization with rotavirus 2/6 virus-like particles induces an antirotavirus immune response localized in the intestinal mucosa and protects against rotavirus infection in mice. J Virol 80: 3823-3832.

107. Ahmed F, Jones DB, Jackson AA (1991) Effect of severe food restriction on the gut following rotavirus infection in mice. Ann Nutr Metab 35: 158-164.

108. Ahmed F, Jones DB, Jackson AA (1991) Effect of vitamin A deficiency on the immune response to epizootic diarrhoea of infant mice (EDIM) rotavirus infection in mice. Br J Nutr 65: 475-485.

109. Ahmed HM, Coulter JB, Nakagomi O, Hart CA, Zaki JM, et al. (2006) Molecular characterization of rotavirus gastroenteritis strains, Iraqi Kurdistan. Emerg Infect Dis 12: 824-826.

110. Ahmed K, Anh DD, Nakagomi O (2007) Rotavirus G5P[6] in child with diarrhea, Vietnam. Emerg Infect Dis 13: 1232-1235.

111. Ahmed K, Nakagomi T, Nakagomi O (2005) Isolation and molecular characterization of a naturally occurring non-structural protein 5 (NSP5) gene reassortant of group A rotavirus of serotype G2P[4] with a long RNA pattern. J Med Virol 77: 323-330.

112. Ahmed MU, Alam MM, Chowdhury NS, Haque MM, Shahid N, et al. (1999) Analysis of human rotavirus G serotype in Bangladesh by enzyme-linked immunosorbent assay and polymerase chain reaction. J Diarrhoeal Dis Res 17: 22-27.

113. Ahmed MU, Kobayashi N, Wakuda M, Sanekata T, Taniguchi K, et al. (2004) Genetic analysis of group B human rotaviruses detected in Bangladesh in 2000 and 2001. J Med Virol 72: 149-155.

114. Ahmed MU, Taniguchi K, Kobayashi N, Urasawa T, Wakasugi F, et al. (1989) Characterization by enzyme-linked immunosorbent assay using subgroup- and serotype-specific monoclonal antibodies of human rotavirus obtained from diarrheic patients in Bangladesh. J Clin Microbiol 27: 1678-1681.

115. Ahmed MU, Urasawa S, Taniguchi K, Urasawa T, Kobayashi N, et al. (1991) Analysis of human rotavirus strains prevailing in Bangladesh in relation to nationwide floods brought by the 1988 monsoon. J Clin Microbiol 29: 2273-2279.

116. Ahmed T, Sumazaki R, Shin K, Nagai Y, Shibasaki M, et al. (1998) Humoral immune and clinical responses to food antigens following acute diarrhoea in children. J Paediatr Child Health 34: 229-232.

117. Ahmetagic S, Jusufovic E, Petrovic J, Stojic V, Delibegovic Z (2003) Acute infectious diarrhea in children. Med Arh 57: 87-92.

118. Aich P, Wilson HL, Kaushik RS, Potter AA, Babiuk LA, et al. (2007) Comparative analysis of innate immune responses following infection of newborn calves with bovine rotavirus and bovine coronavirus. J Gen Virol 88: 2749-2761.

119. Aidara A, Gentile B, Rogier C, Wane H (1993) [Bacterial, viral and parasitic etiologies of acute infantile diarrhea in a rural Senegal]. Dakar Med 38: 187-191.

120. Aihara M, Sakai M, Iwasaki M, Shimakawa K, Kozaki S, et al. (1991) [Isolation of enteropathogenic microorganism from patients with infection of the digestive tract during 1976 to 1988 in Tenri Hospital]. Kansenshogaku Zasshi 65: 864-874.

121. Aijaz S, Gowda K, Jagannath HV, Reddy RR, Maiya PP, et al. (1996) Epidemiology of symptomatic human rotaviruses in Bangalore and Mysore, India, from 1988 to 1994 as determined by electropherotype, subgroup and serotype analysis. Arch Virol 141: 715-726.

122. Aithala G, Al Dhahry SH, Saha A, Elbualy MS (1996) Epidemiological and clinical features of rotavirus gastroenteritis in Oman. J Trop Pediatr 42: 54-57.

123. Aiyar J, Bhan MK, Bhandari N, Kumar R, Raj P, et al. (1990) Rotavirus-specific antibody response in saliva of infants with rotavirus diarrhea. J Infect Dis 162: 1383-1384.

124. Ajjampur SS, Rajendran P, Ramani S, Banerjee I, Monica B, et al. (2008) Closing the diarrhoea diagnostic gap in Indian children by the application of molecular techniques. J Med Microbiol 57: 1364-1368.

125. Akhter J, Burdette JM, Qadri SM, Myint SH (1994) Aetiology of gastroenteritis at a major referral centre in Saudi Arabia. J Int Med Res 22: 47-54.

126. Akhter J, Sikotra S, Qadri SM, Myint SH (1994) Comparison of paediatric viral gastroenteritis at large medical centres in Saudi Arabia and the United Kingdom. J Diarrhoeal Dis Res 12: 257-260.

127. Akihara S, Phan TG, Nguyen TA, Hansman G, Okitsu S, et al. (2005) Existence of multiple outbreaks of viral gastroenteritis among infants in a day care center in Japan. Arch Virol 150: 2061-2075.

128. Akinci A, Tezic T, Gur I, Cetin H, Hatun S (1991) Rotavirus diarrhea in newborn infants. Turk J Pediatr 33: 153-157.

129. Akoua-Koffi C, Akran V, Peenze I, Adjogoua V, de Beer MC, et al. (2007) [Epidemiological and virological aspects Rotavirus diarrhoea in Abidjan, Cote d'Ivoire (1997-2000)]. Bull Soc Pathol Exot 100: 246-249.

130. Akran V, Akoua-Koffi C, Kette H, Dosso M (2001) Electropherotypes of Rotaviruses in Children less than 5 years old in Abidjan, Cote D'ivoire in 1997. Afr J Health Sci 8: 33-38.

131. Al-Ahdal MN, Qadri SM, Al-Dayel F, Khan GY, Cunha BA (1991) Incidence of rotaviral gastroenteritis at a referral centre in Saudi Arabia. Ann Saudi Med 11: 19-22.

132. Alain S, Denis F (2007) [Epidemiology of infectious acute diarrhoea in France and Europe]. Arch Pediatr 14 Suppl 3: S132-144.

133. Alam MM, Ahmed MU, Chowdhury NS, Urasawa S (1999) Detection of group- and subgroup-specific antigens of bovine rotaviruses in Bangladesh. J Diarrhoeal Dis Res 17: 81-84.

134. Alam MM, Kobayashi N, Ishino M, Ahmed MS, Ahmed MU, et al. (2007) Genetic analysis of an ADRV-N-like novel rotavirus strain B219 detected in a sporadic case of adult diarrhea in Bangladesh. Arch Virol 152: 199-208.

135. Alam NH, Ashraf H (2003) Treatment of infectious diarrhea in children. Paediatr Drugs 5: 151-165.

136. Alarcon Menendez P, Alarcon Mangini JL (1994) [Proposal for a practical diagnostic management of the etiology of acute childhood diarrhea]. Rev Gastroenterol Peru 14: 145-149.

137. Albano F, Bruzzese E, Bella A, Cascio A, Titone L, et al. (2007) Rotavirus and not age determines gastroenteritis severity in children: a hospital-based study. Eur J Pediatr 166: 241-247.

138. Albert MJ, Bhat P, Rajan D, Maiya PP, Pereira SM, et al. (1978) Jejunal microbial flora of southern indian infants in health and with acute gastroenteritis. J Med Microbiol 11: 433-440.

139. Albert MJ, Bishop RF (1984) Cultivation of human rotaviruses in cell culture. J Med Virol 13: 377-383.

140. Albert MJ, Bishop RF, Shann FA (1983) Epidemiology of rotavirus diarrhea in the Highlands of Papua, New Guinea, in 1979, as revealed by electrophoresis of genome RNA. J Clin Microbiol 17: 162-164.

141. Albert MJ, Faruque AS, Faruque SM, Sack RB, Mahalanabis D (1999) Case-control study of enteropathogens associated with childhood diarrhea in Dhaka, Bangladesh. J Clin Microbiol 37: 3458-3464.

142. Albert MJ, Soenarto Y, Bishop RF (1982) Epidemiology of rotavirus diarrhea in Yogyakarta, Indonesia, as revealed by electrophoresis of genome RNA. J Clin Microbiol 16: 731-733.

143. Albert MJ, Unicomb LE, Barnes GL, Bishop RF (1987) Cultivation and characterization of rotavirus strains infecting newborn babies in Melbourne, Australia, from 1975 to 1979. J Clin Microbiol 25: 1635-1640.

144. Albert MJ, Unicomb LE, Tzipori SR, Bishop RF (1987) Isolation and serotyping of animal rotaviruses and antigenic comparison with human rotaviruses. Brief report. Arch Virol 93: 123-130.

145. Albrecht H, Stellbrink HJ, Fenske S, Ermer M, Raedler A, et al. (1993) Rotavirus antigen detection in patients with HIV infection and diarrhea. Scand J Gastroenterol 28: 307-310.

146. Albrey MB, Murphy AM (1976) Rotaviruses and acute gastroenteritis of infants and children. Med J Aust 1: 82-85.

147. al-Bwardy MA, Ramia S, al-Frayh AR, Chagla AH, al-Omair AA, et al. (1988) Bacterial, parasitic and viral enteropathogens associated with diarrhoea in Saudi children. Ann Trop Paediatr 8: 26-30.

148. Alfieri AA, Leite JP, Alfieri AF, Jiang B, Glass RI, et al. (1999) Detection of field isolates of human and animal group C rotavirus by reverse transcription-polymerase chain reaction and digoxigenin-labeled oligonucleotide probes. J Virol Methods 83: 35-43.

149. Alfieri AA, Leite JP, Nakagomi O, Kaga E, Woods PA, et al. (1996) Characterization of human rotavirus genotype P[8]G5 from Brazil by probe-hybridization and sequence. Arch Virol 141: 2353-2364.

150. Alfieri AA, Parazzi ME, Takiuchi E, Medici KC, Alfieri AF (2006) Frequency of group A rotavirus in diarrhoeic calves in Brazilian cattle herds, 1998-2002. Trop Anim Health Prod 38: 521-526.

151. Alfieri AF, Alfieri AA, Barreiros MA, Leite JP, Richtzenhain LJ (2004) G and P genotypes of group A rotavirus strains circulating in calves in Brazil, 1996-1999. Vet Microbiol 99: 167-173.

152. Al-Frayh AR, Ramia S, Bakir TM, Zaidi MA (1987) Rotavirus shedding by neonates and possible modes of transmission. J Trop Pediatr 33: 246-248.

153. Al-Gallas N, Bahri O, Bouratbeen A, Ben Haasen A, Ben Aissa R (2007) Etiology of acute diarrhea in children and adults in Tunis, Tunisia, with emphasis on diarrheagenic Escherichia coli: prevalence, phenotyping, and molecular epidemiology. Am J Trop Med Hyg 77: 571-582.

154. Ali A, Bingnan F, Unicomb LE, Rahim Z, Hossain A, et al. (1993) Evaluation of a probe hybridisation serotyping method for group A rotavirus. J Diarrhoeal Dis Res 11: 153-156.

155. Ali MB, Ghenghesh KS, Aissa RB, Abuhelfaia A, Dufani M (2005) Etiology of childhood diarrhea in Zliten, Libya. Saudi Med J 26: 1759-1765.

156. Ali NK, Bhutta ZA (2003) A review of rotavirus diarrhea in Pakistan: how much do we know? J Coll Physicians Surg Pak 13: 297-301.

157. Allen S, Mitchell J, Jones W, Quinn M (1989) A novel bovine rotavirus electropherotype from outbreaks of neonatal diarrhea in Utah beef herds. J Vet Diagn Invest 1: 74-75.

158. Allen SJ, Okoko B, Martinez E, Gregorio G, Dans LF (2004) Probiotics for treating infectious diarrhoea. Cochrane Database Syst Rev: CD003048.

159. Allerberger F, Rossboth D, Dierich MP, Aleksic S, Schmidt H, et al. (1996) Prevalence and clinical manifestations of Shiga toxin-producing Escherichia coli infections in Austrian children. Eur J Clin Microbiol Infect Dis 15: 545-550.

160. Allwinn R, Janz B, Doerr HW (2008) [Viral gastroenteritis. An epidemiologic investigation between the period 2001-2006]. Med Klin (Munich) 103: 389-395.

161. Al-Mashhadani MN, Nakagomi O, Dove W, Ahmed H, Nakagomi T, et al. (2008) Norovirus gastroenteritis among children in Iraqi Kurdistan. J Med Virol 80: 506-509.

162. Almeida JD, Craig CR, Hall TE (1978) Multiple viruses present in the faeces of a scouring calf. Vet Rec 102: 170-171.

163. Almeida JD, Hall T, Banatvala JE, Totterdell BM, Chrystie IL (1978) The effect of trypsin on the growth of rotavirus. J Gen Virol 40: 213-218.

164. Al-Nakib W, Chrystie IL, Banatvala JE, Al-Sayegh F (1980) Rotavirus and non-bacterial infantile gastroenteritis in Kuwait. Int J Epidemiol 9: 355-359.

165. Alonso Franch M, Cilleruelo ML, Tornel J, Ortiz de Lejarazu R, Sanchez Villares E (1983) [Current treatment and prevention of juvenile gastroenteritis]. An Esp Pediatr 19 Suppl 18: 47-60.

166. Alpert G, Bell LM, Kirkpatrick CE, Budnick LD, Campos JM, et al. (1986) Outbreak of cryptosporidiosis in a day-care center. Pediatrics 77: 152-157.

167. Altindis M, Yavru S, Simsek A, Ozkul A, Ceri A, et al. (2004) Rotavirus infection in children with acute diarrhea as detected by latex agglutination, ELISA and polyacrylamide gel electrophoresis. Indian Pediatr 41: 590-594.

168. Alvarado-Aleman F, Guardo-Bustillo C, Galindo E, Mendez-Tena E, Alvarado-Gonzalez S, et al. (1985) [Frequency of enteropathogenic micro-organisms isolated in children with and without acute diarrhea]. Bol Med Hosp Infant Mex 42: 354-359.

169. Alvarez JO, Salazar-Lindo E, Kohatsu J, Miranda P, Stephensen CB (1995) Urinary excretion of retinol in children with acute diarrhea. Am J Clin Nutr 61: 1273-1276.

170. Alvarez V, Barron BL, Benitez O, Herrada R, Miramontes M, et al. (1991) Short electropherotype rotaviruses isolated longitudinally from a cohort of Mexican infants. Rev Latinoam Microbiol 33: 55-60.

171. Alvarez Vega M, Guzman Tirado MG, Mas Lago P (1989) [Comparison of latex agglutination technics and polyacrylamide gel electrophoresis for the rotavirus diagnosis. Habana City, 1986]. Rev Cubana Med Trop 41: 385-397.

172. Alvarez-Munoz MT, Guiscafre-Gallardo JP, Mondragon-Sanchez C, Morales-Castillo ME, Ruiz-Gomez J, et al. (1982) Comparison between viral RNA electrophoresis, ELISA and complement fixation techniques with electronic microscopy to demonstrate rotavirus. Arch Invest Med (Mex) 13: 145-150 passim.

173. Alvarez-Munoz MT, Ruiz-Gomez J, Palacios-Trevino J, Morales-Castillo ME, Cedillo R (1983) [Incidence and types of rotavirus in relation to the age of the patients and different times of the year]. Gac Med Mex 119: 330-333.

174. Al-Yousif Y, Al-Majhdi F, Chard-Bergstrom C, Anderson J, Kapil S (2000) Development, characterization, and diagnostic applications of monoclonal antibodies against bovine rotavirus. Clin Diagn Lab Immunol 7: 288-292.

175. Al-Yousif Y, Anderson J, Chard-Bergstrom C, Bustamante A, Muenzenberger M, et al. (2001) Evaluation of a latex agglutination kit (Virogen Rotatest) for detection of bovine rotavirus in fecal samples. Clin Diagn Lab Immunol 8: 496-498.

176. Al-Yousif Y, Anderson J, Chard-Bergstrom C, Kapil S (2002) Development, evaluation, and application of lateral-flow immunoassay (immunochromatography) for detection of rotavirus in bovine fecal samples. Clin Diagn Lab Immunol 9: 723-725.

177. Amador JJ, Vicari A, Turcios-Ruiz RM, Melendez DA, Malek M, et al. (2008) Outbreak of rotavirus gastroenteritis with high mortality, Nicaragua, 2005. Rev Panam Salud Publica 23: 277-284.

178. Amar CF, East CL, Gray J, Iturriza-Gomara M, Maclure EA, et al. (2007) Detection by PCR of eight groups of enteric pathogens in 4,627 faecal samples: re-examination of the English case-control Infectious Intestinal Disease Study (1993-1996). Eur J Clin Microbiol Infect Dis 26: 311-323.

179. Amarilla A, Espinola EE, Galeano ME, Farina N, Russomando G, et al. (2007) Rotavirus infection in the Paraguayan population from 2004 to 2005: high incidence of rotavirus strains with short electropherotype in children and adults. Med Sci Monit 13: CR333-337.

180. Amer AA, el-Mougi M, Hughes J, el-Tayyeb S, el-Abhar A, et al. (1990) Comparison of latex agglutination test with an ELISA to diagnose rotavirus-associated diarrhoea in infants and young children. J Diarrhoeal Dis Res 8: 87-89.

181. Amini S, Solati AA, Fayaz A, Mahmoodi M (1990) Rotavirus infection in children with acute diarrhea in Tehran. Med J Islam Repub Iran 4: 25-28.

182. Aminu M, Ahmad AA, Umoh JU (2008) Rotavirus infection in four states in north-western Nigeria. Niger J Med 17: 285-290.

183. Aminu M, Ahmad AA, Umoh JU, Dewar J, Esona MD, et al. (2008) Epidemiology of rotavirus infection in north-western Nigeria. J Trop Pediatr 54: 340-342.

184. Anagonou SY, Koumakpai S, Josse R, Massougbodji A, Sadeler BC, et al. (1993) [Rotavirus gastroenteritis in a pediatric service at the National University Hospital Center of Cotonou (Benin)]. Med Trop (Mars) 53: 105-107.

185. Anand T, Lakshmi N, Kumar AG (1994) Rota virus diarrhea among infants and children at Tirupati. Indian Pediatr 31: 46-48.

186. Anand T, Raju TA, Rao MV, Rao LV, Sharma G (2000) Symptomatic human rotavirus subgroups, serotypes & electropherotypes in Hyderabad, India. Indian J Med Res 112: 1-4.

187. Ananthan S, Saravanan P (1998) Analysis of human rotavirus serotypes in children with acute diarrhoea in Chennai by monoclonal antibody based ELISA. Indian J Med Res 108: 58-61.

188. Ananthan S, Saravanan P (2000) Genomic diversity of group A rotavirus RNA from children with acute diarrhoea in Chennai, south India. Indian J Med Res 111: 50-56.

189. Anderson EJ (2007) Rotavirus G9 severity data revisited. Clin Infect Dis 44: 154-155; author reply 155.

190. Anderson EJ (2008) Rotavirus vaccines: viral shedding and risk of transmission. Lancet Infect Dis 8: 642-649.

191. Anderson EJ, Weber SG (2004) Rotavirus infection in adults. Lancet Infect Dis 4: 91-99.

192. Anderson EL, Belshe RB, Bartram J, Crookshanks-Newman F, Chanock RM, et al. (1986) Evaluation of rhesus rotavirus vaccine (MMU 18006) in infants and young children. J Infect Dis 153: 823-831.

193. Andrade GP, Lima LR, Hoshino-Shimizu S, Oliveira MI, Mendonca RZ, et al. (1996) Humoral immunity patterns based on antibody reactivity to rotavirus antigens in Brazilian children under 5 years of age. J Med Virol 49: 212-217.

194. Andreasi MS, Batista SM, Tozetti IA, Ozaki CO, Nogueira MM, et al. (2007) [Rotavirus A among hospitalized infants, up to three years of age, with acute gastroenteritis in Campo Grande, State of Mato Grosso do Sul]. Rev Soc Bras Med Trop 40: 411-414.

195. Andreasi MS, Cardoso DD, Fernandes SM, Tozetti IA, Borges AM, et al. (2008) Adenovirus, calicivirus and astrovirus detection in fecal samples of hospitalized children with acute gastroenteritis from Campo Grande, MS, Brazil. Mem Inst Oswaldo Cruz 103: 741-744.

196. Andres A, Donovan SM, Kuhlenschmidt TB, Kuhlenschmidt MS (2007) Isoflavones at concentrations present in soy infant formula inhibit rotavirus infection in vitro. J Nutr 137: 2068-2073.

197. Andrew ME, Boyle DB, Coupar BE, Reddy D, Bellamy AR, et al. (1992) Vaccinia-rotavirus VP7 recombinants protect mice against rotavirus-induced diarrhoea. Vaccine 10: 185-191.

198. Angel J, Franco MA, Greenberg HB (2007) Rotavirus vaccines: recent developments and future considerations. Nat Rev Microbiol 5: 529-539.

199. Angel J, Franco MA, Greenberg HB, Bass D (1999) Lack of a role for type I and type II interferons in the resolution of rotavirus-induced diarrhea and infection in mice. J Interferon Cytokine Res 19: 655-659.

200. Angel J, Tang B, Feng N, Greenberg HB, Bass D (1998) Studies of the role for NSP4 in the pathogenesis of homologous murine rotavirus diarrhea. J Infect Dis 177: 455-458.

201. Angeretti A, Magi MT, Merlino C, Ferrara B, Negro Ponzi A (1987) Specific serum IgA in rotavirus gastroenteritis. J Med Virol 23: 345-349.

202. Anh DD, Thiem VD, Fischer TK, Canh DG, Minh TT, et al. (2006) The burden of rotavirus diarrhea in Khanh Hoa Province, Vietnam: baseline assessment for a rotavirus vaccine trial. Pediatr Infect Dis J 25: 37-40.

203. Annalisa P, Antonella B, Claudio V, Giampaolo R, Massimo M (1988) Papular or papulovesicular syndromes. Arch Dermatol 124: 1444-1445.

204. Ansaldi F, Lai P, Valle L, Riente R, Durando P, et al. (2008) Burden of rotavirus-associated and non-rotavirus-associated diarrhea among nonhospitalized individuals in central Italy: a 1-year sentinel-based epidemiological and virological surveillance. Clin Infect Dis 46: e51-55.

205. Ansari SA, Springthorpe VS, Sattar SA (1991) Survival and vehicular spread of human rotaviruses: possible relation to seasonality of outbreaks. Rev Infect Dis 13: 448-461.

206. Antsupova AS, Al'tova EE, Zalesskikh AF, Epifanova NV, Dombrovskaia LK (1988) [Data on the epidemiology of rotavirus infection]. Zh Mikrobiol Epidemiol Immunobiol: 34-37.

207. Antsupova AS, Trofimova MN, Epifanova NV, Troitskaia MV (1984) [Electron microscopic diagnosis of viral diseases]. Vopr Virusol 29: 316-319.

208. Anvikar AR, Dolla C, Dutta S, Rao VG, Gadge VS, et al. (2008) Role of Escherichia coli in acute diarrhoea in tribal preschool children of central India. Paediatr Perinat Epidemiol 22: 40-46.

209. Anvikar AR, Dolla C, Dutta S, Rao VG, Gadge VS, et al. (2008) Role of Escherichia coli in acute diarrhoea in tribal preschool children of central India. Paediatr Perinat Epidemiol 22: 40-46.

210. Appleton H, Buckley M, Robertson MH, Thom BT (1978) A search for faecal viruses in new-born and other infants. J Hyg (Lond) 81: 279-283.

211. Araki K, Kobayashi S, Utagawa E, Kobayashi M, Shinozaki T, et al. (1998) [Prevalence of human astrovirus serotypes in Shizuoka 1991-96]. Kansenshogaku Zasshi 72: 12-16.

212. Araki K, Shinozaki T, Irie Y, Miyazawa Y (1999) [Trial of oral administration of Bifidobacterium breve for the prevention of rotavirus infections]. Kansenshogaku Zasshi 73: 305-310.

213. Araujo EC, Clemens SA, Oliveira CS, Justino MC, Rubio P, et al. (2007) Safety, immunogenicity, and protective efficacy of two doses of RIX4414 live attenuated human rotavirus vaccine in healthy infants. J Pediatr (Rio J) 83: 217-224.

214. Araujo IT, Assis RM, Fialho AM, Mascarenhas JD, Heinemann MB, et al. (2007) Brazilian P[8],G1, P[8],G5, P[8],G9, and P[4],G2 rotavirus strains: nucleotide sequence and phylogenetic analysis. J Med Virol 79: 995-1001.

215. Araujo IT, Ferreira MS, Fialho AM, Assis RM, Cruz CM, et al. (2001) Rotavirus genotypes P[4]G9, P[6]G9, and P[8]G9 in hospitalized children with acute gastroenteritis in Rio de Janeiro, Brazil. J Clin Microbiol 39: 1999-2001.

216. Araujo IT, Fialho AM, de Assis RM, Rocha M, Galvao M, et al. (2002) Rotavirus strain diversity in Rio de Janeiro, Brazil: characterization of VP4 and VP7 genotypes in hospitalized children. J Trop Pediatr 48: 214-218.

217. Araujo IT, Heinemann MB, Mascarenhas JD, Assis RM, Fialho AM, et al. (2007) Molecular analysis of the NSP4 and VP6 genes of rotavirus strains recovered from hospitalized children in Rio de Janeiro, Brazil. J Med Microbiol 56: 854-859.

218. Araya M, Figueroa G, Espinoza J, Zarur X, Brunser O (1986) Acute diarrhoea and asymptomatic infection in Chilean preschoolers of low and high socio-economic strata. Acta Paediatr Scand 75: 645-651.

219. Araya M, Spencer E, Brunser O, Espinoza J, Sandino AM (1985) [Comparative study of 2 methods in the diagnosis of rotaviruses in infants with acute and asymptomatic diarrhea]. Rev Chil Pediatr 56: 442-445.

220. Arcangeletti MC, De Conto F, Pinardi F, Medici MC, Valcavi P, et al. (2005) Electron microscopy as a reliable tool for rapid and conventional detection of enteric viral agents: a five-year experience report. Acta Biomed 76: 165-170.

221. Archambault D, Morin G, Elazhary Y, Roy RS (1990) Study of virus excretion in feces of diarrheic and asymptomatic calves infected with rotavirus. Zentralbl Veterinarmed B 37: 73-76.

222. Archambault D, Roy RS, Dea S, Elazhary MA (1984) Comparative study of bovine rotavirus isolates by plaque assay. Can J Comp Med 48: 286-289.

223. Ardern S, Lennon D (1997) Rotavirus gastroenteritis: is vaccine prevention near at hand? N Z Med J 110: 407-409.

224. Ardern-Holmes SL, Lennon D, Pinnock R, Nicholson R, Graham D, et al. (1999) Trends in hospitalization and mortality from rotavirus disease in New Zealand infants. Pediatr Infect Dis J 18: 614-619.

225. Arguelles MH, Villegas GA, Castello A, Abrami A, Ghiringhelli PD, et al. (2000) VP7 and VP4 genotyping of human group A rotavirus in Buenos Aires, Argentina. J Clin Microbiol 38: 252-259.

226. Arias C, Lopez S, Espejo R (1982) Identification of the RNA segments encoding some structural polypeptides of Nebraska Calf Diarrhea virus. Rev Latinoam Microbiol 24: 47-54.

227. Arias CF, Dector MA, Segovia L, Lopez T, Camacho M, et al. (2004) RNA silencing of rotavirus gene expression. Virus Res 102: 43-51.

228. Arias CF, Isa P, Guerrero CA, Mendez E, Zarate S, et al. (2002) Molecular biology of rotavirus cell entry. Arch Med Res 33: 356-361.

229. Arias CF, Lopez S, Mascarenhas JD, Romero P, Cano P, et al. (1994) Neutralizing antibody immune response in children with primary and secondary rotavirus infections. Clin Diagn Lab Immunol 1: 89-94.

230. Arias Vallejo E (1986) [Acute rotavirus enteritis]. Rev Esp Enferm Apar Dig 69: 351-352.

231. Arias Vallejo E (1986) [Cow's milk, in the therapy of rotavirus enteritis]. Rev Esp Enferm Apar Dig 70: 63.

232. Arif M, El-Hazmi MM (2005) Viral gastroenteritis in Saudi children. Saudi Med J 26: 1017-1018.

233. Arikan C, Arslan MT, Kilic M, Aydogdu S (2006) Transient hyperphosphatasemia after pediatric liver transplantation. Pediatr Int 48: 390-392.

234. Arista S, Di Stefano R, Giovannelli L, Sinatra A (1983) [Immunofluorescence reaction for rotavirus analysis in fecal specimens]. Quad Sclavo Diagn 19: 499-506.

235. Arista S, Gioeli MT, Titone L (1981) [Rotaviruses as causal agents of acute gastroenteritis. Virological and serological studies in samples of the population of Palermo]. Ann Sclavo 23: 337-346.

236. Arista S, Giovannelli L, Balsano M, Di Stefano R (1984) [Comparison of diagnostic methods in rotavirus infection: immunofluorescence, ELISA and the latex agglutination test]. Quad Sclavo Diagn 20: 258-263.

237. Arista S, Giovannelli L, Pistoia D, Cascio A, Parea M, et al. (1990) Electropherotypes, subgroups and serotypes of human rotavirus strains causing gastroenteritis in infants and young children in Palermo, Italy, from 1985 to 1989. Res Virol 141: 435-448.

238. Arista S, Vizzi E, Ferraro D, Cascio A, Di Stefano R (1997) Distribution of VP7 serotypes and VP4 genotypes among rotavirus strains recovered from Italian children with diarrhea. Arch Virol 142: 2065-2071.

239. Arista S, Vizzi E, Migliore MC, Di Rosa E, Cascio A (2003) High incidence of G9P181 rotavirus infections in Italian children during the winter season 1999-2000. Eur J Epidemiol 18: 711-714.

240. Armah GE, Gallimore CI, Binka FN, Asmah RH, Green J, et al. (2006) Characterisation of norovirus strains in rural Ghanaian children with acute diarrhoea. J Med Virol 78: 1480-1485.

241. Armah GE, Hori H, Anyanful A, Addo JA, Commey JO, et al. (1995) Human rotavirus subgroups and severity of associated diarrhoea in Ghana. Afr J Health Sci 2: 388-391.

242. Armah GE, Mingle JA, Dodoo AK, Anyanful A, Antwi R, et al. (1994) Seasonality of rotavirus infection in Ghana. Ann Trop Paediatr 14: 223-229.

243. Armah GE, Pager CT, Asmah RH, Anto FR, Oduro AR, et al. (2001) Prevalence of unusual human rotavirus strains in Ghanaian children. J Med Virol 63: 67-71.

244. Armah GE, Steele AD, Binka FN, Esona MD, Asmah RH, et al. (2003) Changing patterns of rotavirus genotypes in ghana: emergence of human rotavirus G9 as a major cause of diarrhea in children. J Clin Microbiol 41: 2317-2322.

245. Aronsson B, Elinder G, Thorstrand C (1992) [Is hypertonic dehydration increasing among children with gastroenteritis?]. Lakartidningen 89: 2268-2269.

246. Arslan H, Inci EK, Azap OK, Karakayali H, Torgay A, et al. (2007) Etiologic agents of diarrhea in solid organ recipients. Transpl Infect Dis 9: 270-275.

247. Arslanagic E, Kruzic V, Kacic M, Skelin M (1984) [An electron microscopy study of the causes of acute gastroenteritis in children]. Lijec Vjesn 106: 175-179.

248. Arthur JD, Bodhidatta L, Echeverria P, Phuphaisan S, Paul S (1992) Diarrheal disease in Cambodian children at a camp in Thailand. Am J Epidemiol 135: 541-551.

249. Arvola T, Laiho K, Torkkeli S, Mykkanen H, Salminen S, et al. (1999) Prophylactic Lactobacillus GG reduces antibiotic-associated diarrhea in children with respiratory infections: a randomized study. Pediatrics 104: e64.

250. Asagi M, Ogawa T, Minetoma T, Sato K, Inaba Y (1986) Detection of transmissible gastroenteritis virus in feces from pigs by reversed passive hemagglutination. Am J Vet Res 47: 2161-2164.

251. Aseffa A (1993) Viral diseases in Ethiopia: a review. East Afr Med J 70: 624-626.

252. Ashbolt NJ (2004) Microbial contamination of drinking water and disease outcomes in developing regions. Toxicology 198: 229-238.

253. Ashdown LR, Koehler JM (1993) The spectrum of Aeromonas-associated diarrhea in tropical Queensland, Australia. Southeast Asian J Trop Med Public Health 24: 347-353.

254. Ashkenazi S (1996) Role of human milk constituents in blocking the adherence of enteric pathogens. Adv Exp Med Biol 408: 187-192.

255. Ashley CR, Caul EO, Clarke SK, Corner BD, Dunn S (1978) Rotavirus infections of apes. Lancet 2: 477.

256. Ashraf H, Ahmed S, Fuchs GJ, Mahalanabis D (2002) Persistent diarrhoea: associated infection and response to a low lactose diet. J Trop Pediatr 48: 142-148.

257. Askaa J, Bloch B (1984) Infection in piglets with a porcine rotavirus-like virus. Experimental inoculation and ultrastructural examination. Arch Virol 80: 291-303.

258. Askaa J, Bloch B, Bertelsen G, Rasmussen KO (1983) Rotavirus associated diarrhoea in nursing piglets and detection of antibody against rotavirus in colostrum, milk and serum. Nord Vet Med 35: 441-447.

259. Asmah RH, Green J, Armah GE, Gallimore CI, Gray JJ, et al. (2001) Rotavirus G and P genotypes in rural Ghana. J Clin Microbiol 39: 1981-1984.

260. Athanassious R, Marsolais G, Assaf R, Dea S, Descoteaux JP, et al. (1994) Detection of bovine coronavirus and type A rotavirus in neonatal calf diarrhea and winter dysentery of cattle in Quebec: evaluation of three diagnostic methods. Can Vet J 35: 163-169.

261. Atii DJ, Ojeh CK, Durojaiye OA (1990) Detection of rotavirus antigen in diarrhoeic and non-diarrhoeic piglets in Nigeria. Rev Elev Med Vet Pays Trop 42: 494-496.

262. Audu R, Omilabu SA, de Beer M, Peenze I, Steele AD (2002) Diversity of human rotavirus VP6, VP7, and VP4 in Lagos State, Nigeria. J Health Popul Nutr 20: 59-64.

263. Audu R, Omilabu SA, Peenze I, Steele D (2002) Viral diarrhoea in young children in two districts in Nigeria. Cent Afr J Med 48: 59-63.

264. Avendano LF, Calderon A, Macaya J, Prenzel I, Duarte E (1982) Rotavirus viral RNA electrophoresis in hospitalized infants with diarrhea in Santiago, Chile. Pediatr Res 16: 329-330.

265. Avendano LF, Calderon A, Vargas S (1981) [Rotavirus and acute diarrhea in infancy: comparison of hospitalized and outpatient cases (author's transl)]. Rev Med Chil 109: 303-305.

266. Avendano LF, Dubinovsky S, James HD, Jr. (1984) Comparison of viral RNA electrophoresis and indirect ELISA methods in the diagnosis of human rotavirus infection. Bull Pan Am Health Organ 18: 245-249.

267. Avendano LF, Duffau G, Emilfork M, Barraza P, Prenzel I, et al. (1984) [Rotavirus in diarrheas of prolonged course]. Rev Chil Pediatr 55: 94-97.

268. Avendano LF, Ojeda JM, Calderon A, Macaya J, Prenzel I, et al. (1980) [Rotavirus in infantile diarrhea (author's transl)]. Rev Med Chil 108: 210-213.

269. Avendano LF, Spencer E, Calderon A, Martinez A (1983) [Rotavirus infection in infants with acute diarrhea. Clinical and epidemiologic aspects]. Rev Med Chil 111: 240-246.

270. Avendano P, Matson DO, Long J, Whitney S, Matson CC, et al. (1993) Costs associated with office visits for diarrhea in infants and toddlers. Pediatr Infect Dis J 12: 897-902.

271. Avery RM, Shelton AP, Beards GM, Omotade OO, Oyejide OC, et al. (1992) Viral agents associated with infantile gastroenteritis in Nigeria: relative prevalence of adenovirus serotypes 40 and 41, astrovirus, and rotavirus serotypes 1 to 4. J Diarrhoeal Dis Res 10: 105-108.

272. Avram G, Zavate O, Combiescu AA, Persu A, Ivan A, et al. (1987) [Detection of the rotavirus group antigen by a screening test using the ELISA-IC kit in subjects with acute gastroenteritis, at the pediatric services of Moldavia]. Virologie 38: 169-175.

273. Awachat PS, Kelkar SD (2004) Evidence of rotavirus AU32 like G9 strains from nontypeable fecal specimens of Indian children hospitalized during 1993-1994. J Med Virol 74: 656-661.

274. Awachat PS, Kelkar SD (2005) Unexpected detection of simian SA11-human reassortant strains of rotavirus G3P[8] genotype from diarrhea epidemic among tribal children of Western India. J Med Virol 77: 128-135.

275. Awachat PS, Kelkar SD (2006) Dual infection due to simian G3--human reassortant and human G9 strains of rotavirus in a child and subsequent spread of serotype G9, leading to diarrhea among grandparents. J Med Virol 78: 134-138.

276. Awasthi S (2008) Next generation of human vaccines: what does the future hold? Hum Vaccin 4: 344-346.

277. Azevedo MS, Yuan L, Jeong KI, Gonzalez A, Nguyen TV, et al. (2005) Viremia and nasal and rectal shedding of rotavirus in gnotobiotic pigs inoculated with Wa human rotavirus. J Virol 79: 5428-5436.

278. Azevedo MS, Yuan L, Pouly S, Gonzales AM, Jeong KI, et al. (2006) Cytokine responses in gnotobiotic pigs after infection with virulent or attenuated human rotavirus. J Virol 80: 372-382.

279. Azim T, Ahmad SM, Sefat EK, Sarker MS, Unicomb LE, et al. (1999) Immune response of children who develop persistent diarrhea following rotavirus infection. Clin Diagn Lab Immunol 6: 690-695.

280. Azim T, Zaki MH, Podder G, Sultana N, Salam MA, et al. (2003) Rotavirus-specific subclass antibody and cytokine responses in Bangladeshi children with rotavirus diarrhoea. J Med Virol 69: 286-295.

281. Babiuk LA, Acres SD, Rouse BT (1977) Solid-phase radioimmunoassay for detecting bovine (neonatal calf diarrhea) rotavirus antibody. J Clin Microbiol 6?-71: 10-15.

282. Babiuk LA, Sabara M, Hudson GR (1985) Rotavirus and coronavirus infections in animals. Prog Vet Microbiol Immunol 1: 80-120.

283. Bach C, Assathiany R, Pincet J, Champsaur H (1978) [Rota virus infantile gastroenteritis: clinical and epidemiological study]. Nouv Presse Med 7: 4146-4147.

284. Bachmann PA (1979) [Rotavirus detection in the feces: experiences with the enzyme-linked immunosorbent assay (ELISA)]. Zentralbl Veterinarmed B 26: 835-842.

285. Bachmann PA (1985) [Pathogenesis and immunology of virus-induced neonatal diarrhea]. Berl Munch Tierarztl Wochenschr 98: 294-298.

286. Bachmann PA, Hess RG (1981) Routine isolation and cultivation of bovine rotaviruses in cell culture. Am J Vet Res 42: 2149-2150.

287. Bae EA, Han MJ, Lee M, Kim DH (2000) In vitro inhibitory effect of some flavonoids on rotavirus infectivity. Biol Pharm Bull 23: 1122-1124.

288. Bagci S, Eis-Hubinger AM, Franz AR, Bierbaum G, Heep A, et al. (2008) Detection of astrovirus in premature infants with necrotizing enterocolitis. Pediatr Infect Dis J 27: 347-350.

289. Baggi F, Peduzzi R (2000) Genotyping of rotaviruses in environmental water and stool samples in Southern Switzerland by nucleotide sequence analysis of 189 base pairs at the 5' end of the VP7 gene. J Clin Microbiol 38: 3681-3685.

290. Bahl R, Ray P, Subodh S, Shambharkar P, Saxena M, et al. (2005) Incidence of severe rotavirus diarrhea in New Delhi, India, and G and P types of the infecting rotavirus strains. J Infect Dis 192 Suppl 1: S114-119.

291. Bajolet O, Chippaux-Hyppolite C (1998) [Rotavirus and other viruses of diarrhea]. Bull Soc Pathol Exot 91: 432-437.

292. Balamurugan R, Janardhan HP, George S, Raghava MV, Muliyil J, et al. (2008) Molecular studies of fecal anaerobic commensal bacteria in acute diarrhea in children. J Pediatr Gastroenterol Nutr 46: 514-519.

293. Baldacci ER, Candeias JA, Breviglieri JC, Grisi SJ (1979) [Viral and bacterial etiology of infantile gastroenteritis cases: clinical characterization]. Rev Saude Publica 13: 47-53.

294. Baljer G, Bachmann PA (1980) [Demonstration of enteropathogenic Escherichia coli strains and rotaviruses in fecal samples from calves with diarrhea]. Zentralbl Veterinarmed B 27: 608-615.

295. Ball JM, Tian P, Zeng CQ, Morris AP, Estes MK (1996) Age-dependent diarrhea induced by a rotaviral nonstructural glycoprotein. Science 272: 101-104.

296. Ballal M, Jyothirlatha, Kotigadde S, Venkatesh A, Shivananda PG (1992) Rotavirus and bacterial enteropathogens causing acute diarrhea. Indian J Pediatr 59: 203-207.

297. Ballal M, Shivananda PG (2002) Rotavirus and enteric pathogens in infantile diarrhoea in Manipal, South India. Indian J Pediatr 69: 393-396.

298. Ballotti S, de Martino M (2007) Rotavirus infections and development of type 1 diabetes: an evasive conundrum. J Pediatr Gastroenterol Nutr 45: 147-156.

299. Balter S, Weiss D, Hanson H, Reddy V, Das D, et al. (2005) Three years of emergency department gastrointestinal syndromic surveillance in New York City: what have we found? MMWR Morb Mortal Wkly Rep 54 Suppl: 175-180.

300. Banatvala JE (1979) The role of viruses in acute diarrhoeal disease. Clin Gastroenterol 8: 569-598.

301. Banatvala JE (1979) Viruses and diarrhoea. Trans R Soc Trop Med Hyg 73: 503-508.

302. Banatvala JE, Chrystie IL (1978) Rotaviral infections in human neonates. J Am Vet Med Assoc 173: 527-530.

303. Banerjee I, Gladstone BP, Le Fevre AM, Ramani S, Iturriza-Gomara M, et al. (2007) Neonatal infection with G10P[11] rotavirus did not confer protection against subsequent rotavirus infection in a community cohort in Vellore, South India. J Infect Dis 195: 625-632.

304. Banerjee I, Iturriza-Gomara M, Rajendran P, Primrose B, Ramani S, et al. (2007) Molecular characterization of G11P[25] and G3P[3] human rotavirus strains associated with asymptomatic infection in South India. J Med Virol 79: 1768-1774.

305. Banerjee I, Primrose Gladstone B, Iturriza-Gomara M, Gray JJ, Brown DW, et al. (2008) Evidence of intrafamilial transmission of rotavirus in a birth cohort in South India. J Med Virol 80: 1858-1863.

306. Banerjee I, Ramani S, Primrose B, Iturriza-Gomara M, Gray JJ, et al. (2007) Modification of rotavirus multiplex RT-PCR for the detection of G12 strains based on characterization of emerging G12 rotavirus strains from South India. J Med Virol 79: 1413-1421.

307. Banerjee I, Ramani S, Primrose B, Moses P, Iturriza-Gomara M, et al. (2006) Comparative study of the epidemiology of rotavirus in children from a community-based birth cohort and a hospital in South India. J Clin Microbiol 44: 2468-2474.

308. Banyai K, Angyal M, Kormendi E, Lakatos F, Uj M, et al. (2002) [Outbreak of human rotavirus infection in an adult community]. Orv Hetil 143: 1347-1352.

309. Banyai K, Bogdan A, Kisfali P, Molnar P, Mihaly I, et al. (2007) Emergence of serotype G12 rotaviruses, Hungary. Emerg Infect Dis 13: 916-919.

310. Banyai K, Deak J, Gray J, Iturriza-Gomara M, Kovacs J, et al. (2007) [EuroRotaNet--European rotavirus strain surveillance network established with Hungarian participation]. Orv Hetil 148: 2043-2045.

311. Banyai K, Forgach P, Erdelyi K, Martella V, Bogdan A, et al. (2005) Identification of the novel lapine rotavirus genotype P[22] from an outbreak of enteritis in a Hungarian rabbitry. Virus Res 113: 73-80.

312. Banyai K, Gentsch JR, Griffin DD, Holmes JL, Glass RI, et al. (2003) Genetic variability among serotype G6 human rotaviruses: identification of a novel lineage isolated in Hungary. J Med Virol 71: 124-134.

313. Banyai K, Gentsch JR, Schipp R, Jakab F, Meleg E, et al. (2005) Dominating prevalence of P[8],G1 and P[8],G9 rotavirus strains among children admitted to hospital between 2000 and 2003 in Budapest, Hungary. J Med Virol 76: 414-423.

314. Banyai K, Jiang B, Bogdan A, Horvath B, Jakab F, et al. (2006) Prevalence and molecular characterization of human group C rotaviruses in Hungary. J Clin Virol 37: 317-322.

315. Banyai K, Sas Y, Varga L, Szucs G (2004) Survey of rotavirus infection in a Hungarian paediatric hospital. A short communication. Acta Microbiol Immunol Hung 51: 431-435.

316. Baqai R (1983) Rotavirus gastroenteritis. J Pak Med Assoc 33: 240-242.

317. Baqai R (1986) Diarrhoeal diseases. J Pak Med Assoc 36: 1-4.

318. Baqai R (2000) Rapid diagnosis of rotavirus in infantile diarrhoea. J Pak Med Assoc 50: 243-244.

319. Baqai R, Zuberi SJ, Khan MA (1985) Significance of E. coli and rotavirus in infantile diarrhoea. J Pak Med Assoc 35: 326-328.

320. Baqui AH, Sack RB, Black RE, Haider K, Hossain A, et al. (1992) Enteropathogens associated with acute and persistent diarrhea in Bangladeshi children less than 5 years of age. J Infect Dis 166: 792-796.

321. Barboi G, Carstet I, Cristescu P, Pirvulescu M, Staicu L, et al. (1987) Isolation of a cytopathic bovine rotavirus strain in the Socialist Republic of Romania. Arch Exp Veterinarmed 41: 926-929.

322. Bardhan PK, Beltinger J, Beltinger RW, Hossain A, Mahalanabis D, et al. (2000) Screening of patients with acute infectious diarrhoea: evaluation of clinical features, faecal microscopy, and faecal occult blood testing. Scand J Gastroenterol 35: 54-60.

323. Bardhan PK, Salam MA, Molla AM (1992) Gastric emptying of liquid in children suffering from acute rotaviral gastroenteritis. Gut 33: 26-29.

324. Barker J (2001) The role of viruses in gastrointestinal disease in the home. J Infect 43: 42-44.

325. Barker RA, Maxwell PH, Hong CP, Cordery MC, Chrystie IL (1988) Paediatric gastroenteritis in the eastern Malaysian state of Sarawak: an epidemiological and clinical study. Trans R Soc Trop Med Hyg 82: 898-901.

326. Barlow RS, Hirst RG, Norton RE, Ashhurst-Smith C, Bettelheim KA (1999) A novel serotype of enteropathogenic Escherichia coli (EPEC) as a major pathogen in an outbreak of infantile diarrhoea. J Med Microbiol 48: 1123-1125.

327. Barman P, Ghosh S, Das S, Varghese V, Chaudhuri S, et al. (2004) Sequencing and sequence analysis of VP7 and NSP5 genes reveal emergence of a new genotype of bovine group B rotaviruses in India. J Clin Microbiol 42: 2816-2818.

328. Barman P, Ghosh S, Samajdar S, Mitra U, Dutta P, et al. (2006) RT-PCR based diagnosis revealed importance of human group B rotavirus infection in childhood diarrhoea. J Clin Virol 36: 222-227.

329. Barnes G (1998) Rotavirus vaccines. Zhonghua Min Guo Xiao Er Ke Yi Xue Hui Za Zhi 39: 17-20.

330. Barnes GL (1989) Rotavirus vaccines: science and politics. Aust Paediatr J 25: 338-339.

331. Barnes GL, Bishop RF (1997) Rotavirus infection and prevention. Curr Opin Pediatr 9: 19-23.

332. Barnes GL, Bishop RF (2006) Rotavirus vaccine--time to act. Med J Aust 185: 352-353.

333. Barnes GL, Callaghan SL, Kirkwood CD, Bogdanovic-Sakran N, Johnston LJ, et al. (2003) Excretion of serotype G1 rotavirus strains by asymptomatic staff: a possible source of nosocomial infection. J Pediatr 142: 722-725.

334. Barnes GL, Doyle LW, Hewson PH, Knoches AM, McLellan JA, et al. (1982) A randomised trial of oral gammaglobulin in low-birth-weight infants infected with rotavirus. Lancet 1: 1371-1373.

335. Barnes GL, Unicomb L, Bishop RF (1992) Severity of rotavirus infection in relation to serotype, monotype and electropherotype. J Paediatr Child Health 28: 54-57.

336. Barnes GL, Uren E, Stevens KB, Bishop RF (1998) Etiology of acute gastroenteritis in hospitalized children in Melbourne, Australia, from April 1980 to March 1993. J Clin Microbiol 36: 133-138.

337. Barnett B (1983) Viral gastroenteritis. Med Clin North Am 67: 1031-1058.

338. Barnett BB (1986) Other viruses with etiologic roles in childhood gastroenteritis. Pediatr Infect Dis 5: S75-82.

339. Barnett BB, Egbert LN, Spendlove RS (1978) Characteristics of neonatal calf diarrhea virus ribonucleic acid. Can J Comp Med 42: 46-53.

340. Barone C, Pettinato R, Avola E, Alberti A, Greco D, et al. (2000) Comparison of three probiotics in the treatment of acute diarrhea in mentally retarded children. Minerva Pediatr 52: 161-165.

341. Barrandeguy M, Parreno V, Lagos Marmol M, Pont Lezica F, Rivas C, et al. (1998) Prevention of rotavirus diarrhoea in foals by parenteral vaccination of the mares: field trial. Dev Biol Stand 92: 253-257.

342. Barraza P, Avendano LF, Spencer E, Calderon A, Prenzel I, et al. (1986) [Hospital infection caused by rotaviruses in infants, Santiago, Chile]. Bol Oficina Sanit Panam 101: 328-338.

343. Barreiros MA, Alfieri AA, Alfieri AF, Medici KC, Leite JP (2003) An outbreak of diarrhoea in one-week-old piglets caused by group A rotavirus genotypes P[7],G3 and P[7],G5. Vet Res Commun 27: 505-512.

344. Barreiros MA, Alfieri AF, Medici KC, Leite JP, Alfieri AA (2004) G and P genotypes of group A rotavirus from diarrhoeic calves born to cows vaccinated against the NCDV (P[1],G6) rotavirus strain. J Vet Med B Infect Dis Vet Public Health 51: 104-109.

345. Barrera F, Rebollo MJ, Espinoza J, Araya M, Brunser O, et al. (1989) [Modular diet and parenteral support in persistent diarrhea]. Rev Chil Pediatr 60: 150-157.

346. Barril PA, Martinez LC, Giordano MO, Castello AA, Rota RP, et al. (2006) Detection of group a human rotavirus G9 genotype circulating in Cordoba, Argentina, as early as 1980. J Med Virol 78: 1113-1118.

347. Barrios H, Viora S, de Franceschi M, Fliess E (1991) [Incidence of rotavirus in intensive-production poultry farms]. Rev Argent Microbiol 23: 15-21.

348. Barron-Romero BL, Barreda-Gonzalez J, Doval-Ugalde R, Zermeno-Eguia Liz J, Huerta-Pena M (1985) Asymptomatic rotavirus infections in day care centers. J Clin Microbiol 22: 116-118.

349. Barrow PA, Brooker BE, Fuller R, Newport MJ, Sojka WJ, et al. (1979) The aetiology of diarrhoea in pigs weaned at two days of age. Res Vet Sci 27: 52-58.

350. Bart KJ, Lin KF (1990) Vaccine-preventable disease and immunization in the developing world. Pediatr Clin North Am 37: 735-756.

351. Bartlett AV, 3rd, Bednarz-Prashad AJ, DuPont HL, Pickering LK (1987) Rotavirus gastroenteritis. Annu Rev Med 38: 399-415.

352. Bartlett AV, Moore M, Gary GW, Starko KM, Erben JJ, et al. (1985) Diarrheal illness among infants and toddlers in day care centers. I. Epidemiology and pathogens. J Pediatr 107: 495-502.

353. Bartlett AV, Moore M, Gary GW, Starko KM, Erben JJ, et al. (1985) Diarrheal illness among infants and toddlers in day care centers. II. Comparison with day care homes and households. J Pediatr 107: 503-509.

354. Bartlett AV, 3rd, Reves RR, Pickering LK (1988) Rotavirus in infant-toddler day care centers: epidemiology relevant to disease control strategies. J Pediatr 113: 435-441.

355. Bartolozzi G, Franceschini E, Cividalli S, Braito A, Dei R, et al. (1982) [Role of viruses in acute gastroenteritis]. Pediatr Med Chir 4: 269-274.

356. Bartz CR, Conklin RH, Tunstall CB, Steele JH (1980) Prevention of murine rotavirus infection with chicken egg yolk immunoglobulins. J Infect Dis 142: 439-441.

357. Basnec SN, Giordano MO, Bennun FR, Nates SV, Vergara M, et al. (1991) Detection of two atypical rotaviruses in the province of Misiones, Argentina. Acta Virol 35: 408-412.

358. Bass D (1994) Can we actively treat rotavirus gastroenteritis? J Pediatr Gastroenterol Nutr 19: 473-474.

359. Bass D, Cordoba E, Dekker C, Schuind A, Cassady C (2004) Intestinal imaging of children with acute rotavirus gastroenteritis. J Pediatr Gastroenterol Nutr 39: 270-274.

360. Bass DM (1997) Interferon gamma and interleukin 1, but not interferon alfa, inhibit rotavirus entry into human intestinal cell lines. Gastroenterology 113: 81-89.

361. Bass DM (2000) Rotavirus vaccinology: good news and bad news. J Pediatr Gastroenterol Nutr 30: 10-11.

362. Bass DM, Baylor M, Broome R, Greenberg HB (1992) Molecular basis of age-dependent gastric inactivation of rhesus rotavirus in the mouse. J Clin Invest 89: 1741-1745.

363. Bass DM, Baylor MR, Chen C, Mackow EM, Bremont M, et al. (1992) Liposome-mediated transfection of intact viral particles reveals that plasma membrane penetration determines permissivity of tissue culture cells to rotavirus. J Clin Invest 90: 2313-2320.

364. Bass ES, Pappano DA, Humiston SG (2007) Rotavirus. Pediatr Rev 28: 183-191.

365. Basu G, Rossouw J, Sebunya TK, Gashe BA, de Beer M, et al. (2003) Prevalence of rotavirus, adenovirus and astrovirus infection in young children with gastroenteritis in Gaborone, Botswana. East Afr Med J 80: 652-655.

366. Basu S, Chatterjee M, Ganguly S, Chandra PK (2007) Efficacy of Lactobacillus rhamnosus GG in acute watery diarrhoea of Indian children: a randomised controlled trial. J Paediatr Child Health 43: 837-842.

367. Basu S, Paul DK, Ganguly S, Chatterjee M, Chandra PK (2008) Efficacy of High-dose Lactobacillus rhamnosus GG in Controlling Acute Watery Diarrhea in Indian Children: A Randomized Controlled Trial. J Clin Gastroenterol.

368. Bates PR, Bailey AS, Wood DJ, Morris DJ, Couriel JM (1993) Comparative epidemiology of rotavirus, subgenus F (types 40 and 41) adenovirus and astrovirus gastroenteritis in children. J Med Virol 39: 224-228.

369. Battikhi MN (2002) Epidemiological study on Jordanian patients suffering from diarrhoea. New Microbiol 25: 405-412.

370. Baumeister BM, Castro AE, McGuire-Rodgers SJ, Ramsay EC (1983) Detection and control of rotavirus infections in zoo animals. J Am Vet Med Assoc 183: 1252-1254.

371. Baumgarte S, de Souza Luna LK, Grywna K, Panning M, Drexler JF, et al. (2008) Prevalence, types, and RNA concentrations of human parechoviruses, including a sixth parechovirus type, in stool samples from patients with acute enteritis. J Clin Microbiol 46: 242-248.

372. Baumgarte S, de Souza Luna LK, Grywna K, Panning M, Drexler JF, et al. (2008) Prevalence, types, and RNA concentrations of human parechoviruses, including a sixth parechovirus type, in stool samples from patients with acute enteritis. J Clin Microbiol 46: 242-248.

373. Bazsika A, Szarka E, Pinter S, Mikola I (1985) [Familial cumulation of the incidence of gastroenteritis caused by Rotavirus]. Orv Hetil 126: 1653-1655.

374. Beards G, Graham C (1995) Temporal distribution of rotavirus G-serotypes in the West Midlands region of the United Kingdom, 1983-1994. J Diarrhoeal Dis Res 13: 235-237.

375. Beards GM, Brown DW (1988) The antigenic diversity of rotaviruses: significance to epidemiology and vaccine strategies. Eur J Epidemiol 4: 1-11.

376. Beards GM, Bryden AS (1981) Evaluation of a new enzyme-linked immunosorbent assay test for rotavirus antigen in faeces. J Clin Pathol 34: 1388-1391.

377. Beards GM, Campbell AD, Cottrell NR, Peiris JS, Rees N, et al. (1984) Enzyme-linked immunosorbent assays based on polyclonal and monoclonal antibodies for rotavirus detection. J Clin Microbiol 19: 248-254.

378. Beards GM, Desselberger U, Flewett TH (1989) Temporal and geographical distributions of human rotavirus serotypes, 1983 to 1988. J Clin Microbiol 27: 2827-2833.

379. Beards GM, Hall C, Green J, Flewett TH, Lamouliatte F, et al. (1984) An enveloped virus in stools of children and adults with gastroenteritis that resembles the Breda virus of calves. Lancet 1: 1050-1052.

380. Beards GM, Pilfold JN, Thouless ME, Flewett TH (1980) Rotavirus serotypes by serum neutralisation. J Med Virol 5: 231-237.

381. Beattie RM, Vieira MC, Phillips AD, Meadows N, Walker-Smith JA (1995) Carbohydrate intolerance after rotavirus gastroenteritis: a rare problem in the 1990s. Arch Dis Child 72: 466.

382. Beau I, Berger A, Servin AL (2007) Rotavirus impairs the biosynthesis of brush-border-associated dipeptidyl peptidase IV in human enterocyte-like Caco-2/TC7 cells. Cell Microbiol 9: 779-789.

383. Bednarz-Prashad AJ, John EI (1983) Effect of clioquinol, an 8-hydroxyquinoline derivative, on rotavirus infection in mice. J Infect Dis 148: 613.

384. Begue RE, Neill MA, Papa EF, Dennehy PH (1994) A prospective study of Shiga-like toxin-associated diarrhea in a pediatric population. J Pediatr Gastroenterol Nutr 19: 164-169.

385. Behymer DE, Riemann HP, Utterback W, C DE, Franti CE (1991) Mass screening of cattle sera against 14 infectious disease agents, using an ELISA system for monitoring health in livestock. Am J Vet Res 52: 1699-1705.

386. Bektimirov TA (1990) [Rotavirus vaccines]. Zh Mikrobiol Epidemiol Immunobiol: 94-98.

387. Belaia Iu A, Shekoian LA, Drozdov SG, Prozorovskii SV, Belaia OF (1985) [Diagnosis of rotavirus infection using coagglutination reactions]. Vopr Virusol 30: 233-236.

388. Belchev L, Arnaudov D, Ignatov G, Boiadzhiev S (1987) [Mixed coli-rotavirus and Cryptosporidium infection in newborn piglets]. Vet Med Nauki 24: 3-8.

389. Belhorn T (1999) Rotavirus diarrhea. Curr Probl Pediatr 29: 198-207.

390. Bell LM, Clark HF, O'Brien EA, Kornstein MJ, Plotkin SA, et al. (1987) Gastroenteritis caused by human rotaviruses (serotype three) in a suckling mouse model. Proc Soc Exp Biol Med 184: 127-132.

391. Bellaiche M, Viala J, Degas V, Cezard JP (2007) [Rotavirus: an ubiquitous infection?]. Arch Pediatr 14 Suppl 3: S156-158.

392. Bellido-Blasco JB, Celades-Porcar ME, Tirado-Balaguer MD, Gonzalez-Cano JM, Gil-Ortuno M, et al. (2006) [Infectious diarrhea study in Castellon, Spain (EDICS): population incidence of sporadic cases in 2004 and comparison with the year 2000]. Med Clin (Barc) 127: 448-450.

393. Bellido-Blasco JB, Gonzalez-Cano JM, Galiano-Arlandis JV, Herrero-Carot C, Tirado-Balaguer MD, et al. (2007) [Risk factors for the occurrence of sporadic Campylobacter, Salmonella and rotavirus diarrhea in preschool children]. An Pediatr (Barc) 66: 367-374.

394. Bellinzoni R, Mattion N, Vallejos L, La Torre JL, Scodeller EA (1987) Atypical rotavirus in chickens in Argentina. Res Vet Sci 43: 130-131.

395. Bellinzoni RC, Blackhall J, Baro N, Auza N, Mattion N, et al. (1989) Efficacy of an inactivated oil-adjuvanted rotavirus vaccine in the control of calf diarrhoea in beef herds in Argentina. Vaccine 7: 263-268.

396. Bellinzoni RC, Blackhall J, Terzolo HR, Moreira AR, Auza N, et al. (1990) Microbiology of diarrhoea in young beef and dairy calves in Argentina. Rev Argent Microbiol 22: 130-136.

397. Bellinzoni RC, Mattion N, La Torre JL, Scodeller EA (1987) Incidence of rotavirus in beef herds in Argentina. Res Vet Sci 42: 257-259.

398. Bellinzoni RC, Mattion NM, Burrone O, Gonzalez A, La Torre JL, et al. (1987) Isolation of group A swine rotaviruses displaying atypical electropherotypes. J Clin Microbiol 25: 952-954.

399. Ben-Ami T, Sinai L, Granot E (2007) Afebrile seizures and rotavirus gastroenteritis: an infrequently recognized association. Clin Pediatr (Phila) 46: 178-180.

400. Benbachir M, El Mdaghri N, Bennani A, Tazi-Lakhsassi L (1984) [Etiological evaluation of acute diarrhea in children hospitalized in Casablanca]. Pathol Biol (Paris) 32: 969-971.

401. Bendali F, Bichet H, Schelcher F, Sanaa M (1999) Pattern of diarrhoea in newborn beef calves in south-west France. Vet Res 30: 61-74.

402. Bendall RP, Gray JJ (1991) Haemorrhagic colitis and haemolytic-uraemic syndrome: false positive reaction with a rotavirus latex agglutination test. J Clin Pathol 44: 609-610.

403. Benenson AS (1991) [Development of a rehydration therapy in diarrheic disease. 1980]. Rev Med Panama 16: 161-172.

404. Benfield DA, Francis DH, McAdaragh JP, Johnson DD, Bergeland ME, et al. (1988) Combined rotavirus and K99 Escherichia coli infection in gnotobiotic pigs. Am J Vet Res 49: 330-337.

405. Benfield DA, Jackwood DJ, Bac I, Saif LJ, Wesley RD (1991) Detection of transmissible gastroenteritis virus using cDNA probes. Arch Virol 116: 91-106.

406. Benfield DA, Stotz I, Moore R, McAdaragh JP (1982) Shedding of rotavirus in feces of sows before and after farrowing. J Clin Microbiol 16: 186-190.

407. Benfield DA, Stotz IJ, Nelson EA, Groon KS (1984) Comparison of a commercial enzyme-linked immunosorbent assay with electron microscopy, fluorescent antibody, and virus isolation for the detection of bovine and porcine rotavirus. Am J Vet Res 45: 1998-2002.

408. Bennet R, Hedlund KO, Ehrnst A, Eriksson M (1995) Nosocomial gastroenteritis in two infant wards over 26 months. Acta Paediatr 84: 667-671.

409. Bentele KH, Albani M (1988) [Acute apparently life threatening events in 62 infants: anamnestic and clinical data]. Klin Padiatr 200: 57-63.

410. Benureau Y, Huet JC, Charpilienne A, Poncet D, Cohen J (2005) Trypsin is associated with the rotavirus capsid and is activated by solubilization of outer capsid proteins. J Gen Virol 86: 3143-3151.

411. Bereciartu A, Bok K, Gomez J (2002) Identification of viral agents causing gastroenteritis among children in Buenos Aires, Argentina. J Clin Virol 25: 197-203.

412. Berger R, Hadziselimovic F, Just M, Reigel F (1984) Influence of breast milk on nosocomial rotavirus infections in infants. Infection 12: 171-174.

413. Berger R, Hadziselimovic F, Just M, Reigel P (1983) Effect of feeding human milk on nosocomial rotavirus infections in an infants ward. Dev Biol Stand 53: 219-228.

414. Berglezova LN, Solodovnikov Iu P, Temkina AA, Pozdeeva LI, Sarzhina LA, et al. (1999) [An outbreak of acute intestinal infections of unknown etiology in a preschool institution (2: answers to questions and the authors' commentary)]. Zh Mikrobiol Epidemiol Immunobiol: 114-115.

415. Bergman A, Young C, Miadi-Fargier H, Gothefors L (2008) [Health care and society have to pay a high price for rotavirus infections in children. A Swedish descriptive cost analysis study]. Lakartidningen 105: 1186-1191.

416. Berkova Z, Crawford SE, Blutt SE, Morris AP, Estes MK (2007) Expression of rotavirus NSP4 alters the actin network organization through the actin remodeling protein cofilin. J Virol 81: 3545-3553.

417. Berkova Z, Crawford SE, Trugnan G, Yoshimori T, Morris AP, et al. (2006) Rotavirus NSP4 induces a novel vesicular compartment regulated by calcium and associated with viroplasms. J Virol 80: 6061-6071.

418. Berkowitz FE, Schoub BD, Cohen F, Thompson D (1983) Rotavirus infection in Black and White newborns. Isr J Med Sci 19: 892-893.

419. Bern C, Unicomb L, Gentsch JR, Banul N, Yunus M, et al. (1992) Rotavirus diarrhea in Bangladeshi children: correlation of disease severity with serotypes. J Clin Microbiol 30: 3234-3238.

420. Bernard S, Jestin A (1985) Rotavirus infections in conventional pigs: kinetics excretion in faeces of rotavirus antigens, antibodies and immune complexes by pigs from birth up to three month of age. Zentralbl Veterinarmed B 32: 306-315.

421. Bernard S, Lantier I, Laude H, Aynaud JM (1986) Detection of transmissible gastroenteritis coronavirus antigens by a sandwich enzyme-linked immunosorbent assay technique. Am J Vet Res 47: 2441-2444.

422. Berner R, Schumacher RF, Forster J (1997) Survey on rotavirus infections in a German pediatric hospital. Eur J Clin Microbiol Infect Dis 16: 479-481.

423. Berner R, Schumacher RF, Hameister S, Forster J (1999) Occurrence and impact of community-acquired and nosocomial rotavirus infections--a hospital-based study over 10 y. Acta Paediatr Suppl 88: 48-52.

424. Bernstein DI (2000) Rotavirus vaccine: current status and future prospects. BioDrugs 14: 275-281.

425. Bernstein DI (2006) Live attenuated human rotavirus vaccine, Rotarix. Semin Pediatr Infect Dis 17: 188-194.

426. Bernstein DI (2007) A live attenuated human rotavirus vaccine. Drugs Today (Barc) 43: 281-291.

427. Bernstein DI, Glass RI, Rodgers G, Davidson BL, Sack DA (1995) Evaluation of rhesus rotavirus monovalent and tetravalent reassortant vaccines in US children. US Rotavirus Vaccine Efficacy Group. Jama 273: 1191-1196.

428. Bernstein DI, Kacica MA, McNeal MM, Schiff GM, Ward RL (1989) Local and systemic antibody response to rotavirus WC3 vaccine in adult volunteers. Antiviral Res 12: 293-300.

429. Bernstein DI, Sack DA, Reisinger K, Rothstein E, Ward RL (2002) Second-year follow-up evaluation of live, attenuated human rotavirus vaccine 89-12 in healthy infants. J Infect Dis 186: 1487-1489.

430. Bernstein DI, Sack DA, Rothstein E, Reisinger K, Smith VE, et al. (1999) Efficacy of live, attenuated, human rotavirus vaccine 89-12 in infants: a randomised placebo-controlled trial. Lancet 354: 287-290.

431. Bernstein DI, Sander DS, Smith VE, Schiff GM, Ward RL (1991) Protection from rotavirus reinfection: 2-year prospective study. J Infect Dis 164: 277-283.

432. Bernstein DI, Smith VE, Sander DS, Pax KA, Schiff GM, et al. (1990) Evaluation of WC3 rotavirus vaccine and correlates of protection in healthy infants. J Infect Dis 162: 1055-1062.

433. Bernstein DI, Smith VE, Sherwood JR, Schiff GM, Sander DS, et al. (1998) Safety and immunogenicity of live, attenuated human rotavirus vaccine 89-12. Vaccine 16: 381-387.

434. Bernstein DI, Ziegler JM, Ward RL (1986) Rotavirus fecal IgA antibody response in adults challenged with human rotavirus. J Med Virol 20: 297-304.

435. Bernstein JM, Hruska JF (1981) Characterization of RNA polymerase products of Nebraska calf diarrhea virus and SA11 rotavirus. J Virol 37: 1071-1074.

436. Berois M, Libersou S, Russi J, Arbiza J, Cohen J (2003) Genetic variation in the VP7 gene of human rotavirus isolated in Montevideo-Uruguay from 1996-1999. J Med Virol 71: 456-462.

437. Besser TE, Gay CC, McGuire TC, Evermann JF (1988) Passive immunity to bovine rotavirus infection associated with transfer of serum antibody into the intestinal lumen. J Virol 62: 2238-2242.

438. Bethell DR, Huang J (2004) Recombinant human lactoferrin treatment for global health issues: iron deficiency and acute diarrhea. Biometals 17: 337-342.

439. Beyer J, Lange E, Fichtner D, Leopoldt D (1983) [Comparative studies using the immunofluorescence technic in experimental transmissible gastroenteritis and rotavirus infection of piglets]. Arch Exp Veterinarmed 37: 151-158.

440. Bhan MK, Arora NK, Kumar A, Mohapatra LN, Deb M, et al. (1985) Enteropathogens colonisation of the jejunum in paediatric diarrhoea. Indian J Med Res 81: 133-139.

441. Bhan MK, Bhandari N, Sazawal S, Clemens J, Raj P, et al. (1989) Descriptive epidemiology of persistent diarrhoea among young children in rural northern India. Bull World Health Organ 67: 281-288.

442. Bhan MK, Kumar R, Khoshoo V, Arora NK, Raj P, et al. (1987) Etiologic role of enterotoxigenic Escherichia coli & rotavirus in acute diarrhoea in Delhi children. Indian J Med Res 85: 604-607.

443. Bhan MK, Lew JF, Sazawal S, Das BK, Gentsch JR, et al. (1993) Protection conferred by neonatal rotavirus infection against subsequent rotavirus diarrhea. J Infect Dis 168: 282-287.

444. Bhan MK, Raj P, Bhandari N, Svensson L, Stintzing G, et al. (1988) Role of enteric adenoviruses and rotaviruses in mild and severe acute enteritis. Pediatr Infect Dis J 7: 320-323.

445. Bhan MK, Sazawal S, Bhatnagar S, Bhandari N, Guha DK, et al. (1990) Glycine, glycyl-glycine and maltodextrin based oral rehydration solution. Assessment of efficacy and safety in comparison to standard ORS. Acta Paediatr Scand 79: 518-526.

446. Bhan MK, Sazawal S, Raj P, Bhandari N, Kumar R, et al. (1989) Aggregative Escherichia coli, Salmonella, and Shigella are associated with increasing duration of diarrhea. Indian J Pediatr 56: 81-86.

447. Bhandari N, Sharma P, Glass RI, Ray P, Greenberg H, et al. (2006) Safety and immunogenicity of two live attenuated human rotavirus vaccine candidates, 116E and I321, in infants: results of a randomised controlled trial. Vaccine 24: 5817-5823.

448. Bhardwaj A, Aggarwal V, Chakravarty A, Mittal SK (1996) Does Rota virus infection cause persistent diarrhoea in childhood? Trop Gastroenterol 17: 18-21.

449. Bhat P, Macaden R, Unnykrishnan P, Rao HG (1985) Rotavirus & bacterial enteropathogens in acute diarrhoeas of young children in Bangalore. Indian J Med Res 82: 105-109.

450. Bhattacharya R, Sahoo GC, Nayak MK, Ghosh S, Dutta P, et al. (2006) Molecular epidemiology of human astrovirus infections in Kolkata, India. Infect Genet Evol 6: 425-435.

451. Bhattacharya R, Sahoo GC, Nayak MK, Rajendran K, Dutta P, et al. (2007) Detection of Genogroup I and II human picobirnaviruses showing small genomic RNA profile causing acute watery diarrhoea among children in Kolkata, India. Infect Genet Evol 7: 229-238.

452. Bhattacharya SK (2003) Progress in the prevention and control of diarrhoeal diseases since Independence. Natl Med J India 16 Suppl 2: 15-19.

453. Biermann U, Herbst W, Krauss H, Schliesser T (1989) [Electron microscopic detection rate of enteral viruses in diarrhea of dogs, cats, calves, swine and foals in the year 1988--electron microscopic study results]. Berl Munch Tierarztl Wochenschr 102: 412-414.

454. Biermann U, Schmitt K, Krauss H (1991) [Electron microscopic virus diagnosis in dogs, cats, calves, swine and foals in the year 1989]. Berl Munch Tierarztl Wochenschr 104: 117-119.

455. Bilenko N, Levy A, Dagan R, Deckelbaum RJ, El-On Y, et al. (2004) Does co-infection with Giardia lamblia modulate the clinical characteristics of enteric infections in young children? Eur J Epidemiol 19: 877-883.

456. Billal DS, Hotomi M, Yamanaka N (2008) Rotavirus vaccine for developing countries. Lancet 372: 444; author reply 445.

457. Bines JE (2005) Rotavirus vaccines and intussusception risk. Curr Opin Gastroenterol 21: 20-25.

458. Bines JE, Ivanoff B, Justice F, Mulholland K (2004) Clinical case definition for the diagnosis of acute intussusception. J Pediatr Gastroenterol Nutr 39: 511-518.

459. Bingnan F, Unicomb LE, Tu GL, Ali A, Malek A, et al. (1991) Cultivation and characterization of novel human group A rotaviruses with long RNA electropherotypes, subgroup II specificities, and serotype 2 VP7 genes. J Clin Microbiol 29: 2224-2227.

460. Bini JC, Ekaza E, Faye-Kette H, Veh KA, Nigue L, et al. (2007) [Detection by RT-PCR of the 1st cases of Astrovirus in human stools in Abidjan, Cote d'Ivoire]. Bull Soc Pathol Exot 100: 243-245.

461. Binka FN, Anto FK, Oduro AR, Awini EA, Nazzar AK, et al. (2003) Incidence and risk factors of paediatric rotavirus diarrhoea in northern Ghana. Trop Med Int Health 8: 840-846.

462. Birch CJ, Heath RL, Gust ID (1988) Use of serotype-specific monoclonal antibodies to study the epidemiology of rotavirus infection. J Med Virol 24: 45-53.

463. Birch CJ, Lehmann NI, Hawker AJ, Marshall JA, Gust ID (1979) Comparison of electron microscopy, enzyme-linked immunosorbent assay, solid-phase radioimmunoassay, and indirect immunofluorescence for detection of human rotavirus antigen in faeces. J Clin Pathol 32: 700-705.

464. Birch CJ, Lewis FA, Kennett ML, Homola M, Pritchard H, et al. (1977) A study of the prevalence of rotavirus infection in children with gastroenteritis admitted to an infectious diseases hospital. J Med Virol 1: 69-77.

465. Biritwum RB, Asante A, Amoo PK, Gyekye AA, Amissah CR, et al. (2004) Community-based cluster surveys on treatment preferences for diarrhoea, severe diarrhoea, and dysentery in children aged less than five years in two districts of Ghana. J Health Popul Nutr 22: 182-190.

466. Biritwum RB, Isomura S, Yamaguchi H, Toba M, Mingle JA (1984) Seroepidemiological study of rotavirus infection in rural Ghana. Ann Trop Paediatr 4: 237-240.

467. Bishai FR, Blaskovic P, Goodwin D (1978) Physicochemical properties of Nebraska calf diarrhea virus hemagglutinin. Can J Microbiol 24: 1425-1430.

468. Bishai FR, Spence L, Goodwin D, Petro R (1979) Use of antisera against bovine (NCDV) and simian (SA11) rotaviruses in ELISA to detect different types of human rotavirus. Can J Microbiol 25: 1118-1124.

469. Bishop R (1988) The present status of rotavirus vaccine development. Southeast Asian J Trop Med Public Health 19: 429-435.

470. Bishop R (1999) Ruth Bishop: rotaviruses and vaccines. Interview by Amanda Tattam. Lancet 353: 1860.

471. Bishop RF (1984) Rotavirus in perspective--a personal view. Aust Paediatr J 20: 9-12.

472. Bishop RF (1993) Development of candidate rotavirus vaccines. Vaccine 11: 247-254.

473. Bishop RF (1996) Natural history of human rotavirus infection. Arch Virol Suppl 12: 119-128.

474. Bishop RF, Barnes GL (1997) Neonatal rotavirus infection: possible effect on prevalence of severe diarrhoea in a community. J Paediatr Child Health 33: 80.

475. Bishop RF, Barnes GL, Cipriani E, Lund JS (1983) Clinical immunity after neonatal rotavirus infection. A prospective longitudinal study in young children. N Engl J Med 309: 72-76.

476. Bishop RF, Bugg HC, Masendycz PJ, Lund JS, Gorrell RJ, et al. (1996) Serum, fecal, and breast milk rotavirus antibodies as indices of infection in mother-infant pairs. J Infect Dis 174 Suppl 1: S22-29.

477. Bishop RF, Cameron DJ, Veenstra AA, Barnes GL (1979) Diarrhea and rotavirus infection associated with differing regimens for postnatal care of newborn babies. J Clin Microbiol 9: 525-529.

478. Bishop RF, Cipriani E, Lund JS, Barnes GL, Hosking CS (1984) Estimation of rotavirus immunoglobulin G antibodies in human serum samples by enzyme-linked immunosorbent assay: expression of results as units derived from a standard curve. J Clin Microbiol 19: 447-452.

479. Bishop RF, Hewstone AS, Davidson GP, Townley RR, Holmes IH, et al. (1976) An epidemic of diarrhoea in human neonates involving a reovirus-like agent and 'enteropathogenic' serotypes of Escherichia coli. J Clin Pathol 29: 46-49.

480. Bishop RF, Masendycz PJ, Bugg HC, Carlin JB, Barnes GL (2001) Epidemiological patterns of rotaviruses causing severe gastroenteritis in young children throughout Australia from 1993 to 1996. J Clin Microbiol 39: 1085-1091.

481. Bishop RF, Tzipori SR, Coulson BS, Unicomb LE, Albert MJ, et al. (1986) Heterologous protection against rotavirus-induced disease in gnotobiotic piglets. J Clin Microbiol 24: 1023-1028.

482. Bishop RF, Unicomb LE, Barnes GL (1991) Epidemiology of rotavirus serotypes in Melbourne, Australia, from 1973 to 1989. J Clin Microbiol 29: 862-868.

483. Bishop RF, Unicomb LE, Soenarto Y, Suwardji H, Ristanto, et al. (1989) Rotavirus serotypes causing acute diarrhoea in hospitalized children in Yogyakarta, Indonesia during 1978-1979. Arch Virol 107: 207-213.

484. Biswas R, Lyon DJ, Nelson EA, Lau D, Lewindon PJ (1996) Aetiology of acute diarrhoea in hospitalized children in Hong Kong. Trop Med Int Health 1: 679-683.

485. Bittencourt JA, Arbo E, Malysz AS, Oravec R, Dias C (2000) Seasonal and age distribution of rotavirus infection in Porto Alegre--Brazil. Braz J Infect Dis 4: 279-283.

486. Bjorkman C, Svensson C, Christensson B, de Verdier K (2003) Cryptosporidium parvum and Giardia intestinalis in calf diarrhoea in Sweden. Acta Vet Scand 44: 145-152.

487. Black RE (1986) Pathogens that cause travelers' diarrhea in Latin America and Africa. Rev Infect Dis 8 Suppl 2: S131-135.

488. Black RE (1990) Epidemiology of travelers' diarrhea and relative importance of various pathogens. Rev Infect Dis 12 Suppl 1: S73-79.

489. Black RE (1991) Would control of childhood infectious diseases reduce malnutrition? Acta Paediatr Scand Suppl 374: 133-140.

490. Black RE, Brown KH, Becker S (1984) Effects of diarrhea associated with specific enteropathogens on the growth of children in rural Bangladesh. Pediatrics 73: 799-805.

491. Black RE, Dykes AC, Anderson KE, Wells JG, Sinclair SP, et al. (1981) Handwashing to prevent diarrhea in day-care centers. Am J Epidemiol 113: 445-451.

492. Black RE, Greenberg HB, Kapikian AZ, Brown KH, Becker S (1982) Acquisition of serum antibody to Norwalk Virus and rotavirus and relation to diarrhea in a longitudinal study of young children in rural Bangladesh. J Infect Dis 145: 483-489.

493. Black RE, Lopez de Romana G, Brown KH, Bravo N, Bazalar OG, et al. (1989) Incidence and etiology of infantile diarrhea and major routes of transmission in Huascar, Peru. Am J Epidemiol 129: 785-799.

494. Black RE, Merson MH, Eusof A, Huq I, Pollard R (1984) Nutritional status, body size and severity of diarrhoea associated with rotavirus or enterotoxigenic Escherichia coli. J Trop Med Hyg 87: 83-89.

495. Black RE, Merson MH, Huq I, Alim AR, Yunus M (1981) Incidence and severity of rotavirus and Escherichia coli diarrhoea in rural Bangladesh. Implications for vaccine development. Lancet 1: 141-143.

496. Black RE, Merson MH, Rahman AS, Yunus M, Alim AR, et al. (1980) A two-year study of bacterial, viral, and parasitic agents associated with diarrhea in rural Bangladesh. J Infect Dis 142: 660-664.

497. Black RE, Merson MH, Taylor PR, Yolken RH, Sack DA (1981) Glucose vs sucrose in oral rehydration solutions for infants and young children with rotavirus-associated diarrhea. Pediatrics 67: 79-83.

498. Black S (2001) Perspectives on the design and analysis of prelicensure trials: bridging the gap to postlicensure studies. Clin Infect Dis 33 Suppl 4: S323-326.

499. Blackhall J, Bellinzoni R, Mattion N, Estes MK, La Torre JL, et al. (1992) A bovine rotavirus serotype 1: serologic characterization of the virus and nucleotide sequence determination of the structural glycoprotein VP7 gene. Virology 189: 833-837.

500. Blacklow NR, Cukor G (1981) Viral gastroenteritis. N Engl J Med 304: 397-406.

501. Blacklow NR, Greenberg HB (1991) Viral gastroenteritis. N Engl J Med 325: 252-264.

502. Blake PA, Ramos S, MacDonald KL, Rassi V, Gomes TA, et al. (1993) Pathogen-specific risk factors and protective factors for acute diarrheal disease in urban Brazilian infants. J Infect Dis 167: 627-632.

503. Blakelock RT, Beasley SW (2003) Infection and the gut. Semin Pediatr Surg 12: 265-274.

504. Blanchard H, Yu X, Coulson BS, von Itzstein M (2007) Insight into host cell carbohydrate-recognition by human and porcine rotavirus from crystal structures of the virion spike associated carbohydrate-binding domain (VP8*). J Mol Biol 367: 1215-1226.

505. Blanco J, Gonzalez EA, Blanco M, Garabal JI, Alonso MP, et al. (1991) Enterotoxigenic Escherichia coli associated with infant diarrhoea in Galicia, north-western Spain. J Med Microbiol 35: 162-167.

506. Blaskovic PJ, Kuderewko O, McLaughlin B, Yong DC, Ball FR (1980) Rapid diagnosis by electron microscopy of nonbacterial gastroenteritis in children. Can Med Assoc J 122: 150, 152.

507. Blaufuss H (1983) Rotavirus infections in a pediatric clinic. Eur J Clin Microbiol 2: 51-53.

508. Block SL, Vesikari T, Goveia MG, Rivers SB, Adeyi BA, et al. (2007) Efficacy, immunogenicity, and safety of a pentavalent human-bovine (WC3) reassortant rotavirus vaccine at the end of shelf life. Pediatrics 119: 11-18.

509. Blomqvist M, Juhela S, Erkkila S, Korhonen S, Simell T, et al. (2002) Rotavirus infections and development of diabetes-associated autoantibodies during the first 2 years of life. Clin Exp Immunol 128: 511-515.

510. Blutt SE, Conner ME (2007) Rotavirus: to the gut and beyond! Curr Opin Gastroenterol 23: 39-43.

511. Blutt SE, Kirkwood CD, Parreno V, Warfield KL, Ciarlet M, et al. (2003) Rotavirus antigenaemia and viraemia: a common event? Lancet 362: 1445-1449.

512. Blutt SE, Matson DO, Crawford SE, Staat MA, Azimi P, et al. (2007) Rotavirus antigenemia in children is associated with viremia. PLoS Med 4: e121.

513. Blutt SE, Warfield KL, Lewis DE, Conner ME (2002) Early response to rotavirus infection involves massive B cell activation. J Immunol 168: 5716-5721.

514. Bo MQ, Zhang FR (1993) [Xi xie ting in the treatment of infantile diarrhea]. Zhongguo Zhong Xi Yi Jie He Za Zhi 13: 343-344, 324.

515. Boccia D, Stolfi I, Lana S, Moro ML (2001) Nosocomial necrotising enterocolitis outbreaks: epidemiology and control measures. Eur J Pediatr 160: 385-391.

516. Bockemuhl J (1985) [Epidemiology, etiology and laboratory diagnosis of infectious diarrhea diseases in the tropics]. Immun Infekt 13: 269-275.

517. Bodhidatta L, Lan NT, Hien BT, Lai NV, Srijan A, et al. (2007) Rotavirus disease in young children from Hanoi, Vietnam. Pediatr Infect Dis J 26: 325-328.

518. Boedeker EC (2005) Enteric infections. Curr Opin Gastroenterol 21: 1-3.

519. Boer LA, Cinquetti M (1991) [Clinical-etiological correlations in 94 cases of acute infantile diarrhea]. Minerva Gastroenterol Dietol 37: 211-218.

520. Bogdanovic G, Eriksson M, Bennet R, Lindekrantz E (2008) [Rotavirus vaccines no life-savers. Markedly reduced morbidity in families with children]. Lakartidningen 105: 1178-1180.

521. Bogstedt AK, Johansen K, Hatta H, Kim M, Casswall T, et al. (1996) Passive immunity against diarrhoea. Acta Paediatr 85: 125-128.

522. Bohl EH (1977) Review of rotavirus infections of man and animals. Proc Annu Meet U S Anim Health Assoc: 474-481.

523. Bohl EH (1979) Rotaviral diarrhea in pigs: brief review. J Am Vet Med Assoc 174: 613-615.

524. Bohl EH, Kohler EM, Saif LJ, Cross RF, Agnes AG, et al. (1978) Rotavirus as a cause of diarrhea in pigs. J Am Vet Med Assoc 172: 458-463.

525. Bohl EH, Saif LJ, Theil KW, Agnes AG, Cross RF (1982) Porcine pararotavirus: detection, differentiation from rotavirus, and pathogenesis in gnotobiotic pigs. J Clin Microbiol 15: 312-319.

526. Bohl EH, Theil KW, Saif LJ (1984) Isolation and serotyping of porcine rotaviruses and antigenic comparison with other rotaviruses. J Clin Microbiol 19: 105-111.

527. Bojsen A, Buesa J, Montava R, Kvistgaard AS, Kongsbak MB, et al. (2007) Inhibitory activities of bovine macromolecular whey proteins on rotavirus infections in vitro and in vivo. J Dairy Sci 90: 66-74.

528. Bok K, Castagnaro N, Borsa A, Nates S, Espul C, et al. (2001) Surveillance for rotavirus in Argentina. J Med Virol 65: 190-198.

529. Bok K, Castagnaro NC, Diaz NE, Borsa A, Cagnoli MR, et al. (1999) [Rotavirus laboratory network: results after one year of observation]. Rev Argent Microbiol 31: 1-12.

530. Bok K, Matson DO, Gomez JA (2002) Genetic variation of capsid protein VP7 in genotype g4 human rotavirus strains: simultaneous emergence and spread of different lineages in Argentina. J Clin Microbiol 40: 2016-2022.

531. Bok K, Palacios G, Sijvarger K, Matson D, Gomez J (2001) Emergence of G9 P[6] human rotaviruses in Argentina: phylogenetic relationships among G9 strains. J Clin Microbiol 39: 4020-4025.

532. Bokovoi AG, Nisevich NI (1992) [The principles of the diagnosis of acute intestinal infections caused by opportunistic bacteria in children]. Pediatriia: 11-15.

533. Bolivar R, Conklin RH, Vollet JJ, Pickering LK, DuPont HL, et al. (1978) Rotavirus in travelers' diarrhea: study of an adult student population in Mexico. J Infect Dis 137: 324-327.

534. Bon F, Fascia P, Dauvergne M, Tenenbaum D, Planson H, et al. (1999) Prevalence of group A rotavirus, human calicivirus, astrovirus, and adenovirus type 40 and 41 infections among children with acute gastroenteritis in Dijon, France. J Clin Microbiol 37: 3055-3058.

535. Bon F, Fromantin C, Aho S, Pothier P, Kohli E (2000) G and P genotyping of rotavirus strains circulating in france over a three-year period: detection of G9 and P[6] strains at low frequencies. The AZAY Group. J Clin Microbiol 38: 1681-1683.

536. Bonno M, Higashigawa M, Nakano T, Miyahara M, Azuma E, et al. (1998) Acute myositis with transient decrease of albumin, immunoglobulin, and complement following rotavirus gastroenteritis. Acta Paediatr Jpn 40: 82-84.

537. Bonzano L, Sabucco M (1990) [Acute gastroenteritis in childhood. Epidemiological comments on a hospital case series]. Minerva Med 81: 37-40.

538. Borgdorff MW, Koopmans MP, Goosen ES, Sprenger MJ (1995) Surveillance of gastroenteritis. Lancet 346: 842-843.

539. Borghan MA, Mori Y, El-Mahmoudy AB, Ito N, Sugiyama M, et al. (2007) Induction of nitric oxide synthase by rotavirus enterotoxin NSP4: implication for rotavirus pathogenicity. J Gen Virol 88: 2064-2072.

540. Borowitz SM (2005) Are antiemetics helpful in young children suffering from acute viral gastroenteritis? Arch Dis Child 90: 646-648.

541. Bos P, Kirsten M, Cronje RE, Steele AD (1995) Monitoring of rotavirus infection in a paediatric hospital by RNA electrophoresis. S Afr Med J 85: 887-891.

542. Bos P, Mnisi YN, Steele AD (1992) The molecular epidemiology of rotavirus infection in Ga-Rankuwa, southern Africa. Cent Afr J Med 38: 286-290.

543. Bosch A, Pinto RM, Comas J, Abad FX (2004) Detection of infectious rotaviruses by flow cytometry. Methods Mol Biol 268: 61-68.

544. Bosch A, Pinto RM, Jofre J (1988) Non-seasonal distribution of rotavirus in Barcelona raw sewage. Zentralbl Bakteriol Mikrobiol Hyg [B] 186: 273-277.

545. Boshuizen JA, Reimerink JH, Korteland-van Male AM, van Ham VJ, Bouma J, et al. (2005) Homeostasis and function of goblet cells during rotavirus infection in mice. Virology 337: 210-221.

546. Boshuizen JA, Reimerink JH, Korteland-van Male AM, van Ham VJ, Koopmans MP, et al. (2003) Changes in small intestinal homeostasis, morphology, and gene expression during rotavirus infection of infant mice. J Virol 77: 13005-13016.

547. Boshuizen JA, Rossen JW, Sitaram CK, Kimenai FF, Simons-Oosterhuis Y, et al. (2004) Rotavirus enterotoxin NSP4 binds to the extracellular matrix proteins laminin-beta3 and fibronectin. J Virol 78: 10045-10053.

548. Both GW, Andrew ME, Boyle DB, Coupar BE, Bellamy AR (1992) Relocation of antigens to the cell surface membrane can enhance immune stimulation and protection. Immunol Cell Biol 70 ( Pt 1): 73-78.

549. Both GW, Lockett LJ, Janardhana V, Edwards SJ, Bellamy AR, et al. (1993) Protective immunity to rotavirus-induced diarrhoea is passively transferred to newborn mice from naive dams vaccinated with a single dose of a recombinant adenovirus expressing rotavirus VP7sc. Virology 193: 940-950.

550. Bothig B, Schulze P, Schreier E, Diedrich S, Michel S (1989) Atypical human rotaviruses in the G.D.R. Acta Virol 33: 320-326.

551. Botic T, Klingberg TD, Weingartl H, Cencic A (2007) A novel eukaryotic cell culture model to study antiviral activity of potential probiotic bacteria. Int J Food Microbiol 115: 227-234.

552. Bourdett-Stanziola L, Jimenez C, Ortega-Barria E (2008) Diversity of human rotavirus G and P genotypes in Panama, Costa Rica, and the Dominican Republic. Am J Trop Med Hyg 79: 921-924.

553. Bourgeois AL, Gardiner CH, Thornton SA, Batchelor RA, Burr DH, et al. (1993) Etiology of acute diarrhea among United States military personnel deployed to South America and west Africa. Am J Trop Med Hyg 48: 243-248.

554. Bowdre JH (1983) Viral gastroenteritis and laboratory detection of rotavirus. Am J Med Technol 49: 665-668.

555. Bowen-Jones J (1989) Infection and cross-infection in a paediatric gastro-enteritis unit. Curationis 12: 30-33.

556. Bowman GD, Nodelman IM, Levy O, Lin SL, Tian P, et al. (2000) Crystal structure of the oligomerization domain of NSP4 from rotavirus reveals a core metal-binding site. J Mol Biol 304: 861-871.

557. Bozdayi G, Dogan B, Dalgic B, Bostanci I, Sari S, et al. (2008) Diversity of human rotavirus G9 among children in Turkey. J Med Virol 80: 733-740.

558. Brade L (1979) [Rotaviruses: origin of acute gastroenteriteis in newborns and infants]. Dtsch Med Wochenschr 104: 991-993.

559. Brady MT, Pacini DL, Budde CT, Connell MJ (1989) Diagnostic studies of nosocomial diarrhea in children: assessing their use and value. Am J Infect Control 17: 77-82.

560. Brandt CD, Arndt CW, Evans GL, Kim HW, Stallings EP, et al. (1987) Evaluation of a latex test for rotavirus detection. J Clin Microbiol 25: 1800-1802.

561. Brandt CD, Kim HW, Rodriguez WJ, Arrobio JO, Jeffries BC, et al. (1982) Rotavirus gastroenteritis and weather. J Clin Microbiol 16: 478-482.

562. Brandt CD, Kim HW, Rodriguez WJ, Arrobio JO, Jeffries BC, et al. (1986) Simultaneous infections with different enteric and respiratory tract viruses. J Clin Microbiol 23: 177-179.

563. Brandt CD, Kim HW, Rodriguez WJ, Arrobio JO, Jeffries BC, et al. (1983) Pediatric viral gastroenteritis during eight years of study. J Clin Microbiol 18: 71-78.

564. Brandt CD, Kim HW, Rodriguez WJ, Thomas L, Yolken RH, et al. (1981) Comparison of direct electron microscopy, immune electron microscopy, and rotavirus enzyme-linked immunosorbent assay for detection of gastroenteritis viruses in children. J Clin Microbiol 13: 976-981.

565. Brandt CD, Kim HW, Yolken RH, Kapikian AZ, Arrobio JO, et al. (1979) Comparative epidemiology of two rotavirus serotypes and other viral agents associated with pediatric gastroenteritis. Am J Epidemiol 110: 243-254.

566. Brassard J, Seyer K, Houde A, Simard C, Trottier YL (2005) Concentration and detection of hepatitis A virus and rotavirus in spring water samples by reverse transcription-PCR. J Virol Methods 123: 163-169.

567. Braun OH (1979) [Virusenteritis in childhood (author's transl)]. Klin Padiatr 191: 245-260.

568. Breer C, Wunderli W, Lee C, Weisser E, Schopfer K (1985) [Rotavirus and pararotavirus infections in adults. Analysis of a nosocomial infection and some sporadic cases]. Schweiz Med Wochenschr 115: 1530-1535.

569. Breinig MK, Zitelli B, Starzl TE, Ho M (1987) Epstein-Barr virus, cytomegalovirus, and other viral infections in children after liver transplantation. J Infect Dis 156: 273-279.

570. Brenner J, Elad D, Bernstein M, Dagoni I, Palfi V, et al. (2005) The detection of an unidentified type of adenovirus in the stools of calves with weak calf syndrome by use of a commercial kit designed for the detection of human adenoviruses. J Vet Med B Infect Dis Vet Public Health 52: 98-101.

571. Brenner-Zada G, Bistrizer Z, Goldman M (2005) [Hyperuricemia following rotavirus infection]. Harefuah 144: 534-535, 600.

572. Bresee J, Fang ZY, Wang B, Nelson EA, Tam J, et al. (2004) First report from the Asian Rotavirus Surveillance Network. Emerg Infect Dis 10: 988-995.

573. Bresee JS, El Arifeen S, Azim T, Chakraborty J, Mounts AW, et al. (2001) Safety and immunogenicity of tetravalent rhesus-based rotavirus vaccine in Bangladesh. Pediatr Infect Dis J 20: 1136-1143.

574. Bresee JS, Hummelman E, Nelson EA, Glass RI (2005) Rotavirus in Asia: the value of surveillance for informing decisions about the introduction of new vaccines. J Infect Dis 192 Suppl 1: S1-5.

575. Brewster DR, Greenwood BM (1993) Seasonal variation of paediatric diseases in The Gambia, west Africa. Ann Trop Paediatr 13: 133-146.

576. Bricout F (1992) [Viral diarrheas]. Presse Med 21: 309-314.

577. Bricout F, Dussaix E, Nicolas JC, Huraux JM, Befekadu E (1977) [Infantile diarrhea and rotavirus (author's transl)]. Pathol Biol (Paris) 25: 43-45.

578. Bridger JC (1980) Detection by electron microscopy of caliciviruses, astroviruses and rotavirus-like particles in the faeces of piglets with diarrhoea. Vet Rec 107: 532-533.

579. Bridger JC (1987) Novel rotaviruses in animals and man. Ciba Found Symp 128: 5-23.

580. Bridger JC, Brown JF (1981) Development of immunity to porcine rotavirus in piglets protected from disease by bovine colostrum. Infect Immun 31: 906-910.

581. Bridger JC, Brown JF (1984) Antigenic and pathogenic relationships of three bovine rotaviruses and a porcine rotavirus. J Gen Virol 65 ( Pt 7): 1151-1158.

582. Bridger JC, Burke B, Beards GM, Desselberger U (1992) The pathogenicity of two porcine rotaviruses differing in their in vitro growth characteristics and genes 4. J Gen Virol 73 ( Pt 11): 3011-3015.

583. Bridger JC, Hall GA, Parsons KR (1992) A study of the basis of virulence variation of bovine rotaviruses. Vet Microbiol 33: 169-174.

584. Bridger JC, Tauscher GI, Desselberger U (1998) Viral determinants of rotavirus pathogenicity in pigs: evidence that the fourth gene of a porcine rotavirus confers diarrhea in the homologous host. J Virol 72: 6929-6931.

585. Bridger JC, Woode GN (1975) Neonatal calf diarrhoea: identification of a reovirus-like (rotavirus) agent in faeces by immunofluorescence and immune electron microscopy. Br Vet J 131: 528-535.

586. Bridger JC, Woode GN (1976) Characterization of two particle types of calf rotavirus. J Gen Virol 31: 245-250.

587. Bridger JC, Woode GN, Jones JM, Flewett TH, Bryden AS, et al. (1975) Transmission of human rotaviruses to gnotobiotic piglets. J Med Microbiol 8: 565-569.

588. Brook I (2008) Microbiology and management of neonatal necrotizing enterocolitis. Am J Perinatol 25: 111-118.

589. Brooks JB (1986) Review of frequency-pulsed electron-capture gas-liquid chromatography studies of diarrheal diseases caused by members of the family Enterobacteriaceae, Clostridium difficile, and rotavirus. J Clin Microbiol 24: 687-691.

590. Brooks JB, Basta MT, el Kholy AM (1985) Studies of metabolites in diarrheal stool specimens containing Shigella species by frequency-pulsed electron capture gas-liquid chromatography. J Clin Microbiol 21: 599-606.

591. Brooks JB, Basta MT, el Kholy AM, Moss CW (1984) Rapid differentiation of enterotoxigenic Escherichia coli that produce heat-stable and heat-labile toxins by frequency-pulsed electron capture gas-liquid chromatography analysis of diarrheal stool specimens. J Clin Microbiol 20: 1145-1153.

592. Brooks JB, Nunez-Montiel OL, Basta MT, Hierholzer JC (1984) Studies of stools from pseudomembranous colitis, rotaviral, and other diarrheal syndromes by frequency-pulsed electron capture gas-liquid chromatography. J Clin Microbiol 20: 549-560.

593. Brooks R, Brown L, Franklin R (1988) Comparison of a protein-stabilized Rotazyme II test, with standard Rotazyme II, and electron microscopy for detection of rotavirus. Diagn Microbiol Infect Dis 11: 205-208.

594. Brooks RG, Brown L, Franklin RB (1989) Comparison of a new rapid test (TestPack Rotavirus) with standard enzyme immunoassay and electron microscopy for the detection of rotavirus in symptomatic hospitalized children. J Clin Microbiol 27: 775-777.

595. Broome RL, Vo PT, Ward RL, Clark HF, Greenberg HB (1993) Murine rotavirus genes encoding outer capsid proteins VP4 and VP7 are not major determinants of host range restriction and virulence. J Virol 67: 2448-2455.

596. Broor S, Dar L (1992) Vaccines against enteric infections. Trop Gastroenterol 13: 96-101.

597. Broor S, Ghosh D, Mathur P (2003) Molecular epidemiology of rotaviruses in India. Indian J Med Res 118: 59-67.

598. Broor S, Husain M, Chatterjee B, Chakraborty A, Seth P (1993) Temporal variation in the distribution of rotavirus electropherotypes in Delhi, India. J Diarrhoeal Dis Res 11: 14-18.

599. Broor S, Husain M, Chatterjee B, Chakraborty A, Seth P (1995) Direct detection and characterization of rotavirus into subgroups by dot blot hybridization and correlation with 'long' and 'short' electropherotypes. Clin Diagn Virol 3: 29-38.

600. Broor S, Singh V (1984) Viral gastroenteritis. Indian J Gastroenterol 3: 225-229.

601. Broor S, Singh V, Venkateshwarlu, Gautam S, Mehta S, et al. (1985) Rotavirus diarrhoea in children in Chandigarh, India. J Diarrhoeal Dis Res 3: 158-161.

602. Brown DW, Campbell L, Tomkins DS, Hambling MH (1989) School outbreak of gastroenteritis due to atypical rotavirus. Lancet 2: 737-738.

603. Brown DW, Mathan MM, Mathew M, Martin R, Beards GM, et al. (1988) Rotavirus epidemiology in Vellore, south India: group, subgroup, serotype, and electrophoretype. J Clin Microbiol 26: 2410-2414.

604. Brown KA, Kriss JA, Moser CA, Wenner WJ, Offit PA (2000) Circulating rotavirus-specific antibody-secreting cells (ASCs) predict the presence of rotavirus-specific ASCs in the human small intestinal lamina propria. J Infect Dis 182: 1039-1043.

605. Brown KA, Offit PA (1998) Rotavirus-specific proteins are detected in murine macrophages in both intestinal and extraintestinal lymphoid tissues. Microb Pathog 24: 327-331.

606. Browning GF, Begg AP (1996) Prevalence of G and P serotypes among equine rotaviruses in the faeces of diarrhoeic foals. Arch Virol 141: 1077-1089.

607. Browning GF, Chalmers RM, Fitzgerald TA, Corley KT, Campbell I, et al. (1992) Rotavirus serotype G3 predominates in horses. J Clin Microbiol 30: 59-62.

608. Browning GF, Chalmers RM, Fitzgerald TA, Snodgrass DR (1991) Serological and genomic characterization of L338, a novel equine group A rotavirus G serotype. J Gen Virol 72 ( Pt 5): 1059-1064.

609. Browning GF, Chalmers RM, Snodgrass DR, Batt RM, Hart CA, et al. (1991) The prevalence of enteric pathogens in diarrhoeic thoroughbred foals in Britain and Ireland. Equine Vet J 23: 405-409.

610. Brunet JP, Cotte-Laffitte J, Linxe C, Quero AM, Geniteau-Legendre M, et al. (2000) Rotavirus infection induces an increase in intracellular calcium concentration in human intestinal epithelial cells: role in microvillar actin alteration. J Virol 74: 2323-2332.

611. Brunet JP, Jourdan N, Cotte-Laffitte J, Linxe C, Geniteau-Legendre M, et al. (2000) Rotavirus infection induces cytoskeleton disorganization in human intestinal epithelial cells: implication of an increase in intracellular calcium concentration. J Virol 74: 10801-10806.

612. Brunser O, Espinoza J, Figueroa G, Araya M, Spencer E, et al. (1992) Field trial of an infant formula containing anti-rotavirus and anti-Escherichia coli milk antibodies from hyperimmunized cows. J Pediatr Gastroenterol Nutr 15: 63-72.

613. Bruscolini F, Pianetti A, Baffone W, Romanini I, Brandi G, et al. (1985) [Prevalence of rotaviruses, Campylobacter and pathogenic enterobacteria in feces of subjects residing in the Urbino area]. Nuovi Ann Ig Microbiol 36: 367-379.

614. Brussieux J, Boisivon A, Michelon B (1985) [Prospective study of rotavirus infection in a maternity unit. Demonstration of a nosocomial infection]. Arch Fr Pediatr 42: 687-689.

615. Brussow H, Benitez O, Uribe F, Sidoti J, Rosa K, et al. (1993) Rotavirus-inhibitory activity in serial milk samples from Mexican women and rotavirus infections in their children during their first year of life. J Clin Microbiol 31: 593-597.

616. Brussow H, Clark HF, Sidoti J (1991) Prevalence of serum neutralizing antibody to serotype 9 rotavirus WI61 in children from South America and central Europe. J Clin Microbiol 29: 208-211.

617. Brussow H, Eichhorn W, Rohwedder A, Snodgrass D, Sidoti J (1991) Cattle develop neutralizing antibodies to rotavirus serotypes which could not be isolated from faeces of symptomatic calves. J Gen Virol 72 ( Pt 7): 1559-1567.

618. Brussow H, Gerna G, Sidoti J, Sarasini A (1992) Neutralizing serum antibodies to serotype 6 human rotaviruses PA151 and PA169 in Ecuadorian and German children. J Clin Microbiol 30: 911-914.

619. Brussow H, Hilpert H, Walther I, Sidoti J, Mietens C, et al. (1987) Bovine milk immunoglobulins for passive immunity to infantile rotavirus gastroenteritis. J Clin Microbiol 25: 982-986.

620. Brussow H, Nakagomi O, Gerna G, Eichhorn W (1992) Isolation of an avianlike group A rotavirus from a calf with diarrhea. J Clin Microbiol 30: 67-73.

621. Brussow H, Offit PA, Gerna G, Bruttin A, Sidoti J (1990) Polypeptide specificity of antiviral serum antibodies in children naturally infected with human rotavirus. J Virol 64: 4130-4136.

622. Brussow H, Rahim H, Freire W (1992) Epidemiological analysis of serologically determined rotavirus and enterotoxigenic Escherichia coli infections in Ecuadorian children. J Clin Microbiol 30: 1585-1587.

623. Brussow H, Sidoti J (1991) Antibody to serotype 8 rotavirus in Ecuadorian and German children. Epidemiol Infect 106: 415-420.

624. Brussow H, Sidoti J, Barclay D, Sotek J, Dirren H, et al. (1990) Prevalence and serotype specificity of rotavirus antibodies in different age groups of Ecuadorian infants. J Infect Dis 162: 615-620.

625. Brussow H, Sidoti J, Dirren H, Freire WB (1995) Effect of malnutrition in Ecuadorian children on titers of serum antibodies to various microbial antigens. Clin Diagn Lab Immunol 2: 62-68.

626. Brussow H, Sidoti J, Rahim H, Dirren H, Freire W (1991) Infectious gastroenteritis does not act as a triggering mechanism for the synthesis of serum IgG antibody to beta-lactoglobulin. J Pediatr Gastroenterol Nutr 13: 402-408.

627. Brussow H, Sidoti J, Sure K, Werchau H (1994) Coproantibodies to rotavirus serotype 1 infection in German children. J Diarrhoeal Dis Res 12: 194-199.

628. Brussow H, Werchau H, Lerner L, Mietens C, Liedtke W, et al. (1988) Seroconversion patterns to four human rotavirus serotypes in hospitalized infants with acute rotavirus gastroenteritis. J Infect Dis 158: 588-595.

629. Bryden AS, Davies HA, Hadley RE, Flewett TH (1975) Rotavirus enteritis in the West Midlands during 1974. Lancet 2: 241-243.

630. Bryden AS, Davies HA, Thouless ME, Flewitt TH (1977) Diagnosis of rotavirus infection by cell culture. J Med Microbiol 10: 121-125.

631. Bryden AS, Thouless ME, Flewett TH (1976) Rotavirus and rabbits. Vet Rec 99: 323.

632. Bryden AS, Thouless ME, Hall CJ, Flewett TH, Wharton BA, et al. (1982) Rotavirus infections in a special-care baby unit. J Infect 4: 43-48.

633. Bucardo F, Karlsson B, Nordgren J, Paniagua M, Gonzalez A, et al. (2007) Mutated G4P[8] rotavirus associated with a nationwide outbreak of gastroenteritis in Nicaragua in 2005. J Clin Microbiol 45: 990-997.

634. Bucher B, Aebi C (2006) Population-based epidemiology of rotavirus hospitalisations in Switzerland. Swiss Med Wkly 136: 726-731.

635. Buchrieser C, Sixl W, Buchrieser V, Miorini T, Stunzner D, et al. (1988) Investigation of human stool samples from the Cape Verde Islands (district Santa Cruz/Santiago). Geogr Med Suppl 1: 61-64.

636. Buesa J, Colomina J, Raga J, Villanueva A, Prat J (1996) Evaluation of reverse transcription and polymerase chain reaction (RT/PCR) for the detection of rotaviruses: applications of the assay. Res Virol 147: 353-361.

637. Buesa J, de Souza CO, Asensi M, Martinez C, Prat J, et al. (2000) VP7 and VP4 genotypes among rotavirus strains recovered from children with gastroenteritis over a 3-year period in Valencia, Spain. Eur J Epidemiol 16: 501-506.

638. Buettcher M, Baer G, Bonhoeffer J, Schaad UB, Heininger U (2007) Three-year surveillance of intussusception in children in Switzerland. Pediatrics 120: 473-480.

639. Buffet-Janvresse C, Bernard E, Magrad H (1976) [Responsibility of the rotavirus in infantile diarrheas]. Nouv Presse Med 5: 1249-1251.

640. Bugarcic A, Taylor JA (2006) Rotavirus nonstructural glycoprotein NSP4 is secreted from the apical surfaces of polarized epithelial cells. J Virol 80: 12343-12349.

641. Buigues RP, Duval B, Rochette L, Boulianne N, Douville-Fradet M, et al. (2002) Hospitalizations for diarrhea in Quebec children from 1985 to 1998: Estimates of rotavirus-associated diarrhea. Can J Infect Dis 13: 239-244.

642. Buitenwerf J, Nuilwijk-van Alphen M, Schaap GJ (1983) Characterization of rotaviral RNA isolated from children with gastroenteritis in two hospitals in Rotterdam. J Med Virol 12: 71-78.

643. Bukenya GB, Kaiser R, Nneka N (1990) Rotavirus from children of an urban settlement of Papua New Guinea. J Trop Pediatr 36: 66-68.

644. Bukholm G (1988) Human rotavirus infection enhances invasiveness of enterobacteria in MA-104 cells. Apmis 96: 1118-1124.

645. Bukrinskaia AG (1986) [Rotaviruses: their structure, chemical composition and biological properties]. Vopr Virusol 31: 645-654.

646. Bukrinskaia AG, Gracheva NM, Starov AI, Moisiadi SA, Blokhina TA (1986) [Rapid diagnosis of rotavirus infections by RNA electrophoresis on polyacrylamide gel]. Vopr Virusol 31: 197-200.

647. Bukrinskaia AG, Sharova NK, Sergeev OV, Vasil'ev B, Ten NL (1990) [The RNA electrophoretypes of the rotaviruses circulating in Moscow and Leningrad in the winter of 1987-1988]. Vopr Virusol 35: 216-218.

648. Bukrinskaia AG, Timina VP, Kitsak V, Pavlova LA, Moisiadi SA (1988) [Development of test systems of immunoenzyme analysis for the rapid diagnosis of rotavirus infection]. Vopr Virusol 33: 444-447.

649. Bulakbasi N, Kocaoglu M, Tayfun C, Ucoz T (2006) Transient splenial lesion of the corpus callosum in clinically mild influenza-associated encephalitis/encephalopathy. AJNR Am J Neuroradiol 27: 1983-1986.

650. Buller CR, Moxley RA (1988) Natural infection of porcine ileal dome M cells with rotavirus and enteric adenovirus. Vet Pathol 25: 516-517.

651. Burdick JR, Levy NS, Klimek EM (1986) Incidence of rotavirus infection in pediatric outpatients with gastroenteritis. J Am Osteopath Assoc 86: 788-792.

652. Burgio GR, Scotta MS, Notarangelo LD, Viola S, De Amici M (1988) [Pro-allergy role of infection. A component of the mode of reacting]. Pediatr Med Chir 10: 203-211.

653. Burke V, Gracey M, Masters P (1985) Rotavirus in children. J Infect Dis 152: 646-647.

654. Burke V, Gracey M, Robinson J, Peck D, Beaman J, et al. (1983) The microbiology of childhood gastroenteritis: Aeromonas species and other infective agents. J Infect Dis 148: 68-74.

655. Burki F, Mostl K, Spiegl E, Horvath E, Szekely H (1986) Reduction of rotavirus-, coronavirus- and E. coli-associated calf-diarrheas in a large-size dairy herd by means of dam vaccination with a triple-vaccine. Zentralbl Veterinarmed B 33: 241-252.

656. Burki F, Schusser G, Szekely H (1983) Clinical, virological and serological evaluation of the efficacy of peroral live rotavirus vaccination in calves kept under normal husbandry conditions. Zentralbl Veterinarmed B 30: 237-250.

657. Burns JW, Siadat-Pajouh M, Krishnaney AA, Greenberg HB (1996) Protective effect of rotavirus VP6-specific IgA monoclonal antibodies that lack neutralizing activity. Science 272: 104-107.

658. Burns JW, Welch SK, Nakata S, Estes MK (1989) Characterization of monoclonal antibodies to human group B rotavirus and their use in an antigen detection enzyme-linked immunosorbent assay. J Clin Microbiol 27: 245-250.

659. Burton EM, Mercado-Deane MG, Patel K (1994) Pneumatosis intestinalis in a child with AIDS and pseudomembranous colitis. Pediatr Radiol 24: 609-610.

660. Busato A, Lentze T, Hofer D, Burnens A, Hentrich B, et al. (1998) A case control study of potential enteric pathogens for calves raised in cow-calf herds. Zentralbl Veterinarmed B 45: 519-528.

661. Bussel J, Lalezari P, Hilgartner M, Partin J, Fikrig S, et al. (1983) Reversal of neutropenia with intravenous gammaglobulin in autoimmune neutropenia of infancy. Blood 62: 398-400.

662. Butchaiah G, Botner AG, Lund E (1984) Studies on the growth of bovine rotavirus in cell cultures. Zentralbl Veterinarmed B 31: 760-769.

663. Butler TC (1984) Viral diarrhoeas. J Diarrhoeal Dis Res 2: 137-141.

664. Buttery J (2006) A rotavirus vaccine for infants prevented rotavirus gastroenteritis with no increase in risk of intussusception. Evid Based Med 11: 113.

665. Buttery J (2007) A rotavirus vaccine for infants prevented rotavirus gastroenteritis with no increase in risk of intussusception. Arch Dis Child Educ Pract Ed 92: ep30.

666. Buttery JP, Kirkwood C (2007) Rotavirus vaccines in developed countries. Curr Opin Infect Dis 20: 253-258.

667. Butz AM, Fosarelli P, Dick J, Cusack T, Yolken R (1993) Prevalence of rotavirus on high-risk fomites in day-care facilities. Pediatrics 92: 202-205.

668. Buzinaro MG, Freitas PP, Kisiellius JJ, Ueda M, Jerez JA (2003) Identification of a bisegmented double-stranded RNA virus (picobirnavirus) in calf faeces. Vet J 166: 185-187.

669. Bywater RJ, Woode GN (1980) Oral fluid replacement by a glucose glycine electrolyte formulation in E coli and rotavirus diarrhoea in pigs. Vet Rec 106: 75-78.

670. Caceres DC, Estrada E, DeAntonio R, Pelaez D (2005) [Acute diarrheal disease: a public health challenge in Colombia]. Rev Panam Salud Publica 17: 6-14.

671. Caceres DC, Pelaez D, Sierra N, Estrada E, Sanchez L (2006) [Burden of rotavirus-related disease among children under five, Colombia, 2004]. Rev Panam Salud Publica 20: 9-21.

672. Caddell A (1997) The Children's Vaccine Initiative. Afr Health 20: 15.

673. Caeiro JP, Mathewson JJ, Smith MA, Jiang ZD, Kaplan MA, et al. (1999) Etiology of outpatient pediatric nondysenteric diarrhea: a multicenter study in the United States. Pediatr Infect Dis J 18: 94-97.

674. Calderon A, Macaya J, Avendano LF, Prenzel I, Ojeda JM, et al. (1980) [Acute diarrhea caused by rotavirus: various clinical aspects]. Rev Chil Pediatr 51: 113-116.

675. Calderon E, Espejo R, Gonzalez N, Hernandez M, Romero P, et al. (1978) [Epidemiological aspects of gastroenteritis dut to Rotavirus]. Bol Med Hosp Infant Mex 35: 45-55.

676. Calderon Marin A, Avendano LF, Varas Palma X, Vargas Munita S (1982) [Detection of rotavirus in hospitalized and ambulatory infants with acute diarrhea in Santiago de Chile]. Bol Med Hosp Infant Mex 39: 89-91.

677. Calderon-Jaimes E (1984) [Viral etiology of gastroenteritis]. Bol Med Hosp Infant Mex 41: 577-579.

678. Callejas D, Estevez J, Blitz-Dorfman L, Garcia D (1994) [Molecular epidemiology of subgroups and serotypes of rotavirus in children less than 4 years of age in the city of Maracaibo with a diarrheal syndrome]. Invest Clin 35: 3-17.

679. Callejas D, Estevez J, Porto-Espinoza L, Monsalve F, Costa-Leon L, et al. (1999) [Effect of climatic factors on the epidemiology of rotavirus infection in children under 5 years of age in the city of Maracaibo, Venezuela]. Invest Clin 40: 81-94.

680. Cama RI, Parashar UD, Taylor DN, Hickey T, Figueroa D, et al. (1999) Enteropathogens and other factors associated with severe disease in children with acute watery diarrhea in Lima, Peru. J Infect Dis 179: 1139-1144.

681. Camarota SC, de Azevedo Mda S, Martins RM, Barbosa AJ, Ferreira Junior PA, et al. (1992) [The occurrence of rotaviruses and adenoviruses in children up to 11 years old without diarrheal symptomatology in Goiania, Goias]. Rev Soc Bras Med Trop 25: 31-35.

682. Cameron DJ, Bishop RF, Davidson GP, Townley RR, Holmes IH, et al. (1975) Letter: Rotavirus infections in obstetric hospitals. Lancet 2: 124-125.

683. Cameron DJ, Bishop RF, Veenstra AA, Barnes GL (1978) Noncultivable viruses and neonatal diarrhea: fifteen-month survey in a newborn special care nursery. J Clin Microbiol 8: 93-98.

684. Cameron DJ, Bishop RF, Veenstra AA, Barnes GL, Holmes IH, et al. (1978) Pattern of shedding of two noncultivable viruses in stools of newborn babies. J Med Virol 2: 7-13.

685. Campbell C, Lang WR (1979) An epidemic of gastroenteritis in Auckland 1978. N Z Med J 90: 233-235.

686. Campbell RE, Lu W, White AK, Duhamel GE (1998) Molecular and immunologic characterization of group A bovine rotavirus field isolates with P8[11] spike protein. Arch Virol 143: 1021-1028.

687. Campos-Outcalt D (2007) Immunization update: latest recommendations from the CDC. J Fam Pract 56: 377-380.

688. Candeias JA, Fagundes-Neto U, Racz ML, Pedra MA, Ferreira VC, et al. (1989) Rotavirus identification in jejunal juice and stools of acute and chronic forms of infantile gastroenteritis. Braz J Med Biol Res 22: 833-839.

689. Candeias JA, Racz ML, Breviglieri JC, Rosenburg CP (1980) [Complement fixation test in the identification of human rotavirus]. Rev Saude Publica 14: 420-424.

690. Candeias JA, Racz ML, Travulsi LR, Murahowsky J (1989) Relative prevalence of rotavirus diarrhoea in children attending outpatient departments of hospitals and general practitioners in Sao Paulo, Brazil. J Diarrhoeal Dis Res 7: 24-27.

691. Candeias JA, Rosenburg CP, Racz ML (1978) [Identification of rotavirus by counter-immunoelectrophoresis in cases of infantile diarrhea]. Rev Saude Publica 12: 99-103.

692. Candia N, Parra GI, Chirico M, Velazquez G, Farina N, et al. (2003) Acute diarrhea in Paraguayan children population: detection of rotavirus electropherotypes. Acta Virol 47: 137-140.

693. Candy DC (1985) New enteric vaccines: application of new knowledge of receptors and recognition in enteric infections. Trans R Soc Trop Med Hyg 79: 577-580.

694. Candy DC (2003) Rotavirus in the blood--another turn of the wheel. Lancet 362: 1429.

695. Candy DC (2007) Rotavirus infection: a systemic illness? PLoS Med 4: e117.

696. Cao D, Santos N, Jones RW, Tatsumi M, Gentsch JR, et al. (2008) The VP7 genes of two G9 rotaviruses isolated in 1980 from diarrheal stool samples collected in Washington, DC, are unique molecularly and serotypically. J Virol 82: 4175-4179.

697. Capano G, Guandalini S, Guarino A, Caprioli A, Falbo V, et al. (1984) Enteric infections, cow's milk intolerance and parenteral infections in 118 consecutive cases of acute diarrhoea in children. Eur J Pediatr 142: 281-285.

698. Capitanio MA, Greenberg SB (1991) Pneumatosis intestinalis in two infants with rotavirus gastroenteritis. Pediatr Radiol 21: 361-362.

699. Caple IW (1989) Neonatal viral diarrhoeas. Aust Vet J 66: 407-408.

700. Caple J (2006) Pentavalent human-bovine reassortant rotavirus vaccine: a review of its efficacy and safety in preventing acute rotavirus gastroenteritis in healthy infants. Drugs Today (Barc) 42: 313-319.

701. Caprioli A, Falbo V, Giraldi V, Ruggeri FM, Capano G, et al. (1985) Acute childhood diarrhoea in Naples: an aetiologic study. Microbiologica 8: 329-337.

702. Caprioli A, Gentile G, Baldassarri L, Bisicchia R, Romoli E, et al. (1989) Cryptosporidium as a common cause of childhood diarrhoea in Italy. Epidemiol Infect 102: 537-540.

703. Caprioli A, Pezzella C, Morelli R, Giammanco A, Arista S, et al. (1996) Enteropathogens associated with childhood diarrhea in Italy. The Italian Study Group on Gastrointestinal Infections. Pediatr Infect Dis J 15: 876-883.

704. Caracciolo S, Minini C, Colombrita D, Foresti I, Avolio M, et al. (2007) Detection of sporadic cases of Norovirus infection in hospitalized children in Italy. New Microbiol 30: 49-52.

705. Carcamo C, Hooton T, Wener MH, Weiss NS, Gilman R, et al. (2005) Etiologies and manifestations of persistent diarrhea in adults with HIV-1 infection: a case-control study in Lima, Peru. J Infect Dis 191: 11-19.

706. Cardoso das D, Soares CM, Azevedo MS, Leite JP, Munford V, et al. (2000) Serotypes and subgroups of rotavirus isolated from children in central Brazil. J Health Popul Nutr 18: 39-43.

707. Cardoso DD, de Brito WM, Martins RM, Kitajima EW, Souza MP, et al. (1989) [Presence of rotavirus and adenovirus in fecal samples of children with gastroenteritis, in the city of Goyania]. Rev Soc Bras Med Trop 22: 67-71.

708. Cardoso DD, Racz ML, Azevedo MS, Martins RM, Soares CM (2001) Genotyping of group A rotavirus samples from Brazilian children by probe hybridization. Braz J Med Biol Res 34: 471-473.

709. Cardoso DD, Soares CM, Dias e Souza MB, de Azevedo Mda S, Martins RM, et al. (2003) Epidemiological features of rotavirus infection in Goiania, Goias, Brazil, from 1986 to 2000. Mem Inst Oswaldo Cruz 98: 25-29.

710. Cardoso DdD, Martins RM, Kitajima EW, Barbosa AJ, Camarota SC, et al. (1992) [Rotavirus and adenovirus in 0- to 5-year-old children hospitalized with or without gastroenteritis in Goiana, GO, Brazil]. Rev Inst Med Trop Sao Paulo 34: 433-439.

711. Carducci A, Vannucchi R, Guidi M, Reali D, Ruschi MA (1988) Human rotavirus detection in stool specimens using enzyme-linked immunosorbent assays and latex agglutination test. Boll Ist Sieroter Milan 67: 241-244.

712. Carducci A, Verani M, Battistini R, Pizzi F, Rovini E, et al. (2006) Epidemiological surveillance of human enteric viruses by monitoring of different environmental matrices. Water Sci Technol 54: 239-244.

713. Carlin JB, Chondros P, Masendycz P, Bugg H, Bishop RF, et al. (1998) Rotavirus infection and rates of hospitalisation for acute gastroenteritis in young children in Australia, 1993-1996. Med J Aust 169: 252-256.

714. Carlin JB, Jackson T, Lane L, Bishop RF, Barnes GL (1999) Cost effectiveness of rotavirus vaccination in Australia. Aust N Z J Public Health 23: 611-616.

715. Carlson JA, Middleton PJ, Szymanski MT, Huber J, Petric M (1978) Fatal rotavirus gastroenteritis: an analysis of 21 cases. Am J Dis Child 132: 477-479.

716. Carmichael LE, Binn LN (1981) New enteric viruses in the dog. Adv Vet Sci Comp Med 25: 1-37.

717. Carmo EH (2006) Diarrheic disease due to rotavirus: magnitude, introduction of the vaccine, and challenges for epidemiological surveillance. Cad Saude Publica 22: 2266.

718. Carmona RC, Timenetsky Mdo C, da Silva FF, Granato CF (2004) Characterization of rotavirus strains from hospitalized and outpatient children with acute diarrhoea in Sao Paulo, Brazil. J Med Virol 74: 166-172.

719. Carmona RC, Timenetsky Mdo C, Morillo SG, Richtzenhain LJ (2006) Human rotavirus serotype G9, Sao Paulo, Brazil, 1996-2003. Emerg Infect Dis 12: 963-968.

720. Carneiro NB, Diniz-Santos DR, Amorim C, Galeno C, Raposo J, et al. (2006) Septic shock complicating acute rotavirus-associated diarrhea. Pediatr Infect Dis J 25: 571-572.

721. Carneiro NB, Diniz-Santos DR, Fagundes SQ, Neves LL, Reges RM, et al. (2005) Clinical and epidemiological aspects of children hospitalized with severe rotavirus-associated gastroenteritis in Salvador, BA, Brazil. Braz J Infect Dis 9: 525-528.

722. Carr ME, McKendrick GD, Spyridakis T (1976) The clinical features of infantile gastroenteritis due to rotavirus. Scand J Infect Dis 8: 241-243.

723. Carraturo A, Catalani V, Tega L (2008) Microbiological and epidemiological aspects of rotavirus and enteric adenovirus infections in hospitalized children in Italy. New Microbiol 31: 329-336.

724. Carter MJ (2005) Enterically infecting viruses: pathogenicity, transmission and significance for food and waterborne infection. J Appl Microbiol 98: 1354-1380.

725. Cartun RW, Van Kruiningen HJ, Pedersen CA, Berman MM (1993) An immunocytochemical search for infectious agents in Crohn's disease. Mod Pathol 6: 212-219.

726. Cartwright-Shamoon J, Dickson GR, Dodge J, Carr KE (1996) Uptake of yeast (Saccharomyces boulardii) in normal and rotavirus treated intestine. Gut 39: 204-209.

727. Carvalho-Costa FA, Assis RM, Fialho AM, Boia MN, Alves DP, et al. (2006) Detection and molecular characterization of group A rotavirus from hospitalized children in Rio de Janeiro, Brazil, 2004. Mem Inst Oswaldo Cruz 101: 291-294.

728. Casadei BM, Ciabatti AM, Ignesti C, Livatino L (1987) [Incidence of rotavirus in pediatric patients hospitalized with gastroenteric symptoms]. Quad Sclavo Diagn 23: 246-250.

729. Casalino M, Yusuf MW, Nicoletti M, Bazzicalupo P, Coppo A, et al. (1988) A two-year study of enteric infections associated with diarrhoeal diseases in children in urban Somalia. Trans R Soc Trop Med Hyg 82: 637-641.

730. Cascio A, Vizzi E, Alaimo C, Arista S (2001) Rotavirus gastroenteritis in Italian children: can severity of symptoms be related to the infecting virus? Clin Infect Dis 32: 1126-1132.

731. Cash P, Freebain E, Brown T, Reid TM (1986) Molecular epidemiology of human rotavirus. J Hyg (Lond) 96: 265-275.

732. Casini T, Cristiano R, Salvi G, Franchini F, Calabri G, et al. (1997) [Evaluation of pediatric patients hospitalized for acute diarrhea from 1990 to 1996]. Pediatr Med Chir 19: 31-35.

733. Casola A, Estes MK, Crawford SE, Ogra PL, Ernst PB, et al. (1998) Rotavirus infection of cultured intestinal epithelial cells induces secretion of CXC and CC chemokines. Gastroenterology 114: 947-955.

734. Casola A, Garofalo RP, Crawford SE, Estes MK, Mercurio F, et al. (2002) Interleukin-8 gene regulation in intestinal epithelial cells infected with rotavirus: role of viral-induced IkappaB kinase activation. Virology 298: 8-19.

735. Cassel-Beraud AM, Michel P, Garbarg-Chenon A (1993) Epidemiological study of infantile rotavirus diarrhoea in Tananarive (Madagascar). J Diarrhoeal Dis Res 11: 82-87.

736. Cassel-Beraud AM, Morvan J, Rakotoarimanana DR, Razanamparany M, Candito D, et al. (1990) [Infantile diarrheal diseases in Madagascar: bacterial, parasitologic and viral study]. Arch Inst Pasteur Madagascar 57: 223-254.

737. Casswall TH, Sarker SA, Faruque SM, Weintraub A, Albert MJ, et al. (2000) Treatment of enterotoxigenic and enteropathogenic Escherichia coli-induced diarrhoea in children with bovine immunoglobulin milk concentrate from hyperimmunized cows: a double-blind, placebo-controlled, clinical trial. Scand J Gastroenterol 35: 711-718.

738. Castello AA, Arguelles MH, Rota RP, Olthoff A, Jiang B, et al. (2006) Molecular epidemiology of group A rotavirus diarrhea among children in Buenos Aires, Argentina, from 1999 to 2003 and emergence of the infrequent genotype G12. J Clin Microbiol 44: 2046-2050.

739. Castello AA, Arguelles MH, Villegas GA, Lopez N, Ghiringhelli DP, et al. (2000) Characterization of human group C rotavirus in Argentina. J Med Virol 62: 199-207.

740. Castello AA, Arguelles MH, Villegas GA, Olthoff A, Glikmann G (2002) Incidence and prevalence of human group C rotavirus infections in Argentina. J Med Virol 67: 106-112.

741. Castilho JG, Botelho MV, Lauretti F, Taniwaki N, Linhares RE, et al. (2004) The in vitro cytopathology of a porcine and the simian (SA-11) strains of rotavirus. Mem Inst Oswaldo Cruz 99: 313-317.

742. Castrucci G, Ferrari M, Angelillo V, Rigonat F, Capodicasa L (1993) Field evaluation of the efficacy of Romovac 50, a new inactivated, adjuvanted bovine rotavirus vaccine. Comp Immunol Microbiol Infect Dis 16: 235-239.

743. Castrucci G, Ferrari M, Frigeri F, Cilli V, Caleffi F, et al. (1983) Experimental infection and cross protection tests in calves with cytopathic strains of bovine rotavirus. Comp Immunol Microbiol Infect Dis 6: 321-332.

744. Castrucci G, Ferrari M, Frigeri F, Cilli V, Donelli G, et al. (1983) A study of cytopathic rotavirus strains isolated from calves with acute enteritis. Comp Immunol Microbiol Infect Dis 6: 253-264.

745. Castrucci G, Ferrari M, Frigeri F, Cilli V, Perucca L, et al. (1985) Isolation and characterization of cytopathic strains of rotavirus from rabbits. Brief report. Arch Virol 83: 99-104.

746. Castrucci G, Ferrari M, Frigeri F, Traldi V, Angelillo V (1994) A study on neonatal calf diarrhea induced by rotavirus. Comp Immunol Microbiol Infect Dis 17: 321-331.

747. Castrucci G, Frigeri F, Angelillo V, Ferrari M, Cilli V, et al. (1987) Field trial evaluation of an inactivated rotavirus vaccine against neonatal diarrhea of calves. Eur J Epidemiol 3: 5-9.

748. Castrucci G, Frigeri F, Ferrari M, Aldrovandi V, Angelillo V, et al. (1989) Immunization against bovine rotaviral infection. Eur J Epidemiol 5: 279-284.

749. Castrucci G, Frigeri F, Ferrari M, Aldrovandi V, Tassini F (1989) Further studies on passive immunization of newborn calves against rotaviral infection. Comp Immunol Microbiol Infect Dis 12: 71-76.

750. Castrucci G, Frigeri F, Ferrari M, Aldrovandi V, Tassini F, et al. (1988) The protection of newborn calves against experimental rotavirus infection by feeding mammary secretions from vaccinated cows. Microbiologica 11: 379-385.

751. Castrucci G, Frigeri F, Ferrari M, Cilli V, Caleffi F, et al. (1984) The efficacy of colostrum from cows vaccinated with rotavirus in protecting calves to experimentally induced rotavirus infection. Comp Immunol Microbiol Infect Dis 7: 11-18.

752. Castrucci G, Frigeri F, Ferrari M, Cilli V, Gualandi GL, et al. (1988) Neonatal calf diarrhea induced by rotavirus. Comp Immunol Microbiol Infect Dis 11: 71-84.

753. Cataloluk O, Iturriza M, Gray J (2005) Molecular characterization of rotaviruses circulating in the population in Turkey. Epidemiol Infect 133: 673-678.

754. Catto-Smith AG, Emselle S, Bishop RF (2008) Changes in macromolecular transport appear early in Caco-2 cells infected with a human rotavirus. Scand J Gastroenterol 43: 314-322.

755. Caul EO, Ashley CR, Darville JM, Bridger JC (1990) Group C rotavirus associated with fatal enteritis in a family outbreak. J Med Virol 30: 201-205.

756. Cavallo JD, Garrabe E (2007) [Infectious aetiologies of travelers' diarrhoea.]. Med Mal Infect 37: 722-727.

757. Cavazza ME, Rodriguez Lemoine V (1994) Detection of microcin production by pathogenic Escherichia coli isolates from children with acute diarrhoea. Acta Cient Venez 45: 106-111.

758. Cebra CK, Mattson DE, Baker RJ, Sonn RJ, Dearing PL (2003) Potential pathogens in feces from unweaned llamas and alpacas with diarrhea. J Am Vet Med Assoc 223: 1806-1808.

759. Cegielski JP, Msengi AE, Miller SE (1994) Enteric viruses associated with HIV infection in Tanzanian children with chronic diarrhea. Pediatr AIDS HIV Infect 5: 296-299.

760. Cevenini R, Mazzaracchio R, Rumpianesi F, Donati M, Moroni A, et al. (1987) Prevalence of enteric adenovirus from acute gastroenteritis: a five year study. Eur J Epidemiol 3: 147-150.

761. Cevenini R, Rumpianesi F, Mazzaracchio R, Donati M, Falcieri E, et al. (1983) Evaluation of a new latex agglutination test for detecting human rotavirus in faeces. J Infect 7: 130-133.

762. Cevenini R, Rumpianesi F, Mazzaracchio R, Donati M, Falcieri E, et al. (1984) A simple immunoperoxidase method for detecting enteric adenovirus and rotavirus in cell culture. J Infect 8: 22-27.

763. Cevenini R, Varoli O, Rumpianesi F, Mazzaracchio R, Nanetti A, et al. (1985) A two-year longitudinal study on the etiology of acute diarrhea in young children in Northern Italy. Microbiologica 8: 51-58.

764. Ceyhan M, Kanra G, Yeniay I, Ciliv G, Vesikari T (1987) Rotaviruses in infants with diarrhea studied by viral RNA electrophoresis in Ankara, Turkey. Turk J Pediatr 29: 145-149.

765. Cezard JP, Bellaiche M, Viala J, Hugot JP (2007) [Medication in infectious acute diarrhea in children]. Arch Pediatr 14 Suppl 3: S169-175.

766. Cezard JP, Duhamel JF, Meyer M, Pharaon I, Bellaiche M, et al. (2001) Efficacy and tolerability of racecadotril in acute diarrhea in children. Gastroenterology 120: 799-805.

767. Chaibi C, Cotte-Laffitte J, Sandre C, Esclatine A, Servin AL, et al. (2005) Rotavirus induces apoptosis in fully differentiated human intestinal Caco-2 cells. Virology 332: 480-490.

768. Chakladar A, Chakrabarti S (1998) Nucleotide sequence of the VP7 gene of human rotavirus isolated in Calcutta, India: possible emergence of a new subtype of serotype I. Intervirology 41: 127-131.

769. Chakravarti A, Broor S, Natarajan R, Setty VS, Mittal SK (1992) Epidemiological and clinical characteristics of acute diarrhoea in children due to human rotavirus. J Trop Pediatr 38: 192-193.

770. Chakravarti A, Kumar S, Mittal SK, Broor S (1991) Comparison of latex agglutination and polyacrylamide gel electrophoresis with enzyme linked immunosorbent assay for detecting human rotavirus in stool specimens. Indian Pediatr 28: 507-510.

771. Chakravarti A, Kumar S, Mittal SK, Broor S (1992) Clinical and epidemiological features of acute gastroenteritis caused by human rotavirus subgroups. J Diarrhoeal Dis Res 10: 21-24.

772. Chakravarti A, Kumaria R, Chakravarti A (2005) Prevalence of genotypes G1-G4 of human rotavirus in a hospital setting in New Delhi. Indian J Gastroenterol 24: 127-128.

773. Chakravarti A, Rawat D, Chakravarti A (2004) Molecular epidemiology of rotavirus in Delhi. Indian J Pathol Microbiol 47: 90-93.

774. Champsaur H, Assathiany R, Pincet J, Bach C (1979) [Rotavirus gastro-enteritis in infants (author's transl)]. Sem Hop 55: 1026-1030.

775. Champsaur H, Assathiany R, Pincet J, Bach C (1979) [Rotavirus gastro-enteritis in infants]. Ann Pediatr (Paris) 26: 26-30.

776. Champsaur H, Henry-Amar M, Goldszmidt D, Prevot J, Bourjouane M, et al. (1984) Rotavirus carriage, asymptomatic infection, and disease in the first two years of life. II. Serological response. J Infect Dis 149: 675-682.

777. Champsaur H, Questiaux E, Prevot J, Henry-Amar M, Goldszmidt D, et al. (1984) Rotavirus carriage, asymptomatic infection, and disease in the first two years of life. I. Virus shedding. J Infect Dis 149: 667-674.

778. Chan MC, Sung JJ, Lam RK, Chan PK, Lai RW, et al. (2006) Sapovirus detection by quantitative real-time RT-PCR in clinical stool specimens. J Virol Methods 134: 146-153.

779. Chan PK, Tam JS, Nelson EA, Fung KS, Adeyemi-Doro FA, et al. (1998) Rotavirus infection in Hong Kong: epidemiology and estimates of disease burden. Epidemiol Infect 120: 321-325.

780. Chan RC, Tam JS, Fok TF, French GL (1989) RNA-electrophoresis as a typing method for nosocomial rotavirus infection in a special-care baby unit. J Hosp Infect 13: 367-375.

781. Chan RY, Tan CE, Czech-Schmidt G, Petersen C (2005) Computerized three-dimensional study of a rotavirus model of biliary atresia: comparison with human biliary atresia. Pediatr Surg Int 21: 615-620.

782. Chandan RC (1999) Enhancing market value of milk by adding cultures. J Dairy Sci 82: 2245-2256.

783. Chandran A, Santosham M (2008) RotaTeq: a three-dose oral pentavalent reassortant rotavirus vaccine. Expert Rev Vaccines 7: 1475-1480.

784. Chang EJ, Zangwill KM, Lee H, Ward JI (2002) Lack of association between rotavirus infection and intussusception: implications for use of attenuated rotavirus vaccines. Pediatr Infect Dis J 21: 97-102.

785. Chang HG, Glass RI, Smith PF, Cicirello HG, Holman RC, et al. (2003) Disease burden and risk factors for hospitalizations associated with rotavirus infection among children in New York State, 1989 through 2000. Pediatr Infect Dis J 22: 808-814.

786. Chang HG, Smith PF, Ackelsberg J, Morse DL, Glass RI (2001) Intussusception, rotavirus diarrhea, and rotavirus vaccine use among children in New York state. Pediatrics 108: 54-60.

787. Chang KO, Kim YJ, Saif LJ (1999) Comparisons of nucleotide and deduced amino acid sequences of NSP4 genes of virulent and attenuated pairs of group A and C rotaviruses. Virus Genes 18: 229-233.

788. Chang KO, Nielsen PR, Ward LA, Saif LJ (1999) Dual infection of gnotobiotic calves with bovine strains of group A and porcine-like group C rotaviruses influences pathogenesis of the group C rotavirus. J Virol 73: 9284-9293.

789. Chang KO, Parwani AV, Saif LJ (1995) Comparative nucleotide and deduced amino acid sequence analysis of VP7 gene of the NCDV Cody (I-801) strain of group A bovine rotavirus. Arch Virol 140: 1279-1283.

790. Chang KO, Parwani AV, Smith D, Saif LJ (1997) Detection of group B rotaviruses in fecal samples from diarrheic calves and adult cows and characterization of their VP7 genes. J Clin Microbiol 35: 2107-2110.

791. Chang KO, Vandal OH, Yuan L, Hodgins DC, Saif LJ (2001) Antibody-secreting cell responses to rotavirus proteins in gnotobiotic pigs inoculated with attenuated or virulent human rotavirus. J Clin Microbiol 39: 2807-2813.

792. Chang MN, Guess HA, Heyse JF (1994) Reduction in burden of illness: a new efficacy measure for prevention trials. Stat Med 13: 1807-1814.

793. Chanock RM, Wyatt RG, Kapikian AZ (1978) Immunization of infants and young children against rotaviral gastroenteritis--prospects and problems. J Am Vet Med Assoc 173: 570-572.

794. Chapin M, Yatabe J, Cherry JD (1983) An outbreak of rotavirus gastroenteritis on a pediatric unit. Am J Infect Control 11: 88-91.

795. Charles MD, Holman RC, Curns AT, Parashar UD, Glass RI, et al. (2006) Hospitalizations associated with rotavirus gastroenteritis in the United States, 1993-2002. Pediatr Infect Dis J 25: 489-493.

796. Chasey D, Banks J (1984) The commonest rotaviruses from neonatal lamb diarrhoea in England and Wales have atypical electropherotypes. Vet Rec 115: 326-327.

797. Chasey D, Banks J (1986) Replication of atypical ovine rotavirus in small intestine and cell culture. J Gen Virol 67 ( Pt 3): 567-576.

798. Chasey D, Bridger JC, McCrae MA (1986) A new type of atypical rotavirus in pigs. Arch Virol 89: 235-243.

799. Chasey D, Davies P (1984) Atypical rotaviruses in pigs and cattle. Vet Rec 114: 16-17.

800. Chasey D, Higgins RJ, Jeffrey M, Banks J (1989) Atypical rotavirus and villous epithelial cell syncytia in piglets. J Comp Pathol 100: 217-222.

801. Chatterjee B, Husain M, Kavita, Seth P, Broor S (1996) Diversity of rotavirus strains infecting pediatric patients in New Delhi, India. J Trop Pediatr 42: 207-210.

802. Chatterjee NK, Moore DW, Monroe SS, Glass RI, Cambridge MJ, et al. (2004) Molecular epidemiology of outbreaks of viral gastroenteritis in New York State, 1998-1999. Clin Infect Dis 38 Suppl 3: S303-310.

803. Chauhan RS, Singh NP (1992) Cell-mediated immune response in rotavirus-infected calves: leucocyte migration inhibition assay. J Comp Pathol 107: 115-118.

804. Chauhan RS, Singh NP (1992) Rapid diagnosis of rotavirus infection in calves by dot immunobinding assay. Vet Rec 130: 381.

805. Chege GK, Steele AD, Hart CA, Snodgrass DR, Omolo EO, et al. (2005) Experimental infection of non-human primates with a human rotavirus isolate. Vaccine 23: 1522-1528.

806. Chemaly RF, Yen-Lieberman B, Schindler SA, Goldfarb J, Hall GS, et al. (2003) Rotaviral and bacterial gastroenteritis in children during winter: an evaluation of physician ordering patterns. J Clin Virol 28: 44-50.

807. Chen CM, Hung T, Bridger JC, McCrae MA (1985) Chinese adult rotavirus is a group B rotavirus. Lancet 2: 1123-1124.

808. Chen DM, Qian Y, Zhang Y, Chang RX (2003) [Sequence analysis of VP7 gene from rotavirus field strain from Guangzhou, China]. Zhonghua Shi Yan He Lin Chuang Bing Du Xue Za Zhi 17: 165-168.

809. Chen GM, Hung T, Mackow ER (1990) Identification of the gene encoding the group B rotavirus VP7 equivalent: primary characterization of the ADRV segment 9 RNA. Virology 178: 311-315.

810. Chen GM, Werner-Eckert R, Tao H, Mackow ER (1991) Expression of the major inner capsid protein of the group B rotavirus ADRV: primary characterization of genome segment 5. Virology 182: 820-829.

811. Chen HJ, Chen BS, Wang SF, Lai MH (1991) Rotavirus gastroenteritis in children: a clinical study of 125 patients in Hsin-Tien area. Zhonghua Min Guo Xiao Er Ke Yi Xue Hui Za Zhi 32: 73-78.

812. Chen HN, Dennehy PH, Oh W, Lee CN, Huang ML, et al. (1997) Outbreak and control of a rotaviral infection in a nursery. J Formos Med Assoc 96: 884-889.

813. Chen KT, Chen PY, Tang RB, Huang YF, Lee PI, et al. (2005) Sentinel hospital surveillance for rotavirus diarrhea in Taiwan, 2001-2003. J Infect Dis 192 Suppl 1: S44-48.

814. Chen KT, Fan SF, Tang RB, Huang YF, Lee PI, et al. (2007) Hospital-based study of the economic burden associated with rotavirus diarrhea in Taiwan. Vaccine 25: 4266-4272.

815. Chen MF, Gao Y, Cong X, Sun CL, Zhu JY, et al. (2008) [Etiological study on sporadic viral gastroenteritis among adult in Beijing]. Zhonghua Yi Xue Za Zhi 88: 265-267.

816. Chen MF, Gao Y, Jia LP, Zhang Y, Qian Y, et al. (2007) [A study on viral gastroenteritis attributed to noroviruses in hospitals]. Zhonghua Liu Xing Bing Xue Za Zhi 28: 141-143.

817. Chen SC, Fynan EF, Robinson HL, Lu S, Greenberg HB, et al. (1997) Protective immunity induced by rotavirus DNA vaccines. Vaccine 15: 899-902.

818. Chen SD, Xie XL, Du BN, Su QH, Wei QD, et al. (1984) Infantile rotavirus enteritis treated with herbal Valeriana jatamansi (VJ). J Tradit Chin Med 4: 297-300.

819. Chen SM, Ni YH, Chen HL, Chang MH (2006) Microbial etiology of acute gastroenteritis in hospitalized children in Taiwan. J Formos Med Assoc 105: 964-970.

820. Chen SY, Chang YC, Lee YS, Chao HC, Tsao KC, et al. (2007) Molecular epidemiology and clinical manifestations of viral gastroenteritis in hospitalized pediatric patients in Northern Taiwan. J Clin Microbiol 45: 2054-2057.

821. Chen SY, Tsai CN, Chao HC, Lai MW, Lin TY, et al. (2008) Acute gastroenteritis caused by multiple enteric pathogens in children. Epidemiol Infect: 1-4.

822. Chen WK, Campbell T, VanCott J, Saif LJ (1995) Enumeration of isotype-specific antibody-secreting cells derived from gnotobiotic piglets inoculated with porcine rotaviruses. Vet Immunol Immunopathol 45: 265-284.

823. Chen Y, Wen Y, Liu X, Xiong X, Cao Z, et al. (2008) Full genomic analysis of human rotavirus strain TB-Chen isolated in China. Virology 375: 361-373.

824. Chen Y, Zhao J, Yan L (1999) [Comparison of three methods in detection of rotavirus infection in neonates]. Zhonghua Shi Yan He Lin Chuang Bing Du Xue Za Zhi 13: 180-182.

825. Chen YE, Beasley S, Grimwood K (2005) Intussusception and rotavirus associated hospitalisation in New Zealand. Arch Dis Child 90: 1077-1081.

826. Chen ZD, Shen XS, Lu H (1996) [Study of etiology on inpatients with diarrhea in Suixian county of east Henan]. Zhonghua Liu Xing Bing Xue Za Zhi 17: 272-274.

827. Cheng AC, McDonald JR, Thielman NM (2005) Infectious diarrhea in developed and developing countries. J Clin Gastroenterol 39: 757-773.

828. Cheng WX, Jin Y, Duan ZJ, Xu ZQ, Qi HM, et al. (2008) Human bocavirus in children hospitalized for acute gastroenteritis: a case-control study. Clin Infect Dis 47: 161-167.

829. Cherkasova LV, Tibekin AT, Zaitsev BE, Berglezova LN, Solodovnikov Iu P, et al. (2004) [Etiological structure of enteric infection outbreaks in Moscow in 1993-2002]. Zh Mikrobiol Epidemiol Immunobiol: 102-105.

830. Chermesh I, Eliakim R (2006) Probiotics and the gastrointestinal tract: where are we in 2005? World J Gastroenterol 12: 853-857.

831. Chernesky M, Castriciano S, Mahony J, DeLong D (1985) Examination of the Rotazyme II enzyme immunoassay for the diagnosis of rotavirus gastroenteritis. J Clin Microbiol 22: 462-464.

832. Chernesky M, Castriciano S, Mahony J, Spiewak M, Schaefer L (1988) Ability of TESTPACK ROTAVIRUS enzyme immunoassay to diagnose rotavirus gastroenteritis. J Clin Microbiol 26: 2459-2461.

833. Cheung EY, Hnatko SI, Gunning H, Wilson J (1982) Comparison of Rotazyme and direct electron microscopy for detection of rotavirus in human stools. J Clin Microbiol 16: 562-563.

834. Chhabra P, Chitambar SD (2008) Norovirus genotype IIb associated acute gastroenteritis in India. J Clin Virol 42: 429-432.

835. Chhin S, Harwell JI, Bell JD, Rozycki G, Ellman T, et al. (2006) Etiology of chronic diarrhea in antiretroviral-naive patients with HIV infection admitted to Norodom Sihanouk Hospital, Phnom Penh, Cambodia. Clin Infect Dis 43: 925-932.

836. Chi H, Sun W, Chan WT, Lee HC, Fang SB (2001) Pediatric Salmonella enterocolitis in a teaching hospital in Taitung: A four-year analysis. Acta Paediatr Taiwan 42: 297-300.

837. Chiappini E, Azzari C, Moriondo M, Galli L, de Martino M (2005) Viraemia is a common finding in immunocompetent children with rotavirus infection. J Med Virol 76: 265-267.

838. Chiba S, Akihara M, Kogasaka R, Horino K, Nakao T, et al. (1979) An out-break of acute gastroenteritis due to rotavirus in an infant home. Tohoku J Exp Med 127: 265-271.

839. Chiba S, Kogasaka R, Akihara M, Horino K, Nakao T (1979) Recurrent attack of rotavirus gastroenteritis after adenovirus-induced diarrhoea. Arch Dis Child 54: 398-400.

840. Chiba S, Nakata S, Ukae S, Adachi N (1993) Virological and serological aspects of immune resistance to rotavirus gastroenteritis. Clin Infect Dis 16 Suppl 2: S117-121.

841. Chiba S, Yokoyama T, Nakata S, Morita Y, Urasawa T, et al. (1986) Protective effect of naturally acquired homotypic and heterotypic rotavirus antibodies. Lancet 2: 417-421.

842. Chiba Y, Miyazaki C, Makino Y, Mutanda LN, Kibue A, et al. (1984) Rotavirus infection of young children in two districts of Kenya from 1982 to 1983 as analyzed by electrophoresis of genomic RNA. J Clin Microbiol 19: 579-582.

843. Chikhi-Brachet R, Bon F, Toubiana L, Pothier P, Nicolas JC, et al. (2002) Virus diversity in a winter epidemic of acute diarrhea in France. J Clin Microbiol 40: 4266-4272.

844. Chilarski A (1981) [Current theories on the etiology of Crohn disease]. Pediatr Pol 56: 699-703.

845. Chimura Y, Annaka M, Shibazaki S, Adachi K, Shinkai T, et al. (2002) [An epidemic of rotavirus infection in a nursing home for the elderly in Japan]. Kansenshogaku Zasshi 76: 450-454.

846. Chinsangaram J, Schore CE, Guterbock W, Weaver LD, Osburn BI (1995) Prevalence of group A and group B rotaviruses in the feces of neonatal dairy calves from California. Comp Immunol Microbiol Infect Dis 18: 93-103.

847. Chippaux-Hyppolite C, Loukou Yao G, Chippaux A (1991) [Rotavirus infections and their prevention with vaccines in children]. Bull Soc Pathol Exot 84: 918-925.

848. Chitambar SD, Tatte VS, Dhongde R, Kalrao V (2008) High frequency of rotavirus viremia in children with acute gastroenteritis: discordance of strains detected in stool and sera. J Med Virol 80: 2169-2176.

849. Chiu TF, Lee CN, Lee PI, Kao CL, Lin HC, et al. (2000) Rotavirus gastroenteritis in children: 5-year experience in a medical center. J Microbiol Immunol Infect 33: 181-186.

850. Cho KO, Hasoksuz M, Nielsen PR, Chang KO, Lathrop S, et al. (2001) Cross-protection studies between respiratory and calf diarrhea and winter dysentery coronavirus strains in calves and RT-PCR and nested PCR for their detection. Arch Virol 146: 2401-2419.

851. Choi AH, McNeal MM, Flint JA, Basu M, Lycke NY, et al. (2002) The level of protection against rotavirus shedding in mice following immunization with a chimeric VP6 protein is dependent on the route and the coadministered adjuvant. Vaccine 20: 1733-1740.

852. Choi NW, Estes MK, Langridge WH (2005) Oral immunization with a shiga toxin B subunit: rotavirus NSP4(90) fusion protein protects mice against gastroenteritis. Vaccine 23: 5168-5176.

853. Choi SW, Park CH, Silva TM, Zaenker EI, Guerrant RL (1996) To culture or not to culture: fecal lactoferrin screening for inflammatory bacterial diarrhea. J Clin Microbiol 34: 928-932.

854. Chou IC, Tsai CH, Tsai FJ (1998) Rotavirus associated with poliomyelitis-like syndrome. Pediatr Infect Dis J 17: 930-931.

855. Chouikha A, Fodha I, Noomen S, Bouzid L, Mastouri M, et al. (2007) Group A rotavirus strains circulating in the eastern center of Tunisia during a ten-year period (1995-2004). J Med Virol 79: 1002-1008.

856. Chowdhury F, Khan AI, Hossain MI, Malek MA, Faruque AS (2005) Presence of neutral fat in stool and its association with aetiology and presenting features of diarrhoea in children. Trop Gastroenterol 26: 80-84.

857. Chowdhury HR, Yunus M, Zaman K, Rahman A, Faruque SM, et al. (2001) The efficacy of bismuth subsalicylate in the treatment of acute diarrhoea and the prevention of persistent diarrhoea. Acta Paediatr 90: 605-610.

858. Christensen ML (1989) Human viral gastroenteritis. Clin Microbiol Rev 2: 51-89.

859. Christie CD, Duncan ND, Thame KT, Smith HD (2006) New rotavirus vaccines for infant gastroenteritis arriving soon. West Indian Med J 55: 1-3.

860. Christy C, Offit P, Clark HF, Treanar J (1993) Evaluation of a bovine-human rotavirus reassortant vaccine in infants. J Infect Dis 168: 1598-1599.

861. Christy C, Vosefski D, Madore HP (1990) Comparison of three enzyme immunoassays to tissue culture for the diagnosis of rotavirus gastroenteritis in infants and young children. J Clin Microbiol 28: 1428-1430.

862. Chrystie IL, Totterdell B, Baker MJ, Scopes JW, Banatvala JE (1975) Letter: Rotavirus infections in a maternity unit. Lancet 2: 79.

863. Chrystie IL, Totterdell BM, Banatvala JE (1978) Asymptomatic endemic rotavirus infections in the newborn. Lancet 1: 1176-1178.

864. Chudzio T, Kasatiya S, Irvine N, Sankar-Mistry P (1989) Rapid screening test for the diagnosis of rotavirus infection. J Clin Microbiol 27: 2394-2396.

865. Chueh LL, Chu RM, Li WI, Chang WF (1982) Isolation and identification of swine rotavirus in Taiwan. Zhonghua Min Guo Wei Sheng Wu Ji Mian Yi Xue Za Zhi 15: 212-220.

866. Chunge RN, Simwa JM, Karumba PN, Kenya PR, Kinoti SN, et al. (1992) Comparative aetiology of childhood diarrhoea in Kakamega and Kiambu Districts, Kenya. East Afr Med J 69: 437-441.

867. Chunge RN, Wamola IA, Kinoti SN, Muttunga J, Mutanda LN, et al. (1989) Mixed infections in childhood diarrhoea: results of a community study in Kiambu District, Kenya. East Afr Med J 66: 715-723.

868. Chyou SC, Leu YJ, Huang FY, Lee HC, Yang DI (1988) An etiological study of infectious diarrhea in infants and children in Taipei area. Zhonghua Min Guo Xiao Er Ke Yi Xue Hui Za Zhi 29: 213-220.

869. Ciarlet M, Conner ME, Finegold MJ, Estes MK (2002) Group A rotavirus infection and age-dependent diarrheal disease in rats: a new animal model to study the pathophysiology of rotavirus infection. J Virol 76: 41-57.

870. Ciarlet M, Estes MK (2001) Interactions between rotavirus and gastrointestinal cells. Curr Opin Microbiol 4: 435-441.

871. Ciarlet M, Estes MK (2001) Rotavirus and calicivirus infections of the gastrointestinal tract. Curr Opin Gastroenterol 17: 10-16.

872. Ciarlet M, Gilger MA, Barone C, McArthur M, Estes MK, et al. (1998) Rotavirus disease, but not infection and development of intestinal histopathological lesions, is age restricted in rabbits. Virology 251: 343-360.

873. Ciarlet M, Heaton PM (2007) The Pentavalent Rotavirus Vaccine: Discovery to Licensure and Beyond. Clin Infect Dis 45.

874. Ciarlet M, Hidalgo M, Gorziglia M, Liprandi F (1994) Characterization of neutralization epitopes on the VP7 surface protein of serotype G11 porcine rotaviruses. J Gen Virol 75 ( Pt 8): 1867-1873.

875. Ciarlet M, Hoffmann C, Lorusso E, Baselga R, Cafiero MA, et al. (2008) Genomic characterization of a novel group A lamb rotavirus isolated in Zaragoza, Spain. Virus Genes 37: 250-265.

876. Ciarlet M, Hyser JM, Estes MK (2002) Sequence analysis of the VP4, VP6, VP7, and NSP4 gene products of the bovine rotavirus WC3. Virus Genes 24: 107-118.

877. Ciarlet M, P Ia, Conner ME, Liprandi F (2001) Antigenic and molecular analyses reveal that the equine rotavirus strain H-1 is closely related to porcine, but not equine, rotaviruses: interspecies transmission from pigs to horses? Virus Genes 22: 5-20.

878. Ciarlet M, Pina CI, Garcia O, Liprandi F (1997) Identification of bovine rotaviruses in Venezuela: antigenic and molecular characterization of a bovine rotavirus strain. Res Virol 148: 289-297.

879. Ciarlet M, Reggeti F, Pina CI, Liprandi F (1994) Equine rotaviruses with G14 serotype specificity circulate among venezuelan horses. J Clin Microbiol 32: 2609-2612.

880. Ciarlet M, Sani-Grosso R, Yuan G, Liu GF, Heaton PM, et al. (2008) Concomitant use of the oral pentavalent human-bovine reassortant rotavirus vaccine and oral poliovirus vaccine. Pediatr Infect Dis J 27: 874-880.

881. Cicchetti R, Iacobini M, Midulla F, Papoff P, Mancuso M, et al. (2006) Pantoea agglomerans sepsis after rotavirus gastroenteritis. Pediatr Infect Dis J 25: 280-281.

882. Cicek C, Karatas T, Altuglu I, Koturoglu G, Kurugol Z, et al. (2007) Comparison of ELISA with shell vial cell culture method for the detection of human rotavirus in fecal specimens. New Microbiol 30: 113-118.

883. Cicirello HG, Das BK, Gupta A, Bhan MK, Gentsch JR, et al. (1994) High prevalence of rotavirus infection among neonates born at hospitals in Delhi, India: predisposition of newborns for infection with unusual rotavirus. Pediatr Infect Dis J 13: 720-724.

884. Cilla G, Perez-Trallero E, Lopez-Lopategui MC, Gilsetas A, Gomariz M (2000) Incidence, seasonality and serotypes of rotavirus in Gipuzkoa (Basque Country), Spain. A 14-year study. Epidemiol Infect 125: 677-683.

885. Cilla G, Perez-Trallero GE, Pineiro LD, Iturzaeta A, Vicente D (1999) Hospitalizations for rotavirus gastroenteritis in Gipuzkoa (Basque country), Spain. Emerg Infect Dis 5: 834-835.

886. Cilli V, Castrucci G (1981) Viral diarrhea of young animals: a review. Comp Immunol Microbiol Infect Dis 4: 229-242.

887. Cisse MF, Le Guenno B, Gaye A, Boye CS, Sourabie S, et al. (1988) [Electrophoretic types of RNA from human rotaviruses isolated from a population of children in Dakar (Senegal)]. Dakar Med 33: 56-59.

888. Cisse MF, Leguenno B, Sourabie S, Gaye A, Boye CS, et al. (1989) [Electrophoretypes of the genome of rotaviruses isolated from Senegalese children]. Bull Soc Pathol Exot Filiales 82: 637-644.

889. Cisse MF, Ouangre RA, Gaye A, Boye CS, Sow AI, et al. (1989) [Causes of infectious gastro-enteritis in children in Dakar]. Presse Med 18: 1827-1830.

890. Clark B, McKendrick M (2004) A review of viral gastroenteritis. Curr Opin Infect Dis 17: 461-469.

891. Clark HF, Bernstein DI, Dennehy PH, Offit P, Pichichero M, et al. (2004) Safety, efficacy, and immunogenicity of a live, quadrivalent human-bovine reassortant rotavirus vaccine in healthy infants. J Pediatr 144: 184-190.

892. Clark HF, Borian FE, Bell LM, Modesto K, Gouvea V, et al. (1988) Protective effect of WC3 vaccine against rotavirus diarrhea in infants during a predominantly serotype 1 rotavirus season. J Infect Dis 158: 570-587.

893. Clark HF, Borian FE, Plotkin SA (1990) Immune protection of infants against rotavirus gastroenteritis by a serotype 1 reassortant of bovine rotavirus WC3. J Infect Dis 161: 1099-1104.

894. Clark HF, Burke CJ, Volkin DB, Offit P, Ward RL, et al. (2003) Safety, immunogenicity and efficacy in healthy infants of G1 and G2 human reassortant rotavirus vaccine in a new stabilizer/buffer liquid formulation. Pediatr Infect Dis J 22: 914-920.

895. Clark HF, Dolan KT, Horton-Slight P, Palmer J, Plotkin SA (1985) Diverse serologic response to rotavirus infection of infants in a single epidemic. Pediatr Infect Dis 4: 626-631.

896. Clark HF, Hoshino Y, Bell LM, Groff J, Hess G, et al. (1987) Rotavirus isolate WI61 representing a presumptive new human serotype. J Clin Microbiol 25: 1757-1762.

897. Clark HF, Lawley D, Shrager D, Jean-Guillaume D, Offit PA, et al. (2004) Infant immune response to human rotavirus serotype G1 vaccine candidate reassortant WI79-9: different dose response patterns to virus surface proteins VP7 and VP4. Pediatr Infect Dis J 23: 206-211.

898. Clark HF, Lawley DA, Schaffer A, Patacsil JM, Marcello AE, et al. (2004) Assessment of the epidemic potential of a new strain of rotavirus associated with the novel G9 serotype which caused an outbreak in the United States for the first time in the 1995-1996 season. J Clin Microbiol 42: 1434-1438.

899. Clark HF, Offit PA (2004) Vaccines for rotavirus gastroenteritis universally needed for infants. Pediatr Ann 33: 536-543.

900. Clark HF, Offit PA, Dolan KT, Tezza A, Gogalin K, et al. (1986) Response of adult human volunteers to oral administration of bovine and bovine/human reassortant rotaviruses. Vaccine 4: 25-31.

901. Clark HF, Offit PA, Ellis RW, Eiden JJ, Krah D, et al. (1996) The development of multivalent bovine rotavirus (strain WC3) reassortant vaccine for infants. J Infect Dis 174 Suppl 1: S73-80.

902. Clark HF, Offit PA, Plotkin SA, Heaton PM (2006) The new pentavalent rotavirus vaccine composed of bovine (strain WC3) -human rotavirus reassortants. Pediatr Infect Dis J 25: 577-583.

903. Clark JD, Hill SM, Phillips AD (1988) Investigation of hospital-acquired rotavirus gastroenteritis using RNA electrophoresis. J Med Virol 26: 289-299.

904. Clark KJ, Sarr AB, Grant PG, Phillips TD, Woode GN (1998) In vitro studies on the use of clay, clay minerals and charcoal to adsorb bovine rotavirus and bovine coronavirus. Vet Microbiol 63: 137-146.

905. Clark KJ, Tamborello TJ, Xu Z, Mann FE, Jr., Bonnot CE, et al. (1996) An unusual group-A rotavirus associated with an epidemic of diarrhea among three-month-old calves. J Am Vet Med Assoc 208: 552-554.

906. Clemens J, Keckich N, Naficy A, Glass R, Rao M (1999) Public health considerations for the introduction of new rotavirus vaccines for infants: a case study of tetravalent rhesus rotavirus-based reassortant vaccine. Epidemiol Rev 21: 24-42.

907. Clemens J, Rao M, Ahmed F, Ward R, Huda S, et al. (1993) Breast-feeding and the risk of life-threatening rotavirus diarrhea: prevention or postponement? Pediatrics 92: 680-685.

908. Clemens JD, Ahmed M, Butler T, Greenough WB, 3rd, Sack DA, et al. (1983) Rotavirus diarrhoea: an expanding clinical spectrum. J Trop Med Hyg 86: 117-122.

909. Clemens JD, Ward RL, Rao MR, Sack DA, Knowlton DR, et al. (1992) Seroepidemiologic evaluation of antibodies to rotavirus as correlates of the risk of clinically significant rotavirus diarrhea in rural Bangladesh. J Infect Dis 165: 161-165.

910. Clementi M, Pauri P, Bagnarelli P, Carloni G, Calegari L (1981) Diagnosis of human rotavirus infections: comparison of an electrophoretic method, a modified complement fixation test and electron microscopy for rotavirus detection. Arch Virol 67: 341-344.

911. Clements-Mann ML, Dudas R, Hoshino Y, Nehring P, Sperber E, et al. (2001) Safety and immunogenicity of live attenuated quadrivalent human-bovine (UK) reassortant rotavirus vaccine administered with childhood vaccines to infants. Vaccine 19: 4676-4684.

912. Cleton-Soeteman MI, van Pelt BC (1977) [Gastroenteritis due to rotavirus and spreading of the infection through the hospital]. Ned Tijdschr Geneeskd 121: 866-869.

913. Cobeljic M, Mel D, Arsic B, Krstic L, Sokolovski B, et al. (1989) The association of enterotoxigenic and enteropathogenic Escherichia coli and other enteric pathogens with childhood diarrhoea in Yugoslavia. Epidemiol Infect 103: 53-62.

914. Codd AA, Narang HK (1986) An ion-exchange capture technique for routine identification of faecal viruses by electron microscopy. J Virol Methods 14: 229-235.

915. Coelho C, Vinatea CE, Heinert AP, Simoes CM, Barardi CR (2003) Comparison between specific and multiplex reverse transcription-polymerase chain reaction for detection of hepatitis A virus, poliovirus and rotavirus in experimentally seeded oysters. Mem Inst Oswaldo Cruz 98: 465-468.

916. Coelho CA, Moreira FL, Maffei HV, Coelho KI (1983) [Incidence of viral particles in children with acute or protracted diarrhea, seen at the Hospital das Clinicas of the Faculdade de Medicina de Botucatu from February 1980 to February 1981]. Rev Inst Med Trop Sao Paulo 25: 113-119.

917. Coelho KI, Bryden AS, Hall C, Flewett TH (1981) Pathology of rotavirus infection in suckling mice: A study by conventional histology, immunofluorescence, ultrathin sections, and scanning electron microscopy. Ultrastruct Pathol 2: 59-80.

918. Coffin SE (2000) Rotavirus Vaccines: Current Controversies and Future Directions. Curr Infect Dis Rep 2: 68-72.

919. Coffin SE, Elser J, Marchant C, Sawyer M, Pollara B, et al. (2006) Impact of acute rotavirus gastroenteritis on pediatric outpatient practices in the United States. Pediatr Infect Dis J 25: 584-589.

920. Cohen J (2001) Medicine. Rethinking a vaccine's risk. Science 293: 1576-1577.

921. Cohen MB (1991) Etiology and mechanisms of acute infectious diarrhea in infants in the United States. J Pediatr 118: S34-39.

922. Cohen MB, Nataro JP, Bernstein DI, Hawkins J, Roberts N, et al. (2005) Prevalence of diarrheagenic Escherichia coli in acute childhood enteritis: a prospective controlled study. J Pediatr 146: 54-61.

923. Coiro JR, Bendati MM, de Almeida Neto AJ, Heuser CF, Vasconcellos VL (1983) Rotavirus infection in Brazilian children with acute enteritis: a seasonal variation study. Am J Trop Med Hyg 32: 1186-1188.

924. Coiro JR, De Almeida Neto AJ, Heuser MC, Bendati MM, Vasconcellos VL (1985) Acute enteritis associated with rotavirus presence in Brazilian children: evaluations on prevalence, therapy and age group. J Diarrhoeal Dis Res 3: 78-83.

925. Coiro JR, Heuser MC, Vasconcellos VL (1988) Rapid laboratory detection of rotavirus in faeces from children with acute enteritis. J Diarrhoeal Dis Res 6: 35-36.

926. Coiro JR, Heuser MC, Vasconcellos VL, Rosa Y (1987) Pathogens associated with acute enteritis in Brazilian children. J Diarrhoeal Dis Res 5: 110-111.

927. Coker AO, Dosunmu-Ogunbi O, Odugbemi T, Alabi SA, Macaulay SA (1987) A study on the prevalence of rotavirus diarrhoeas in Ohaozara local government area, Imo State, Nigeria and the Lagos University Teaching Hospital, Lagos, Nigeria. East Afr Med J 64: 586-589.

928. Collins J, Candy DC, Starkey WG, Spencer AJ, Osborne MP, et al. (1990) Disaccharidase activities in small intestine of rotavirus-infected suckling mice: a histochemical study. J Pediatr Gastroenterol Nutr 11: 395-403.

929. Collins J, Starkey WG, Wallis TS, Clarke GJ, Worton KJ, et al. (1988) Intestinal enzyme profiles in normal and rotavirus-infected mice. J Pediatr Gastroenterol Nutr 7: 264-272.

930. Collins JE, Benfield DA, Duimstra JR (1989) Comparative virulence of two porcine group-A rotavirus isolates in gnotobiotic pigs. Am J Vet Res 50: 827-835.

931. Collins JK, Riegel CA, Olson JD, Fountain A (1987) Shedding of enteric coronavirus in adult cattle. Am J Vet Res 48: 361-365.

932. Collins PJ, Cullinane A, Martella V, O'Shea H (2008) Molecular characterization of equine rotavirus in Ireland. J Clin Microbiol 46: 3346-3354.

933. Collins PJ, Martella V, O'Shea H (2008) Detection and characterization of group C rotaviruses in asymptomatic piglets in ireland. J Clin Microbiol 46: 2973-2979.

934. Colomba C, De Grazia S, Giammanco GM, Saporito L, Scarlata F, et al. (2006) Viral gastroenteritis in children hospitalised in Sicily, Italy. Eur J Clin Microbiol Infect Dis 25: 570-575.

935. Colomina J, Gil MT, Codoner P, Buesa J (1998) Viral proteins VP2, VP6, and NSP2 are strongly precipitated by serum and fecal antibodies from children with rotavirus symptomatic infection. J Med Virol 56: 58-65.

936. Colomina J, Raga J, Gil MT, Buesa J (1998) [Virus-specific serum and fecal antibodies response in children with acute rotavirus gastroenteritis]. Enferm Infecc Microbiol Clin 16: 55-60.

937. Coluchi N, Munford V, Manzur J, Vazquez C, Escobar M, et al. (2002) Detection, subgroup specificity, and genotype diversity of rotavirus strains in children with acute diarrhea in Paraguay. J Clin Microbiol 40: 1709-1714.

938. Combee CL, Collinge ML, Britt EM (1986) Cryptosporidiosis in a hospital-associated day care center. Pediatr Infect Dis 5: 528-532.

939. Combiescu AA, Persu A, Mazilu M, Moldovan L, Ficiu M, et al. (1985) [ELISA-IC for the detection of the rotavirus group antigen, in the stools of children with acute gastroenteritis]. Rev Ig Bacteriol Virusol Parazitol Epidemiol Pneumoftiziol Bacteriol Virusol Parazitol Epidemiol 30: 317-326.

940. Compton SR, Homberger FR, Paturzo FX, Clark JM (2004) Efficacy of three microbiological monitoring methods in a ventilated cage rack. Comp Med 54: 382-392.

941. Cone R, Mohan K, Thouless M, Corey L (1988) Nosocomial transmission of rotavirus infection. Pediatr Infect Dis J 7: 103-109.

942. Conner M, Kalica A, Kita J, Quick S, Schiff E, et al. (1984) Isolation and characteristics of an equine reovirus type 3 and an antibody prevalence survey to reoviruses in horses located in New York State. Vet Microbiol 9: 15-25.

943. Conner ME, Darlington RW (1980) Rotavirus infection in foals. Am J Vet Res 41: 1699-1703.

944. Conner ME, Gillespie JH, Schiff EI, Frey MS (1983) Detection of rotavirus in horses with and without diarrhea by electron microscopy and Rotazyme test. Cornell Vet 73: 280-287.

945. Constantiniu S, Avram G, Ambarus A, Zavate O (1991) Studies of bacterial, rotaviral and Cryptosporidium etiology of acute diarrheal diseases in hospitalized children. Roum Arch Microbiol Immunol 50: 53-60.

946. Constenla D, Ortega-Barria E, Rheingans RD, Antil L, Saez-Llorens X (2008) [Economic impact of rotavirus vaccination in Panama]. An Pediatr (Barc) 68: 128-135.

947. Constenla D, O'Ryan M, Navarrete MS, Antil L, Rheingans RD (2006) [Potential cost effectiveness of a rotavirus vaccine in Chile]. Rev Med Chil 134: 679-688.

948. Constenla D, Perez-Schael I, Rheingans RD, Antil L, Salas H, et al. (2006) [Assessment of the economic impact of the antiretroviral vaccine in Venezuela]. Rev Panam Salud Publica 20: 213-222.

949. Constenla DO, Linhares AC, Rheingans RD, Antil LR, Waldman EA, et al. (2008) Economic impact of a rotavirus vaccine in Brazil. J Health Popul Nutr 26: 388-396.

950. Contino MF, Lebby T, Arcinue EL (1994) Rotaviral gastrointestinal infection causing afebrile seizures in infancy and childhood. Am J Emerg Med 12: 94-95.

951. Contrepois M, Gouet P (1983) [Kinetics of fecal excretion of Escherichia coli K99+ by calves after experimental infection]. Ann Rech Vet 14: 141-146.

952. Contreras G (1989) Effect of the administration of oral poliovirus vaccine on infantile diarrhoea mortality. Vaccine 7: 211-212.

953. Contreras JF, Menchaca GE, Padilla-Noriega L, Tamez RS, Greenberg HB, et al. (1995) Heterogeneity of VP4 neutralization epitopes among serotype P1A human rotavirus strains. Clin Diagn Lab Immunol 2: 506-508.

954. Conway PH, Edwards S, Stucky ER, Chiang VW, Ottolini MC, et al. (2006) Variations in management of common inpatient pediatric illnesses: hospitalists and community pediatricians. Pediatrics 118: 441-447.

955. Conway SP (1989) Rotavirus gastro-enteritis presenting as acute intestinal obstruction. J Infect 18: 97-98.

956. Cook SM, Glass RI, LeBaron CW, Ho MS (1990) Global seasonality of rotavirus infections. Bull World Health Organ 68: 171-177.

957. Cooper GL, Charlton BR, Bickford AA, Nordhausen R (2004) Hexamita meleagridis (Spironucleus meleagridis) infection in chukar partridges associated with high mortality and intracellular trophozoites. Avian Dis 48: 706-710.

958. Cooper VL (2000) Diagnosis of neonatal pig diarrhea. Vet Clin North Am Food Anim Pract 16: 117-133.

959. Cordle CT, Schaller JP, Winship TR, Candler EL, Hilty MD, et al. (1991) Passive immune protection from diarrhea caused by rotavirus or E. coli: an animal model to demonstrate and quantitate efficacy. Adv Exp Med Biol 310: 317-327.

960. Corl BA, Harrell RJ, Moon HK, Phillips O, Weaver EM, et al. (2007) Effect of animal plasma proteins on intestinal damage and recovery of neonatal pigs infected with rotavirus. J Nutr Biochem 18: 778-784.

961. Corl BA, Odle J, Niu X, Moeser AJ, Gatlin LA, et al. (2008) Arginine activates intestinal p70(S6k) and protein synthesis in piglet rotavirus enteritis. J Nutr 138: 24-29.

962. Corl BA, Odle J, Niu X, Moeser AJ, Gatlin LA, et al. (2008) Arginine activates intestinal p70(S6k) and protein synthesis in piglet rotavirus enteritis. J Nutr 138: 24-29.

963. Cornaglia E, Elazhary Y, Talbot B (1993) Bovine rotavirus type detection by neutralizing monoclonal antibodies. Arch Virol 129: 243-250.

964. Cornaglia EM, Elazhary YM, Brodeur BR, Talbot BG (1992) Monoclonal anti-idiotype induces antibodies against bovine Q17 rotavirus. J Virol 66: 5763-5769.

965. Cornaglia EM, Fernandez FM, Gottschalk M, Barrandeguy ME, Luchelli A, et al. (1992) Reduction in morbidity due to diarrhea in nursing beef calves by use of an inactivated oil-adjuvanted rotavirus-Escherichia coli vaccine in the dam. Vet Microbiol 30: 191-202.

966. Cornell SL (1997) Confronting the consequences of rotavirus: diarrhea and dehydration. Adv Nurse Pract 5: 41-44.

967. Correa A, Solarte Y, Barrera J, Mogollon D, Gutierrez MF (1999) [Molecular characterization of rotavirus in the city of Santafe de Bogota, Colombia. Determination of the electrophenotypes and typing of a strain by RT-PCR]. Rev Latinoam Microbiol 41: 167-173.

968. Corthier G (1981) Detection of rotavirus immune complexes: relationship between rotavirus antibodies and rotavirus antigens in faeces. J Virol Methods 3: 277-282.

969. Corthier G, Cohen J, Scherrer R (1980) Isolation of pig rotavirus in France. Identification and experimental infections. Ann Rech Vet 11: 45-48.

970. Corthier G, Moreau C, Muller MC, Dubos F (1985) Influence of rotaviral diarrhea on intestinal microflora establishment in conventional and axenic mice. Prog Clin Biol Res 181: 179-181.

971. Corwin AL, Subekti D, Sukri NC, Willy RJ, Master J, et al. (2005) A large outbreak of probable rotavirus in Nusa Tenggara Timur, Indonesia. Am J Trop Med Hyg 72: 488-494.

972. Costa C, Candeias JA, Capeletti EL (1990) [Electropherotypes of rotavirus in children with and without gastroenteritis]. Rev Saude Publica 24: 152-155.

973. Costa PS, Cardoso DD, Grisi SJ, Silva PA, Fiaccadori F, et al. (2004) [Rotavirus A infections and reinfections: genotyping and vaccine implications]. J Pediatr (Rio J) 80: 119-122.

974. Costa Sde M, Gomes TA, Haapalainen E, Fagundes-Neto U (1997) [Scanning electronic microscopy of the small intestine in persistent diarrhea]. Arq Gastroenterol 34: 112-120.

975. Costantini V, Parreno V, Barrandeguy M, Combessies G, Bardon JC, et al. (2002) [Group A bovine rotavirus: diagnosis and antigenic characterization of strains circulating in the Argentine Republic, 1994-1999]. Rev Argent Microbiol 34: 110-116.

976. Coste A, Cohen J, Reinhardt M, Kraehenbuhl JP, Sirard JC (2001) Nasal immunisation with Salmonella typhimurium producing rotavirus VP2 and VP6 antigens stimulates specific antibody response in serum and milk but fails to protect offspring. Vaccine 19: 4167-4174.

977. Coste A, Sirard JC, Johansen K, Cohen J, Kraehenbuhl JP (2000) Nasal immunization of mice with virus-like particles protects offspring against rotavirus diarrhea. J Virol 74: 8966-8971.

978. Cotterill AM, Walker-Smith JA (1986) Childhood epidemiology. Gastro-intestinal tract. Br Med Bull 42: 176-180.

979. Coulson BS (1987) Variation in neutralization epitopes of human rotaviruses in relation to genomic RNA polymorphism. Virology 159: 209-216.

980. Coulson BS (1998) Longitudinal studies of neutralizing antibody responses to rotavirus in stools and sera of children following severe rotavirus gastroenteritis. Clin Diagn Lab Immunol 5: 897-901.

981. Coulson BS, Grimwood K, Bishop RF, Barnes GL (1989) Evaluation of end-point titration, single dilution and capture enzyme immunoassays for measurement of antirotaviral IgA and IgM in infantile secretions and serum. J Virol Methods 26: 53-65.

982. Coulson BS, Grimwood K, Hudson IL, Barnes GL, Bishop RF (1992) Role of coproantibody in clinical protection of children during reinfection with rotavirus. J Clin Microbiol 30: 1678-1684.

983. Coulson BS, Grimwood K, Masendycz PJ, Lund JS, Mermelstein N, et al. (1990) Comparison of rotavirus immunoglobulin A coproconversion with other indices of rotavirus infection in a longitudinal study in childhood. J Clin Microbiol 28: 1367-1374.

984. Coulson BS, Holmes IH (1984) An improved enzyme-linked immunosorbent assay for the detection of rotavirus in faeces of neonates. J Virol Methods 8: 165-179.

985. Coulson BS, Masendycz PJ (1990) Measurement of rotavirus-neutralizing coproantibody in children by fluorescent focus reduction assay. J Clin Microbiol 28: 1652-1654.

986. Courouble G, Dufillot D, Sans A, Malpote E, Berchel C, et al. (2000) [Acute childhood gastroenteritis study at Central University Hospital of Pointe-a-Pitre/Abymes, Guadeloupe, from November 1997 to March 1998]. Bull Soc Pathol Exot 93: 58-61.

987. Coussement W, Ducatelle R, Debouck P, Hoorens J (1982) Pathology of experimental CV777 coronavirus enteritis in piglets. I. Histological and histochemical study. Vet Pathol 19: 46-56.

988. Coussement W, Ducatelle R, Geeraerts G, Berghen P (1981) Baby pig diarrhea caused by coccidiosis. Vet Q 3: 57-60.

989. Coutinho RA, van der Noordaa J (1976) [Rotavirus as a cause of acute gastroenteritis]. Ned Tijdschr Geneeskd 120: 1898-1899.

990. Cowden JM (2001) An estimate of the costs of cases of rotavirus infection admitted to hospital in Scotland, 1997. Health Bull (Edinb) 59: 188-192.

991. Cox GJ, Matsui SM, Lo RS, Hinds M, Bowden RA, et al. (1994) Etiology and outcome of diarrhea after marrow transplantation: a prospective study. Gastroenterology 107: 1398-1407.

992. Cox MJ, James VL, Azevedo RS, Massad E, Medley GF (1998) Infection with group C rotavirus in a suburban community in Brazil. Trop Med Int Health 3: 891-895.

993. Cravioto A, Reyes RE, Trujillo F, Uribe F, Navarro A, et al. (1990) Risk of diarrhea during the first year of life associated with initial and subsequent colonization by specific enteropathogens. Am J Epidemiol 131: 886-904.

994. Crawford SE, Estes MK, Ciarlet M, Barone C, O'Neal CM, et al. (1999) Heterotypic protection and induction of a broad heterotypic neutralization response by rotavirus-like particles. J Virol 73: 4813-4822.

995. Crawford SE, Patel DG, Cheng E, Berkova Z, Hyser JM, et al. (2006) Rotavirus viremia and extraintestinal viral infection in the neonatal rat model. J Virol 80: 4820-4832.

996. Crawley JM, Bishop RF, Barnes GL (1993) Rotavirus gastroenteritis in infants aged 0-6 months in Melbourne, Australia: implications for vaccination. J Paediatr Child Health 29: 219-221.

997. Creese AL (1986) Cost effectiveness of potential immunization interventions against diarrhoeal disease. Soc Sci Med 23: 231-240.

998. Crewe E, Murphy AM (1980) Further studies on neonatal rotavirus infections. Med J Aust 1: 61-63.

999. Cromien JL, Himmelreich CA, Glass RI, Storch GA (1987) Evaluation of new commercial enzyme immunoassay for rotavirus detection. J Clin Microbiol 25: 2359-2362.

1000. Crotti D, D'Annibale ML (2007) [Acute and prolonged infectious diarrheas, of microbial and viral etiology: methods of clinical and microbiological diagnosis]. Recenti Prog Med 98: 553-559.

1001. Crotti D, D'Annibale ML, Fonzo G, Medori MC, Ubaldi M (2002) [Enteric infections in Perugia's area: laboratory diagnosis, clinical aspects and epidemiology during 2001]. Infez Med 10: 81-87.

1002. Crouch CF (1985) Vaccination against enteric rota and coronaviruses in cattle and pigs: enhancement of lactogenic immunity. Vaccine 3: 284-291.

1003. Crouch CF, Acres SD (1984) Prevalence of rotavirus and coronavirus antigens in the feces of normal cows. Can J Comp Med 48: 340-342.

1004. Crowley DS, Ryan MJ, Wall PG (1997) Gastroenteritis in children under 5 years of age in England and Wales. Commun Dis Rep CDR Rev 7: R82-86.

1005. Croxson MC, Bellamy AR (1979) Two strains of human rotavirus in Auckland. N Z Med J 90: 235-237.

1006. Cruickshank JG, Zilberg G (1976) Winter diarrhoea and rotaviruses in Rhodesia. S Afr Med J 50: 1895-1896.

1007. Cruickshank R, Ashdown L, Croese J (1988) Human cryptosporidiosis in North Queensland. Aust N Z J Med 18: 582-586.

1008. Cruz JR, Caceres P, Cano F, Flores J, Bartlett A, et al. (1990) Adenovirus types 40 and 41 and rotaviruses associated with diarrhea in children from Guatemala. J Clin Microbiol 28: 1780-1784.

1009. Cryan B, Lynch M, Whyte D (1997) Rotavirus in Ireland. Euro Surveill 2: 15-16.

1010. Cuadras MA, Feigelstock DA, An S, Greenberg HB (2002) Gene expression pattern in Caco-2 cells following rotavirus infection. J Virol 76: 4467-4482.

1011. Cubitt WD, Holzel H (1980) An outbreak of rotavirus infection in a long-stay ward of a geriatric hospital. J Clin Pathol 33: 306-308.

1012. Cucchiara S, Falconieri P, Di Nardo G, Parcelii MA, Dito L, et al. (2002) New therapeutic approach in the management of intestinal disease: probiotics in intestinal disease in paediatric age. Dig Liver Dis 34 Suppl 2: S44-47.

1013. Cuckova J, Valicek L, Macku M, Bartosova D (1983) [The role of rotaviruses in the etiology of acute gastroenteritis in children hospitalized at the Pediatric Teaching Hospital in Brno]. Cas Lek Cesk 122: 984-987.

1014. Cuestas Montanes E, Appendino Camacho J, Valle Toselli M (2005) [Rotavirus diarrhea in a population covered by private health insurance in Cordoba, Argentina]. An Pediatr (Barc) 63: 369-372.

1015. Cui F (1992) [Epidemiological investigation on pathogens of acute infectious diarrhea in Ning Xia]. Zhonghua Liu Xing Bing Xue Za Zhi 13: 85-88.

1016. Cui MC, Li CH (2008) [Clinical observation on drug-separated moxibustion at Shenque (CV 8) for treatment of infantile autumn diarrhea]. Zhongguo Zhen Jiu 28: 194-196.

1017. Cukor G, Berry MK, Blacklow NR (1978) Simplified radioimmunoassay for detection of human rotavirus in stools. J Infect Dis 138: 906-910.

1018. Cukor G, Blacklow NR (1984) Human viral gastroenteritis. Microbiol Rev 48: 157-179.

1019. Cukor G, Blacklow NR, Braverman LE (1982) Antibodies to gastroenteritis viruses in cystic fibrosis patients. J Med Virol 9: 161-164.

1020. Cukor G, Blacklow NR, Capozza FE, Panjvani ZF, Bednarek F (1979) Persistence of antibodies to rotavirus in human milk. J Clin Microbiol 9: 93-96.

1021. Cukor G, Perron DM, Hudson R, Blacklow NR (1984) Detection of rotavirus in human stools by using monoclonal antibody. J Clin Microbiol 19: 888-892.

1022. Cunliffe N, Nakagomi O (2007) Introduction of rotavirus vaccines in developing countries: remaining challenges. Ann Trop Paediatr 27: 157-167.

1023. Cunliffe NA, Allan C, Lowe SJ, Sopwith W, Booth AJ, et al. (2007) Healthcare-associated rotavirus gastroenteritis in a large paediatric hospital in the UK. J Hosp Infect 67: 240-244.

1024. Cunliffe NA, Bresee JS, Hart CA (2002) Rotavirus vaccines: development, current issues and future prospects. J Infect 45: 1-9.

1025. Cunliffe NA, Dove W, Bunn JE, Ben Ramadam M, Nyangao JW, et al. (2001) Expanding global distribution of rotavirus serotype G9: detection in Libya, Kenya, and Cuba. Emerg Infect Dis 7: 890-892.

1026. Cunliffe NA, Dove W, Jiang B, Thinwda Cert BD, Broadhead RL, et al. (2001) Detection of group C rotavirus in children with acute gastroenteritis in Blantyre, Malawi. Pediatr Infect Dis J 20: 1088-1090.

1027. Cunliffe NA, Gentsch JR, Kirkwood CD, Gondwe JS, Dove W, et al. (2000) Molecular and serologic characterization of novel serotype G8 human rotavirus strains detected in Blantyre, Malawi. Virology 274: 309-320.

1028. Cunliffe NA, Gondwe JS, Broadhead RL, Molyneux ME, Woods PA, et al. (1999) Rotavirus G and P types in children with acute diarrhea in Blantyre, Malawi, from 1997 to 1998: predominance of novel P[6]G8 strains. J Med Virol 57: 308-312.

1029. Cunliffe NA, Gondwe JS, Graham SM, Thindwa BD, Dove W, et al. (2001) Rotavirus strain diversity in Blantyre, Malawi, from 1997 to 1999. J Clin Microbiol 39: 836-843.

1030. Cunliffe NA, Gondwe JS, Kirkwood CD, Graham SM, Nhlane NM, et al. (2001) Effect of concomitant HIV infection on presentation and outcome of rotavirus gastroenteritis in Malawian children. Lancet 358: 550-555.

1031. Cunliffe NA, Kilgore PE, Bresee JS, Steele AD, Luo N, et al. (1998) Epidemiology of rotavirus diarrhoea in Africa: a review to assess the need for rotavirus immunization. Bull World Health Organ 76: 525-537.

1032. Cunliffe NA, Nakagomi O (2005) A critical time for rotavirus vaccines: a review. Expert Rev Vaccines 4: 521-532.

1033. Cunliffe NA, Ngwira BM, Dove W, Nakagomi O, Nakagomi T, et al. (2009) Serotype g12 rotaviruses, Lilongwe, Malawi. Emerg Infect Dis 15: 87-90.

1034. Cunliffe NA, Rogerson S, Dove W, Thindwa BD, Greensill J, et al. (2002) Detection and characterization of rotaviruses in hospitalized neonates in Blantyre, Malawi. J Clin Microbiol 40: 1534-1537.

1035. Cunliffe NA, Woods PA, Leite JP, Das BK, Ramachandran M, et al. (1997) Sequence analysis of NSP4 gene of human rotavirus allows classification into two main genetic groups. J Med Virol 53: 41-50.

1036. Cunningham AL, Grohman GS, Harkness J, Law C, Marriott D, et al. (1988) Gastrointestinal viral infections in homosexual men who were symptomatic and seropositive for human immunodeficiency virus. J Infect Dis 158: 386-391.

1037. Cushing AH, Anderson L (1982) Diarrhea in breast-fed and non-breast-fed infants. Pediatrics 70: 921-925.

1038. Cusi MG, Valensin PE, Barberi A, Guglielmetti P, Rossolini A (1986) Viral childhood gastroenteritis. Microbiologica 9: 471-478.

1039. Cutillo S (1978) [Rotavirus infections]. Pediatria (Napoli) 86: VI-VIII.

1040. Cutting WA (1979) Viral gastroenteritis in children. Trop Doct 9: 16-20.

1041. da Costa Mendes VM, de Beer M, Peenze I, Steele AD (1993) Molecular epidemiology and subgroup analysis of bovine group A rotaviruses associated with diarrhea in South African calves. J Clin Microbiol 31: 3333-3335.

1042. da Costa Mendes VM, De Beer MC, Els HJ, Goosen GH, Theron J, et al. (1994) Rotavirus in Saanen goats. J S Afr Vet Assoc 65: 132-133.

1043. da Rosa e Silva ML, Naveca FG, Pires de Carvalho I (2001) Epidemiological aspects of rotavirus infections in Minas Gerais, Brazil. Braz J Infect Dis 5: 215-222.

1044. da Silva Domingues AL, da Silva Vaz MG, Moreno M, Camara FP (2000) Molecular epidemiology of group A rotavirus causing acute diarrhea in infants and young children hospitalized in Rio de Janeiro, Brazil, 1995-1996. Braz J Infect Dis 4: 119-125.

1045. da Silva S, da Silva SP, Gouveia Yde S, da Silva Nde O, Melo ME, et al. (2003) [Occurrence of Cryptosporidium sp in fecal samples of children less than 10 years old with clinical indication of Rotavirus]. Rev Soc Bras Med Trop 36: 421-423.

1046. da Silva Vaz M, da Silva Domingues AL, Moreno M, Portela Camara F (1999) Molecular Epidemiology of Group A Rotavirus Causing Acute Diarrhea in Infants and Young Children in Rio de Janeiro, Brazil, 1997-1998. Braz J Infect Dis 3: 156-162.

1047. Dagan R, Bar-David Y, Sarov B, Katz M, Kassis I, et al. (1990) Rotavirus diarrhea in Jewish and Bedouin children in the Negev region of Israel: epidemiology, clinical aspects and possible role of malnutrition in severity of illness. Pediatr Infect Dis J 9: 314-321.

1048. D'Agostino J (2006) Considerations in assessing the clinical course and severity of rotavirus gastroenteritis. Clin Pediatr (Phila) 45: 203-212.

1049. Dai GZ, Sun MS, Liu SQ, Ding XF, Chen YD, et al. (1987) First report of an epidemic of diarrhoea in human neonates involving the new rotavirus and biological characteristics of the epidemic virus strain (KMB/R85). J Med Virol 22: 365-373.

1050. Dai GZ, Yang YF, Li JX, Ren LP, Zhang XS, et al. (1982) [Studies on the etiology of autumnal acute gastroenteritis in Kunming. II. Determination of rotavirus by an enzyme-linked immunosorbent assay]. Zhongguo Yi Xue Ke Xue Yuan Xue Bao 4: 397-398.

1051. Dai GZ, Zhang XS, Li JX, Yang YF, Ren LP, et al. (1982) [Studies on the etiology of automnal acute gastroenteritis in Kunming. I. A preliminary report on the detection of rotavirus]. Zhongguo Yi Xue Ke Xue Yuan Xue Bao 4: 395-396.

1052. Dalton RM, Roman ER, Negredo AA, Wilhelmi ID, Glass RI, et al. (2002) Astrovirus acute gastroenteritis among children in Madrid, Spain. Pediatr Infect Dis J 21: 1038-1041.

1053. D'Angelo G, Angeletti C, Catassi C, Coppa GV (1998) [Probiotics in childhood]. Minerva Pediatr 50: 163-173.

1054. Dani C, Trevisanuto D, Cantarutti F, Zanardo V (1994) [A case of neonatal necrotizing enterocolitis due to rotavirus]. Pediatr Med Chir 16: 185-186.

1055. Danner K (1983) [Viral enteritis in cattle]. Tierarztl Prax 11: 149-161.

1056. Danovaro-Holliday MC, Wood AL, LeBaron CW (2002) Rotavirus vaccine and the news media, 1987-2001. Jama 287: 1455-1462.

1057. Dar AM, Kapil S, Goyal SM (1998) Comparison of immunohistochemistry, electron microscopy, and direct fluorescent antibody test for the detection of bovine coronavirus. J Vet Diagn Invest 10: 152-157.

1058. Dar VS, Ghosh S, Broor S (1994) Rapid detection of rotavirus by using colloidal gold particles labeled with monoclonal antibody. J Virol Methods 47: 51-58.

1059. Das BK, Gentsch JR, Hoshino Y, Ishida S, Nakagomi O, et al. (1993) Characterization of the G serotype and genogroup of New Delhi newborn rotavirus strain 116E. Virology 197: 99-107.

1060. Das D, Metzger K, Heffernan R, Balter S, Weiss D, et al. (2005) Monitoring over-the-counter medication sales for early detection of disease outbreaks--New York City. MMWR Morb Mortal Wkly Rep 54 Suppl: 41-46.

1061. das Dores de Paula Cardoso D, Fiaccadori FS, Borges de Lima Dias e Souza M, Bringel Martins RM, Gagliardi Leite JP (2002) Detection and genotyping of astroviruses from children with acute gastroenteritis from Goiania, Goias, Brazil. Med Sci Monit 8: CR624-628.

1062. Das S, Varghese V, Chaudhuri S, Barman P, Kojima K, et al. (2004) Genetic variability of human rotavirus strains isolated from Eastern and Northern India. J Med Virol 72: 156-161.

1063. Das S, Varghese V, Chaudhury S, Barman P, Mahapatra S, et al. (2003) Emergence of novel human group A rotavirus G12 strains in India. J Clin Microbiol 41: 2760-2762.

1064. Dattani SJ, Connelly JF (1996) Oral immunoglobulins for gastroenteritis. Ann Pharmacother 30: 1323-1324.

1065. Dauvergne M, Brun A, Soulebot JP (1983) Passive protection of newborn calves against rotavirus by vaccination of their dams. Dev Biol Stand 53: 245-255.

1066. David E, Andronescu D, Serban D, Jebeleanu L, Cocean S, et al. (1996) [The etiology of acute diarrheal diseases in hospitalized children and as outpatients]. Bacteriol Virusol Parazitol Epidemiol 41: 37-41.

1067. Davidson G, Elliott EJ, Kirkwood C, Pearce R (2007) Preventing rotavirus gastroenteritis: do you have the facts? J Paediatr Child Health 43: 564-567.

1068. Davidson GP (1986) Viral diarrhoea. Clin Gastroenterol 15: 39-53.

1069. Davidson GP (1996) Passive protection against diarrheal disease. J Pediatr Gastroenterol Nutr 23: 207-212.

1070. Davidson GP, Barnes GL (1979) Structural and functional abnormalities of the small intestine in infants and young children with rotavirus enteritis. Acta Paediatr Scand 68: 181-186.

1071. Davidson GP, Bishop RF, Townley RR, Holmes IH (1975) Importance of a new virus in acute sporadic enteritis in children. Lancet 1: 242-246.

1072. Davidson GP, Butler RN (2000) Probiotics in pediatric gastrointestinal disorders. Curr Opin Pediatr 12: 477-481.

1073. Davidson GP, Gall DG, Petric M, Butler DG, Hamilton JR (1977) Human rotavirus enteritis induced in conventional piglets. Intestinal structure and transport. J Clin Invest 60: 1402-1409.

1074. Davidson GP, Hogg RJ, Kirubakaran CP (1983) Serum and intestinal immune response to rotavirus enteritis in children. Infect Immun 40: 447-452.

1075. Davidson GP, Whyte PB, Daniels E, Franklin K, Nunan H, et al. (1989) Passive immunisation of children with bovine colostrum containing antibodies to human rotavirus. Lancet 2: 709-712.

1076. Davis JF, McMurtry JP, Vasilatos-Younken R, Connolly BM, Woolcock PR, et al. (1997) Experimental reproduction of a spiking mortality syndrome of turkeys. Avian Dis 41: 269-278.

1077. De A, Nanivadekar R, Mathur M, Gogate A, Kulkarni MV (2005) Prevalence of rotaviral diarrhoea in hospitalized children. Indian J Med Microbiol 23: 67-68.

1078. de Angelis GL, Zanacca C, Banchini G, Caprio P, Bernasconi S (1987) [Home-coming diarrhea. Presentation of a clinical case]. Pediatr Med Chir 9: 237-238.

1079. de Beer M, Peenze I, da Costa Mendes VM, Steele AD (1997) Comparison of electron microscopy, enzyme-linked immunosorbent assay and latex agglutination for the detection of bovine rotavirus in faeces. J S Afr Vet Assoc 68: 93-96.

1080. De Boissieu D, Lebon P, Badoual J, Bompard Y, Dupont C (1993) Rotavirus induces alpha-interferon release in children with gastroenteritis. J Pediatr Gastroenterol Nutr 16: 29-32.

1081. de Bruin E, Duizer E, Vennema H, Koopmans MP (2006) Diagnosis of Norovirus outbreaks by commercial ELISA or RT-PCR. J Virol Methods 137: 259-264.

1082. de Castro L, Rodrigues Ddos P, Flauzino R, Moura M, Leite JP (1994) An outbreak of diarrhoea associated with rotavirus serotype 1 in a day care nursery in Rio de Janeiro, Brazil. Mem Inst Oswaldo Cruz 89: 5-9.

1083. de Gamarra E, Helardot P, Moriette G, Murat I, Relier JP (1983) Necrotizing enterocolitis in full-term newborns. Biol Neonate 44: 185-192.

1084. De Grazia S, Giammanco GM, Martella V, Ramirez S, Colomba C, et al. (2008) Rare AU-1-like G3P[9] human rotaviruses with a Kun-like NSP4 gene detected in children with diarrhea in Italy. J Clin Microbiol 46: 357-360.

1085. De Grazia S, Ramirez S, Giammanco GM, Colomba C, Martella V, et al. (2007) Diversity of human rotaviruses detected in Sicily, Italy, over a 5-year period (2001-2005). Arch Virol 152: 833-837.

1086. De Groote G, Desmyter J, Vantrappen G, Phillips CA (1977) Rotavirus antibodies in Crohn's disease and ulcerative colitis. Lancet 1: 1263-1264.

1087. de la Cruz F, Guzman MG, Esquivel M, Grandio O, Vazquez S, et al. (1990) [Clinical and epidemiologic aspects of acute diarrhea caused by rotavirus in children. Cuba, 1982-1984]. Rev Cubana Med Trop 42: 178-187.

1088. de la Fuente R, Garcia A, Ruiz-Santa-Quiteria JA, Luzon M, Cid D, et al. (1998) Proportional morbidity rates of enteropathogens among diarrheic dairy calves in central Spain. Prev Vet Med 36: 145-152.

1089. de la Fuente R, Luzon M, Ruiz-Santa-Quiteria JA, Garcia A, Cid D, et al. (1999) Cryptosporidium and concurrent infections with other major enterophatogens in 1 to 30-day-old diarrheic dairy calves in central Spain. Vet Parasitol 80: 179-185.

1090. de la Vega SL, Osornio-Vargas AR, Ruiz-Palacios GM (1987) [Coagglutination test for detecting human rotaviruses in feces. Comparison with the ELISA test]. Bol Med Hosp Infant Mex 44: 373-379.

1091. De Leener K, Rahman M, Matthijnssens J, Van Hoovels L, Goegebuer T, et al. (2004) Human infection with a P[14], G3 lapine rotavirus. Virology 325: 11-17.

1092. De Leeuw PW (1976) [Rotavirus infection in calves (author's transl)]. Tijdschr Diergeneeskd 101: 1298-1305.

1093. de Leeuw PW, Ellens DJ, Straver PJ, van Balken JA, Moerman A, et al. (1980) Rotavirus infections in calves in dairy herds. Res Vet Sci 29: 135-141.

1094. de Leeuw PW, Ellens DJ, Talmon FP, Zimmer GN, Kommerij R (1980) Rotavirus infections in calves: efficacy of oral vaccination in endemically infected herds. Res Vet Sci 29: 142-147.

1095. de Leeuw PW, Tiessink JW (1985) Laboratory experiments on oral vaccination of calves against rotavirus or coronavirus induced diarrhoea. Zentralbl Veterinarmed B 32: 55-64.

1096. de Leeuw PW, van Nieuwstadt AP, van Balken JA, Ellens DJ (1977) Rotavirus infections in calves. II. Experimental infections with a Dutch isolate. Tijdschr Diergeneeskd 102: 515-524.

1097. de Mendonca JS (1982) [Infectious diarrhea of viral and bacterial etiology]. Rev Paul Med 100: 24-29.

1098. de Mol P (1979) [Pediatric aspects of acute infectious diarrhea]. Acta Gastroenterol Belg 42: 509-514.

1099. De Mol P, Zissis G, Butzler JP, Mutwewingabo A, Andre FE (1986) Failure of live, attenuated oral rotavirus vaccine. Lancet 2: 108.

1100. de Oliveira LH, Danovaro-Holliday MC, Matus CR, Andrus JK (2008) Rotavirus vaccine introduction in the Americas: progress and lessons learned. Expert Rev Vaccines 7: 345-353.

1101. de Roos NM, Katan MB (2000) Effects of probiotic bacteria on diarrhea, lipid metabolism, and carcinogenesis: a review of papers published between 1988 and 1998. Am J Clin Nutr 71: 405-411.

1102. de Rougemont A, Kaplon J, Lebon P, Huet F, Denis F, et al. (2008) Unexpected substitution of dominant rotavirus G genotypes in French hospitalized children over five consecutive seasons. Eur J Clin Microbiol Infect Dis.

1103. De Rycke J, Bernard S, Laporte J, Naciri M, Popoff MR, et al. (1986) Prevalence of various enteropathogens in the feces of diarrheic and healthy calves. Ann Rech Vet 17: 159-168.

1104. De Rycke J, Le Roux P, Melik N, Raimbault P (1982) [Frequency of enteropathogenic K99+ ST+ Escherichia coli and rotaviruses in neonatal diarrhea of calves. Survey of a veterinarian's clientele in Sarthe]. Ann Rech Vet 12: 403-411.

1105. De Sierra MJ, Sanchez AM, Quiricci L, Diamont A, Rodriguez G, et al. (2002) Electropherotypes of rotaviral RNA from cases of infantile diarrhea in Uruguay. Acta Virol 46: 103-106.

1106. de Silva DG, Mendis L, Soysa P (1984) Rota virus associated carbohydrate intolerance in Sri Lankan children. Ceylon Med J 29: 193-197.

1107. de Silva DG, Mendis LN, Sheron N, Alexander GJ, Candy DC, et al. (1993) Concentrations of interleukin 6 and tumour necrosis factor in serum and stools of children with Shigella dysenteriae 1 infection. Gut 34: 194-198.

1108. de Soarez PC, Valentim J, Sartori AM, Novaes HM (2008) Cost-effectiveness analysis of routine rotavirus vaccination in Brazil. Rev Panam Salud Publica 23: 221-230.

1109. de Verdier K, Ohagen P, Alenius S (2003) No effect of a homeopathic preparation on neonatal calf diarrhoea in a randomised double-blind, placebo-controlled clinical trial. Acta Vet Scand 44: 97-101.

1110. de Verdier Klingenberg K (2000) Enhancement of clinical signs in experimentally rotavirus infected calves by combined viral infections. Vet Rec 147: 717-719.

1111. de Verdier Klingenberg K, Nilsson M, Svensson L (1999) Rotavirus G-type restriction, persistence, and herd type specificity in Swedish cattle herds. Clin Diagn Lab Immunol 6: 181-185.

1112. de Verdier Klingenberg K, Svensson L (1998) Group A rotavirus as a cause of neonatal calf enteritis in Sweden. Acta Vet Scand 39: 195-199.

1113. de Verdier Klingenberg K, Vagsholm I, Alenius S (1999) Incidence of diarrhea among calves after strict closure and eradication of bovine viral diarrhea virus infection in a dairy herd. J Am Vet Med Assoc 214: 1824-1828.

1114. de Villiers FP, Steele AD, Driessen M (2003) Central nervous system involvement in neonatal rotavirus infection. Ann Trop Paediatr 23: 309-312.

1115. de Visser NA, Breukink HJ, van Zijderveld FG, de Leeuw PW (1987) Enteric infections in veal calves: a longitudinal study on four veal calf units. Vet Q 9: 289-296.

1116. De Vos B, Delem A, Hardt K, Bock HL, Meurice F, et al. (2006) A short report on clinical evaluation of RIX4414: highlights of world-wide development. Vaccine 24: 3777-3778.

1117. De Vos B, Vesikari T, Linhares AC, Salinas B, Perez-Schael I, et al. (2004) A rotavirus vaccine for prophylaxis of infants against rotavirus gastroenteritis. Pediatr Infect Dis J 23: S179-182.

1118. de Vrese M, Schrezenmeir J (2008) Probiotics, prebiotics, and synbiotics. Adv Biochem Eng Biotechnol 111: 1-66.

1119. de Vries WP, Houben AW, Stobberingh EE (1986) Comparison of four commercial assays for detection of rotavirus in childhood gastroenteritis. Eur J Clin Microbiol 5: 542-544.

1120. de Wit MA, Koopmans MP, Kortbeek LM, van Leeuwen NJ, Bartelds AI, et al. (2001) Gastroenteritis in sentinel general practices,The Netherlands. Emerg Infect Dis 7: 82-91.

1121. de Wit MA, Koopmans MP, Kortbeek LM, van Leeuwen NJ, Vinje J, et al. (2001) Etiology of gastroenteritis in sentinel general practices in the netherlands. Clin Infect Dis 33: 280-288.

1122. de Wit MA, Koopmans MP, van der Blij JF, van Duynhoven YT (2000) Hospital admissions for rotavirus infection in the Netherlands. Clin Infect Dis 31: 698-704.

1123. de Wit MA, Koopmans MP, van Duynhoven YT (2003) Risk factors for norovirus, Sapporo-like virus, and group A rotavirus gastroenteritis. Emerg Infect Dis 9: 1563-1570.

1124. de Zoysa I, Feachem RG (1985) Interventions for the control of diarrhoeal diseases among young children: rotavirus and cholera immunization. Bull World Health Organ 63: 569-583.

1125. Dea S, Archambault D, Elazhary MA, Roy RS (1986) Genomic variations and antigenic relationships among cytopathic rotavirus strains isolated in Quebec dairy herds. Can J Vet Res 50: 126-129.

1126. Dea S, Begin ME, Archambault D, Elazhary MA, Roy RS (1985) Distinct rotaviruses isolated from asymptomatic calves. Cornell Vet 75: 307-318.

1127. Dea S, Elazhary MA, Roy RS (1986) Distinct serotypes of porcine rotavirus associated with diarrhea in suckling piglets in southern Quebec. Can J Vet Res 50: 130-132.

1128. Dea S, Tijssen P (1988) Viral agents associated with outbreaks of diarrhea in turkey flocks in Quebec. Can J Vet Res 52: 53-57.

1129. Dearlove J, Latham P, Dearlove B, Pearl K, Thomson A, et al. (1983) Clinical range of neonatal rotavirus gastroenteritis. Br Med J (Clin Res Ed) 286: 1473-1475.

1130. Debouck P, Pensaert M (1979) Experimental infection of pigs with Belgian isolates of the porcine rotavirus. Zentralbl Veterinarmed B 26: 517-526.

1131. Debouck P, Pensaert M (1983) Rotavirus excretion in suckling pigs followed under field circumstances. Ann Rech Vet 14: 447-448.

1132. Decaluwe H, Harrison LM, Mariscalco MM, Gendrel D, Bohuon C, et al. (2006) Procalcitonin in children with Escherichia coli O157:H7 associated hemolytic uremic syndrome. Pediatr Res 59: 579-583.

1133. Decre D, Barbut F, Petit JC (2000) [Role of the microbiology laboratory in the diagnosis of nosocomial diarrhea]. Pathol Biol (Paris) 48: 733-744.

1134. Dector MA, Romero P, Lopez S, Arias CF (2002) Rotavirus gene silencing by small interfering RNAs. EMBO Rep 3: 1175-1180.

1135. Deen JL (2003) Evolution in the recommendations for oral rehydration therapy according to World Health Organisation guidelines: where to go from here? J Indian Med Assoc 101: 366-368, 370.

1136. Deepa R, Durga Rao C, Suguna K (2007) Structure of the extended diarrhea-inducing domain of rotavirus enterotoxigenic protein NSP4. Arch Virol 152: 847-859.

1137. Deepa R, Jagannath MR, Kesavulu MM, Durga Rao C, Suguna K (2004) Expression, purification, crystallization and preliminary crystallographic analysis of the diarrhoea-causing and virulence-determining region of rotaviral nonstructural protein NSP4. Acta Crystallogr D Biol Crystallogr 60: 135-136.

1138. Dei R, Urbano P, Franceschini E, Bartolozzi G, De Mayo E (1980) [Serodiagnosis of rotavirus infections in gastroenteritis of infants]. Boll Ist Sieroter Milan 59: 159-165.

1139. Dekker J, Tytgat KM (1993) Binding of rotavirus by a 46 kD milk-glycoprotein may prevent gastroenteritis. J Pediatr Gastroenterol Nutr 17: 228-230.

1140. del Castillo Martin F (1992) [The principal enteropathogens in infantile diarrhea in Spain]. Med Clin (Barc) 99: 69-74.

1141. del Refugio Gonzalez-Losa M, Polanco-Marin GG, Manzano-Cabrera L, Puerto-Solis M (2001) Acute gastroenteritis associated with rotavirus in adults. Arch Med Res 32: 164-167.

1142. del RG-LM, Rodriguez-Angulo E, Manzano-Cabrera L, Mejia-Camara J, Puerto-Solis M (2005) Detection of unusual strains of RV in patients with acute diarrhoea in Mexico. J Clin Virol 32: 325-328.

1143. Delage G, McLaughlin B, Berthiaume L (1978) A clinical study of rotavirus gastroenteritis. J Pediatr 93: 455-457.

1144. Delem A, Berge E, Brucher JM, Lobmann M, Zygraich N (1985) The neurovirulence of human and animal rotaviruses in cercopithecus monkeys. J Biol Stand 13: 107-114.

1145. Delem A, Lobmann M, Zygraich N (1984) A bovine rotavirus developed as a candidate vaccine for use in humans. J Biol Stand 12: 443-445.

1146. Dellert SF, Cohen MB (1994) Diarrheal disease. Established pathogens, new pathogens, and progress in vaccine development. Gastroenterol Clin North Am 23: 637-654.

1147. Delmas O, Breton M, Sapin C, Le Bivic A, Colard O, et al. (2007) Heterogeneity of Raft-type membrane microdomains associated with VP4, the rotavirus spike protein, in Caco-2 and MA 104 cells. J Virol 81: 1610-1618.

1148. Delpiano ML, Riquelme RJ, Casado FM, Alvarez HX (2006) [Clinical features and costs of rotavirus gastroenteritis in infants: community versus nosocomialy acquired infection]. Rev Chilena Infectol 23: 35-42.

1149. Deng FM (1985) [Epidemiology of rota-virus gastro-enteritis]. Zhonghua Liu Xing Bing Xue Za Zhi 6: 251-253.

1150. Deng L, Jia LY, Chen DM, Zhang Y, Qian Y (2007) [Clinical manifestations of norovirus gastroenteritis in infants and children]. Zhonghua Liu Xing Bing Xue Za Zhi 28: 676-678.

1151. Denis F, Barriere E, Venot C, Ranger-Rogez S, Durepaire N, et al. (1997) [Virus and gastrointestinal infections]. Ann Biol Clin (Paris) 55: 275-287.

1152. Dennehy PH (2000) Transmission of rotavirus and other enteric pathogens in the home. Pediatr Infect Dis J 19: S103-105.

1153. Dennehy PH (2005) Rotavirus vaccines: an update. Curr Opin Pediatr 17: 88-92.

1154. Dennehy PH (2006) A short report on the highlights of world-wide development of RIX4414: a North American experience comparative evaluation of safety and immunogenicity of two dosages of an oral live attenuated human rotavirus vaccine (RIX4414) in infants in the United States and Canada. Vaccine 24: 3780-3781.

1155. Dennehy PH (2007) Rotavirus vaccines--an update. Vaccine 25: 3137-3141.

1156. Dennehy PH (2008) Rotavirus vaccines: an overview. Clin Microbiol Rev 21: 198-208.

1157. Dennehy PH, Brady RC, Halperin SA, Ward RL, Alvey JC, et al. (2005) Comparative evaluation of safety and immunogenicity of two dosages of an oral live attenuated human rotavirus vaccine. Pediatr Infect Dis J 24: 481-488.

1158. Dennehy PH, Cortese MM, Begue RE, Jaeger JL, Roberts NE, et al. (2006) A case-control study to determine risk factors for hospitalization for rotavirus gastroenteritis in U.S. children. Pediatr Infect Dis J 25: 1123-1131.

1159. Dennehy PH, Gauntlett DR (1988) Evaluation of a new enzyme immunoassay (TESTPACK rotavirus) for the detection of rotavirus in fecal specimens. Diagn Microbiol Infect Dis 11: 201-203.

1160. Dennehy PH, Gauntlett DR, Spangenberger SE (1990) Choice of reference assay for the detection of rotavirus in fecal specimens: electron microscopy versus enzyme immunoassay. J Clin Microbiol 28: 1280-1283.

1161. Dennehy PH, Gauntlett DR, Tente WE (1988) Comparison of nine commercial immunoassays for the detection of rotavirus in fecal specimens. J Clin Microbiol 26: 1630-1634.

1162. Dennehy PH, Hartin M, Nelson SM, Reising SF (1999) Evaluation of the ImmunoCardSTAT! rotavirus assay for detection of group A rotavirus in fecal specimens. J Clin Microbiol 37: 1977-1979.

1163. Dennehy PH, Nelson SM, Spangenberger S, Noel JS, Monroe SS, et al. (2001) A prospective case-control study of the role of astrovirus in acute diarrhea among hospitalized young children. J Infect Dis 184: 10-15.

1164. Dennehy PH, Peter G (1985) Risk factors associated with nosocomial rotavirus infection. Am J Dis Child 139: 935-939.

1165. Dennehy PH, Rodgers GC, Jr., Ward RL, Markwick AJ, Mack M, et al. (1996) Comparative evaluation of reactogenicity and immunogenicity of two dosages of oral tetravalent rhesus rotavirus vaccine. US Rhesus Rotavirus Vaccine Study Group. Pediatr Infect Dis J 15: 1012-1018.

1166. Dennehy PH, Schutzbank TE, Thorne GM (1994) Evaluation of an automated immunodiagnostic assay, VIDAS Rotavirus, for detection of rotavirus in fecal specimens. J Clin Microbiol 32: 825-827.

1167. Dennehy PH, Tente WE, Fisher DJ, Veloudis BA, Peter G (1989) Lack of impact of rapid identification of rotavirus-infected patients on nosocomial rotavirus infections. Pediatr Infect Dis J 8: 290-296.

1168. Dennin RH (1978) [Rotavirus as a causative agent of infantile gastroenteritis. Diagnosis and epidemiology (author's transl)]. Immun Infekt 6: 118-122.

1169. Denno DM, Stapp JR, Boster DR, Qin X, Clausen CR, et al. (2005) Etiology of diarrhea in pediatric outpatient settings. Pediatr Infect Dis J 24: 142-148.

1170. Deo RC, Groft CM, Rajashankar KR, Burley SK (2002) Recognition of the rotavirus mRNA 3' consensus by an asymmetric NSP3 homodimer. Cell 108: 71-81.

1171. Deorari AK, Bhan MK, Arora NK, Ghai OP, Kumar R, et al. (1982) Stool electrolyte composition in relation to etiology in acute gastroenteritis. Indian Pediatr 19: 217-220.

1172. Derbyshire JB (1989) The interferon sensitivity of selected porcine viruses. Can J Vet Res 53: 52-55.

1173. Derbyshire JB, Woode GN (1978) Classification of rotaviruses: report from the World Health Organization/Food and Agriculture Organization Comparative Virology Program. J Am Vet Med Assoc 173: 519-521.

1174. Desai HS, Banker DD (1993) Rotavirus infection among children in Bombay. Indian J Med Sci 47: 27-33.

1175. Desenclos JC, Rebiere I, Letrillard L, Flahault A, Hubert B (1999) Diarrhoea-related morbidity and rotavirus infection in France. Acta Paediatr Suppl 88: 42-47.

1176. Desgrandchamps D, Munzinger J (1989) [Infectious gastroenteritis in the immunocompetent child. Significance of Cryptosporidium spp. and Aeromonas ssp.]. Schweiz Med Wochenschr 119: 276-281.

1177. Desikan P, Daniel JD, Kamalarathnam CN, Mathan MM (1996) Molecular epidemiology of nosocomial rotavirus infection. J Diarrhoeal Dis Res 14: 12-15.

1178. Desselberger U (1988) [Molecular epidemiology of rotaviruses]. Immun Infekt 16: 182-188.

1179. Desselberger U (1997) Viral factors determining rotavirus pathogenicity. Arch Virol Suppl 13: 131-139.

1180. Desselberger U (1998) Prospects for vaccines against rotaviruses. Rev Med Virol 8: 43-52.

1181. Desselberger U (1998) Viral gastroenteritis. Curr Opin Infect Dis 11: 565-575.

1182. Desselberger U (2000) Gastroenteritis viruses: research update and perspectives. Gastroenteritis viruses, Novartis Foundation Symposium 238, London, UK, 16-18 May 2000. Mol Med Today 6: 383-384.

1183. Desselberger U (2005) RotaTeq (sanofi pasteur/Wistar Institute/Children's Hospital of Philadelphia). Curr Opin Investig Drugs 6: 199-208.

1184. Desselberger U, Hung T, Follett EA (1986) Genome analysis of human rotaviruses by oligonucleotide mapping of isolated RNA segments. Virus Res 4: 357-368.

1185. Devulapalli CS (2000) Rotavirus gastroenteritis possibly causing reye syndrome. Acta Paediatr 89: 613-614.

1186. Dewan N, Faruque AS, Fuchs GJ (1998) Nutritional status and diarrhoeal pathogen in hospitalized children in Bangladesh. Acta Paediatr 87: 627-630.

1187. Dewey C, Carman S, Pasma T, Josephson G, McEwen B (2003) Relationship between group A porcine rotavirus and management practices in swine herds in Ontario. Can Vet J 44: 649-653.

1188. Dewilde A, Krembel C, Roussel M, Duriez D, Fruchart A, et al. (1983) [The viral incidence in childhood diarrhea]. Ann Pediatr (Paris) 30: 422-426.

1189. DeWitt TG (1989) Acute diarrhea in children. Pediatr Rev 11: 6-13.

1190. Dhama K, Chauhan RS, Mahendran M, Malik SV (2009) Rotavirus diarrhea in bovines and other domestic animals. Vet Res Commun 33: 1-23.

1191. Dharakul T, Rott L, Greenberg HB (1990) Recovery from chronic rotavirus infection in mice with severe combined immunodeficiency: virus clearance mediated by adoptive transfer of immune CD8+ T lymphocytes. J Virol 64: 4375-4382.

1192. Di Lernia V, Lombardi M, Lo Scocco G (2004) Infantile acute hemorrhagic edema and rotavirus infection. Pediatr Dermatol 21: 548-550.

1193. Di Lernia V, Ricci C (2006) Skin manifestations with Rotavirus infections. Int J Dermatol 45: 759-761.

1194. Di Matteo A, Sarasini A, Scotta MS, Parea M, Licardi G, et al. (1989) Nosocomial outbreak of infant rotavirus diarrhea due to the appearance of a new serotype 4 strain. J Med Virol 27: 100-104.

1195. di Somma C, Fiore L, Di Lonardo A, Ridolfi B, Garzillo C, et al. (2003) Cross-reactivity between the major Parietaria allergen and rotavirus VP4 protein. Allergy 58: 503-510.

1196. Diamanti E, Superti F, Tinari A, Marziano ML, Giovannangeli S, et al. (1996) An epidemiological study on viral infantile diarrhoea in Tirana. New Microbiol 19: 9-14.

1197. Diaz J, Garcia-Martos P, Chozas N (1990) [Etiological research on acute gastroenteritis in the city of Cadiz]. Aten Primaria 7: 498-501.

1198. Dick W, Braun OH, Nagel W (1985) [Lysozyme in children with acute and chronic inflammatory intestinal diseases]. Padiatr Padol 20: 143-150.

1199. Dickman KG, Hempson SJ, Anderson J, Lippe S, Zhao L, et al. (2000) Rotavirus alters paracellular permeability and energy metabolism in Caco-2 cells. Am J Physiol Gastrointest Liver Physiol 279: G757-766.

1200. Dickson J, Smith VW, Coackley W, McKean P, Adams PS (1979) Rotavirus infection of foals. Aust Vet J 55: 207-208.

1201. Diez-Domingo J, Martin IO, Sanz AB, Lopez AG, Martinez CC, et al. (2006) Rotavirus gastroenteritis among children under five years of age in Valencia, Spain. Pediatr Infect Dis J 25: 455-457.

1202. Difazio MP, Braun L, Freedman S, Hickey P (2007) Rotavirus-induced seizures in childhood. J Child Neurol 22: 1367-1370.

1203. Diggle L (2007) Rotavirus diarrhoea and future prospects for prevention. Br J Nurs 16: 970-974.

1204. DiGiacomo RF, Thouless ME (1986) Epidemiology of naturally occurring rotavirus infection in rabbits. Lab Anim Sci 36: 153-156.

1205. Dimitriadis A, Bruggink LD, Marshall JA (2006) Evaluation of the Dako IDEIA norovirus EIA assay for detection of norovirus using faecal specimens from Australian gastroenteritis outbreaks. Pathology 38: 157-165.

1206. Dimitriadis A, Marshall JA (2005) Evaluation of a commercial enzyme immunoassay for detection of norovirus in outbreak specimens. Eur J Clin Microbiol Infect Dis 24: 615-618.

1207. Dimitrov DH, Estes MK, Rangelova SM, Shindarov LM, Melnick JL, et al. (1983) Detection of antigenically distinct rotaviruses from infants. Infect Immun 41: 523-526.

1208. Dimitrov DH, Graham DY, Lopez J, Muchinik G, Velasco G, et al. (1984) RNA electropherotypes of human rotaviruses from North and South America. Bull World Health Organ 62: 321-329.

1209. Dimitrov DH, Shindarov LM, Rangelova S (1986) Occurrence of antigenically distinct rotaviruses in infants in Bulgaria. Eur J Clin Microbiol 5: 471-473.

1210. Dirksen G, Bachmann PA (1977) [Occurrence of rota- and coronavirus as a cause of neonatal calf diarrhea in the Federal Republic of Germany]. Berl Munch Tierarztl Wochenschr 90: 475-477.

1211. DiStefano DJ, Kraiouchkine N, Mallette L, Maliga M, Kulnis G, et al. (2005) Novel rotavirus VP7 typing assay using a one-step reverse transcriptase PCR protocol and product sequencing and utility of the assay for epidemiological studies and strain characterization, including serotype subgroup analysis. J Clin Microbiol 43: 5876-5880.

1212. Divizia M, Gabrieli R, Donia D, Macaluso A, Bosch A, et al. (2004) Waterborne gastroenteritis outbreak in Albania. Water Sci Technol 50: 57-61.

1213. Diwakarla S, Clark R, Palombo EA (2002) Expanding distribution of human serotype G6 rotaviruses in Australia. Microbiol Immunol 46: 499-502.

1214. Djuretic T, Ramsay M, Gay N, Wall P, Ryan M, et al. (1999) An estimate of the proportion of diarrhoeal disease episodes seen by general practitioners attributable to rotavirus in children under 5 y of age in England and Wales. Acta Paediatr Suppl 88: 38-41.

1215. Doan LT, Okitsu S, Nishio O, Pham DT, Nguyen DH, et al. (2003) Epidemiological features of rotavirus infection among hospitalized children with gastroenteristis in Ho Chi Minh City, Vietnam. J Med Virol 69: 588-594.

1216. Doan TN, Nguyen VC (1986) Preliminary study on rotavirus diarrhoea in hospitalized children at Hanoi. J Diarrhoeal Dis Res 4: 81-82.

1217. Dodet B, Heseltine E, Mary C, Saliou P (1997) [Rotaviruses in human and veterinary medicine]. Sante 7: 195-199.

1218. Doit C, Mariani-Kurkdjian P, Bourrillon A, Bingen E (2007) [Rotavirus infections in a paediatric hospital during 5 years.]. Arch Pediatr 14: 1465-1467.

1219. Dolan KT, Twist EM, Horton-Slight P, Forrer C, Bell LM, Jr., et al. (1985) Epidemiology of rotavirus electropherotypes determined by a simplified diagnostic technique with RNA analysis. J Clin Microbiol 21: 753-758.

1220. Domingues AL, Morais AT, Cruz RL, Moreira LP, Gouvea VS (2008) Rotavirus-associated infantile diarrhea in Uberaba, Minas Gerais, on the wake of the Brazilian vaccination program. J Clin Virol 43: 298-301.

1221. Dominick HC (1979) [Gastroenteritis in children]. MMW Munch Med Wochenschr 121: 349-350.

1222. Dominick HC, Maass G (1979) [Rotavirus infections in children (author's transl)]. Klin Padiatr 191: 33-39.

1223. Donelli G, Rocchi G, Menichella D, Varveri A, Tangucci F, et al. (1982) [Epidemiological study of rotavirus infections in premature infants]. Ann Ist Super Sanita 18: 437-439.

1224. Donelli G, Ruggeri FM, Tinari A, Marziano ML, Menichella D, et al. (1988) A three-year diagnostic and epidemiological study on viral infantile diarrhoea in Rome. Epidemiol Infect 100: 311-320.

1225. Donelli G, Superti F (1994) The rotavirus genus. Comp Immunol Microbiol Infect Dis 17: 305-320.

1226. Donelli G, Superti F, Tinari A, Marziano ML, Caione D, et al. (1993) Viral childhood diarrhoea in Rome: a diagnostic and epidemiological study. New Microbiol 16: 215-225.

1227. Dong J, Tian M (1994) [Study of carbon agglutination test for the rapid diagnosis of rotavirus]. Wei Sheng Wu Xue Bao 34: 156-159.

1228. Dong JL, Liang BG, Jin YS, Zhang WJ, Wang T (2005) Oral immunization with pBsVP6-transgenic alfalfa protects mice against rotavirus infection. Virology 339: 153-163.

1229. Dong Y, Zeng CQ, Ball JM, Estes MK, Morris AP (1997) The rotavirus enterotoxin NSP4 mobilizes intracellular calcium in human intestinal cells by stimulating phospholipase C-mediated inositol 1,4,5-trisphosphate production. Proc Natl Acad Sci U S A 94: 3960-3965.

1230. Donovan SM, Zijlstra RT, Odle J (1994) Use of the piglet to study the role of growth factors in neonatal intestinal development. Endocr Regul 28: 153-162.

1231. Dormitzer PR, Both GW, Greenberg HB (1994) Presentation of neutralizing epitopes by engineered rotavirus VP7's expressed by recombinant vaccinia viruses. Virology 204: 391-402.

1232. Dormitzer PR, Greenberg HB, Harrison SC (2000) Purified recombinant rotavirus VP7 forms soluble, calcium-dependent trimers. Virology 277: 420-428.

1233. Dormitzer PR, Nason EB, Prasad BV, Harrison SC (2004) Structural rearrangements in the membrane penetration protein of a non-enveloped virus. Nature 430: 1053-1058.

1234. Dossetor JF, Chrystie IL, Totterdell BM (1979) Rotavirus gastro-enteritis in northern Nigeria. Trans R Soc Trop Med Hyg 73: 115-116.

1235. Dove W, Cunliffe NA, Gondwe JS, Broadhead RL, Molyneux ME, et al. (2005) Detection and characterization of human caliciviruses in hospitalized children with acute gastroenteritis in Blantyre, Malawi. J Med Virol 77: 522-527.

1236. Dowe G, King SD, Maitland PB, Swaby-Ellis DE (1988) Laboratory investigations on rotavirus in infantile gastroenteritis in Jamaica. Trans R Soc Trop Med Hyg 82: 155-159.

1237. Dowling JM, Wynne H (1981) Role of enteric adenoviruses and rotaviruses in infantile gastroenteritis. Lancet 2: 305-306.

1238. Dreesman J, Weber KA, Dallugge-Tamm H, Holscher J, Pulz M (2001) [Surveillance of gastroenteritic infections in lower Saxony: results and experiences from a regional public-health project over six years]. Gesundheitswesen 63: 763-768.

1239. Driesen SJ, Carland PG, Fahy VA (1993) Studies on preweaning piglet diarrhoea. Aust Vet J 70: 259-262.

1240. Drozdov SG (1981) [Rotavirus gastroenteritis]. Zh Mikrobiol Epidemiol Immunobiol: 7-16.

1241. Drozdov SG (1983) [Viral gastroenteritis]. Vestn Akad Med Nauk SSSR: 40-49.

1242. Drozdov SG, Shekoian LA, Korolev MB (1981) [Rotavirus gastroenteritis. Laboratory diagnosis and study methods]. Vopr Virusol: 644-649.

1243. Drozdov SG, Shekoian LA, Korolev MB, Andzhaparidze AG (1979) [Human rotavirus in cell culture: its isolation and passage]. Vopr Virusol: 389-392.

1244. Drozdov SG, Shekoian LA, Korolev MB, Nesterina LF (1979) [Virological and serological study of rotavirus gastroenteritis]. Vopr Virusol: 385-389.

1245. D'Souza RM, Hall G, Becker NG (2008) Climatic factors associated with hospitalizations for rotavirus diarrhoea in children under 5 years of age. Epidemiol Infect 136: 56-64.

1246. D'Souza RM, Hall G, Becker NG (2008) Climatic factors associated with hospitalizations for rotavirus diarrhoea in children under 5 years of age. Epidemiol Infect 136: 56-64.

1247. Duan ZJ, Li DD, Zhang Q, Liu N, Huang CP, et al. (2007) Novel human rotavirus of genotype G5P[6] identified in a stool specimen from a Chinese girl with diarrhea. J Clin Microbiol 45: 1614-1617.

1248. Duangmani C, Suvongse C, Echeverria P, Vanapruks V, Punyarachun P (1985) Vertical transmission of enteric pathogens at birth. Ann Trop Paediatr 5: 15-18.

1249. Dubey AP, Rajeshwari K, Chakravarty A, Famularo G (2008) Use of VSL[sharp]3 in the treatment of rotavirus diarrhea in children: preliminary results. J Clin Gastroenterol 42 Suppl 3 Pt 1: S126-129.

1250. Dubos F, Lorrot M, Soulier M, Rozenberg F, Lebon P, et al. (2004) [Interferon alpha production in the serum of very young infants after viral infections]. Med Mal Infect 34: 561-565.

1251. Dubourguier HC, Gouet P, Mandard O, Contrepois M, Bachelerie C (1978) Scanning electron microscopy of abomasium and intestine of gnotoxenic calves infected either with rotavirus, coronarivus or enteropathogenic Escherichia coli or with rotavirus and E. coli. Ann Rech Vet 9: 441-451.

1252. Duffau Toro G, Emilfork Soto M (1985) [Acute diarrhea syndrome. Rehydration and maintenance therapy using oral gluco-electrolyte formulas]. Bol Oficina Sanit Panam 98: 136-143.

1253. Duffy LC (2000) Interactions mediating bacterial translocation in the immature intestine. J Nutr 130: 432S-436S.

1254. Duffy LC, Byers TE, Riepenhoff-Talty M, La Scolea LJ, Zielezny M, et al. (1986) The effects of infant feeding on rotavirus-induced gastroenteritis: a prospective study. Am J Public Health 76: 259-263.

1255. Duffy LC, Riepenhoff-Talty M, Byers TE, La Scolea LJ, Zielezny MA, et al. (1986) Modulation of rotavirus enteritis during breast-feeding. Implications on alterations in the intestinal bacterial flora. Am J Dis Child 140: 1164-1168.

1256. Duffy LC, Zielezny MA, Riepenhoff-Talty M, Dryja D, Sayahtaheri-Altaie S, et al. (1994) Reduction of virus shedding by B. bifidum in experimentally induced MRV infection. Statistical application for ELISA. Dig Dis Sci 39: 2334-2340.

1257. Duffy LC, Zielezny MA, Riepenhoff-Talty M, Dryja D, Sayahtaheri-Altaie S, et al. (1993) Effectiveness of Bifidobacterium bifidum in experimentally induced MRV infection: dietary implications in formulas for newborns. Endocr Regul 27: 223-229.

1258. Duffy LC, Zielezny MA, Riepenhoff-Talty M, Dryja D, Sayahtaheri-Altaie S, et al. (1994) Effectiveness of Bifidobacterium bifidum in mediating the clinical course of murine rotavirus diarrhea. Pediatr Res 35: 690-695.

1259. Dufillot D, Courouble G, Nicolas M, Perez JM, Sans A, et al. (1999) [Causes of acute enteritis in hospitalized children in Guadeloupe]. Arch Pediatr 6: 1132-1133.

1260. Dupont C (1999) [Important factors in rotavirus infections]. Arch Pediatr 6 Suppl 2: 327s-329s.

1261. Dupont C (2000) [Bacterial flora in the infant and intestinal immunity: Implication and prospects for infant food with probiotics]. Arch Pediatr 7 Suppl 2: 252s-255s.

1262. DuPont HL (1978) Enteropathogenic organisms. New etiologic agents and concepts of disease. Med Clin North Am 62: 945-960.

1263. DuPont HL (1978) Interventions in diarrheas of infants and young children. J Am Vet Med Assoc 173: 649-653.

1264. DuPont HL (1984) Rotaviral gastroenteritis--some recent developments. J Infect Dis 149: 663-666.

1265. Dupuis P, Beby A, Bourgoin A, Lussier-Bonneau MD, Agius G (1995) [Epidemic of viral gastroenteritis in an elderly community]. Presse Med 24: 356-358.

1266. Durepaire N, Pradie MP, Ploy MC, Mounier M, Ranger-Rogez S, et al. (1995) [Adenoviruses from stool samples in hospital units. Comparison with main pathogens in gastroenteritis (rotavirus, Campylobacter, Salmonella)]. Pathol Biol (Paris) 43: 601-610.

1267. Durham PJ, Farquharson BC, Stevenson BJ (1979) Rotavirus and coronavirus associated diarrohoea in calves. N Z Vet J 27: 266, 271-262.

1268. Durham PJ, Hassard LE, Norman GR, Yemen RL (1989) Viruses and virus-like particles detected during examination of feces from calves and piglets with diarrhea. Can Vet J 30: 876-881.

1269. Durham PJ, Stevenson BJ, Farquharson BC (1979) Rotavirus and coronavirus associated diarrhoea in domestic animals. N Z Vet J 27: 30-32.

1270. Durigon EL, Candeias JA, Jerez JA, Bittencourt MJ, Ortolani EL (1991) Comparison of staphylococcal co-agglutination with other assays for rapid diagnosis of rotavirus infection in humans, calves and piglets. J Virol Methods 35: 73-79.

1271. Dus Santos MJ, Wigdorovitz A (2005) Transgenic plants for the production of veterinary vaccines. Immunol Cell Biol 83: 229-238.

1272. Dutta P (1994) Usefulness of ORT in certain special situations of diarrhoeal diseases. Indian J Public Health 38: 44-49.

1273. Dutta P, Bhattacharya SK, Saha MR, Dutta D, Bhattacharya MK, et al. (1992) Nosocomial rotavirus diarrhea in two medical wards of a pediatric hospital in Calcutta. Indian Pediatr 29: 701-706.

1274. Dutta P, Mitra U, Rasaily R, Bhattacharya SK, De SP, et al. (1993) Prospective study of nosocomial enteric infections in a pediatric hospital, Calcutta. Indian Pediatr 30: 187-194.

1275. Dutta SR, Khalfan SA, Baig BH, Philipose L, Fulayfil R (1990) Epidemiology of rotavirus diarrhoea in children under five years in Bahrain. Int J Epidemiol 19: 722-727.

1276. Dwyer RM (1993) Rotaviral diarrhea. Vet Clin North Am Equine Pract 9: 311-319.

1277. Dwyer RM (1995) Disinfecting equine facilities. Rev Sci Tech 14: 403-418.

1278. Dyall-Smith ML, Lazdins I, Tregear GW, Holmes IH (1986) Location of the major antigenic sites involved in rotavirus serotype-specific neutralization. Proc Natl Acad Sci U S A 83: 3465-3468.

1279. Ebina T (1996) Prophylaxis of rotavirus gastroenteritis using immunoglobulin. Arch Virol Suppl 12: 217-223.

1280. Ebina T, Ohta M, Kanamaru Y, Yamamoto-Osumi Y, Baba K (1992) Passive immunizations of suckling mice and infants with bovine colostrum containing antibodies to human rotavirus. J Med Virol 38: 117-123.

1281. Ebina T, Sato A, Umezu K, Ishida N, Ohyama S, et al. (1985) Prevention of rotavirus infection by oral administration of cow colostrum containing antihumanrotavirus antibody. Med Microbiol Immunol 174: 177-185.

1282. Ebina T, Tsukada K (1991) Protease inhibitors prevent the development of human rotavirus-induced diarrhea in suckling mice. Microbiol Immunol 35: 583-588.

1283. Ebina T, Tsukada K, Umezu K, Nose M, Tsuda K, et al. (1990) Gastroenteritis in suckling mice caused by human rotavirus can be prevented with egg yolk immunoglobulin (IgY) and treated with a protein-bound polysaccharide preparation (PSK). Microbiol Immunol 34: 617-629.

1284. Ebrahim GJ (2008) Rotaviruses and rotavirus vaccines. J Trop Pediatr 54: 79-82.

1285. Echeverria P, Blacklow NR, Cukor GG, Vibulbandhitkit S, Changchawalit S, et al. (1983) Rotavirus as a cause of severe gastroenteritis in adults. J Clin Microbiol 18: 663-667.

1286. Echeverria P, Blacklow NR, Sanford LB, Cukor GG (1981) Travelers' diarrhea among American Peace Corps volunteers in rural Thailand. J Infect Dis 143: 767-771.

1287. Echeverria P, Blacklow NR, Zipkin C, Vollet JJ, Olson JA, et al. (1979) Etiology of gastroenteritis among Americans living in the Philippines. Am J Epidemiol 109: 493-501.

1288. Echeverria P, Hoge CW, Bodhidatta L, Tungtaem C, Herrmann J, et al. (1994) Etiology of diarrhea in a rural community in western Thailand: importance of enteric viruses and enterovirulent Escherichia coli. J Infect Dis 169: 916-919.

1289. Echeverria P, Jackson LR, Hoge CW, Arness MK, Dunnavant GR, et al. (1993) Diarrhea in U.S. troops deployed to Thailand. J Clin Microbiol 31: 3351-3352.

1290. Echeverria P, Ramirez G, Blacklow NR, Ksiazek T, Cukor G, et al. (1979) Travelers' diarrhea among U.S. Army troops in South Korea. J Infect Dis 139: 215-219.

1291. Echeverria P, Taylor DN, Leksomboon U, Blacklow NR, Pinnoi S, et al. (1986) Identification of enteric pathogens in the small and large intestine of children with diarrhea. Diagn Microbiol Infect Dis 4: 277-284.

1292. Echeverria P, Taylor DN, Lexsomboon U, Bhaibulaya M, Blacklow NR, et al. (1989) Case-control study of endemic diarrheal disease in Thai children. J Infect Dis 159: 543-548.

1293. Eckmann L (2002) Small bowel infections. Curr Opin Gastroenterol 18: 197-202.

1294. Edelman R (1985) Prevention and treatment of infectious diarrhea. Speculations on the next 10 years. Am J Med 78: 99-106.

1295. Edelman R (1987) Perspective on the development and deployment of rotavirus vaccines. Pediatr Infect Dis J 6: 704-710.

1296. Edelman R, Levine MM (1980) Acute diarrheal infections in infants I. Bacterial and viral causes. Hosp Pract 15: 97-104.

1297. Edmonson LM, Ebbert JO, Evans JM (2000) Report of a rotavirus outbreak in an adult nursing home population. J Am Med Dir Assoc 1: 175-179.

1298. Edwards S, Chasey D, Napthine P, Banks J, Hewitt-Taylor C, et al. (1987) A comparison of three rapid diagnostic methods for the detection of rotavirus infection in calves. Vet Microbiol 13: 19-25.

1299. Eepejo RT, Calderon E, Gonzalez N, Salomon A, Martuscelli A, et al. (1978) Rotavirus gastroenteritis in hospitalized infants and young children in Mexico City. Rev Latinoam Microbiol 20: 239-246.

1300. Effler PV, Holman RC, Parashar UD, Glass RI (2000) Diarrhea-associated hospitalizations among children in Hawaii. Hawaii Med J 59: 362-365.

1301. Egashira M, Takayanagi T, Moriuchi M, Moriuchi H (2007) Does daily intake of bovine lactoferrin-containing products ameliorate rotaviral gastroenteritis? Acta Paediatr 96: 1242-1244.

1302. Eggers HJ (1985) [Viral diarrheas]. Zentralbl Bakteriol Mikrobiol Hyg [B] 180: 128-133.

1303. Eggers HJ (1986) [Nosocomial virus infections]. Zentralbl Bakteriol Mikrobiol Hyg [B] 183: 114-119.

1304. Eggers HJ (1990) [Rotavirus infections]. Z Gastroenterol Verh 25: 75-78.

1305. Eglee Perez M, Glass R, Alvarez G, Pericchi LR, Gonzalez R, et al. (2000) Rhesus rotavirus-based quadrivalent vaccine is efficacious despite age, socioeconomic conditions and seasonality in Venezuela. Vaccine 19: 976-981.

1306. Ehlken B, Laubereau B, Karmaus W, Petersen G, Rohwedder A, et al. (2002) Prospective population-based study on rotavirus disease in Germany. Acta Paediatr 91: 769-775.

1307. Ehrenkranz P, Lanata CF, Penny ME, Salazar-Lindo E, Glass RI (2001) Rotavirus diarrhea disease burden in Peru: the need for a rotavirus vaccine and its potential cost savings. Rev Panam Salud Publica 10: 240-248.

1308. Eichhorn W, Bachmann PA, Baljer G, Plank P, Schneider P (1983) Vaccination of cows with a combined rotavirus/enterotoxigenic "E. coli" K99 vaccine to protect newborn calves against diarrhoea. Dev Biol Stand 53: 237-243.

1309. Eichhorn W, Bachmann PA, Werhahn H, Jacobi R (1986) Occurrence and isolation in tissue culture of equine rotaviruses. Zentralbl Veterinarmed B 33: 155-159.

1310. Eiden J, Lederman HM, Vonderfecht S, Yolken R (1986) T-cell-deficient mice display normal recovery from experimental rotavirus infection. J Virol 57: 706-708.

1311. Eiden J, Losonsky GA, Johnson J, Yolken RH (1985) Rotavirus RNA variation during chronic infection of immunocompromised children. Pediatr Infect Dis 4: 632-637.

1312. Eiden J, Vonderfecht S, Theil K, Torres-Medina A, Yolken RH (1986) Genetic and antigenic relatedness of human and animal strains of antigenically distinct rotaviruses. J Infect Dis 154: 972-982.

1313. Eiden J, Vonderfecht S, Yolken RH (1985) Evidence that a novel rotavirus-like agent of rats can cause gastroenteritis in man. Lancet 2: 8-11.

1314. Eiden JJ, Firoozmand F, Sato S, Vonderfecht SL, Yin FZ, et al. (1989) Detection of group B rotavirus in fecal specimens by dot hybridization with a cloned cDNA probe. J Clin Microbiol 27: 422-426.

1315. Eiden JJ, Mouzinho A, Lindsay DA, Glass RI, Fang ZY, et al. (1994) Serum antibody response to recombinant major inner capsid protein following human infection with group B rotavirus. J Clin Microbiol 32: 1599-1603.

1316. Eiden JJ, Nataro J, Vonderfecht S, Petric M (1992) Molecular cloning, sequence analysis, in vitro expression, and immunoprecipitation of the major inner capsid protein of the IDIR strain of group B rotavirus (GBR). Virology 188: 580-589.

1317. Eiden JJ, Verleur DG, Vonderfecht SL, Yolken RH (1988) Duration and pattern of asymptomatic rotavirus shedding by hospitalized children. Pediatr Infect Dis J 7: 564-569.

1318. Eiden JJ, Wee SB, Vonderfecht SL (1992) In vitro transcription and translation of group B rotavirus strain IDIR gene 8 and immunoprecipitation by human sera. J Clin Microbiol 30: 440-443.

1319. Eiden JJ, Wilde J, Firoozmand F, Yolken R (1991) Detection of animal and human group B rotaviruses in fecal specimens by polymerase chain reaction. J Clin Microbiol 29: 539-543.

1320. Eiguchi Y, Yamagishi H, Fukusho A, Shimizu Y, Matumoto M (1987) Hemagglutination and hemagglutination-inhibition tests with porcine rotavirus. Kitasato Arch Exp Med 60: 167-172.

1321. Einerhand AW (1998) Rotavirus NSP4 acts as a viral enterotoxin to induce diarrhea and is a potential target for rotavirus vaccines. J Pediatr Gastroenterol Nutr 27: 123-124.

1322. Eiros Bouza JM, Luquero Alcalde FJ, Bachiller Luque MR, Castrodeza Sanz J, Ortiz de Lejarazu Leonardo R (2007) [Rotavirus gastroenteritis: analysis of disease burden]. An Pediatr (Barc) 66: 90-92.

1323. Eiros Bouza JM, Ortiz de Lejarazu R, Luquero Alcalde FJ, Bachiller Luque MR, Solis Sanchez P, et al. (2007) [Viral etiology of pediatric gastroenteritis. A 20-year time-series analysis]. An Pediatr (Barc) 66: 92-93.

1324. Eisenberg JN, Cevallos W, Ponce K, Levy K, Bates SJ, et al. (2006) Environmental change and infectious disease: how new roads affect the transmission of diarrheal pathogens in rural Ecuador. Proc Natl Acad Sci U S A 103: 19460-19465.

1325. Eisengart LJ, Chou PM, Iyer K, Cohran VC, Rajaram V (2008) Rotavirus Infection in Small Bowel Transplant: A Histologic Comparison with Acute Cellular Rejection. Pediatr Dev Pathol: 1.

1326. Ekern LJ, Schipper IA, McMahon KJ (1981) Neonatal bovine enteritis: detection of rotavirus by counterimmunoelectrophoresis and enzyme-linked immunosorbent assay. Can J Comp Med 45: 135-139.

1327. el Assouli SM, Banjar ZM, Mohammed KA, Zamakhchari FT (1992) Rotavirus infection in children in Saudi Arabia. Am J Trop Med Hyg 46: 272-277.

1328. el Assouli SM, Mohammed KA, Banjar ZM (1995) Human rotavirus genomic RNA electropherotypes in Jeddah, Saudi Arabia from 1988 to 1992. Ann Trop Paediatr 15: 45-53.

1329. Elad D, Brenner J, Markovitcs A, Shlomovitz S, Basan J, et al. (2002) Influence of diet on the shedding of Candida glabrata by experimentally infected preweaned calves. Vet J 164: 275-279.

1330. Elaraby I, el-Sharkawy S, Abbassy A, Hussein M (1992) A study on delayed hypersensitivity to rotavirus in infancy and childhood. Ann Trop Paediatr 12: 83-86.

1331. el-Assouli SM (1996) Inter-relationships among subgroups, serotypes, and electropherotypes of rotaviruses isolated from humans. J Diarrhoeal Dis Res 14: 201-206.

1332. elAssouli SM, Banjar ZM, Mohammed KA, Milaat WA, elAssouli MZ (1996) Genetic and antigenic analysis of human rotavirus prevalent in Al-Taif, Saudi Arabia. J Trop Pediatr 42: 211-219.

1333. El-Attar L, Dhaliwal W, Howard CR, Bridger JC (2001) Rotavirus cross-species pathogenicity: molecular characterization of a bovine rotavirus pathogenic for pigs. Virology 291: 172-182.

1334. El-Attar L, Dhaliwal W, Iturriza-Gomara M, Bridger JC (2002) Identification and molecular characterization of a bovine G3 rotavirus which causes age-independent diarrhea in cattle. J Clin Microbiol 40: 937-942.

1335. El-Hodhod MA, Nassar MF, Ezz El-Arab S, Ahmed EF (2008) Rotavirus fecal antigen retrieval in infantile intussusception. Eur J Clin Microbiol Infect Dis 27: 879-881.

1336. Elias MM (1977) Distribution and titres of rotavirus antibodies in different age groups. J Hyg (Lond) 79: 365-372.

1337. Elias MM (1977) Separation and infectivity of two particle types of human rotavirus. J Gen Virol 37: 191-194.

1338. Ellens DJ, de Leeuw PW (1977) Detection of infantile gastroenteritis virus (rotavirus) by ELISA. Lancet 1: 1363-1364.

1339. Ellens DJ, de Leeuw PW (1980) [Neonatal diarrhoea in calves (author's transl)]. Tijdschr Diergeneeskd 105: 644-649.

1340. Ellens DJ, de Leeuw PW, Straver PJ (1978) The detection of rotavirus specific antibody in colostrum and milk by ELISA. Ann Rech Vet 9: 337-342.

1341. Elliott EJ, Da Cunha Ferreira RM, Cameron D, Farthing MJ, Walker-Smith JA (1989) Evaluation of three oral rehydration solutions designed for use in developed communities. Aliment Pharmacol Ther 3: 233-243.

1342. Elliott EJ, Dalby-Payne JR (2004) 2. Acute infectious diarrhoea and dehydration in children. Med J Aust 181: 565-570.

1343. Ellis GR, Daniels E (1988) Comparison of direct electron microscopy and enzyme immunoassay for the detection of rotaviruses in calves, lambs, piglets and foals. Aust Vet J 65: 133-135.

1344. Ellis ME, Watson B, Mandal BK, Dunbar EM, Craske J, et al. (1984) Micro-organisms in gastroenteritis. Arch Dis Child 59: 848-855.

1345. El-Mekki A, Al-Nakib W, Sethi SK, El-Khalik DA, Al-Wuhaib M (1984) Pseudoreplica electron microscopy for the detection of rotavirus: comparison with high-speed centrifugation electron microscopy and ELISA. J Virol Methods 9: 79-85.

1346. El-Mohamady H, Abdel-Messih IA, Youssef FG, Said M, Farag H, et al. (2006) Enteric pathogens associated with diarrhea in children in Fayoum, Egypt. Diagn Microbiol Infect Dis 56: 1-5.

1347. el-Mougi M, Amer A, el-Abhar A, Hughes J, el-Shafie A (1989) Epidemiological and clinical features of rotavirus associated acute infantile diarrhoea in Cairo, Egypt. J Trop Pediatr 35: 230-233.

1348. Elschner M, Prudlo J, Hotzel H, Otto P, Sachse K (2002) Nested reverse transcriptase-polymerase chain reaction for the detection of group A rotaviruses. J Vet Med B Infect Dis Vet Public Health 49: 77-81.

1349. Elschner M, Schrader C, Hotzel H, Prudlo J, Sachse K, et al. (2005) Isolation and molecular characterisation of equine rotaviruses from Germany. Vet Microbiol 105: 123-129.

1350. el-Sheikh SM, el-Assouli SM (2001) Prevalence of viral, bacterial and parasitic enteropathogens among young children with acute diarrhoea in Jeddah, Saudi Arabia. J Health Popul Nutr 19: 25-30.

1351. Emilfork M, Duffau G, Avendano L (1982) [Rotavirus and prolonged diarrhea syndrome of infants]. Bol Med Hosp Infant Mex 39: 345-348.

1352. Endara P, Trueba G, Solberg OD, Bates SJ, Ponce K, et al. (2007) Symptomatic and subclinical infection with rotavirus P[8]G9, rural Ecuador. Emerg Infect Dis 13: 574-580.

1353. Enemark HL, Ahrens P, Bille-Hansen V, Heegaard PM, Vigre H, et al. (2003) Cryptosporidium parvum: infectivity and pathogenicity of the 'porcine' genotype. Parasitology 126: 407-416.

1354. Enemark HL, Bille-Hansen V, Lind P, Heegaard PM, Vigre H, et al. (2003) Pathogenicity of Cryptosporidium parvum--evaluation of an animal infection model. Vet Parasitol 113: 35-57.

1355. England JJ, Poston RP (1980) Electron microscopic identification and subsequent isolation of a rotavirus from a dog with fatal neonatal diarrhea. Am J Vet Res 41: 782-783.

1356. Engleberg NC, Holburt EN, Barrett TJ, Gary GW, Jr., Trujillo MH, et al. (1982) Epidemiology of diarrhea due to rotavirus on an Indian reservation: risk factors in the home environment. J Infect Dis 145: 894-898.

1357. Ennever FK, Paskett ED (1994) Re: "Child care increases the risk of clinic visits for acute diarrhea and diarrhea due to rotavirus". Am J Epidemiol 139: 542.

1358. Enouf V, Langella P, Commissaire J, Cohen J, Corthier G (2001) Bovine rotavirus nonstructural protein 4 produced by Lactococcus lactis is antigenic and immunogenic. Appl Environ Microbiol 67: 1423-1428.

1359. Erhard MH, Bergmann J, Renner M, Hofmann A, Heinritzi K (1996) [Prophylactic effect of specific egg yolk antibodies in diarrhea caused by Escherichia coli K88 (F4) in weaned piglets]. Zentralbl Veterinarmed A 43: 217-223.

1360. Erhard MH, Kellner J, Eichelberger J, Losch U (1993) [New possibilities in oral immunoprophylaxis of newborn diarrhea in calves--a field study using specific egg antibodies]. Berl Munch Tierarztl Wochenschr 106: 383-387.

1361. Eriksson K, Holmgren J (2002) Recent advances in mucosal vaccines and adjuvants. Curr Opin Immunol 14: 666-672.

1362. Escarzaga E, Castro Munoz E, Valdivieso R, Ceronio Rosas B, Salinas-Vazquez J (1984) [Rotavirus infection in children of 2 cities of the state of Chiapas, Mexico]. Rev Latinoam Microbiol 26: 39-45.

1363. Escobar Castro H, Perdomo Giraldi M, Tamariz-Martel Moreno A, Suarez Cortina L (1988) [Oral vaccination with live attenuated rotavirus]. An Esp Pediatr 28: 527-529.

1364. Esona MD, Armah GE, Geyer A, Steele AD (2004) Detection of an unusual human rotavirus strain with G5P[8] specificity in a Cameroonian child with diarrhea. J Clin Microbiol 42: 441-444.

1365. Esona MD, Armah GE, Steele AD (2003) Molecular epidemiology of rotavirus infection in Western Cameroon. J Trop Pediatr 49: 160-163.

1366. Esona MD, Geyer A, Banyai K, Page N, Aminu M, et al. (2009) Novel human rotavirus genotype G5P[7] from child with diarrhea, Cameroon. Emerg Infect Dis 15: 83-86.

1367. Esona MD, Humphrey CD, Dennehy PH, Jiang B (2008) Prevalence of group C rotavirus among children in Rhode Island, United States. J Clin Virol 42: 221-224.

1368. Espejo R, Martinez E, Lopez S, Munoz O (1980) Different polypeptide composition of two human rotavirus types. Infect Immun 28: 230-237.

1369. Espejo R, Romero P, Calderon E, Gonzalez N (1978) [Diagnosis of Rotavirus using viral RNA electrophoresis]. Bol Med Hosp Infant Mex 35: 323-331.

1370. Espejo R, Romero P, Calderon E, Gonzalez N (1978) [Existence of 2 types of Rotavirus associated with acute gastroenteritis in children]. Bol Med Hosp Infant Mex 35: 217-222.

1371. Espejo RT, Avendano LF, Munoz O, Romero P, Eternod JG, et al. (1980) Comparison of human rotaviruses isolated in Mexico City and in Santiago, Chile, by electrophoretic migration of their double-stranded ribonucleic acid genome segments. Infect Immun 30: 342-348.

1372. Espejo RT, Calderon E, Gonzalez N, Salomon A, Martuscelli A, et al. (1979) Presence of two distinct types of rotavirus in infants and young children hospitalized with acute gastroenteritis in Mexico City, 1977. J Infect Dis 139: 474-477.

1373. Espejo RT, Munoz O, Serafin F, Romero P (1980) Shift in the prevalent human rotavirus detected by ribonucleic acid segment differences. Infect Immun 27: 351-354.

1374. Espejo RT, Puerto F, Soler C, Gonzalez N (1984) Characterization of a human pararotavirus. Infect Immun 44: 112-116.

1375. Espinosa Larios EL, Ruiz-Gomez J (1981) [Persistence of transplacental antibodies against rotavirus in children less than 6 months of age]. Bol Med Hosp Infant Mex 38: 595-598.

1376. Espinosa-Larios EL, Colorado-Dominguez J, Padilla-Fierro R, Cetina-Sauri G, Duran-Linan G, et al. (1983) [Frequency of acute infectious gastroenteritis caused by rotavirus in children of various populations of the Mexican Republic]. Bol Med Hosp Infant Mex 40: 188-191.

1377. Espinoza F, Bucardo F, Paniagua M, Svensson L, Hallander HO, et al. (2006) Shifts of rotavirus g and p types in Nicaragua--2001-2003. Pediatr Infect Dis J 25: 1078-1080.

1378. Espinoza F, Paniagua M, Hallander H, Hedlund KO, Svensson L (1997) Prevalence and characteristics of severe rotavirus infections in Nicaraguan children. Ann Trop Paediatr 17: 25-32.

1379. Espul C, Cuello H, Navarta LM, Mamani N, O'Ryan M, et al. (1993) [Characterization of antigenic types of circulating rotaviruses in Mendoza, Argentina based on typing of the external VP7 capsid protein]. Acta Gastroenterol Latinoam 23: 211-216.

1380. Espul C, Martinez N, Noel JS, Cuello H, Abrile C, et al. (2004) Prevalence and characterization of astroviruses in Argentinean children with acute gastroenteritis. J Med Virol 72: 75-82.

1381. Esquivel Rivero M, de la Cruz Castillo F, Guzman Tirado MG, Vazquez Ramudo S (1989) [Rotavirus infection in children with acute diarrhea in Habana City]. Rev Cubana Med Trop 41: 435-442.

1382. Essers B, Burnens AP, Lanfranchini FM, Somaruga SG, von Vigier RO, et al. (2000) Acute community-acquired diarrhea requiring hospital admission in Swiss children. Clin Infect Dis 31: 192-196.

1383. Estes MK, Ball JM, Crawford SE, O'Neal C, Opekun AA, et al. (1997) Virus-like particle vaccines for mucosal immunization. Adv Exp Med Biol 412: 387-395.

1384. Estes MK, Conner ME, Gilger MA, Graham DY (1989) Molecular biology and immunology of rotavirus infections. Immunol Invest 18: 571-581.

1385. Estes MK, Crawford SE, Penaranda ME, Petrie BL, Burns JW, et al. (1987) Synthesis and immunogenicity of the rotavirus major capsid antigen using a baculovirus expression system. J Virol 61: 1488-1494.

1386. Estes MK, Graham DY (1979) Epidemic viral gastroenteritis. Am J Med 66: 1001-1007.

1387. Estes MK, Graham DY (1985) Rotavirus antigens. Adv Exp Med Biol 185: 201-214.

1388. Estes MK, Graham DY, Dimitrov DH (1984) The molecular epidemiology of rotavirus gastroenteritis. Prog Med Virol 29: 1-22.

1389. Estes MK, Kang G, Zeng CQ, Crawford SE, Ciarlet M (2001) Pathogenesis of rotavirus gastroenteritis. Novartis Found Symp 238: 82-96; discussion 96-100.

1390. Estes MK, Morris AP (1999) A viral enterotoxin. A new mechanism of virus-induced pathogenesis. Adv Exp Med Biol 473: 73-82.

1391. Estevez Touzard M, Diaz Gonzalez M, Monte Boada RJ, Toledo Rodriguez I, Ramon Bravo J (1993) [The infectious etiology of acute diarrheal diseases in the Republic of Cuba, 1991]. Rev Cubana Med Trop 45: 139-145.

1392. Eugster AK, Sidwa T (1979) Rotaviruses in diarrheic feces of a dog. Vet Med Small Anim Clin 74: 817-819.

1393. Eugster AK, Whitford HW, Mehr LE (1978) Concurrent rotavirus and Salmonella infections in foals. J Am Vet Med Assoc 173: 857-858.

1394. Evans DG, Olarte J, DuPont HL, Evans DJ, Jr., Galindo E, et al. (1977) Enteropathogens associated with pediatric diarrhea in Mexico City. J Pediatr 91: 65-68.

1395. Evans MG, Waxler GL, Newman JP (1986) Prevalence of K88, K99, and 987P pili of Escherichia coli in neonatal pigs with enteric colibacillosis. Am J Vet Res 47: 2431-2434.

1396. Evans RH (1984) Rotavirus-associated diarrhea in young raccoons (Procyon lotor), striped skunks (Mephitis mephitis) and red foxes (Vulpes vulpes). J Wildl Dis 20: 79-85.

1397. Evermann JF, McKeirnan AJ, Smith AW, Skilling DE, Ott RL (1985) Isolation and identification of caliciviruses from dogs with enteric infections. Am J Vet Res 46: 218-220.

1398. Eyanga E, Jetteur P, Thiry E, Wellemans G, Dubuisson J, et al. (1989) [Research on antibodies against BHV-1, BHV-2, BHV-4, BVD-MD virus, bovine adenovirus A and B, rotavirus and coronavirus in cattle in western Zaire: complementary results]. Rev Elev Med Vet Pays Trop 42: 155-161.

1399. Fabiana A, Donia D, Gabrieli R, Petrinca AR, Cenko F, et al. (2007) Influence of enteric viruses on gastroenteritis in Albania: epidemiological and molecular analysis. J Med Virol 79: 1844-1849.

1400. Fagbami AH, Johnson OA, David-West TS (1985) Rotavirus infection in children presenting with acute gastroenteritis Ibadan, Nigeria. Trans R Soc Trop Med Hyg 79: 114-115.

1401. Fagbami AH, Oyejide CO, Enahoro F (1987) Neonatal rotavirus infection in urban and rural communities in Nigeria. Trop Geogr Med 39: 341-344.

1402. Fagundes-Neto U, de Andrade JA (1999) Acute diarrhea and malnutrition: lethality risk in hospitalized infants. J Am Coll Nutr 18: 303-308.

1403. Fahey KJ, Snodgrass DR, Campbell I, Dawson AM, Burrells C (1981) IgG1 antibody in milk protects lambs against rotavirus diarrhoea. Vet Immunol Immunopathol 2: 27-33.

1404. Fairchild PG, Blacklow NR (1988) Viral diarrhea. Infect Dis Clin North Am 2: 677-684.

1405. Falcone E, Tarantino M, Di Trani L, Cordioli P, Lavazza A, et al. (1999) Determination of bovine rotavirus G and P serotypes in italy by PCR. J Clin Microbiol 37: 3879-3882.

1406. Fall M, Sarr M, Signate-Sy H, Sow HD, Ould Cheikh A, et al. (1989) [Epidemiological study of diarrhea in children 0-5 yr of age]. Dakar Med 34: 166-171.

1407. Fang ZY, Deng SS, Wang CA, Ye WW, Chen GM, et al. (1985) [Analysis of the genome of rotavirus from the feces of adult infectious diarrhea]. Zhongguo Yi Xue Ke Xue Yuan Xue Bao 7: 93-96.

1408. Fang ZY, Glass RI, Penaranda M, Dong H, Monroe SS, et al. (1989) Purification and characterization of adult diarrhea rotavirus: identification of viral structural proteins. J Virol 63: 2191-2197.

1409. Fang ZY, Monroe SS, Dong H, Penaranda M, Wen L, et al. (1992) Coding assignments of the genome of adult diarrhea rotavirus. Arch Virol 125: 53-69.

1410. Fang ZY, Sun YP, Ye XH, Wang H, Zhang Q, et al. (2006) [Astrovirus infection among hospitalized children with acute diarrhea in seven regions of China, 1998-2005]. Zhonghua Liu Xing Bing Xue Za Zhi 27: 673-676.

1411. Fang ZY, Wang B, Kilgore PE, Bresee JS, Zhang LJ, et al. (2005) Sentinel hospital surveillance for rotavirus diarrhea in the People's Republic of China, August 2001-July 2003. J Infect Dis 192 Suppl 1: S94-99.

1412. Fang ZY, Xie HP, Lv HX, Zhang Q, Duan ZJ, et al. (2007) [Investigation of human calicivirus (HuCV) diarrhea among infantile and young children in China, 1999--2005]. Bing Du Xue Bao 23: 9-15.

1413. Fang ZY, Yang H, Qi J, Zhang J, Sun LW, et al. (2002) Diversity of rotavirus strains among children with acute diarrhea in China: 1998-2000 surveillance study. J Clin Microbiol 40: 1875-1878.

1414. Fang ZY, Yang H, Zhang J, Li YF, Hou AC, et al. (2000) Child rotavirus infection in association with acute gastroenteritis in two Chinese sentinel hospitals. Pediatr Int 42: 401-405.

1415. Fang ZY, Ye Q, Ho MS, Dong H, Qing S, et al. (1989) Investigation of an outbreak of adult diarrhea rotavirus in China. J Infect Dis 160: 948-953.

1416. Farahtaj F, Gallimore CI, Iturriza-Gomara M, Taremi M, Zali MR, et al. (2007) Rotavirus VP7, VP4 and VP6 genotypes co-circulating in Tehran, Iran, between 2003 and 2004. Epidemiol Infect 135: 834-838.

1417. Farnworth ER (2008) The evidence to support health claims for probiotics. J Nutr 138: 1250S-1254S.

1418. Farthing MJ (1988) History and rationale of oral rehydration and recent developments in formulating an optimal solution. Drugs 36 Suppl 4: 80-90.

1419. Farthing MJ (1989) Disease-related animal models for optimising oral rehydration solution composition. Acta Paediatr Scand Suppl 364: 23-30.

1420. Farthing MJ (2001) Treatment of gastrointestinal viruses. Novartis Found Symp 238: 289-300; discussion 300-285.

1421. Faruque AS, Mahalanabis D, Islam A, Hoque SS, Hasnat A (1993) Common diarrhea pathogens and the risk of dehydration in young children with acute watery diarrhea: a case-control study. Am J Trop Med Hyg 49: 93-100.

1422. Faruque AS, Malek MA, Khan AI, Huq S, Salam MA, et al. (2004) Diarrhoea in elderly people: aetiology, and clinical characteristics. Scand J Infect Dis 36: 204-208.

1423. Fassi-Fehri MM, Johnson DW, Taoudi A, Berrada J (1988) [Epidemiology of diarrhea caused by Escherichia coli and rotavirus in calves and lambs in Morocco]. Ann Rech Vet 19: 59-64.

1424. Fau C, Billaud G, Pinchinat S, Lina B, Kaplon J, et al. (2008) [Epidemiology and burden of rotavirus diarrhea in day care centers in Lyon, France]. Arch Pediatr 15: 1183-1192.

1425. Fauvel M, Spence L, Babiuk LA, Petro R, Bloch S (1978) Hemagglutination and hemagglutination-inhibition studies with a strain of Nebraska calf diarrhea virus (bovine rotavirus). Intervirology 9: 95-105.

1426. Fayram SL, Planta F, Aarnaes SL, Peterson EM, de la Maza LM (1987) Rotavirus gastroenteritis in southern California. Diagn Microbiol Infect Dis 7: 59-62.

1427. Fazli A, Bradley SJ, Kiefel MJ, Jolly C, Holmes IH, et al. (2001) Synthesis and biological evaluation of sialylmimetics as rotavirus inhibitors. J Med Chem 44: 3292-3301.

1428. Fedorova OF, Novikova NA, Epifanova NV, Lukovnikova LB, Kniagina ON, et al. (2005) [Optimization of RT-PCR for the identification of VP4 gene of group A rotaviruses and evaluation of its diagnostic efficiency]. Vopr Virusol 50: 39-41.

1429. Feeney SA, Mitchell SJ, Mitchell F, Wyatt DE, Fairley D, et al. (2006) Association of the G4 rotavirus genotype with gastroenteritis in adults. J Med Virol 78: 1119-1123.

1430. Feklisova LV, Ritova VV, Ratushkina LS (1980) [Diarrhea in children due to a new viral agent]. Sov Med: 50-54.

1431. Feklisova LV, Shebekova VM, Novokshonova VA, Shekoian LA, Blokhina TA (1989) [Severe form of Rotavirus infection during the period of seasonal rise of its incidence]. Pediatriia: 35-40.

1432. Fenaux M, Cuadras MA, Feng N, Jaimes M, Greenberg HB (2006) Extraintestinal spread and replication of a homologous EC rotavirus strain and a heterologous rhesus rotavirus in BALB/c mice. J Virol 80: 5219-5232.

1433. Feng J (1991) [A report of surveillance on acute diarrhoeal diseases in Wuwei Prefecture from 1987 to 1988]. Zhonghua Liu Xing Bing Xue Za Zhi 12: 95-98.

1434. Feng N, Burns JW, Bracy L, Greenberg HB (1994) Comparison of mucosal and systemic humoral immune responses and subsequent protection in mice orally inoculated with a homologous or a heterologous rotavirus. J Virol 68: 7766-7773.

1435. Feng N, Jaimes MC, Lazarus NH, Monak D, Zhang C, et al. (2006) Redundant role of chemokines CCL25/TECK and CCL28/MEC in IgA+ plasmablast recruitment to the intestinal lamina propria after rotavirus infection. J Immunol 176: 5749-5759.

1436. Feng N, Kim B, Fenaux M, Nguyen H, Vo P, et al. (2008) Role of interferon in homologous and heterologous rotavirus infection in the intestines and extraintestinal organs of suckling mice. J Virol 82: 7578-7590.

1437. Feng N, Lawton JA, Gilbert J, Kuklin N, Vo P, et al. (2002) Inhibition of rotavirus replication by a non-neutralizing, rotavirus VP6-specific IgA mAb. J Clin Invest 109: 1203-1213.

1438. Ferguson A, Paul G, Snodgrass DR (1981) Lactose tolerance in lambs with rotavirus diarrhoea. Gut 22: 114-119.

1439. Fernandes JV, Fonseca SM, Azevedo JC, Maranhao Hde S, Fonseca MH, et al. (2000) [Rotavirus detection in feces of children with acute diarrhea]. J Pediatr (Rio J) 76: 300-304.

1440. Fernandez D, Valle I, Llamos R, Guerra M, Sorell L, et al. (1994) Rapid detection of rotavirus in faeces using a dipstick system with monoclonal antibodies and colloidal gold as marker. J Virol Methods 48: 315-323.

1441. Fernandez Fernandez MA, Madruga Garrido M, Blanco Martinez B, Rufo Campos M (2008) [Status epilepticus associated with mild rotavirus gastroenteritis]. An Pediatr (Barc) 69: 263-266.

1442. Fernandez FM, Conner ME, Hodgins DC, Parwani AV, Nielsen PR, et al. (1998) Passive immunity to bovine rotavirus in newborn calves fed colostrum supplements from cows immunized with recombinant SA11 rotavirus core-like particle (CLP) or virus-like particle (VLP) vaccines. Vaccine 16: 507-516.

1443. Fernandez J, Sandino A, Yudelevich A, Avendano LF, Venegas A, et al. (1992) Rotavirus detection by dot blot hybridization assay using a non-radioactive synthetic oligodeoxynucleotide probe. Epidemiol Infect 108: 175-184.

1444. Fernandez J, Sandino AM, Pizarro J, Avendano LF, Pizarro JM, et al. (1991) Characterization of rotavirus electropherotypes excreted by symptomatic and asymptomatic infants. Epidemiol Infect 106: 189-198.

1445. Fernandez-Fernandez MA, Madruga-Garrido M, Blanco-Martinez B, Mateos-Checa R, Rufo-Campos M (2008) [Epileptic status associated with mild gastroenteritis caused by rotavirus]. Rev Neurol 47: 278.

1446. Fernbach SK, Lloyd-Still JD (1984) The radiographic findings in severe rotavirus-induced colitis. J Can Assoc Radiol 35: 192-194.

1447. Ferner WT, Miskuff RL, Yolken RH, Vonderfecht SL (1987) Comparison of methods for detection of serum antibody to murine rotavirus. J Clin Microbiol 25: 1364-1369.

1448. Ferrante P, Cappellini D, Ferliga A, Ballerini P, Cauda-Pedretti C, et al. (1983) [Rotavirus infections in a neonatal pathology unit]. Boll Ist Sieroter Milan 62: 37-46.

1449. Ferrante P, Fasan M, Barbi M (1980) [Seroepidemiologic survey of the spread of rotavirus infection in a Milan population]. Boll Ist Sieroter Milan 59: 126-132.

1450. Ferrari M, Francavilla M, Milanesi G, Capucci L (1987) A comparison of seven strains of porcine rotavirus as studied by serum neutralization and RNA electrophoresis. Microbiologica 10: 339-344.

1451. Ferrari M, Gualandi GL, Gelmetti D (1986) Isolation of cytophatic strains of rotavirus from pigs. Microbiologica 9: 287-294.

1452. Ferreira MS, Xavier MP, Fumian TM, Victoria M, Oliveira SA, et al. (2008) Acute gastroenteritis cases associated with noroviruses infection in the state of Rio de Janeiro. J Med Virol 80: 338-344.

1453. Ferreira MS, Xavier MP, Fumian TM, Victoria M, Oliveira SA, et al. (2008) Acute gastroenteritis cases associated with noroviruses infection in the state of Rio de Janeiro. J Med Virol 80: 338-344.

1454. Ferson MJ (1996) Hospitalisations for rotavirus gastroenteritis among children under five years of age in New South Wales. Med J Aust 164: 273-276.

1455. Ferson MJ, Henry R (1998) Paediatric rotavirus gastroenteritis: where to now in prevention and treatment? Med J Aust 169: 241-242.

1456. Ferson MJ, Stringfellow S, McPhie K, McIver CJ, Simos A (1997) Longitudinal study of rotavirus infection in child-care centres. J Paediatr Child Health 33: 157-160.

1457. Fiehring C (1987) [Gastrointestinal microecology from the viewpoint of the pediatrician]. Nahrung 31: 371-375.

1458. Fiehring C, Korting HJ, Jung G (1984) [Detection of rotavirus in patients with malabsorption]. Nahrung 28: 679-681.

1459. Fiehring C, Korting HJ, Jung G (1984) [Rotavirus and malabsorption. Immunofluorescence microscopy studies of small intestine specimens]. Dtsch Z Verdau Stoffwechselkr 44: 1-5.

1460. Figueroa G, Araya M, Ibanez S, Clerc N, Brunser O (1986) Enteropathogens associated with acute diarrhea in hospitalized infants. J Pediatr Gastroenterol Nutr 5: 226-231.

1461. Figura N, Rossolini A (1985) A prospective etiological and clinical study on gastroenteritis in Italian children. Boll Ist Sieroter Milan 64: 302-310.

1462. Fijan S, Poljsak-Prijatelj M, Steyer A, Koren S, Cencic A, et al. (2006) Rotaviral RNA found in wastewaters from hospital laundry. Int J Hyg Environ Health 209: 97-102.

1463. Fijtman NL, Barrandeguy ME, Cornaglia EM, Schudel AA (1987) Variations and persistency of electropherotypes of bovine rotavirus field isolates. Brief report. Arch Virol 96: 275-281.

1464. Filho EP, da Costa Faria NR, Fialho AM, de Assis RS, Almeida MM, et al. (2007) Adenoviruses associated with acute gastroenteritis in hospitalized and community children up to 5 years old in Rio de Janeiro and Salvador, Brazil. J Med Microbiol 56: 313-319.

1465. Finlaison DS (1995) Faecal viruses of dogs--an electron microscope study. Vet Microbiol 46: 295-305.

1466. Fiore ES, Barini AM, de Mendonca ER, de Lima QL, Molica T, et al. (1986) [Incidence of rotavirus in the Menino Jesus Pediatric Hospital (SP): ELISA--a practical and efficient method for its detection]. Arq Gastroenterol 23: 242-245.

1467. Firer MA, Hosking CS, Hill DJ (1988) Possible role for rotavirus in the development of cows' milk enteropathy in infants. Clin Allergy 18: 53-61.

1468. Fischer TK (2001) Incidence of hospitalizations due to rotavirus gastroenteritis in Denmark. Acta Paediatr 90: 1073-1075.

1469. Fischer TK (2003) Epidemiological studies of rotavirus infection in Guinea-Bissau, West Africa. Dan Med Bull 50: 103-117.

1470. Fischer TK, Anh DD, Antil L, Cat ND, Kilgore PE, et al. (2005) Health care costs of diarrheal disease and estimates of the cost-effectiveness of rotavirus vaccination in Vietnam. J Infect Dis 192: 1720-1726.

1471. Fischer TK, Ashley D, Kerin T, Reynolds-Hedmann E, Gentsch J, et al. (2005) Rotavirus antigenemia in patients with acute gastroenteritis. J Infect Dis 192: 913-919.

1472. Fischer TK, Bresee JS, Glass RI (2004) Rotavirus vaccines and the prevention of hospital-acquired diarrhea in children. Vaccine 22 Suppl 1: S49-54.

1473. Fischer TK, Eugen-Olsen J, Pedersen AG, Molbak K, Bottiger B, et al. (2005) Characterization of rotavirus strains in a Danish population: high frequency of mixed infections and diversity within the VP4 gene of P[8] strains. J Clin Microbiol 43: 1099-1104.

1474. Fischer TK, Gentsch JR (2004) Rotavirus typing methods and algorithms. Rev Med Virol 14: 71-82.

1475. Fischer TK, Molbak K (2001) The costs of an outbreak--an example from a Danish day care setting. Vaccine 20: 637-638.

1476. Fischer TK, Nielsen NM, Wohlfahrt J, Paerregaard A (2007) Incidence and cost of rotavirus hospitalizations in Denmark. Emerg Infect Dis 13: 855-859.

1477. Fischer TK, Viboud C, Parashar U, Malek M, Steiner C, et al. (2007) Hospitalizations and deaths from diarrhea and rotavirus among children <5 years of age in the United States, 1993-2003. J Infect Dis 195: 1117-1125.

1478. Fitts SW, Green M, Reyes J, Nour B, Tzakis AG, et al. (1995) Clinical features of nosocomial rotavirus infection in pediatric liver transplant recipients. Clin Transplant 9: 201-204.

1479. Flahault A, Blanchon T, Dorleans Y, Toubiana L, Vibert JF, et al. (2006) Virtual surveillance of communicable diseases: a 20-year experience in France. Stat Methods Med Res 15: 413-421.

1480. Fleenor JT, Hoffman TM, Bush DM, Paridon SM, Clark BJ, 3rd, et al. (2002) Pneumatosis intestinalis after pediatric thoracic organ transplantation. Pediatrics 109: E78-78.

1481. Fleet GH, Heiskanen P, Reid I, Buckle KA (2000) Foodborne viral illness--status in Australia. Int J Food Microbiol 59: 127-136.

1482. Fleming FE, Graham KL, Taniguchi K, Takada Y, Coulson BS (2007) Rotavirus-neutralizing antibodies inhibit virus binding to integrins alpha 2 beta 1 and alpha 4 beta 1. Arch Virol 152: 1087-1101.

1483. Flewett TH (1976) Implications of recent virological researches. Ciba Found Symp: 237-250.

1484. Flewett TH (1978) Electron microscopy in the diagnosis of infectious diarrhea. J Am Vet Med Assoc 173: 538-543.

1485. Flewett TH (1982) New prospects for control of virus diarrhoea in children. J R Soc Med 75: 493-494.

1486. Flewett TH (1984) Rotaviruses: expectations for a vaccine. Diarrhoea Dialogue: 3.

1487. Flewett TH, Arias CF, Avendano LF, Ghafoor A, Mathan MM, et al. (1989) Comparative evaluation of the WHO and DAKOPATTS enzyme-linked immunoassay kits for rotavirus detection. Bull World Health Organ 67: 369-374.

1488. Flewett TH, Beards GM, Brown DW, Sanders RC (1987) The diagnostic gap in diarrhoeal aetiology. Ciba Found Symp 128: 238-249.

1489. Flewett TH, Woode GN (1978) The rotaviruses. Arch Virol 57: 1-23.

1490. Flores J, Boeggeman E, Purcell RH, Sereno M, Perez I, et al. (1983) A dot hybridisation assay for detection of rotavirus. Lancet 1: 555-558.

1491. Flores J, Kapikian AZ (1990) Vaccines against viral diarrhoea. Baillieres Clin Gastroenterol 4: 675-693.

1492. Flores J, Midthun K, Hoshino Y, Green K, Gorziglia M, et al. (1986) Conservation of the fourth gene among rotaviruses recovered from asymptomatic newborn infants and its possible role in attenuation. J Virol 60: 972-979.

1493. Flores J, Nakagomi O, Nakagomi T, Glass R, Gorziglia M, et al. (1986) The role of rotaviruses in pediatric diarrhea. Pediatr Infect Dis 5: S53-62.

1494. Flores J, Perez I, White L, Perez M, Kalica AR, et al. (1982) Genetic relatedness among human rotaviruses as determined by RNA hybridization. Infect Immun 37: 648-655.

1495. Flores J, Perez-Schael I, Blanco M, Rojas AM, Alfonzo E, et al. (1993) Reactogenicity and immunogenicity of a high-titer rhesus rotavirus-based quadrivalent rotavirus vaccine. J Clin Microbiol 31: 2439-2445.

1496. Flores J, Perez-Schael I, Gonzalez M, Garcia D, Perez M, et al. (1987) Protection against severe rotavirus diarrhoea by rhesus rotavirus vaccine in Venezuelan infants. Lancet 1: 882-884.

1497. Flores J, Taniguchi K, Green K, Perez-Schael I, Garcia D, et al. (1988) Relative frequencies of rotavirus serotypes 1, 2, 3, and 4 in Venezuelan infants with gastroenteritis. J Clin Microbiol 26: 2092-2095.

1498. Flores J, White L, Blanco M, Perez-Schael I (1994) Serological response to rotavirus infection in newborn infants. J Med Virol 42: 97-102.

1499. Flores-Abuxapqui JJ, Suarez-Hoil GJ, Puc-Franco MA, Heredia-Navarrete MR, Franco-Monsreal J (1993) [Prevalence of enteropathogens in children with liquid diarrhea]. Rev Latinoam Microbiol 35: 351-356.

1500. Floret D, Lina B, Pinchinat S, Billaud G, Ait-Belghiti F, et al. (2006) Epidemiology and burden of rotavirus diarrhea in day care centers in Lyon, France. Eur J Pediatr 165: 905-906.

1501. Fodha I, Chouikha A, Dewar J, Trabelsi A, Boujaafar N, et al. (2007) [Prevalence of adenovirus antigens in children presenting with acute diarrhoea]. Med Trop (Mars) 67: 256-258.

1502. Fodha I, Chouikha A, Peenze I, De Beer M, Dewar J, et al. (2006) Identification of viral agents causing diarrhea among children in the Eastern Center of Tunisia. J Med Virol 78: 1198-1203.

1503. Foldenauer A, Vossbeck S, Pohlandt F (1998) Neonatal hypocalcaemia associated with rotavirus diarrhoea. Eur J Pediatr 157: 838-842.

1504. Follett EA, Desselberger U (1983) Cocirculation of different rotavirus strains in a local outbreak of infantile gastroenteritis: monitoring by rapid and sensitive nucleic acid analysis. J Med Virol 11: 39-52.

1505. Follett EA, Sanders RC, Beards GM, Hundley F, Desselberger U (1984) Molecular epidemiology of human rotaviruses. Analysis of outbreaks of acute gastroenteritis in Glasgow and the west of Scotland 1981/82 and 1982/83. J Hyg (Lond) 92: 209-222.

1506. Fontana M, Zuin G, Galli L, Paccagnini S, Villa M, et al. (1988) Fecal alpha-1-antitrypsin excretion in acute diarrhea: relationship with causative pathogens. Helv Paediatr Acta 43: 211-218.

1507. Fontana M, Zuin G, Mammino A, Tocalli L, Marchisio P, et al. (1996) Rotavirus infection and diarrhea in healthy and HIV-infected children: a cohort study. J Pediatr Gastroenterol Nutr 23: 492-496.

1508. Fonteyne J, Zissis G, Lambert JP (1978) Recurrent rotavirus gastroenteritis. Lancet 1: 983.

1509. Fonteyne JL, Zissis G, Butzler JP, Lambert JP, De Kegel D, et al. (1977) Diarrhea with rotaviruses in a pediatric surgery in Brussels. Acta Clin Belg 32: 280-281.

1510. Foppa IM, Karmaus W, Ehlken B, Fruhwirth M, Heininger U, et al. (2006) Health care-associated rotavirus illness in pediatric inpatients in Germany, Austria, and Switzerland. Infect Control Hosp Epidemiol 27: 633-635.

1511. Forbes C, Hawkes M, Nesbitt S (2004) Stool viruses among paediatric patients from a Nairobi clinic, Kenya. East Afr Med J 81: 562-567.

1512. Ford T (2006) Emerging issues in water and health research. J Water Health 4 Suppl 1: 59-65.

1513. Ford-Jones EL, Mindorff CM, Gold R, Petric M (1990) The incidence of viral-associated diarrhea after admission to a pediatric hospital. Am J Epidemiol 131: 711-718.

1514. Ford-Jones EL, Wang E, Petric M, Corey P, Moineddin R, et al. (2000) Hospitalization for community-acquired, rotavirus-associated diarrhea: a prospective, longitudinal, population-based study during the seasonal outbreak. The Greater Toronto Area/Peel Region PRESI Study Group. Pediatric Rotavirus Epidemiology Study for Immunization. Arch Pediatr Adolesc Med 154: 578-585.

1515. Ford-Jones EL, Wang E, Petric M, Corey P, Moineddin R, et al. (2000) Rotavirus-associated diarrhea in outpatient settings and child care centers. The Greater Toronto Area/Peel Region PRESI Study Group. Pediatric Rotavirus Epidemiology Study for Immunization. Arch Pediatr Adolesc Med 154: 586-593.

1516. Forrer CB, Rodden JM, Clark HF, Friedman HM (1989) Discrepant rotavirus results in two laboratories using the same enzyme immunoassay. Am J Clin Pathol 91: 85-87.

1517. Forster J, Hammerschmidt T (2007) [Burden of acute rotavirus gastroenteritis (RV-AGE) in Germany: a comparison of federal statistics and epidemiological data]. Gesundheitswesen 69: 227-232.

1518. Forster J, Knoop U (1983) [Nosocomial dyspepsia in newborn and young infants. A 15-month prospective study with continuous Rotavirus surveillance]. Monatsschr Kinderheilkd 131: 441-447.

1519. Forster J, Luthardt T (1979) [Rota virus infection in the newborn and infant -- epidemiological aspects (author's transl)]. Klin Padiatr 191: 472-476.

1520. Forster J, Pastor S (1983) Epidemiology of human rotaviruses as determined by electrophoresis of genome RNA. Eur J Clin Microbiol 2: 141-147.

1521. Foster RH, Wagstaff AJ (1998) Tetravalent Human-Rhesus Reassortant Rotavirus Vaccine: A Review of its Immunogenicity, Tolerability and Protective Efficacy against Paediatric Rotavirus Gastroenteritis. BioDrugs 9: 155-178.

1522. Foster SO, Palmer EL, Gary GW, Jr., Martin ML, Herrmann KL, et al. (1980) Gastroenteritis due to rotavirus in an isolated Pacific island group: an epidemic of 3,439 cases. J Infect Dis 141: 32-39.

1523. Fourquet F, Desenclos JC, Maurage C, Baron S (2003) [Acute gastro-enteritis in children in France: estimates of disease burden through national hospital discharge data]. Arch Pediatr 10: 861-868.

1524. Fox CH, Dang G (2004) Probiotics in the prevention and treatment of diarrhea. J Altern Complement Med 10: 601-603.

1525. Fragoso M, Kumar A, Murray DL (1986) Rotavirus in nasopharyngeal secretions of children with upper respiratory tract infections. Diagn Microbiol Infect Dis 4: 87-88.

1526. Franco MA, Angel J, Greenberg HB (2006) Immunity and correlates of protection for rotavirus vaccines. Vaccine 24: 2718-2731.

1527. Franco MA, Feng N, Greenberg HB (1996) Molecular determinants of immunity and pathogenicity of rotavirus infection in the mouse model. J Infect Dis 174 Suppl 1: S47-50.

1528. Franco MA, Greenberg HB (2000) Immunity to homologous rotavirus infection in adult mice. Trends Microbiol 8: 50-52.

1529. Freedman SB (2007) Acute infectious pediatric gastroenteritis: beyond oral rehydration therapy. Expert Opin Pharmacother 8: 1651-1665.

1530. Freeman MM, Kerin T, Hull J, McCaustland K, Gentsch J (2008) Enhancement of detection and quantification of rotavirus in stool using a modified real-time RT-PCR assay. J Med Virol 80: 1489-1496.

1531. Freestone DS (1985) The need for new antiviral agents. Antiviral Res 5: 307-324.

1532. Freitas ER, Soares CM, Fiaccadori FS, Souza M, Parente JA, et al. (2008) Occurrence of group A rotavirus mixed P genotypes infections in children living in Goiania-Goias, Brazil. Eur J Clin Microbiol Infect Dis 27: 1065-1069.

1533. Freitas RB, Gabbay YB, Pereira JD, Linhares AC, Lins ZC, et al. (1987) [Familial outbreaks of acute gastroenteritis associated with rotavirus in Belem, Para]. Rev Latinoam Microbiol 29: 226-229.

1534. Fric P (2002) [Probiotics in gastroenterology]. Z Gastroenterol 40: 197-201.

1535. Friedman MG, Galil A, Sarov B, Margalith M, Katzir G, et al. (1988) Two sequential outbreaks of rotavirus gastroenteritis: evidence for symptomatic and asymptomatic reinfections. J Infect Dis 158: 814-822.

1536. Friedman MG, Segal B, Zedaka R, Sarov B, Margalith M, et al. (1993) Serum and salivary responses to oral tetravalent reassortant rotavirus vaccine in newborns. Clin Exp Immunol 92: 194-199.

1537. Froggatt PC, Barry Vipond I, Ashley CR, Lambden PR, Clarke IN, et al. (2004) Surveillance of norovirus infection in a study of sporadic childhood gastroenteritis in South West England and South Wales, during one winter season (1999-2000). J Med Virol 72: 307-311.

1538. Fromantin C, Piroth L, Petitpas I, Pothier P, Kohli E (1998) Oral delivery of homologous and heterologous strains of rotavirus to BALB/c mice induces the same profile of cytokine production by spleen cells. Virology 244: 252-260.

1539. Fruhwirth M, Berger K, Ehlken B, Moll-Schuler I, Brosl S, et al. (2001) Economic impact of community- and nosocomially acquired rotavirus gastroenteritis in Austria. Pediatr Infect Dis J 20: 184-188.

1540. Fruhwirth M, Brosl S, Ellemunter H, Moll-Schuler I, Rohwedder A, et al. (2000) Distribution of rotavirus VP4 genotypes and VP7 serotypes among nonhospitalized and hospitalized patients with gastroenteritis and patients with nosocomially acquired gastroenteritis in Austria. J Clin Microbiol 38: 1804-1806.

1541. Fruhwirth M, Fischer H, Simma B, Hochleitner B, Konigsrainer A, et al. (2001) Rotavirus infection as cause of tacrolimus elevation in solid-organ-transplanted children. Pediatr Transplant 5: 88-92.

1542. Fruhwirth M, Heininger U, Ehlken B, Petersen G, Laubereau B, et al. (2001) International variation in disease burden of rotavirus gastroenteritis in children with community- and nosocomially acquired infection. Pediatr Infect Dis J 20: 784-791.

1543. Fruhwirth M, Karmaus W, Moll-Schuler I, Brosl S, Mutz I (2001) A prospective evaluation of community acquired gastroenteritis in paediatric practices: impact and disease burden of rotavirus infection. Arch Dis Child 84: 393-397.

1544. Fu C, Wang M, Liang J, He T, Wang D, et al. (2007) Effectiveness of Lanzhou lamb rotavirus vaccine against rotavirus gastroenteritis requiring hospitalization: A matched case-control study. Vaccine 25: 8756-8761.

1545. Fu W, Hao W, Peng Y (1998) [Detection of rotavirus RNA using DIG labelled probe prepared by polymerase chain reaction]. Zhonghua Shi Yan He Lin Chuang Bing Du Xue Za Zhi 12: 77-79.

1546. Fu ZF, Hampson DJ (1987) Group A rotavirus excretion patterns in naturally infected pigs. Res Vet Sci 43: 297-300.

1547. Fujii R, Kuzuya M, Hamano M, Ogura H, Yamada M, et al. (2000) Neutralization assay for human group C rotaviruses using a reverse passive hemagglutination test for endpoint determination. J Clin Microbiol 38: 50-54.

1548. Fujinaga S, Kaneko K, Ohtomo Y, Takada M, Kobayashi K, et al. (2005) Acute renal failure due to obstructive uric acid stones associated with rotavirus gastroenteritis. Pediatr Nephrol 20: 239-240.

1549. Fujita K, Kaku M, Yanagase Y, Ezaki T, Furuse K, et al. (1990) Physicochemical characteristics and flora of diarrhoeal and recovery faeces in children with acute gastro-enteritis in Kenya. Ann Trop Paediatr 10: 339-345.

1550. Fujita Y (1990) [Comparison of clinical features of rotavirus infection with rotavirus antigen titers in feces obtained during the acute phase]. Kansenshogaku Zasshi 64: 1168-1171.

1551. Fujita Y (1990) [Fecal IgA antibody against rotavirus and clinical manifestations of acute viral gastroenteritis in children]. Kansenshogaku Zasshi 64: 980-985.

1552. Fujita Y (1990) [Rotavirus infection--clinical symptoms and influence of climate]. Kansenshogaku Zasshi 64: 1255-1263.

1553. Fujita Y, Hiyoshi K, Wakasugi N, Sakuta R, Yanagida Y, et al. (1988) [Transient improvement of the West syndrome in two cases following rotavirus colitis]. No To Hattatsu 20: 59-63.

1554. Fujita Y, Yamada H, Araki K, Kobayashi M, Tajima T, et al. (1994) [Detection of group C rotavirus in the day care center]. Kansenshogaku Zasshi 68: 723-727.

1555. Fujita Y, Yamada H, Araki K, Tajima T, Abe T, et al. (1992) [Serotypes and electropherotypes of group A human rotaviruses in patients with acute gastroenteritis in Saitama area, 1988-1991]. Kansenshogaku Zasshi 66: 721-728.

1556. Fukai K, Maeda Y, Fujimoto K, Itou T, Sakai T (2002) Changes in the prevalence of rotavirus G and P types in diarrheic calves from the Kagoshima prefecture in Japan. Vet Microbiol 86: 343-349.

1557. Fukai K, Onoda H, Itou T, Sato M, Miura Y, et al. (2004) Genetic and serological characterization of novel serotype G8 bovine group A rotavirus strains isolated in Japan. J Vet Med Sci 66: 1413-1416.

1558. Fukai K, Saito T, Fukuda O, Hagiwara A, Inoue K, et al. (2006) Molecular characterisation of equine group A rotavirus, Nasuno, isolated in Tochigi Prefecture, Japan. Vet J 172: 369-373.

1559. Fukai K, Saito T, Inoue K, Sato M (2004) Molecular characterization of novel P[14],G8 bovine group A rotavirus, Sun9, isolated in Japan. Virus Res 105: 101-106.

1560. Fukai K, Sakai T, Hirose M, Itou T (1999) Prevalence of calf diarrhea caused by bovine group A rotavirus carrying G serotype 8 specificity. Vet Microbiol 66: 301-311.

1561. Fukai K, Sakai T, Kamata H (1998) Distribution of G serotypes and P genotypes of bovine group A rotavirus isolated in Japan. Aust Vet J 76: 418-422.

1562. Fukai K, Takahashi T, Tajima K, Koike S, Iwane K, et al. (2007) Molecular characterization of a novel bovine group A rotavirus. Vet Microbiol 123: 217-224.

1563. Fukai K, Yamada K, Inoue K (2005) Serological characterization of novel P11[14],G8 bovine group A rotavirus, Sun9, isolated in Japan. Virus Res 114: 167-171.

1564. Fukuda S, Takao S, Kuwayama M, Shimazu Y, Miyazaki K (2006) Rapid detection of norovirus from fecal specimens by real-time reverse transcription-loop-mediated isothermal amplification assay. J Clin Microbiol 44: 1376-1381.

1565. Fukui Y, Suzuki M, Yanai Y, Eda J, Terasawa N, et al. (1989) [Rotavirus infections in traveler's diarrhea]. Kansenshogaku Zasshi 63: 1296-1300.

1566. Fukushima A, Yoo YC, Yoshimatsu K, Matsuzawa K, Tamura M, et al. (1996) Effect of MDP-Lys(L18) as a mucosal immunoadjuvant on protection of mucosal infections by Sendai virus and rotavirus. Vaccine 14: 485-491.

1567. Fukusho A, Shimizu Y, Ito Y (1981) Isolation of cytopathic porcine rotavirus in cell roller culture in the presence of trypsin. Arch Virol 69: 49-60.

1568. Fukutomi T, Fujiwara M, Sanekata T, Akashi H (1995) Isolation of a serotype G6P11 bovine rotavirus showing two-way cross-neutralization with the serotype G10P11 virus. J Vet Med Sci 57: 739-741.

1569. Fulton RW, Johnson CA, Pearson NJ, Woode GN (1981) Isolation of a rotavirus from a newborn dog with diarrhea. Am J Vet Res 42: 841-843.

1570. Fun BN, Unicomb L, Rahim Z, Banu NN, Podder G, et al. (1991) Rotavirus-associated diarrhea in rural Bangladesh: two-year study of incidence and serotype distribution. J Clin Microbiol 29: 1359-1363.

1571. Furuya Y, Katayama T, Miyahara K, Kobayashi A, Funabiki T (2007) Detection of the rotavirus a genome from the cerebrospinal fluid of a gastroenteritis patient: a case report. Jpn J Infect Dis 60: 148-149.

1572. Fyderek K, Pituch-Noworolska A, Stopyrowa J, Malachowski J (1987) [Rotavirus as a cause of acute diarrhea in infants]. Pediatr Pol 62: 241-244.

1573. Gabbay YB, Jiang B, Oliveira CS, Mascarenhas JD, Leite JP, et al. (1999) An outbreak of group C rotavirus gastroenteritis among children attending a day-care centre in Belem, Brazil. J Diarrhoeal Dis Res 17: 69-74.

1574. Gabbay YB, Linhares AC, Cavalcante-Pepino EL, Nakamura LS, Oliveira DS, et al. (2007) Prevalence of human astrovirus genotypes associated with acute gastroenteritis among children in Belem, Brazil. J Med Virol 79: 530-538.

1575. Gabbay YB, Mascarenhas JD, Linhares AC, Freitas RB (1989) Atypical rotavirus among diarrhoeic children living in Belem, Brazil. Mem Inst Oswaldo Cruz 84: 5-8.

1576. Gabutti G, Marsella M, Lazzara C, Fiumana E, Cavallaro A, et al. (2007) Epidemiology and burden of rotavirus-associated hospitalizations in Ferrara, Italy. J Prev Med Hyg 48: 5-9.

1577. Gaggero A, Avendano LF, Fernandez J, Spencer E (1992) Nosocomial transmission of rotavirus from patients admitted with diarrhea. J Clin Microbiol 30: 3294-3297.

1578. Gaggero A, O'Ryan M, Noel JS, Glass RI, Monroe SS, et al. (1998) Prevalence of astrovirus infection among Chilean children with acute gastroenteritis. J Clin Microbiol 36: 3691-3693.

1579. Galati JC, Harsley S, Richmond P, Carlin JB (2006) The burden of rotavirus-related illness among young children on the Australian health care system. Aust N Z J Public Health 30: 416-421.

1580. Galdiero E, Marinelli A, Pisciotta MG, Pagliara I, Di Monteforte ES, et al. (2005) Reverse transcriptase-PCR for the detection of Astrovirus in children with nosocomial acute diarrhoea in Naples, Italy. Med Mal Infect 35: 213-217.

1581. Galil A, Antverg R, Katzir G, Zentner B, Margalith M, et al. (1986) Involvement of infants, children, and adults in a rotavirus gastroenteritis outbreak in a kibbutz in southern Israel. J Med Virol 18: 317-326.

1582. Galko NV, Makarova NG, Vashukova SS, Safonova NV, Sukhinin VP (1984) [Detection of rotavirus in the feces of children with diarrhea]. Vopr Virusol 29: 596-599.

1583. Gallay A, De Valk H, Cournot M, Ladeuil B, Hemery C, et al. (2006) A large multi-pathogen waterborne community outbreak linked to faecal contamination of a groundwater system, France, 2000. Clin Microbiol Infect 12: 561-570.

1584. Gallimore CI, Cheesbrough JS, Lamden K, Bingham C, Gray JJ (2005) Multiple norovirus genotypes characterised from an oyster-associated outbreak of gastroenteritis. Int J Food Microbiol 103: 323-330.

1585. Gallimore CI, Pipkin C, Shrimpton H, Green AD, Pickford Y, et al. (2005) Detection of multiple enteric virus strains within a foodborne outbreak of gastroenteritis: an indication of the source of contamination. Epidemiol Infect 133: 41-47.

1586. Gallimore CI, Taylor C, Gennery AR, Cant AJ, Galloway A, et al. (2006) Environmental monitoring for gastroenteric viruses in a pediatric primary immunodeficiency unit. J Clin Microbiol 44: 395-399.

1587. Ganaba R, Belanger D, Dea S, Bigras-Poulin M (1995) A seroepidemiological study of the importance in cow-calf pairs of respiratory and enteric viruses in beef operations from northwestern Quebec. Can J Vet Res 59: 26-33.

1588. Gao JM, Wang T, Chen JL, Chen JY, Lin Y, et al. (2004) [Three kinds of viruses isolated from diarrhea patients of infants and preschool children in Fuzhou city]. Zhonghua Liu Xing Bing Xue Za Zhi 25: 456-457.

1589. Gao YG, Jin Y, Liu YL, Ye XH (2006) [Variation and significance of serum and stool IL-18 and IFN-gamma levels in children with rotavirus enteritis]. Zhongguo Dang Dai Er Ke Za Zhi 8: 304-306.

1590. Gaon D, Garcia H, Winter L, Rodriguez N, Quintas R, et al. (2003) Effect of Lactobacillus strains and Saccharomyces boulardii on persistent diarrhea in children. Medicina (B Aires) 63: 293-298.

1591. Garaicoechea L, Bok K, Jones LR, Combessies G, Odeon A, et al. (2006) Molecular characterization of bovine rotavirus circulating in beef and dairy herds in Argentina during a 10-year period (1994-2003). Vet Microbiol 118: 1-11.

1592. Garaicoechea L, Olichon A, Marcoppido G, Wigdorovitz A, Mozgovoj M, et al. (2008) Llama-derived single-chain antibody fragments directed to rotavirus VP6 protein possess broad neutralizing activity in vitro and confer protection against diarrhea in mice. J Virol 82: 9753-9764.

1593. Garbag-Chenon A, Fontaine JL, Lasfargues G, Clark HF, Guyot J, et al. (1989) Reactogenicity and immunogenicity of rotavirus WC3 vaccine in 5-12 month old infants. Res Virol 140: 207-217.

1594. Garbarg-Chenon A (1987) [Viruses of pediatric gastroenteritis]. Ann Pediatr (Paris) 34: 503-507.

1595. Garcia A, Ruiz-Santa-Quiteria JA, Orden JA, Cid D, Sanz R, et al. (2000) Rotavirus and concurrent infections with other enteropathogens in neonatal diarrheic dairy calves in Spain. Comp Immunol Microbiol Infect Dis 23: 175-183.

1596. Garcia JL, Marquez S, Alvarez-Dardet C, Perea EJ (1989) [Healthy carriers of enteropathogenic micro-organisms among the child population of Seville]. Enferm Infecc Microbiol Clin 7: 478-481.

1597. Garcia-Garcia ML, Calvo C, Casas I, Bracamonte T, Rellan A, et al. (2007) Human metapneumovirus bronchiolitis in infancy is an important risk factor for asthma at age 5. Pediatr Pulmonol 42: 458-464.

1598. Garcia-Sanchez J, Corral C, Halaihel NG, Simon MC, Alonso JL, et al. (1993) Survey of rotavirus infection in a dairy herd: comparison between polyacrylamide gel electrophoresis and two commercial tests. Vet Microbiol 34: 321-332.

1599. Gascon J (2006) Epidemiology, etiology and pathophysiology of traveler's diarrhea. Digestion 73 Suppl 1: 102-108.

1600. Gascon J, Vila J, Valls ME, Ruiz L, Vidal J, et al. (1993) Etiology of traveller's diarrhea in Spanish travellers to developing countries. Eur J Epidemiol 9: 217-223.

1601. Gassama A, Sow PS, Fall F, Camara P, Gueye-N'diaye A, et al. (2001) Ordinary and opportunistic enteropathogens associated with diarrhea in Senegalese adults in relation to human immunodeficiency virus serostatus. Int J Infect Dis 5: 192-198.

1602. Gassama A, Thiaw B, Dia NM, Fall F, Camara P, et al. (2001) [Infective etiology of diarrhea in adults with HIV infection in Dakar: a case-control study on 594 patients]. Dakar Med 46: 46-50.

1603. Gatheru Z, Kobayashi N, Adachi N, Chiba S, Muli J, et al. (1993) Characterization of human rotavirus strains causing gastroenteritis in Kenya. Epidemiol Infect 110: 419-423.

1604. Gatti MS, de Castro AF, Ferraz MM, Fialho AM, Pereira HG (1989) Viruses with bisegmented double-stranded RNA in pig faeces. Res Vet Sci 47: 397-398.

1605. Gatti MS, Ferraz MM, Racz ML, de Castro AF (1993) Rotavirus excretion in naturally infected pigs with and without diarrhoea. Vet Microbiol 37: 187-190.

1606. Gatti MS, Hara NH, Ferraz MM, Pestana de Castro AF (1989) Presence of group A and non-A rotaviruses in neonatal piglets in Campinas, SP, Brazil. Med Microbiol Immunol 178: 347-349.

1607. Gatti MS, Ricci LC, Serafim MB, De Castro AF (1989) [The incidence of enterotoxigenic Escherichia coli, rotavirus and Clostridium perfringens from cases of diarrhea in children, in the region of Campinas, SP, Brazil]. Rev Inst Med Trop Sao Paulo 31: 392-398.

1608. Gault E, Chikhi-Brachet R, Delon S, Schnepf N, Albiges L, et al. (1999) Distribution of human rotavirus G types circulating in Paris, France, during the 1997-1998 epidemic: high prevalence of type G4. J Clin Microbiol 37: 2373-2375.

1609. Gbewonyo AJ (1982) Rapid and reliable method for diagnostic electron microscopy of faeces. J Microsc 126: 191-195.

1610. Gebhard RL, Greenberg HB, Singh N, Henry P, Sharp HL, et al. (1982) Acute viral enteritis and exacerbations of inflammatory bowel disease. Gastroenterology 83: 1207-1209.

1611. Geier DA, King PG, Sykes LK, Geier MR (2008) RotaTeq vaccine adverse events and policy considerations. Med Sci Monit 14: PH9-16.

1612. Gelbart B, Hansen-Knarhoi M, Binns P, Krause V (2006) Rotavirus outbreak in a remote Aboriginal community: the burden of disease. J Paediatr Child Health 42: 775-780.

1613. Gelberg HB, Patterson JS, Woode GN (1991) A longitudinal study of rotavirus antibody titers in swine in a closed specific pathogen-free herd. Vet Microbiol 28: 231-242.

1614. Gelberg HB, Woode GN, Kniffen TS, Hardy M, Hall WF (1991) The shedding of group A rotavirus antigen in a newly established closed specific pathogen-free swine herd. Vet Microbiol 28: 213-229.

1615. Gellert GA, Waterman SH, Ewert D, Oshiro L, Giles MP, et al. (1990) An outbreak of acute gastroenteritis caused by a small round structured virus in a geriatric convalescent facility. Infect Control Hosp Epidemiol 11: 459-464.

1616. Gendrel D, Akaga R, Ivanoff B, Okouoyo E, Nguemby-Mbina C (1984) [Acute gastroenteritis and breast feeding in Gabon. Preliminary results]. Med Trop (Mars) 44: 323-325.

1617. Gendrel D, Basse N, Palmer P, Marc E, Taty-Taty R, et al. (1999) [Coincidental outbreaks of rotavirus and respiratory syncytial virus in Paris: a survey from 1993 to 1998]. Arch Pediatr 6: 735-739.

1618. Gendrel D, Sitbon M, Richard-Lenoble D, Galliot A, Kombila M, et al. (1985) [Etiology of acute infantile gastroenteritis in Gabon]. Bull Soc Pathol Exot Filiales 78: 290-295.

1619. Genoud J (1975) [Infantile gastroenteritis: a new viral agent, the rotavirus]. Pediatrie 30: 432-434.

1620. Gentsch JR, Das BK, Jiang B, Bhan MK, Glass RI (1993) Similarity of the VP4 protein of human rotavirus strain 116E to that of the bovine B223 strain. Virology 194: 424-430.

1621. Gentsch JR, Glass RI, Woods P, Gouvea V, Gorziglia M, et al. (1992) Identification of group A rotavirus gene 4 types by polymerase chain reaction. J Clin Microbiol 30: 1365-1373.

1622. Gentsch JR, Woods PA, Ramachandran M, Das BK, Leite JP, et al. (1996) Review of G and P typing results from a global collection of rotavirus strains: implications for vaccine development. J Infect Dis 174 Suppl 1: S30-36.

1623. Georges MC, Roure C, Tauxe RV, Meunier DM, Merlin M, et al. (1987) Diarrheal morbidity and mortality in children in the Central African Republic. Am J Trop Med Hyg 36: 598-602.

1624. Georges MC, Wachsmuth IK, Meunier DM, Nebout N, Didier F, et al. (1984) Parasitic, bacterial, and viral enteric pathogens associated with diarrhea in the Central African Republic. J Clin Microbiol 19: 571-575.

1625. Georges-Courbot MC, Baya C, Abdul-Wahid S, Meunier D, Georges AJ (1984) [The role of rotaviruses in the etiology of infantile diarrhea in the Central African Republic]. Bull Soc Pathol Exot Filiales 77: 32-38.

1626. Georges-Courbot MC, Beraud AM, Beards GM, Campbell AD, Gonzalez JP, et al. (1988) Subgroups, serotypes, and electrophoretypes of rotavirus isolated from children in Bangui, Central African Republic. J Clin Microbiol 26: 668-671.

1627. Georges-Courbot MC, Monges J, Siopathis MR, Roungou JB, Gresenguet G, et al. (1991) Evaluation of the efficacy of a low-passage bovine rotavirus (strain WC3) vaccine in children in Central Africa. Res Virol 142: 405-411.

1628. Georgescu L, Turcanu L, Vasilescu E, Dragan M, Plavosin L, et al. (1985) Rotavirus infection in gastroenteritis in children. Morphol Embryol (Bucur) 31: 187-189.

1629. Gerbeaux J, Labrune B, Tournier G (1978) [Pediatrics in 1977]. Rev Prat 28: 1703-1725.

1630. Gerli R, Rossolini A, Braito A (1981) [Personal experience with the application of the electron microscope in research on Rotavirus in the feces of children with acute gastroenteritis]. Ann Sclavo 23: 522-529.

1631. German A, Fattal-German M (1984) [Virologic and immunologic studies on rotavirus excretion in children]. Bull Acad Natl Med 168: 243-248.

1632. German-Fattal M, Bingen E, Lambert-Zechovsky N, German A, Proux MC (1983) [Value of the detection of rotaviruses by an immunoenzymatic technic in the etiologic diagnosis of pediatric gastroenteritis]. Ann Pharm Fr 41: 355-365.

1633. German-Fattal M, Martinez L, German A (1984) [Rotavirus excretion in infants. Virologic and immunologic aspects]. Ann Pharm Fr 42: 227-233.

1634. Germani Y, Morillon M, Begaud E, Dubourdieu H, Costa R, et al. (1994) Two-year study of endemic enteric pathogens associated with acute diarrhea in New Caledonia. J Clin Microbiol 32: 1532-1536.

1635. Gerna G, Battaglia M, Milenesi G, Passarani N, Percivalle E, et al. (1984) Serotyping of cell culture-adapted subgroup 2 human rotavirus strains by neutralization. Infect Immun 43: 722-729.

1636. Gerna G, Cereda PM, Revello MG, Cattaneo E, Battaglia M, et al. (1981) Antigenic and biological relationships between human coronavirus OC43 and neonatal calf diarrhoea coronavirus. J Gen Virol 54: 91-102.

1637. Gerna G, Forster J, Parea M, Sarasini A, Di Matteo A, et al. (1990) Nosocomial outbreak of neonatal gastroenteritis caused by a new serotype 4, subtype 4B human rotavirus. J Med Virol 31: 175-182.

1638. Gerna G, Passarani N, Battaglia M, Rondanelli EG (1985) Human enteric coronaviruses: antigenic relatedness to human coronavirus OC43 and possible etiologic role in viral gastroenteritis. J Infect Dis 151: 796-803.

1639. Gerna G, Passarani N, Cattaneo E, Torsellini M, Percivalle E, et al. (1984) Diagnosis of acute non-bacterial gastroenteritis by rotavirus detection and serology. Microbiologica 7: 29-39.

1640. Gerna G, Sarasini A, Arista S, di Matteo A, Giovannelli L, et al. (1990) Prevalence of human rotavirus serotypes in some European countries 1981-1988. Scand J Infect Dis 22: 5-10.

1641. Gerna G, Sarasini A, di Matteo A, Parea M, Orsolini P, et al. (1988) Identification of two subtypes of serotype 4 human rotavirus by using VP7-specific neutralizing monoclonal antibodies. J Clin Microbiol 26: 1388-1392.

1642. Gerna G, Sarasini A, Di Matteo A, Parea M, Torsellini M, et al. (1989) Rapid detection of human rotavirus strains in stools by single-sandwich enzyme-linked immunosorbent assay systems using monoclonal antibodies. J Virol Methods 24: 43-56.

1643. Gerna G, Sarasini A, Parea M, Arista S, Miranda P, et al. (1992) Isolation and characterization of two distinct human rotavirus strains with G6 specificity. J Clin Microbiol 30: 9-16.

1644. Gerna G, Sarasini A, Passarani N, Torsellini M, Parea M, et al. (1987) Comparative evaluation of a commercial enzyme-linked immunoassay and solid-phase immune electron microscopy for rotavirus detection in stool specimens. J Clin Microbiol 25: 1137-1139.

1645. Gerna G, Sarasini A, Torsellini M, Torre D, Parea M, et al. (1990) Group- and type-specific serologic response in infants and children with primary rotavirus infections and gastroenteritis caused by a strain of known serotype. J Infect Dis 161: 1105-1111.

1646. Gerna G, Sarasini A, Zentilin L, Di Matteo A, Miranda P, et al. (1990) Isolation in Europe of 69 M-like (serotype 8) human rotavirus strains with either subgroup I or II specificity and a long RNA electropherotype. Arch Virol 112: 27-40.

1647. Gerna G, Sears J, Hoshino Y, Steele AD, Nakagomi O, et al. (1994) Identification of a new VP4 serotype of human rotaviruses. Virology 200: 66-71.

1648. Gerna G, Steele AD, Hoshino Y, Sereno M, Garcia D, et al. (1994) A comparison of the VP7 gene sequences of human and bovine rotaviruses. J Gen Virol 75 ( Pt 7): 1781-1784.

1649. Gerna G, Torsellini M, Passarani N, Battaglia M, Percivalle E, et al. (1984) Subgrouping of human rotavirus strains by complement fixation, indirect double-antibody sandwich enzyme-linked immunosorbent assay and solid-phase immune electron microscopy. Arch Virol 81: 193-203.

1650. Geyer A, Crewe-Brown HH, Greeff AS, Fripp PJ, Steele AD, et al. (1993) The microbial aetiology of summer paediatric gastroenteritis at Ga-Rankuwa Hospital in South Africa. East Afr Med J 70: 78-81.

1651. Geyer A, Sebata T, Peenze I, Steele A (1995) A molecular epidemiological study of porcine rotaviruses. J S Afr Vet Assoc 66: 202-205.

1652. Geyer A, Sebata T, Peenze I, Steele AD (1996) Group B and C porcine rotaviruses identified for the first time in South Africa. J S Afr Vet Assoc 67: 115-116.

1653. Geyer A, Steele AD, Peenze I, Lecatsas G (1994) Astrovirus-like particles, adenoviruses and rotaviruses associated with diarrhoea in piglets. J S Afr Vet Assoc 65: 164-166.

1654. Ghazi HO, Khan MA, Telmesani AM, Idress B, Mahomed MF (2005) Rotavirus infection in infants and young children in Makkah, Saudi Arabia. J Pak Med Assoc 55: 231-234.

1655. Ghisolfi J, Thouvenot JP, Olives JP, Brunerie M, Couvras O (1986) [Stool electrolyte concentration in acute infantile diarrhea in France]. Arch Fr Pediatr 43: 317-320.

1656. Ghose LH, Schnagl RD, Holmes IH (1978) Comparison of an enzyme-linked immunosorbent assay for quantitation of rotavirus antibodies with complement fixation in an epidemiological survey. J Clin Microbiol 8: 268-276.

1657. Ghosh AR, Nair GB, Dutta P, Pal SC, Sen D (1991) Acute diarrhoeal diseases in infants aged below six months in hospital in Calcutta, India: an aetiological study. Trans R Soc Trop Med Hyg 85: 796-798.

1658. Ghosh AR, Paul M, Pal SC, Sen D (1990) Etiological agents of diarrhoea. Indian J Public Health 34: 54-61.

1659. Ghosh S, Samajdar S, Sinha M, Kobayashi N, Taniguchi K, et al. (2008) Molecular characterization of rare bovine group A rotavirus G15P[11] and G15P[21] strains from eastern India: identification of simian SA11-like VP6 genes in G15P[21] strains. Virus Genes 37: 241-249.

1660. Ghosh S, Varghese V, Samajdar S, Sinha M, Kobayashi N, et al. (2007) Molecular characterization of bovine group A rotavirus G3P[3] strains. Arch Virol 152: 1935-1940.

1661. Ghosh S, Varghese V, Sinha M, Kobayashi N, Naik TN (2007) Evidence for interstate transmission and increase in prevalence of bovine group B rotavirus strains with a novel VP7 genotype among diarrhoeic calves in Eastern and Northern states of India. Epidemiol Infect 135: 1324-1330.

1662. Ghosh SK, Naik TN (1989) Evidence for a new rotavirus subgroup in India. Epidemiol Infect 102: 523-530.

1663. Giammarioli AM, Mackow ER, Fiore L, Greenberg HB, Ruggeri FM (1996) Production and characterization of murine IgA monoclonal antibodies to the surface antigens of rhesus rotavirus. Virology 225: 97-110.

1664. Gianino P, Mastretta E, Longo P, Laccisaglia A, Sartore M, et al. (2002) Incidence of nosocomial rotavirus infections, symptomatic and asymptomatic, in breast-fed and non-breast-fed infants. J Hosp Infect 50: 13-17.

1665. Giaquinto C, Callegaro S, Andreola B, Bernuzzi M, Cantarutti L, et al. (2008) Prospective study of the burden of acute gastroenteritis and rotavirus gastroenteritis in children less than 5 years of age, in Padova, Italy. Infection 36: 351-357.

1666. Giaquinto C, Van Damme P, Huet F, Gothefors L, Maxwell M, et al. (2007) Clinical consequences of rotavirus acute gastroenteritis in Europe, 2004-2005: the REVEAL study. J Infect Dis 195 Suppl 1: S26-35.

1667. Giaquinto C, Van Damme P, Huet F, Gothefors L, Van der Wielen M (2007) Costs of community-acquired pediatric rotavirus gastroenteritis in 7 European countries: the REVEAL Study. J Infect Dis 195 Suppl 1: S36-S44.

1668. Giaquinto C, Vanin M, Anglani F, Errico G, Ruga E, et al. (1985) A new screening test for rotavirus infection. Infection 13: 260-262.

1669. Gibson JJ, Alexander GR (1985) Correlates of infant death from infectious diarrhea in the southeastern United States. South Med J 78: 26-30.

1670. Gil A, Carrasco P, Jimenez R, San-Martin M, Oyaguez I, et al. (2004) Burden of hospitalizations attributable to rotavirus infection in children in Spain, period 1999-2000. Vaccine 22: 2221-2225.

1671. Gil AI, Lanata CF, Butron B, Gabilondo A, Molina M, et al. (1996) Incidence of Vibrio cholerae O1 diarrhea in children at the onset of cholera epidemic in periurban Lima, Peru. Pediatr Infect Dis J 15: 415-418.

1672. Gil de Miguel A, Carrasco Garrido P, Esteban Hernandez J, San-Martin Rodriguez M, Gonzalez Lopez A (2006) [Burden of hospitalizations attributable to rotavirus infection in children in the Autonomous Region of Madrid, Spain, period 1999-2000]. An Pediatr (Barc) 64: 530-535.

1673. Gil MT, de Souza CO, Asensi M, Buesa J (2000) Homotypic protection against rotavirus-induced diarrhea in infant mice breast-fed by dams immunized with the recombinant VP8* subunit of the VP4 capsid protein. Viral Immunol 13: 187-200.

1674. Gilger MA, Matson DO, Conner ME, Rosenblatt HM, Finegold MJ, et al. (1992) Extraintestinal rotavirus infections in children with immunodeficiency. J Pediatr 120: 912-917.

1675. Gill H, Prasad J (2008) Probiotics, immunomodulation, and health benefits. Adv Exp Med Biol 606: 423-454.

1676. Gill H, Prasad J (2008) Probiotics, immunomodulation, and health benefits. Adv Exp Med Biol 606: 423-454.

1677. Gillespie J, Kalica A, Conner M, Schiff E, Barr M, et al. (1984) The isolation, propagation and characterization of tissue-cultured equine rotaviruses. Vet Microbiol 9: 1-14.

1678. Gilligan PH (1986) Diarrheal disease in the hospitalized patient. Infect Control 7: 607-609.

1679. Gimenez Sanchez F, Delgado Rubio A, Martinon Torres F, Asensi Botet F, Miranda Valdivieso M, et al. (2008) [Family impact of rotavirus gastroenteritis in children under two years.]. An Pediatr (Barc) 69: 515-520.

1680. Gimenez Sanchez F, Martinon Torres F, Bernaola Iturbe E, Baca Cots M, de Juan Martin F, et al. (2006) [The role of the rotavirus vaccine in childhood vaccination schedules]. An Pediatr (Barc) 64: 573-577.

1681. Ginevskaia V, Shekoian LA, Khaustov VI, Galbadrakh D, Ivanova OE (1988) [Detection of the rotavirus antigen in clinical material and environmental objects using variants of immunoenzyme analysis]. Vopr Virusol 33: 493-497.

1682. Ginevskaya VA, Amitina NN, Eremeeva TP, Shirman GA, Priimagi LS, et al. (1994) Electropherotypes and serotypes of human rotavirus in Estonia in 1989-1992. Arch Virol 137: 199-207.

1683. Ginevskaya VA, Eremeeva TP, Zangaladze ED, Shirman GA, Kazantseva VA, et al. (1991) Analysis of rotaviral gastroenteritis in Tbilisi. Acta Virol 35: 232-237.

1684. Ginn DI, Ward RL, Hamparian VV, Hughes JH (1992) Inhibition of rotavirus in vitro transcription by optimal concentrations of monoclonal antibodies specific for rotavirus VP6. J Gen Virol 73 ( Pt 11): 3017-3022.

1685. Gionchetti P, Rizzello F, Venturi A, Campieri M (2000) Probiotics in infective diarrhoea and inflammatory bowel diseases. J Gastroenterol Hepatol 15: 489-493.

1686. Giordano MO, Basnec SN, Nates SV, Bennun F, Depetris AR (1991) Rapid techniques for diagnostic and epidemiological studies of rotavirus infection. J Virol Methods 35: 59-63.

1687. Giordano MO, Ferreyra LJ, Isa MB, Martinez LC, Yudowsky SI, et al. (2001) The epidemiology of acute viral gastroenteritis in hospitalized children in Cordoba City, Argentina: an insight of disease burden. Rev Inst Med Trop Sao Paulo 43: 193-197.

1688. Giordano MO, Martinez LC, Depetris AR, Medeot SI, Nates SV (1997) Rapid vertical agarose: silver stain detection of rotavirus. Viral Immunol 10: 59-64.

1689. Giordano MO, Martinez LC, Ferreyra LJ, Isa MB, Paez Rearte M, et al. (2005) Discrepancies in viral gastroenteritis diagnosis: an unusual dual reovirus-adenovirus infection case. J Clin Virol 32: 71-72.

1690. Giordano MO, Martinez LC, Isa MB, Ferreyra LJ, Canna F, et al. (2002) Twenty year study of the occurrence of reovirus infection in hospitalized children with acute gastroenteritis in Argentina. Pediatr Infect Dis J 21: 880-882.

1691. Giordano MO, Martinez LC, Rinaldi D, Guinard S, Naretto E, et al. (1998) Detection of picobirnavirus in HIV-infected patients with diarrhea in Argentina. J Acquir Immune Defic Syndr Hum Retrovirol 18: 380-383.

1692. Giordano MO, Masachessi G, Martinez LC, Barril PA, Ferreyra LJ, et al. (2008) Two instances of large genome profile picobirnavirus occurrence in Argentinian infants with diarrhea over a 26-year period (1977-2002). J Infect 56: 371-375.

1693. Giorgi PL, Catassi C, Coppa GV, Valentini V, Sbarbati A (1985) [New protagonists of infectious diarrhea in childhood. Pathogenetic and clinical aspects]. Minerva Pediatr 37: 29-48.

1694. Giovanelli M, Gupte GL, Sharif K, Mayer DA, Mirza DF (2008) Chronic rejection after combined liver and small bowel transplantation in a child with chronic intestinal pseudo-obstruction: a case report. Transplant Proc 40: 1763-1767.

1695. Girardet JP, Fontaine JL (1988) [Current treatment of acute diarrhea in infants]. Ann Pediatr (Paris) 35: 609-612.

1696. Girish R, Broor S, Dar L, Ghosh D (2002) Foodborne outbreak caused by a Norwalk-like virus in India. J Med Virol 67: 603-607.

1697. Givon-Lavi N, Greenberg D, Dagan R (2008) Comparison between two severity scoring scales commonly used in the evaluation of rotavirus gastroenteritis in children. Vaccine 26: 5798-5801.

1698. Gizatulina SS, Birger MO, Nikovskaia MI, Mastiukova Iu N, Potashova LA (1992) [Intestinal microflora in young children with rotavirus infection]. Zh Mikrobiol Epidemiol Immunobiol: 29-30.

1699. Gjersvik P (2006) [Vaccination against rotavirus in Uzbekistan?]. Tidsskr Nor Laegeforen 126: 2694.

1700. Gladstone BP, Iturriza-Gomara M, Ramani S, Monica B, Banerjee I, et al. (2008) Polymerase chain reaction in the detection of an 'outbreak' of asymptomatic viral infections in a community birth cohort in south India. Epidemiol Infect 136: 399-405.

1701. Glasgow JF, McClure BG, Connolly JH, O'Neill HJ (1978) Nosocomial rotavirus gastroenteritis in a neonatal nursery. Ulster Med J 47: 50-56.

1702. Glass RI (1999) Commentary: Reanalysis of the results of two rotavirus vaccine trials: an appraisal of the reappraisal. Pediatr Infect Dis J 18: 1006-1007.

1703. Glass RI (2006) New hope for defeating rotavirus. Sci Am 294: 46-51, 54-45.

1704. Glass RI, Belliot G, Ivanoff B, Parashar U, Gentsch J, et al. (1999) [Prevention of rotavirus diarrhea by vaccination]. Arch Pediatr 6 Suppl 2: 323s-326s.

1705. Glass RI, Bhan MK, Ray P, Bahl R, Parashar UD, et al. (2005) Development of candidate rotavirus vaccines derived from neonatal strains in India. J Infect Dis 192 Suppl 1: S30-35.

1706. Glass RI, Bresee J, Jiang B, Gentsch J, Ando T, et al. (2001) Gastroenteritis viruses: an overview. Novartis Found Symp 238: 5-19; discussion 19-25.

1707. Glass RI, Bresee J, Jiang B, Parashar U, Yee E, et al. (2006) Rotavirus and rotavirus vaccines. Adv Exp Med Biol 582: 45-54.

1708. Glass RI, Bresee JS, Parashar U, Miller M, Gentsch JR (1997) Rotavirus vaccines at the threshold. Nat Med 3: 1324-1325.

1709. Glass RI, Bresee JS, Parashar U, Turcios R, Fischer TK, et al. (2005) Rotavirus vaccines: past, present, and future. Arch Pediatr 12: 844-847.

1710. Glass RI, Bresee JS, Parashar UD, Holman RC, Gentsch JR (1999) First rotavirus vaccine licensed: is there really a need? Acta Paediatr Suppl 88: 2-8.

1711. Glass RI, Bresee JS, Parashar UD, Jiang B, Gentsch J (2004) The future of rotavirus vaccines: a major setback leads to new opportunities. Lancet 363: 1547-1550.

1712. Glass RI, Bresee JS, Turcios R, Fischer TK, Parashar UD, et al. (2005) Rotavirus vaccines: targeting the developing world. J Infect Dis 192 Suppl 1: S160-166.

1713. Glass RI, Gentsch J, Smith JC (1994) Rotavirus vaccines: success by reassortment? Science 265: 1389-1391.

1714. Glass RI, Gentsch JR, Ivanoff B (1996) New lessons for rotavirus vaccines. Science 272: 46-48.

1715. Glass RI, Keith J, Nakagomi O, Nakagomi T, Askaa J, et al. (1985) Nucleotide sequence of the structural glycoprotein VP7 gene of Nebraska calf diarrhea virus rotavirus: comparison with homologous genes from four strains of human and animal rotaviruses. Virology 141: 292-298.

1716. Glass RI, Kilgore PE, Holman RC, Jin S, Smith JC, et al. (1996) The epidemiology of rotavirus diarrhea in the United States: surveillance and estimates of disease burden. J Infect Dis 174 Suppl 1: S5-11.

1717. Glass RI, Parashar UD (2006) The promise of new rotavirus vaccines. N Engl J Med 354: 75-77.

1718. Glass RI, Parashar UD, Bresee JS, Turcios R, Fischer TK, et al. (2006) Rotavirus vaccines: current prospects and future challenges. Lancet 368: 323-332.

1719. Glass RI, Stoll BJ (1989) The protective effect of human milk against diarrhea. A review of studies from Bangladesh. Acta Paediatr Scand Suppl 351: 131-136.

1720. Glass RI, Stoll BJ, Wyatt RG, Hoshino Y, Banu H, et al. (1986) Observations questioning a protective role for breast-feeding in severe rotavirus diarrhea. Acta Paediatr Scand 75: 713-718.

1721. Gleizes O, Desselberger U, Tatochenko V, Rodrigo C, Salman N, et al. (2006) Nosocomial rotavirus infection in European countries: a review of the epidemiology, severity and economic burden of hospital-acquired rotavirus disease. Pediatr Infect Dis J 25: S12-21.

1722. Gofti-Laroche L, Gratacap-Cavallier B, Demanse D, Genoulaz O, Seigneurin JM, et al. (2003) Are waterborne astrovirus implicated in acute digestive morbidity (E.MI.R.A. study)? J Clin Virol 27: 74-82.

1723. Goh KT (1979) Epidemiology of diarrhoeal diseases in Singapore. Asian J Infect Dis 3: 47-56.

1724. Goh Rowland SG, Lloyd-Evans N, Williams K, Rowland MG (1985) The etiology of diarrhoea studied in the community in young urban Gambian children. J Diarrhoeal Dis Res 3: 7-13.

1725. Goldin BR (1998) Health benefits of probiotics. Br J Nutr 80: S203-207.

1726. Golding J, Emmett PM, Rogers IS (1997) Gastroenteritis, diarrhoea and breast feeding. Early Hum Dev 49 Suppl: S83-103.

1727. Goldwater PN, Bettelheim KA, Ellis-Pegler RB (1981) Escherichia coli in gastroenteritis of children in Auckland, New Zealand. J Hyg (Lond) 87: 413-419.

1728. Goldwater PN, Chrystie IL, Banatvala JE (1979) Rotaviruses and the respiratory tract. Br Med J 2: 1551.

1729. Goldwater PN, Rowland K, Thesinger M, Abbott K, Grieve A, et al. (2001) Rotavirus encephalopathy: pathogenesis reviewed. J Paediatr Child Health 37: 206-209.

1730. Goma Brufau AR, Vega Romero M, Martinez Ubieto P, Marco JJ, Salcedo Avizanda S, et al. (1988) [Epidemic outbreak of necrotizing enterocolitis coincident with an epidemic of neonatal rotavirus gastroenteritis]. An Esp Pediatr 29: 307-310.

1731. Gomara MI, Simpson R, Perault AM, Redpath C, Lorgelly P, et al. (2008) Structured surveillance of infantile gastroenteritis in East Anglia, UK: incidence of infection with common viral gastroenteric pathogens. Epidemiol Infect 136: 23-33.

1732. Gombold JL, Ramig RF (1986) Analysis of reassortment of genome segments in mice mixedly infected with rotaviruses SA11 and RRV. J Virol 57: 110-116.

1733. Gomes KA, Stupka JA, Gomez J, Parra GI (2007) Molecular characterization of calicivirus strains detected in outbreaks of gastroenteritis in Argentina. J Med Virol 79: 1703-1709.

1734. Gomes TA, Blake PA, Trabulsi LR (1989) Prevalence of Escherichia coli strains with localized, diffuse, and aggregative adherence to HeLa cells in infants with diarrhea and matched controls. J Clin Microbiol 27: 266-269.

1735. Gomes TA, Rassi V, MacDonald KL, Ramos SR, Trabulsi LR, et al. (1991) Enteropathogens associated with acute diarrheal disease in urban infants in Sao Paulo, Brazil. J Infect Dis 164: 331-337.

1736. Gomez de Caso JA, Franco Yague JA, Castillo Izquierdo JM, Ruiz Cosin C (1996) [Study of a disease outbreak in a home for the aged]. Aten Primaria 17: 211-214.

1737. Gomez J, Bercovich A, Alvarez A, Garrido D, Grinstein S (1990) [Seroepidemiology of human rotaviruses in a community of the Avellaneda district, Province of Buenos Aires]. Rev Argent Microbiol 22: 182-191.

1738. Gomez J, Estes MK, Matson DO, Bellinzoni R, Alvarez A, et al. (1990) Serotyping of human rotaviruses in Argentina by ELISA with monoclonal antibodies. Arch Virol 112: 249-259.

1739. Gomez JA, Bercovich JA, Grinstein S (1985) [Comparison of enzyme immunoassay, counterimmunoelectrophoresis and polyacrylamide gel electrophoresis for the diagnosis of rotaviruses]. Rev Argent Microbiol 17: 111-114.

1740. Gomez JA, Biscotti EL, Bercovich JA, Grinstein S (1986) Epidemiology of human rotaviruses in Argentina as determined by RNA genome electrophoresis. Intervirology 26: 174-180.

1741. Gomez JA, Nates S, De Castagnaro NR, Espul C, Borsa A, et al. (1998) Anticipating rotavirus vaccines: review of epidemiologic studies of rotavirus diarrhea in Argentina. Rev Panam Salud Publica 3: 69-78.

1742. Gomez JA, Sordo ME, Gentile A (2002) Epidemiologic patterns of diarrheal disease in Argentina: estimation of rotavirus disease burden. Pediatr Infect Dis J 21: 843-850.

1743. Gomez-Lado C, Garcia-Reboredo M, Monasterio-Corral L, Bravo-Mata M, Eiris-Punal J, et al. (2005) [Benign seizures associated with mild gastroenteritis: apropos of two cases]. An Pediatr (Barc) 63: 558-560.

1744. Gomwalk NE, Gosham LT, Umoh UJ (1990) Rotavirus gastroenteritis in pediatric diarrhoea in Jos, Nigeria. J Trop Pediatr 36: 52-55.

1745. Gomwalk NE, Umoh UJ, Gosham LT, Ahmad AA (1993) Influence of climatic factors on rotavirus infection among children with acute gastroenteritis in Zaria, northern Nigeria. J Trop Pediatr 39: 293-297.

1746. Goncalves JL, Lopes RC, Oliveira DB, Costa SS, Miranda MM, et al. (2005) In vitro anti-rotavirus activity of some medicinal plants used in Brazil against diarrhea. J Ethnopharmacol 99: 403-407.

1747. Gonzales-Loza Mdel R, Polanco-Marin GG, Puerto-Solis M (2000) [Identification of rotavirus associated to serotype G2 in Yucatan, Mexico]. Rev Soc Bras Med Trop 33: 553-557.

1748. Gonzalez AM, Jaimes MC, Cajiao I, Rojas OL, Cohen J, et al. (2003) Rotavirus-specific B cells induced by recent infection in adults and children predominantly express the intestinal homing receptor alpha4beta7. Virology 305: 93-105.

1749. Gonzalez AM, Nguyen TV, Azevedo MS, Jeong K, Agarib F, et al. (2004) Antibody responses to human rotavirus (HRV) in gnotobiotic pigs following a new prime/boost vaccine strategy using oral attenuated HRV priming and intranasal VP2/6 rotavirus-like particle (VLP) boosting with ISCOM. Clin Exp Immunol 135: 361-372.

1750. Gonzalez FS, Sordo ME, Rowensztein G, Sabbag L, Roussos A, et al. (1999) [Rotavirus diarrhea. Impact in a pediatric hospital of Buenos Aires]. Medicina (B Aires) 59: 321-326.

1751. Gonzalez GG, Pujol FH, Liprandi F, Deibis L, Ludert JE (1998) Prevalence of enteric viruses in human immunodeficiency virus seropositive patients in Venezuela. J Med Virol 55: 288-292.

1752. Gonzalez P, Sanches A, Rivera P, Jimenez C, Hernandez F (1997) Rotavirus and coronavirus outbreak: etiology of annual diarrhea in Costa Rican children. Rev Biol Trop 45: 989-991.

1753. Gonzalez R, Franco M, Sarmiento L, Romero M, Schael IP (2005) Serum IgA levels induced by rotavirus natural infection, but not following immunization with the RRV-TV vaccine (Rotashield), correlate with protection. J Med Virol 76: 608-612.

1754. Gonzalez-Carretero P, Noguera A, Fortuny C (2006) Rotavirus gastroenteritis leading to secondary bacteremia in previously healthy infants. Pediatrics 118: 2255-2256; author reply 2256-2257.

1755. Gonzalez-Losa Mdel R, Rodriguez-Angulo E, Puerto-Solis M, Noguchi H (2008) [Living with animals and rotavirus]. Salud Publica Mex 50: 271.

1756. Gonzalez-Losa MR, Puerto-Solis M, Polanco-Marin GG, Peniche-Rodriguez R, Puerto FI (1994) [Frequency of serotype G rotavirus isolated from children with diarrhea in Merida, Yucatan, Mexico]. Rev Invest Clin 46: 215-219.

1757. Goodgame RW (1999) Viral infections of the gastrointestinal tract. Curr Gastroenterol Rep 1: 292-300.

1758. Goodgame RW (2001) Viral causes of diarrhea. Gastroenterol Clin North Am 30: 779-795.

1759. Goossens LM, Standaert B, Hartwig N, Hovels AM, Al MJ (2008) The cost-utility of rotavirus vaccination with Rotarix (RIX4414) in the Netherlands. Vaccine 26: 1118-1127.

1760. Gorbach SL (2000) Probiotics and gastrointestinal health. Am J Gastroenterol 95: S2-4.

1761. Gorbach SL (2002) Probiotics in the third millennium. Dig Liver Dis 34 Suppl 2: S2-7.

1762. Gorbachev EN, Verbov VN, Artiukhov AI, Noskov FS (1988) [Comparative evaluation of methods for diagnosing rotavirus gastroenteritis]. Vopr Virusol 33: 763-767.

1763. Gorbachev EN, Verbov VN, Artiukhov AI, Noskov FS (1989) [The use of commercial immunoenzyme and latex preparations for the demonstration of human rotavirus antigens]. Zh Mikrobiol Epidemiol Immunobiol: 68-73.

1764. Gorbachev EN, Verbov VN, Artiukhov AN, Noskov FS (1989) [Immunoenzyme analysis and latex agglutination reaction: use in diagnosing rotavirus gastroenteritis]. Vopr Virusol 34: 109-113.

1765. Gorbachev EN, Verbov VN, Galko NV, Makarova NG, Vashukova SS (1986) [Immunoenzyme analysis in the diagnosis of human rotavirus gastroenteritis (methodological aspects)]. Vopr Virusol 31: 743-746.

1766. Gordon AG (1982) Rotavirus infections and the sompe syndrome. J Infect Dis 146: 117-118.

1767. Gorrell RJ, Palombo EA (1996) Use of non-radioactive probes for VP4 typing of human rotaviruses. J Virol Methods 61: 59-64.

1768. Gorziglia M, Green K, Nishikawa K, Taniguchi K, Jones R, et al. (1988) Sequence of the fourth gene of human rotaviruses recovered from asymptomatic or symptomatic infections. J Virol 62: 2978-2984.

1769. Gorziglia M, Hoshino Y, Buckler-White A, Blumentals I, Glass R, et al. (1986) Conservation of amino acid sequence of VP8 and cleavage region of 84-kDa outer capsid protein among rotaviruses recovered from asymptomatic neonatal infection. Proc Natl Acad Sci U S A 83: 7039-7043.

1770. Gorziglia M, Larralde G, Kapikian AZ, Chanock RM (1990) Antigenic relationships among human rotaviruses as determined by outer capsid protein VP4. Proc Natl Acad Sci U S A 87: 7155-7159.

1771. Gorziglia M, Nishikawa K, Hoshino Y, Taniguchi K (1990) Similarity of the outer capsid protein VP4 of the Gottfried strain of porcine rotavirus to that of asymptomatic human rotavirus strains. J Virol 64: 414-418.

1772. Gosciniak G, Sobieszczanska B, Grzybek-Hryncewicz K (1990) [Rotavirus diarrhea in children hospitalized in Wroclaw clinics]. Przegl Lek 47: 682-685.

1773. Gothefors L, Wadell G, Juto P, Taniguchi K, Kapikian AZ, et al. (1989) Prolonged efficacy of rhesus rotavirus vaccine in Swedish children. J Infect Dis 159: 753-757.

1774. Gothefors L, Young C, Backman M (2008) [Rotavirus infection in children--Swedish data in a European perspective. A prospective observational study shows burden in health care]. Lakartidningen 105: 1181-1185.

1775. Goto Y, Kurogi H, Inaba Y, Matumoto M (1986) Sequential isolation of rotavirus from individual calves. Vet Microbiol 11: 177-184.

1776. Goudeau A, Sambourg M, Pinon G, Denis F (1986) [Infectious viral diarrheas. Comparison of research methods for rotaviruses]. Ann Biol Clin (Paris) 44: 406-409.

1777. Gouedard H, Chastel C, Quillien MC, Castel Y (1981) [Rotavirus and acute gastroenteritis in childhood: a one-year survey in a department of general pediatrics (author's transl)]. Ann Pediatr (Paris) 28: 403-407.

1778. Gouet P, Contrepois M, Dubourguier HC, Riou Y, Scherrer R, et al. (1978) The experimental production of diarrhoea in colostrum deprived axenic and gnotoxenic calves with enteropathogenic Escherichia coli, rotavirus, coronavirus and in a combined infection of rotavirus and E. coli. Ann Rech Vet 9: 433-440.

1779. Gough RE, Cox WJ, Devoy J (1992) Isolation and identification of rotavirus from racing pigeons. Vet Rec 130: 273.

1780. Gouvea V, de Castro L, Timenetsky MC, Greenberg H, Santos N (1994) Rotavirus serotype G5 associated with diarrhea in Brazilian children. J Clin Microbiol 32: 1408-1409.

1781. Gouvea V, Glass RI, Woods P, Taniguchi K, Clark HF, et al. (1990) Polymerase chain reaction amplification and typing of rotavirus nucleic acid from stool specimens. J Clin Microbiol 28: 276-282.

1782. Gouvea V, Ho MS, Glass R, Woods P, Forrester B, et al. (1990) Serotypes and electropherotypes of human rotavirus in the USA: 1987-1989. J Infect Dis 162: 362-367.

1783. Gouvea V, Lima RC, Linhares RE, Clark HF, Nosawa CM, et al. (1999) Identification of two lineages (WA-like and F45-like) within the major rotavirus genotype P[8]. Virus Res 59: 141-147.

1784. Gouvea V, Santos N (1999) Rotavirus serotype G5: an emerging cause of epidemic childhood diarrhea. Vaccine 17: 1291-1292.

1785. Gouvea V, Santos N, Timenetsky Mdo C (1994) Identification of bovine and porcine rotavirus G types by PCR. J Clin Microbiol 32: 1338-1340.

1786. Gouvea V, Santos N, Timenetsky Mdo C (1994) VP4 typing of bovine and porcine group A rotaviruses by PCR. J Clin Microbiol 32: 1333-1337.

1787. Gouvea V, Santos N, Timenetsky Mdo C, Estes MK (1994) Identification of Norwalk virus in artificially seeded shellfish and selected foods. J Virol Methods 48: 177-187.

1788. Gouvea VS, Alencar AA, Barth OM, de Castro L, Fialho AM, et al. (1986) Diarrhoea in mice infected with a human rotavirus. J Gen Virol 67 ( Pt 3): 577-581.

1789. Gouvea VS, de Castro L, Pereira HG (1987) A combined dot nitrocellulose-enzyme immunoassay for rotavirus and adenovirus. J Virol Methods 18: 57-65.

1790. Gouyon JB, Kohli E, Petion AM, Pothier P (1989) [Value of rapid diagnostic methods in rotavirus infection in the neonatal period]. Arch Fr Pediatr 46: 187-190.

1791. Goveia MG, DiNubile MJ, Dallas MJ, Heaton PM, Kuter BJ (2008) Efficacy of pentavalent human-bovine (WC3) reassortant rotavirus vaccine based on breastfeeding frequency. Pediatr Infect Dis J 27: 656-658.

1792. Goveia MG, Rodriguez ZM, Dallas MJ, Itzler RF, Boslego JW, et al. (2007) Safety and efficacy of the pentavalent human-bovine (WC3) reassortant rotavirus vaccine in healthy premature infants. Pediatr Infect Dis J 26: 1099-1104.

1793. Gracey M (1988) Gastro-enteritis in Australian children: studies on the aetiology of acute diarrhoea. Ann Trop Paediatr 8: 68-75.

1794. Gracey M, Burke V, Robinson J (1983) Patterns of intestinal infection in Australian Aboriginal children. Ann Trop Paediatr 3: 35-39.

1795. Gracey M, Phadke MA, Burke V, Raut SK, Singh B (1984) Aspirin in acute gastroenteritis: a clinical and microbiological study. J Pediatr Gastroenterol Nutr 3: 692-695.

1796. Gracheva NM, Avakov AA, Novikova AV, Shcherbakov IT, Blokhina TA (1989) [Sporadic Rotavirus gastroenteritis in adults]. Sov Med: 103-106.

1797. Gracheva NM, Leont'eva NI, Bondarenko VM, Fialkina SV, Konovalova GN, et al. (2003) [Clinical and microbiological features of acute enteric mixed infection caused by Hafnia alvei and rotavirus]. Zh Mikrobiol Epidemiol Immunobiol: 62-65.

1798. Graham DY, Dufour GR, Estes MK (1987) Minimal infective dose of rotavirus. Arch Virol 92: 261-271.

1799. Graham DY, Estes MK (1991) Pathogenesis and treatment of rotavirus diarrhea. Gastroenterology 101: 1140-1141.

1800. Graham DY, Sackman JW, Estes MK (1984) Pathogenesis of rotavirus-induced diarrhea. Preliminary studies in miniature swine piglet. Dig Dis Sci 29: 1028-1035.

1801. Graham J, Whorwell PJ, Machin D, Wright R (1981) Lymphocyte transformation to specific antigens associated with Crohn's disease. Hepatogastroenterology 28: 258-260.

1802. Grandien M, Sterner G, Kalin M, Engardt L (1990) Management of pregnant women with diarrhoea at term and of healthy carriers of infectious agents in stools at delivery. Scand J Infect Dis Suppl 71: 9-18.

1803. Grant JP (1989) Putting biomedical knowledge to use in the Third World. Ann N Y Acad Sci 569: xi-xvii.

1804. Granzow H, Schirrmeier H, Beyer J, Lange E (1988) [Morphologic studies of virus infection of the intestinal tract--virus replication and cytopathology in cell cultures and enterocytes in piglets. 1. Ultrastructure of intestinal epithelium without viral infiltration and in rotavirus infection]. Arch Exp Veterinarmed 42: 558-570.

1805. Grassano Morin A, de Champs C, Lafeuille H, Meyer M (2000) [Nosocomial intestinal infections in an infant ward. The importance of phone inquiries of the families]. Arch Pediatr 7: 1059-1063.

1806. Grassi T, De Donno A, Guido M, Gabutti G (2006) G-genotyping of rotaviruses in stool samples in Salento, Italy. J Prev Med Hyg 47: 138-141.

1807. Grassi T, De Donno A, Guido M, Gabutti G (2008) The epidemiology and disease burden of rotavirus infection in the Salento peninsula, Italy. Turk J Pediatr 50: 132-136.

1808. Gratacap-Cavallier B, Genoulaz O, Brengel-Pesce K, Soule H, Innocenti-Francillard P, et al. (2000) Detection of human and animal rotavirus sequences in drinking water. Appl Environ Microbiol 66: 2690-2692.

1809. Grauballe PC, Genner J, Meyling A, Hornsleth A (1977) [Acute gastroenteritis caused by rotavirus. A summary of Danish studies]. Ugeskr Laeger 139: 3047-3049.

1810. Grauballe PC, Genner J, Meyling A, Hornsleth A (1977) Rapid diagnosis of rotavirus infections: comparison of electron microscopy and immunoelectroosmophoresis for the detection of rotavirus in human infantile gastroenteritis. J Gen Virol 35: 203-218.

1811. Grauballe PC, Hornsleth A, Hjelt K, Krasilnikoff PA (1986) Detection by ELISA of immunoglobulin G subclass-specific antibody responses in rotavirus infections in children. J Med Virol 18: 277-281.

1812. Grauballe PC, Jarzabek Z (1984) Comparison of indirect double antibody and double antibody sandwich ELISA techniques with latex agglutination test for the diagnosis of human rotavirus infection. Acta Virol 28: 59-63.

1813. Grauballe PC, Vestergaard BF, Meyling A, Genner J (1981) Optimized enzyme-linked immunosorbent assay for detection of human and bovine rotavirus in stools: Comparison with electron-microscopy, immunoelectro-osmophoresis, and fluorescent antibody techniques. J Med Virol 7: 29-40.

1814. Gray J (1989) Gastrointestinal infection. Practitioner 233: 784-786.

1815. Gray J, Vesikari T, Van Damme P, Giaquinto C, Mrukowicz J, et al. (2008) Rotavirus. J Pediatr Gastroenterol Nutr 46 Suppl 2: S24-31.

1816. Grech V, Calvagna V, Falzon A, Mifsud A (2001) Fatal, rotavirus-associated myocarditis and pneumonitis in a 2-year-old boy. Ann Trop Paediatr 21: 147-148.

1817. Green J, Wright PA, Gallimore CI, Mitchell O, Morgan-Capner P, et al. (1998) The role of environmental contamination with small round structured viruses in a hospital outbreak investigated by reverse-transcriptase polymerase chain reaction assay. J Hosp Infect 39: 39-45.

1818. Green KY, Kapikian AZ (1992) Identification of VP7 epitopes associated with protection against human rotavirus illness or shedding in volunteers. J Virol 66: 548-553.

1819. Green KY, Taniguchi K, Mackow ER, Kapikian AZ (1990) Homotypic and heterotypic epitope-specific antibody responses in adult and infant rotavirus vaccinees: implications for vaccine development. J Infect Dis 161: 667-679.

1820. Greenberg BL, Sack RB, Salazar-Lindo E, Budge E, Gutierrez M, et al. (1991) Measles-associated diarrhea in hospitalized children in Lima, Peru: pathogenic agents and impact on growth. J Infect Dis 163: 495-502.

1821. Greenberg D, Givon-Lavi N, Newman N, Wheeler J, Cohen Z, et al. (2008) Intussusception in children in Southern Israel: disparity between 2 populations. Pediatr Infect Dis J 27: 236-240.

1822. Greenberg DE, Wilimas JA, Buckingham SC (2003) Hematologic findings in children with rotavirus-positive and -negative diarrhea. Pediatr Hematol Oncol 20: 453-456.

1823. Greenberg H, McAuliffe V, Valdesuso J, Wyatt R, Flores J, et al. (1983) Serological analysis of the subgroup protein of rotavirus, using monoclonal antibodies. Infect Immun 39: 91-99.

1824. Greenberg HB (1993) Rotavirus vaccination--current status. A brief summary. Ann N Y Acad Sci 700: 32-35.

1825. Greenberg HB, Clark HF, Offit PA (1994) Rotavirus pathology and pathophysiology. Curr Top Microbiol Immunol 185: 255-283.

1826. Greenberg HB, Gebhard RL, McClain CJ, Soltis RD, Kapikian AZ (1979) Antibodies to viral gastroenteritis viruses in Crohn's disease. Gastroenterology 76: 349-350.

1827. Greenberg HB, Matsui SM (1992) Astroviruses and caliciviruses: emerging enteric pathogens. Infect Agents Dis 1: 71-91.

1828. Greenberg HB, Valdesuso J, Kapikian AZ, Chanock RM, Wyatt RG, et al. (1979) Prevalence of antibody to the Norwalk virus in various countries. Infect Immun 26: 270-273.

1829. Greenberg HB, Valdesuso J, Yolken RH, Gangarosa E, Gary W, et al. (1979) Role of Norwalk virus in outbreaks of nonbacterial gastroenteritis. J Infect Dis 139: 564-568.

1830. Greenberg HB, Vo PT, Jones R (1986) Cultivation and characterization of three strains of murine rotavirus. J Virol 57: 585-590.

1831. Greenwood BM, Byass P, Greenwood AM, Hayes RJ, Menon A, et al. (1989) Lack of an association between acute gastroenteritis, acute respiratory infections and malaria in young Gambian children. Trans R Soc Trop Med Hyg 83: 595-598.

1832. Gregorio L, Sutton CL, Lee DA (1997) Central pontine myelinolysis in a previously healthy 4-year-old child with acute rotavirus gastroenteritis. Pediatrics 99: 738-743.

1833. Grehn M, Kunz J, Sigg P, Slongo R, Zbinden R (1990) Nosocomial rotavirus infections in neonates: means of prevention and control. J Perinat Med 18: 369-374.

1834. Grice AS, Lambden PR, Caul EO, Clarke IN (1994) Sequence conservation of the major outer capsid glycoprotein of human group C rotaviruses. J Med Virol 44: 166-171.

1835. Griffin DD, Fletcher M, Levy ME, Ching-Lee M, Nogami R, et al. (2002) Outbreaks of adult gastroenteritis traced to a single genotype of rotavirus. J Infect Dis 185: 1502-1505.

1836. Griffiths FH, Els HJ, Steele AD, Alexander JJ (1990) Tubular rotavirus structures in human stool. S Afr Med J 78: 171-172.

1837. Griffiths FH, Steele AD, Alexander JJ (1992) The molecular epidemiology of rotavirus-associated gastro-enteritis in the Transkei, southern Africa. Ann Trop Paediatr 12: 259-264.

1838. Griffiths RI, Anderson GF, Powe NR, Oliveras E, Herbert RJ, et al. (1995) Economic impact of immunization against rotavirus gastroenteritis. Evidence from a clinical trial. Arch Pediatr Adolesc Med 149: 407-414.

1839. Grillner L, Broberger U, Chrystie I, Ransjo U (1985) Rotavirus infections in newborns: an epidemiological and clinical study. Scand J Infect Dis 17: 349-355.

1840. Grimprel E (2001) [Epidemiology of infant bronchiolitis in France]. Arch Pediatr 8 Suppl 1: 83S-92S.

1841. Grimprel E, Parez N, Gault E, Garbarg-Chenon A, Begue P (2001) [Acute diarrhea and rotavirus infection in the child: assessment of data from emergency care and and the microbiology laboratory of the Armand-Trousseau (Paris) Hospital between 1988 and 2001]. Arch Pediatr 8: 1318-1324.

1842. Grimwood K (2005) Unraveling rotaviruses, oral vaccines and intussusception. J Paediatr Child Health 41: 471-472.

1843. Grimwood K, Bines JE (2007) Rotavirus vaccines must perform in low-income countries too. Lancet 370: 1739-1740.

1844. Grimwood K, Buttery JP (2007) Clinical update: rotavirus gastroenteritis and its prevention. Lancet 370: 302-304.

1845. Grimwood K, Carzino R, Barnes GL, Bishop RF (1995) Patients with enteric adenovirus gastroenteritis admitted to an Australian pediatric teaching hospital from 1981 to 1992. J Clin Microbiol 33: 131-136.

1846. Grimwood K, Coakley JC, Hudson IL, Bishop RF, Barnes GL (1988) Serum aspartate aminotransferase levels after rotavirus gastroenteritis. J Pediatr 112: 597-600.

1847. Grimwood K, Huang QS, Cohet C, Gosling IA, Hook SM, et al. (2006) Rotavirus hospitalisation in New Zealand children under 3 years of age. J Paediatr Child Health 42: 196-203.

1848. Grimwood K, Lambert SB (2009) Rotavirus vaccines: Opportunities and challenges. Hum Vaccin 5.

1849. Grimwood K, Lund JC, Coulson BS, Hudson IL, Bishop RF, et al. (1988) Comparison of serum and mucosal antibody responses following severe acute rotavirus gastroenteritis in young children. J Clin Microbiol 26: 732-738.

1850. Grisaru-Soen G, Engelhard D, Pearl S, Schlesinger Y, Shtein M, et al. (2008) [Hospitalizations associated with rotavirus gastroenteritis in Israel--a retrospective study]. Harefuah 147: 8-11, 96.

1851. Groothuis JR, Berman S, Chapman J (1986) Effect of carbohydrate ingested on outcome in infants with mild gastroenteritis. J Pediatr 108: 903-906.

1852. Grosfeld JL, Rescorla FJ, West KW (1986) Short bowel syndrome in infancy and childhood. Analysis of survival in 60 patients. Am J Surg 151: 41-46.

1853. Grunow JE, Dunton SF, Waner JL (1985) Human rotavirus-like particles in a hepatic abscess. J Pediatr 106: 73-76.

1854. Gu Y, Gu Q, Kodama H, Mueller WE, Ushijima H (2000) Development of antirotavirus agents in Asia. Pediatr Int 42: 440-447.

1855. Guandalini S (1989) Overview of childhood acute diarrhoea in Europe: implications for oral rehydration therapy. Acta Paediatr Scand Suppl 364: 5-12.

1856. Guandalini S (2002) The treatment of acute diarrhea in the third millennium: a pediatrician's perspective. Acta Gastroenterol Belg 65: 33-36.

1857. Guandalini S (2008) Acute diarrhea in children in europe: do we know how to treat it? J Pediatr Gastroenterol Nutr 46 Suppl 2: S77-80.

1858. Guandalini S (2008) Probiotics for children with diarrhea: an update. J Clin Gastroenterol 42 Suppl 2: S53-57.

1859. Guandalini S, Pensabene L, Zikri MA, Dias JA, Casali LG, et al. (2000) Lactobacillus GG administered in oral rehydration solution to children with acute diarrhea: a multicenter European trial. J Pediatr Gastroenterol Nutr 30: 54-60.

1860. Guardado JA, Clara WA, Turcios RM, Fuentes RA, Valencia D, et al. (2004) Rotavirus in El Salvador: an outbreak, surveillance and estimates of disease burden, 2000-2002. Pediatr Infect Dis J 23: S156-160.

1861. Guarino A (1996) Active treatment of viral diarrhea with passive immunotherapy. J Pediatr Gastroenterol Nutr 23: 337.

1862. Guarino A (2008) Foreword: ESPGHAN/ESPID evidence-based guidelines for the management of acute gastroenteritis in children in Europe. J Pediatr Gastroenterol Nutr 46 Suppl 2: vii-viii.

1863. Guarino A, Albano F, Canani RB, Bruzzese E (2002) HIV, fatal rotavirus infection, and treatment options. Lancet 359: 74.

1864. Guarino A, Canani RB, Russo S, Albano F, Canani MB, et al. (1994) Oral immunoglobulins for treatment of acute rotaviral gastroenteritis. Pediatrics 93: 12-16.

1865. Guarino A, Canani RB, Spagnuolo MI, Albano F, Di Benedetto L (1997) Oral bacterial therapy reduces the duration of symptoms and of viral excretion in children with mild diarrhea. J Pediatr Gastroenterol Nutr 25: 516-519.

1866. Guarino A, Casola A, Bruzzese E, Saini M, Nitsch L, et al. (1996) Human serum immunoglobulin counteracts rotaviral infection in Caco-2 cells. Pediatr Res 40: 881-887.

1867. Guarino A, Guandalini S, Albano F, Mascia A, De Ritis G, et al. (1991) Enteral immunoglobulins for treatment of protracted rotaviral diarrhea. Pediatr Infect Dis J 10: 612-614.

1868. Guarino A, Lo Vecchio A, Canani RB (2009) Probiotics as prevention and treatment for diarrhea. Curr Opin Gastroenterol 25: 18-23.

1869. Guarino A, Russo S, Castaldo A, Spagnuolo MI, Tarallo L, et al. (1996) Passive immunotherapy for rotavirus-induced diarrhoea in children with HIV infection. Aids 10: 1176-1178.

1870. Guarino A, Spagnuolo MI, Russo S, Albano F, Guandalini S, et al. (1995) Etiology and risk factors of severe and protracted diarrhea. J Pediatr Gastroenterol Nutr 20: 173-178.

1871. Guarner F (2002) [The colon as an organ: habitat of bacterial flora]. Nutr Hosp 17 Suppl 2: 7-10.

1872. Gudkov VG, Rytik PG, Shumko VV, Virinskaya AS, Nepljuev IV, et al. (1991) Adaptation of human rotavirus to cell culture and characterization of the isolated strain. Acta Virol 35: 81-85.

1873. Gueddana N, Khadraoui S, Hamza B, Chamakh N, Jaraya S, et al. (1984) [Rotavirus and acute gastroenteritis in children]. Tunis Med 62: 37-41.

1874. Gueguen C, Maga A, McCrae MA, Bataillon G (1996) Caprine and bovine B rotaviruses in western France: group identification by Northern hybridization. Vet Res 27: 171-176.

1875. Guerin N (1985) Aetiological forms. Child Trop: 10.

1876. Guerin-Danan C, Andrieux C, Popot F, Charpilienne A, Vaissade P, et al. (1997) Pattern of metabolism and composition of the fecal microflora in infants 10 to 18 months old from day care centers. J Pediatr Gastroenterol Nutr 25: 281-289.

1877. Guerin-Danan C, Meslin JC, Chambard A, Charpilienne A, Relano P, et al. (2001) Food supplementation with milk fermented by Lactobacillus casei DN-114 001 protects suckling rats from rotavirus-associated diarrhea. J Nutr 131: 111-117.

1878. Guerin-Danan C, Meslin JC, Lambre F, Charpilienne A, Serezat M, et al. (1998) Development of a heterologous model in germfree suckling rats for studies of rotavirus diarrhea. J Virol 72: 9298-9302.

1879. Guerrant RL, Kirchhoff LV, Shields DS, Nations MK, Leslie J, et al. (1983) Prospective study of diarrheal illnesses in northeastern Brazil: patterns of disease, nutritional impact, etiologies, and risk factors. J Infect Dis 148: 986-997.

1880. Guerrero ML, Moreno-Espinosa S, Tuz-Dzib F, Solis-Albino J, Ortega-Gallegos H, et al. (2004) Breastfeeding and natural colonization with Lactobacillus spp as protection against rotavirus-associated diarrhea. Adv Exp Med Biol 554: 451-455.

1881. Guerrero ML, Noel JS, Mitchell DK, Calva JJ, Morrow AL, et al. (1998) A prospective study of astrovirus diarrhea of infancy in Mexico City. Pediatr Infect Dis J 17: 723-727.

1882. Guimaraes MA, Chagas V, Nozawa C, Takiya C, Maia PC, et al. (1994) Experimental infection of newborn mice with murine EDIM rotavirus. Braz J Med Biol Res 27: 671-675.

1883. Guiraldes E, Trivino X, Hodgson MI, Quintana JC, Quintana C (1995) Treatment of acute infantile diarrhoea with a commercial rice-based oral rehydration solution. J Diarrhoeal Dis Res 13: 207-211.

1884. Guiscafre H, Gonzalez S, Parra R, Lemus H, Alvarez MT, et al. (1988) [Strategies for improving the therapeutic patterns used in acute diarrhea in primary medical care units. III. Etiology and clinical picture of the cases studied]. Arch Invest Med (Mex) 19: 361-370.

1885. Gulati BR, Deepa R, Singh BK, Rao CD (2007) Diversity in Indian equine rotaviruses: identification of genotype G10,P6[1] and G1 strains and a new VP7 genotype (G16) strain in specimens from diarrheic foals in India. J Clin Microbiol 45: 972-978.

1886. Gulati BR, Maherchandani S, Patnayak DP, Pandey R (1997) RNA profile and structural protein analysis of rotaviruses isolated from diarrhoeal calves in India. J Diarrhoeal Dis Res 15: 12-16.

1887. Gulati BR, Nakagomi O, Koshimura Y, Nakagomi T, Pandey R (1999) Relative frequencies of G and P types among rotaviruses from Indian diarrheic cow and buffalo calves. J Clin Microbiol 37: 2074-2076.

1888. Gump D, Caul E, Eade O, Greenberg H, Kapikian A, et al. (1981) Lymphocytotoxic and microbial antibodies in Crohn's disease and matched controls. Antonie Van Leeuwenhoek 47: 455-464.

1889. Gunasena S, Nakagomi O, Isegawa Y, Kaga E, Nakagomi T, et al. (1993) Relative frequency of VP4 gene alleles among human rotaviruses recovered over a 10-year period (1982-1991) from Japanese children with diarrhea. J Clin Microbiol 31: 2195-2197.

1890. Gunn PR, Sato F, Powell KF, Bellamy AR, Napier JR, et al. (1985) Rotavirus neutralizing protein VP7: antigenic determinants investigated by sequence analysis and peptide synthesis. J Virol 54: 791-797.

1891. Gunnlaugsson G, Smedman L, da Silva MC, Grandien M, Zetterstrom R (1989) Rotavirus serology and breast-feeding in young children in rural Guinea-Bissau. Acta Paediatr Scand 78: 62-66.

1892. Gunson RN, Mackie P, Leanord A, Carman WF (2003) First rotavirus, now astrovirus: the evolving benefits of RT-PCR. Commun Dis Public Health 6: 66-67.

1893. Gunson RN, Miller J, Leonard A, Carman WF (2003) Importance of PCR in the diagnosis and understanding of rotavirus illness in the community. Commun Dis Public Health 6: 63-65.

1894. Gunther H, Otto P, Heilmann P (1983) [Studies of the occurrence of diarrhea pathogens in experimental calves of the Institute as a contribution to their epizootiology]. Arch Exp Veterinarmed 37: 293-297.

1895. Gunzburg S, Gracey M, Burke V, Chang B (1992) Epidemiology and microbiology of diarrhoea in young Aboriginal children in the Kimberley region of Western Australia. Epidemiol Infect 108: 67-76.

1896. Guo CT, Nakagomi O, Mochizuki M, Ishida H, Kiso M, et al. (1999) Ganglioside GM(1a) on the cell surface is involved in the infection by human rotavirus KUN and MO strains. J Biochem 126: 683-688.

1897. Guo TX, Fang RX, Li GH, Qian Y (2001) [A fusion protein of rotavirus VP6 and cholera toxin B subunit: expression in Escherichia coli and analysis of biological activities]. Sheng Wu Gong Cheng Xue Bao 17: 621-625.

1898. Gupta DN, Sen D, Saha MR, Sengupta PG, Sikder SN, et al. (1990) Report of an outbreak of diarrhoeal disease caused by cholera followed by rotavirus in Manipur. Indian J Public Health 34: 62-65.

1899. Gurgel RQ, Correia JB, Cuevas LE (2008) Effect of rotavirus vaccination on circulating virus strains. Lancet 371: 301-302.

1900. Gurgel RQ, Cunliffe NA, Nakagomi O, Cuevas LE (2008) Rotavirus genotypes circulating in Brazil before national rotavirus vaccination: a review. J Clin Virol 43: 1-8.

1901. Gurwith M, Hinde D, Gross R, Rowe B (1978) A prospective study of enteropathogenic Escherichia coli in endemic diarrheal disease. J Infect Dis 137: 292-297.

1902. Gurwith M, Wenman W, Gurwith D, Brunton J, Feltham S, et al. (1983) Diarrhea among infants and young children in Canada: a longitudinal study in three northern communities. J Infect Dis 147: 685-692.

1903. Gurwith MJ, Williams TW (1977) Gastroenteritis in children: a two-year review in Manitoba. I. Etiology. J Infect Dis 136: 239-247.

1904. Guscetti F, Hoop RK, Steiger R, Burgi E, Bertschinger HU, et al. (1994) [Diarrheal diseases in 1 to 4 week old suckling piglets from problem herds: microbial spectrum, histology, enzyme histochemistry]. Schweiz Arch Tierheilkd 136: 366-376.

1905. Gusmao RH, Mascarenhas JD, Gabbay YB, Linhares AC (1994) Nosocomial transmission of an avian-like rotavirus strain among children in Belem, Brazil. J Diarrhoeal Dis Res 12: 129-132.

1906. Gusmao RH, Mascarenhas JD, Gabbay YB, Lins-Lainson Z, Ramos FL, et al. (1999) Rotavirus subgroups, G serotypes, and electrophoretypes in cases of nosocomial infantile diarrhoea in Belem, Brazil. J Trop Pediatr 45: 81-86.

1907. Gusmao RH, Mascarenhas JD, Gabbay YB, Lins-Lainson Z, Ramos FL, et al. (1995) Rotaviruses as a cause of nosocomial, infantile diarrhoea in northern Brazil: pilot study. Mem Inst Oswaldo Cruz 90: 743-749.

1908. Gust ID, Pringle RC, Barnes GL, Davidson GP, Bishop RF (1977) Complement-fixing antibody response to rotavirus infection. J Clin Microbiol 5: 125-130.

1909. Gutierrez J, Soriano H, O'Ryan M, Avendano P, D'Ottone K, et al. (1989) [Pathogenic agents in acute non-enterocolic diarrheal syndrome]. Rev Chil Pediatr 60: 28-33.

1910. Gutierrez MF, Alvarado MV, Martinez E, Ajami NJ (2007) Presence of viral proteins in drinkable water--sufficient condition to consider water a vector of viral transmission? Water Res 41: 373-378.

1911. Gutierrez MF, Matiz A, Trespalacios AA, Parra M, Riano M, et al. (2006) Virus diversity of acute diarrhea in tropical highlands. Rev Latinoam Microbiol 48: 17-23.

1912. Gutierrez-Aguirre I, Banjac M, Steyer A, Poljsak-Prijatelj M, Peterka M, et al. (2008) Concentrating rotaviruses from water samples using monolithic chromatographic supports. J Chromatogr A.

1913. Gutierrez-Aguirre I, Steyer A, Boben J, Gruden K, Poljsak-Prijatelj M, et al. (2008) Sensitive detection of multiple rotavirus genotypes with a single reverse transcription-real-time quantitative PCR assay. J Clin Microbiol 46: 2547-2554.

1914. Gutzwiller A (2000) Glucose and galactose absorption after ingestion of milk containing hydrolysed lactose in calves with diarrhoea. J Vet Med A Physiol Pathol Clin Med 47: 495-500.

1915. Guyot J, Gonvers JJ, Pyndiah N, Heitz M (1984) [Value of fecal leukocyte studies in cases of acute diarrhea]. Schweiz Med Wochenschr 114: 634-636.

1916. Haber P, Chen RT, Zanardi LR, Mootrey GT, English R, et al. (2004) An analysis of rotavirus vaccine reports to the vaccine adverse event reporting system: more than intussusception alone? Pediatrics 113: e353-359.

1917. Haffejee IE (1990) Cow's milk-based formula, human milk, and soya feeds in acute infantile diarrhea: a therapeutic trial. J Pediatr Gastroenterol Nutr 10: 193-198.

1918. Haffejee IE (1990) Persistent diarrhoea following gastroenteritis. J Diarrhoeal Dis Res 8: 143-146.

1919. Haffejee IE (1991) Neonatal rotavirus infections. Rev Infect Dis 13: 957-962.

1920. Haffejee IE (1991) The pathophysiology, clinical features and management of rotavirus diarrhoea. Q J Med 79: 289-299.

1921. Haffejee IE (1995) The epidemiology of rotavirus infections: a global perspective. J Pediatr Gastroenterol Nutr 20: 275-286.

1922. Haffejee IE, Moosa A (1990) Rotavirus serology and excretion in hospitalized non-diarrhoeal patients. Ann Trop Paediatr 10: 173-178.

1923. Haffejee IE, Moosa A (1990) Rotavirus studies in Indian (Asian) South African infants with acute gastro-enteritis: I. Microbiological and epidemiological aspects. Ann Trop Paediatr 10: 165-172.

1924. Haffejee IE, Moosa A (1990) Rotavirus studies in Indian (Asian) South African infants with acute gastro-enteritis: II. Clinical aspects and outcome. Ann Trop Paediatr 10: 245-254.

1925. Haffejee IE, Moosa A, Windsor I (1990) Circulating and breast-milk anti-rotaviral antibodies and neonatal rotavirus infections: a maternal-neonatal study. Ann Trop Paediatr 10: 3-14.

1926. Haider K, Huq MI, Hossain A, Shahid NS, Holmes IH (1985) Electropherotypes of ds-RNA of rotavirus in infants and young children with gastroenteritis in Bangladesh. J Diarrhoeal Dis Res 3: 219-222.

1927. Haigh JC, Mackintosh C, Griffin F (2002) Viral, parasitic and prion diseases of farmed deer and bison. Rev Sci Tech 21: 219-248.

1928. Haikala OJ, Kokkonen JO, Leinonen MK, Nurmi T, Mantyjarvi R, et al. (1983) Rapid detection of rotavirus in stool by latex agglutination: comparison with radioimmunoassay and electron microscopy and clinical evaluation of the test. J Med Virol 11: 91-97.

1929. Hakim SL, Gan CC, Malkit K, Azian MN, Chong CK, et al. (2007) Parasitic infections among Orang Asli (aborigine) in the Cameron Highlands, Malaysia. Southeast Asian J Trop Med Public Health 38: 415-419.

1930. Halaihel N, Lievin V, Alvarado F, Vasseur M (2000) Rotavirus infection impairs intestinal brush-border membrane Na(+)-solute cotransport activities in young rabbits. Am J Physiol Gastrointest Liver Physiol 279: G587-596.

1931. Halaihel N, Lievin V, Ball JM, Estes MK, Alvarado F, et al. (2000) Direct inhibitory effect of rotavirus NSP4(114-135) peptide on the Na(+)-D-glucose symporter of rabbit intestinal brush border membrane. J Virol 74: 9464-9470.

1932. Hall GA (1987) Comparative pathology of infection by novel diarrhoea viruses. Ciba Found Symp 128: 192-217.

1933. Hall GA, Bridger JC, Chandler RL, Woode GN (1976) Gnotobiotic piglets experimentally infected with neonatal calf diarrhoea reovirus-like agent (Rotavirus). Vet Pathol 13: 197-210.

1934. Hall GA, Bridger JC, Parsons KR, Cook R (1993) Variation in rotavirus virulence: a comparison of pathogenesis in calves between two rotaviruses of different virulence. Vet Pathol 30: 223-233.

1935. Hall GA, Reynolds DJ, Chanter N, Morgan JH, Parsons KR, et al. (1985) Dysentery caused by Escherichia coli (S102-9) in calves: natural and experimental disease. Vet Pathol 22: 156-163.

1936. Hall GA, Reynolds DJ, Parsons KR, Bland AP, Morgan JH (1988) Pathology of calves with diarrhoea in southern Britain. Res Vet Sci 45: 240-250.

1937. Hallstrom M, Vesikari T, Janas M, Ikonen S, Tammela O (2001) Screening of rotavirus and adenovirus infections during prolonged hospitalization in a neonatal unit. Acta Paediatr 90: 1196-1198.

1938. Halvorsrud J, Orstavik I (1980) An epidemic of rotavirus-associated gastroenteritis in a nursing home for the elderly. Scand J Infect Dis 12: 161-164.

1939. Hamann HP, Herbst W, Krauss H (1989) [Comparative studies of the detection of rotavirus in fecal samples of calves with diarrhea with the latex test "Slidex Rota-Kit 2" and electron microscopy]. Berl Munch Tierarztl Wochenschr 102: 346-347.

1940. Hamano M, Kuzuya M, Fujii R, Ogura H, Mori T, et al. (1999) Outbreak of acute gastroenteritis caused by human group C rotavirus in a primary school. Jpn J Infect Dis 52: 170-171.

1941. Hambraeus BA, Hambraeus LE, Wadell G (1989) Animal model of rotavirus infection in rabbits--protection obtained without shedding of viral antigen. Arch Virol 107: 237-251.

1942. Hamilton JR (1985) Treatment of acute diarrhea. Pediatr Clin North Am 32: 419-427.

1943. Hamilton JR (1985) Viral diarrhea. Pediatr Ann 14: 25-28.

1944. Hamilton JR (1988) Viral enteritis. Pediatr Clin North Am 35: 89-101.

1945. Hamilton JR (1990) The pathophysiological basis for viral diarrhea: a progress report. J Pediatr Gastroenterol Nutr 11: 150-154.

1946. Hamir AN, Morin M, Rupprecht CE (1990) Rotaviral enteritis in a raccoon. J Wildl Dis 26: 262-264.

1947. Hammami S, Castro AE, Osburn BI (1990) Comparison of polyacrylamide gel electrophoresis, an enzyme-linked-immunosorbent assay, and an agglutination test for the direct identification of bovine rotavirus from feces and coelectrophoresis of viral RNAs. J Vet Diagn Invest 2: 184-190.

1948. Hammami S, Sawyer MM, Castro AE, Holmberg CA, Osburn BI (1989) Detection of rotavirus in fecal samples from calves by a cell culture indirect immunofluorescence, an Ag-capture ELISA, a tissue culture ELISA, and a commercial Ag-capture ELISA. J Vet Diagn Invest 1: 72-73.

1949. Hammarstrom L, Weiner CK (2008) Targeted antibodies in dairy-based products. Adv Exp Med Biol 606: 321-343.

1950. Hammarstrom L, Weiner CK (2008) Targeted antibodies in dairy-based products. Adv Exp Med Biol 606: 321-343.

1951. Hammitt MC, Bueschel DM, Keel MK, Glock RD, Cuneo P, et al. (2007) A possible role for Clostridium difficile in the etiology of calf enteritis. Vet Microbiol.

1952. Hammitt MC, Bueschel DM, Keel MK, Glock RD, Cuneo P, et al. (2008) A possible role for Clostridium difficile in the etiology of calf enteritis. Vet Microbiol 127: 343-352.

1953. Hammond L, Papadopoulos S, Johnson CF, MaWhinney S, Nelson B, et al. (2002) Use of an Internet-based community surveillance network to predict seasonal communicable disease morbidity. Pediatrics 109: 414-418.

1954. Hampson DJ, Hinton M, Kidder DE (1985) Coliform numbers in the stomach and small intestine of healthy pigs following weaning at three weeks of age. J Comp Pathol 95: 353-362.

1955. Han AM, U KM, Hlaing T, Bozikov J, Dezelic G, et al. (1985) Epidemiological model of acute bacterial and viral diarrhoeal diseases. J Diarrhoeal Dis Res 3: 65-72.

1956. Hanlon P, Hanlon L, Marsh V, Byass P, Shenton F, et al. (1987) Epidemiology of rotavirus in a periurban Gambian community. Ann Trop Paediatr 7: 238-243.

1957. Hansman GS, Doan LT, Kguyen TA, Okitsu S, Katayama K, et al. (2004) Detection of norovirus and sapovirus infection among children with gastroenteritis in Ho Chi Minh City, Vietnam. Arch Virol 149: 1673-1688.

1958. Haque R, Mondal D, Kirkpatrick BD, Akther S, Farr BM, et al. (2003) Epidemiologic and clinical characteristics of acute diarrhea with emphasis on Entamoeba histolytica infections in preschool children in an urban slum of Dhaka, Bangladesh. Am J Trop Med Hyg 69: 398-405.

1959. Haralambiev H, Georgiev G, Mitov B, Tsvetkov P (1987) Application of an attenuated vaccine, RoCo-81, against viral enteritis of calves. Acta Vet Hung 35: 469-473.

1960. Harp JA, Myers LL, Rich JE, Gates NL (1981) Role of Salmonella arizonae and other infective agents in enteric disease of lambs. Am J Vet Res 42: 596-599.

1961. Harries JT (1982) Mechanisms and mediators of intestinal secretion in the small intestine. J Pediatr Gastroenterol Nutr 1: 575-582.

1962. Harris AM, Chowdhury F, Begum YA, Khan AI, Faruque AS, et al. (2008) Shifting prevalence of major diarrheal pathogens in patients seeking hospital care during floods in 1998, 2004, and 2007 in Dhaka, Bangladesh. Am J Trop Med Hyg 79: 708-714.

1963. Harris CC, Yolken RH, Krokan H, Hsu IC (1979) Ultrasensitive enzymatic radioimmunoassay: application to detection of cholera toxin and rotavirus. Proc Natl Acad Sci U S A 76: 5336-5339.

1964. Harris JP, Jit M, Cooper D, Edmunds WJ (2007) Evaluating rotavirus vaccination in England and Wales. Part I. Estimating the burden of disease. Vaccine 25: 3962-3970.

1965. Harris L, Tudehope D (1983) Necrotizing enterocolitis and human rotavirus. Med J Aust 1: 104-105.

1966. Harrison MS (1998) Rotavirus: an overview--from discovery to vaccine. Pediatr Nurs 24: 317-323.

1967. Hart CA, Cunliffe NA (1999) Viral gastroenteritis. Curr Opin Infect Dis 12: 447-457.

1968. Hartmann H, Gunther H, Meyer H, Kreutzer B, Henniger A (1980) [Studies of carbohydrate absorption in clinically healthy and diarrheal calves]. Arch Exp Veterinarmed 34: 527-541.

1969. Haschek B, Klein D, Benetka V, Herrera C, Sommerfeld-Stur I, et al. (2006) Detection of bovine torovirus in neonatal calf diarrhoea in Lower Austria and Styria (Austria). J Vet Med B Infect Dis Vet Public Health 53: 160-165.

1970. Hasegawa A, Inouye S, Matsuno S, Yamaoka K, Eko R, et al. (1984) Isolation of human rotaviruses with a distinct RNA electrophoretic pattern from Indonesia. Microbiol Immunol 28: 719-722.

1971. Hasegawa A, Matsuno S, Inouye S, Kono R, Tsurukubo Y, et al. (1982) Isolation of human rotaviruses in primary cultures of monkey kidney cells. J Clin Microbiol 16: 387-390.

1972. Hasegawa A, Mukoyama A, Akatani K, Ikegami N, Urasawa S, et al. (1993) Serotyping of human rotavirus by enzyme immuno-assay with monoclonal antibodies. Jpn J Med Sci Biol 46: 221-226.

1973. Hashizume M, Armstrong B, Wagatsuma Y, Faruque AS, Hayashi T, et al. (2007) Rotavirus infections and climate variability in Dhaka, Bangladesh: a time-series analysis. Epidemiol Infect: 1-9.

1974. Hashizume M, Armstrong B, Wagatsuma Y, Faruque AS, Hayashi T, et al. (2008) Rotavirus infections and climate variability in Dhaka, Bangladesh: a time-series analysis. Epidemiol Infect 136: 1281-1289.

1975. Hassan EM, el-Meneza SA, el-Rashidy Z, Rashad R, Rabie S, et al. (1989) Detection of enteropathogens in diarrhoeal diseases among malnourished Egyptian infant and children. J Egypt Public Health Assoc 64: 461-474.

1976. Hasso SA, Pandey R, Thapliyal DC, Al-Samarrae SA (1983) Rotavirus infection of young calves in Iraq. Acta Virol 27: 93.

1977. Hasso SA, Pandey R, Zenad MM (1985) Clinical and biochemical aspects of bovine rotavirus infection in Iraq. J Diarrhoeal Dis Res 3: 88-91.

1978. Hatta H, Tsuda K, Akachi S, Kim M, Yamamoto T, et al. (1993) Oral passive immunization effect of anti-human rotavirus IgY and its behavior against proteolytic enzymes. Biosci Biotechnol Biochem 57: 1077-1081.

1979. Hattori H, Torii S, Nagafuji H, Tabata Y, Hata A (1992) Benign acute myositis associated with rotavirus gastroenteritis. J Pediatr 121: 748-749.

1980. Haug KW, Orstavik I, Kvelstad G (1978) Rotavirus infections in families. A clinical and virological study. Scand J Infect Dis 10: 265-269.

1981. Haupt RM, Isikci O, Kimble WL, Sotos GL, Fu J (2006) Physicians' knowledge and attitudes about rotavirus gastroenteritis and rotavirus vaccine. Pediatr Ann 35: 54-61.

1982. Hayashi M, Nagai M, Hayakawa Y, Takeuchi K, Tsunemitsu H (2001) Outbreak of diarrhoea and milk drop in cows infected with bovine group B rotavirus. Vet Rec 149: 331-332.

1983. Haynes JS, Reynolds DL, Fagerland JA, Fix AS (1994) Morphogenesis of enteric lesions induced by group D rotavirus in ringneck pheasant chicks (Phasianus colchicus). Vet Pathol 31: 74-81.

1984. He KW, Lin JH, Ding ZD, He JH, Jiang JY, et al. (1992) Serotyping and antigenic comparison of some animal rotaviruses isolated in China. Zentralbl Veterinarmed B 39: 299-302.

1985. He ST, He FZ, Wu CR (1996) [Clinical and experimental study on treatment of rotaviral enteritis with qiwei baizhu powder]. Zhongguo Zhong Xi Yi Jie He Za Zhi 16: 132-135.

1986. He ST, He FZ, Wu CR, Li SX, Liu WX, et al. (2001) Treatment of rotaviral gastroenteritis with Qiwei Baizhu powder. World J Gastroenterol 7: 735-740.

1987. He XQ, Cheng L, Li W, Xie XM, Ma M, et al. (2008) Detection and distribution of rotavirus in municipal sewage treatment plants (STPs) and surface water in Beijing. J Environ Sci Health A Tox Hazard Subst Environ Eng 43: 424-429.

1988. Heaton PM, Goveia MG, Miller JM, Offit P, Clark HF (2005) Development of a pentavalent rotavirus vaccine against prevalent serotypes of rotavirus gastroenteritis. J Infect Dis 192 Suppl 1: S17-21.

1989. Heczko PB, Strus M, Jawien M, Szymanski H (2005) [Medical applications of probiotics]. Wiad Lek 58: 640-646.

1990. Hedberg CW, Osterholm MT (1993) Outbreaks of food-borne and waterborne viral gastroenteritis. Clin Microbiol Rev 6: 199-210.

1991. Heffernan R, Mostashari F, Das D, Karpati A, Kulldorff M, et al. (2004) Syndromic surveillance in public health practice, New York City. Emerg Infect Dis 10: 858-864.

1992. Hegazy MM, Maklouf LM, El Hamshary EM, Dawoud HA, Eida AM (2008) Protein profile and morphometry of cultured human Blastocystis hominis from children with gastroenteritis and healthy ones. J Egypt Soc Parasitol 38: 453-464.

1993. Heijbel H, Slaine K, Seigel B, Wall P, McNabb SJ, et al. (1987) Outbreak of diarrhea in a day care center with spread to household members: the role of Cryptosporidium. Pediatr Infect Dis J 6: 532-535.

1994. Heiman EM, McDonald SM, Barro M, Taraporewala ZF, Bar-Magen T, et al. (2008) Group A human rotavirus genomics: evidence that gene constellations are influenced by viral protein interactions. J Virol 82: 11106-11116.

1995. Heinrich HW, Liebermann H, Hahnefeld H, Schirrmeier H (1983) [Experiences obtained thus far in the immunoprophylaxis of rotavirus infections in calves]. Arch Exp Veterinarmed 37: 317-322.

1996. Hellard ME, Sinclair MI, Hogg GG, Fairley CK (2000) Prevalence of enteric pathogens among community based asymptomatic individuals. J Gastroenterol Hepatol 15: 290-293.

1997. Henderson RH, Keja J, Hayden G, Galazka A, Clements J, et al. (1988) Immunizing the children of the world: progress and prospects. Bull World Health Organ 66: 535-543.

1998. Hendricks MK, Cuevas LE, Hart CA (1995) Rotavirus diarrhoea in Thai infants and children. Ann Trop Paediatr 15: 147-152.

1999. Hennig-Pauka I, Stelljes I, Waldmann KH (2003) Studies on the effect of specific egg antibodies against Escherichia coli infections in piglets. Dtsch Tierarztl Wochenschr 110: 49-54.

2000. Henry FJ, Bartholomew RK (1990) Epidemiology and transmission of rotavirus infections and diarrhoea in St. Lucia, West Indies. West Indian Med J 39: 205-212.

2001. Herbst W, Lange H, Danner K, Krauss H, Schliesser T (1989) [Electron microscopic virus detection in fecal samples from swine with enteric diseases between 1981 and 1987]. Dtsch Tierarztl Wochenschr 96: 294, 296.

2002. Herbst W, Lange H, Zschock M, Krauss H (1986) [Detection of calf rotavirus in the Rotascreen latex test and electron microscopy--a comparative study]. Dtsch Tierarztl Wochenschr 93: 317-319.

2003. Herbst W, Zschock M, Hamann HP, Lange H, Weiss R, et al. (1987) [The occurrence of rotavirus and fimbriae-bearing E. coli types in foals with diarrhea]. Berl Munch Tierarztl Wochenschr 100: 364-366.

2004. Hernandez F, Alvarez RM, Oviedo MT (1987) [Epizootiology of bovine diarrheas in Costa Rica]. Rev Latinoam Microbiol 29: 113-117.

2005. Hernandez F, Mata L, Lopez ME, Lizano C (1977) [Rotavirus in children with severe malnutrition]. Bol Med Hosp Infant Mex 34: 993-1000.

2006. Hernandez F, Monge R, Jimenez C, Taylor L (1997) Rotavirus and hepatitis A virus in market lettuce (Latuca sativa) in Costa Rica. Int J Food Microbiol 37: 221-223.

2007. Hernandez O, Fernandez J, Valenzuela S, Sandino AM, Pizarro J, et al. (1992) Respiratory syncytial virus detection by dot blot hybridization with a nonradioactive synthetic oligo deoxynucleotide probe. J Med Virol 37: 165-169.

2008. Hernandez-Chavarria F (2002) [A view of tropical biology through the electron microscope]. Rev Biol Trop 50: 927-940.

2009. Hernandez-Henriquez H, Soto-Escalona A, Anez F, Blitz de Dorfman L (1987) [Prolonged excretion of rotavirus in infants with diarrhea]. Bol Med Hosp Infant Mex 44: 650-653.

2010. Herrmann B, Lawrenz-Wolf B, Seewald C, Selb B, Wehinger H (1993) [5th day convulsions of the newborn infant in rotavirus infections]. Monatsschr Kinderheilkd 141: 120-123.

2011. Herrmann JE, Blacklow NR, Perron-Henry DM, Clements E, Taylor DN, et al. (1988) Incidence of enteric adenoviruses among children in Thailand and the significance of these viruses in gastroenteritis. J Clin Microbiol 26: 1783-1786.

2012. Herrmann JE, Chen SC, Fynan EF, Santoro JC, Greenberg HB, et al. (1996) Protection against rotavirus infections by DNA vaccination. J Infect Dis 174 Suppl 1: S93-97.

2013. Herrmann JE, Nowak NA, Blacklow NR (1985) Detection of Norwalk virus in stools by enzyme immunoassay. J Med Virol 17: 127-133.

2014. Herrmann JE, Taylor DN, Echeverria P, Blacklow NR (1991) Astroviruses as a cause of gastroenteritis in children. N Engl J Med 324: 1757-1760.

2015. Hess RG, Bachmann PA (1981) Distribution of antibodies to rotavirus in serum and lacteal secretions of naturally infected swine and their suckling pigs. Am J Vet Res 42: 1149-1152.

2016. Hess RG, Bachmann PA, Baljer G, Mayr A, Pospischil A, et al. (1984) Synergism in experimental mixed infections of newborn colostrum-deprived calves with bovine rotavirus and enterotoxigenic Escherichia coli (ETEC). Zentralbl Veterinarmed B 31: 585-596.

2017. Heyman M, Corthier G, Petit A, Meslin JC, Moreau C, et al. (1987) Intestinal absorption of macromolecules during viral enteritis: an experimental study on rotavirus-infected conventional and germ-free mice. Pediatr Res 22: 72-78.

2018. Heyse JF, Kuter BJ, Dallas MJ, Heaton P (2008) Evaluating the safety of a rotavirus vaccine: the REST of the story. Clin Trials 5: 131-139.

2019. Hieber JP, Shelton S, Nelson JD, Leon J, Mohs E (1978) Comparison of human rotavirus disease in tropical and temperate settings. Am J Dis Child 132: 853-858.

2020. Hien BT, Trang do T, Scheutz F, Cam PD, Molbak K, et al. (2007) Diarrhoeagenic Escherichia coli and other causes of childhood diarrhoea: a case-control study in children living in a wastewater-use area in Hanoi, Vietnam. J Med Microbiol 56: 1086-1096.

2021. Hildreth C, Thomas M, Ridgway GL (1981) Rotavirus infection in an obstetric unit. Br Med J (Clin Res Ed) 282: 231.

2022. Hilpert H, Brussow H, Mietens C, Sidoti J, Lerner L, et al. (1987) Use of bovine milk concentrate containing antibody to rotavirus to treat rotavirus gastroenteritis in infants. J Infect Dis 156: 158-166.

2023. Hines ME, 2nd, Styer EL, Baldwin CA, Cole JR, Jr. (1995) Combined adenovirus and rotavirus enteritis with Escherichia coli septicemia in an emu chick (Dromaius novaehollandiae). Avian Dis 39: 646-651.

2024. Hiraga C, Kodama Y, Sugiyama T, Ichikawa Y (1990) Prevention of human rotavirus infection with chicken egg yolk immunoglobulins containing rotavirus antibody in cat. Kansenshogaku Zasshi 64: 118-123.

2025. Hiramoto I, Nakagomi T, Nakagomi O (2005) Population-based estimates of the cumulative risk of hospitalization potentially associated with rotavirus diarrhea among children living in two cities in Akita Prefecture, Japan. Jpn J Infect Dis 58: 73-77.

2026. Hiruma M, Ide S, Kume T (1985) A case of neonatal calf diarrhea associated with natural infection with rotavirus. Nippon Juigaku Zasshi 47: 517-521.

2027. Hjelt K (1988) Acute rotavirus gastroenteritis in children. Clinical, epidemiological and immunological aspects. Dan Med Bull 35: 222-236.

2028. Hjelt K (1991) [Nosocomial virus infections in pediatric departments. Rotavirus and respiratory syncytial virus]. Ugeskr Laeger 153: 2102-2104.

2029. Hjelt K, Grauballe PC, Andersen L, Schiotz PO, Howitz P, et al. (1986) Antibody response in serum and intestine in children up to six months after a naturally acquired rotavirus gastroenteritis. J Pediatr Gastroenterol Nutr 5: 74-80.

2030. Hjelt K, Grauballe PC, Paerregaard A, Nielsen OH, Krasilnikoff PA (1987) Protective effect of preexisting rotavirus-specific immunoglobulin A against naturally acquired rotavirus infection in children. J Med Virol 21: 39-47.

2031. Hjelt K, Grauballe PC, Schiotz PO, Andersen L, Krasilnikoff PA (1985) Intestinal and serum immune response to a naturally acquired rotavirus gastroenteritis in children. J Pediatr Gastroenterol Nutr 4: 60-66.

2032. Hjelt K, Krasilnikoff PA, Grauballe PC (1984) Incidence of hospitalisation and outpatient clinical visits caused by rotavirus and non-rotavirus acute gastroenteritis. A study of children living in the southern district of Copenhagen County. Dan Med Bull 31: 249-251.

2033. Hjelt K, Krasilnikoff PA, Grauballe PC, Rasmussen SW (1985) Clinical features in hospitalised children with acute gastroenteritis. Does the rotavirus syndrome exist? Acta Paediatr Scand 74: 96-101.

2034. Hjelt K, Krasilnikoff PA, Grauballe PC, Rasmussen SW (1985) Nosocomial acute gastroenteritis in a paediatric department, with special reference to rotavirus infections. Acta Paediatr Scand 74: 89-95.

2035. Hjelt K, Nielson OH, Paerregaard A, Grauballe PC, Krasilnikoff PA (1987) Acute gastroenteritis in children attending day-care centres with special reference to rotavirus infections. II. Clinical manifestations. Acta Paediatr Scand 76: 763-768.

2036. Hjelt K, Paerregaard A, Nielsen OH, Grauballe PC, Gaarslev K, et al. (1987) Acute gastroenteritis in children attending day-care centres with special reference to rotavirus infections. I. Aetiology and epidemiologic aspects. Acta Paediatr Scand 76: 754-762.

2037. Ho AM, Nelson EA, Walker DG (2008) Rotavirus vaccination for Hong Kong children: an economic evaluation from the Hong Kong Government perspective. Arch Dis Child 93: 52-58.

2038. Ho AM, Nelson EA, Walker DG (2008) Rotavirus vaccination for Hong Kong children: an economic evaluation from the Hong Kong Government perspective. Arch Dis Child 93: 52-58.

2039. Ho L, Bradford BJ (1995) Hypernatremic dehydration and rotavirus enteritis. Clin Pediatr (Phila) 34: 440-441.

2040. Ho MS, Floyd RL, Glass RI, Pallansch MA, Jones B, et al. (1989) Simultaneous administration of rhesus rotavirus vaccine and oral poliovirus vaccine: immunogenicity and reactogenicity. Pediatr Infect Dis J 8: 692-696.

2041. Ho MS, Glass RI, Pinsky PF, Anderson LJ (1988) Rotavirus as a cause of diarrheal morbidity and mortality in the United States. J Infect Dis 158: 1112-1116.

2042. Hoblet KH, Saif LJ, Kohler EM, Theil KW, Bech-Nielsen S, et al. (1986) Efficacy of an orally administered modified-live porcine-origin rotavirus vaccine against postweaning diarrhea in pigs. Am J Vet Res 47: 1697-1703.

2043. Hochleitner BW, Bosmuller C, Nehoda H, Fruhwirt M, Simma B, et al. (2001) Increased tacrolimus levels during diarrhea. Transpl Int 14: 230-233.

2044. Hochwald C, Kivela L (1999) Rotavirus vaccine, live, oral, tetravalent (RotaShield). Pediatr Nurs 25: 203-204, 207.

2045. Hodes HL (1980) Gastroenteritis with special reference to rotavirus. Adv Pediatr 27: 195-245.

2046. Hodgins DC, Kang SY, deArriba L, Parreno V, Ward LA, et al. (1999) Effects of maternal antibodies on protection and development of antibody responses to human rotavirus in gnotobiotic pigs. J Virol 73: 186-197.

2047. Hoet AE, Nielsen PR, Hasoksuz M, Thomas C, Wittum TE, et al. (2003) Detection of bovine torovirus and other enteric pathogens in feces from diarrhea cases in cattle. J Vet Diagn Invest 15: 205-212.

2048. Hoet AE, Smiley J, Thomas C, Nielsen PR, Wittum TE, et al. (2003) Association of enteric shedding of bovine torovirus (Breda virus) and other enteropathogens with diarrhea in veal calves. Am J Vet Res 64: 485-490.

2049. Hofmann W (1983) [Therapy and prevention of rotavirus and coronavirus infections in calves]. Berl Munch Tierarztl Wochenschr 96: 453-457.

2050. Hofmann W (1987) [How often must maternal vaccination be repeated for the prophylaxis of rotavirus and coronavirus infections (neonatal diarrhea) in the calf?]. Dtsch Tierarztl Wochenschr 94: 298-301.

2051. Hofmann W, Danner K, Seeger K (1985) [Initial experiences in the treatment of virus-induced diarrheas in calves with interferon produced by genetic engineering]. Dtsch Tierarztl Wochenschr 92: 278-280.

2052. Hoge CW, Echeverria P, Rajah R, Jacobs J, Malthouse S, et al. (1995) Prevalence of Cyclospora species and other enteric pathogens among children less than 5 years of age in Nepal. J Clin Microbiol 33: 3058-3060.

2053. Hoh H, Presser W, Wigand R (1983) [Nosocomial rotavirus infections in adults]. Dtsch Med Wochenschr 108: 1586-1591.

2054. Hohl R (2007) [2006 immunization plan--details of rotavirus vaccination]. Wien Med Wochenschr 157: 133-135.

2055. Hohmann B, Holzwarth M, Saur G, Krautzberger W (1993) Neonatal necrotizing enterocolitis and co-existing defect of the intestinal musculature. Eur J Pediatr 152: 540-541.

2056. Hokkanen L, Poutiainen E, Valanne L, Salonen O, Iivanainen M, et al. (1996) Cognitive impairment after acute encephalitis: comparison of herpes simplex and other aetiologies. J Neurol Neurosurg Psychiatry 61: 478-484.

2057. Holdaway MD (1983) Gastroenteritis in children. N Z Med J 96: 89-91.

2058. Holdaway MD, Kalmakoff J, Schroeder BA, Wright GC, Todd BA, et al. (1982) Rotavirus infection in Otago: a serological study. N Z Med J 95: 110-112.

2059. Holdaway MD, Kalmakoff J, Todd BA, Jennings LC (1985) Rotavirus infection in a small community. J Med Virol 15: 389-398.

2060. Holdaway MD, Todd BA, Schroeder BA, Kalmakoff J (1982) Rotavirus infection in New Zealand. N Z Med J 95: 67-69.

2061. Holland RE (1990) Some infectious causes of diarrhea in young farm animals. Clin Microbiol Rev 3: 345-375.

2062. Hollander R (1987) [Rotavirus infection in the older child and adult]. Immun Infekt 15: 216-219.

2063. Holley DL, Allen SD, Barnett BB (1984) Enzyme-linked immunosorbent assay, using monoclonal antibody, to detect enterotoxic Escherichia coli K99 antigen in feces of dairy calves. Am J Vet Res 45: 2613-2616.

2064. Holm S, Andersson Y, Gothefors L, Lindberg T (1992) Increased protein absorption after acute gastroenteritis in children. Acta Paediatr 81: 585-588.

2065. Holman RC, Parashar UD, Clarke MJ, Kaufman SF, Glass RI (1999) Trends in diarrhea-associated hospitalizations among American Indian and Alaska native children, 1980-1995. Pediatrics 103: E11.

2066. Holmes IH (1979) Viral gastroenteritis. Prog Med Virol 25: 1-36.

2067. Holmes IH, Rodger SM, Schnagl RD, Ruck BJ, Gust ID, et al. (1976) Is lactase the receptor and uncoating enzyme for infantile enteritis (rota) viruses? Lancet 1: 1387-1388.

2068. Holmes WR (1999) Rotavirus infection and rates of hospitalisation for acute gastroenteritis in young children in Australia, 1993-1996. Med J Aust 170: 189-190.

2069. Holmgren J, Czerkinsky C, Eriksson K, Mharandi A (2003) Mucosal immunisation and adjuvants: a brief overview of recent advances and challenges. Vaccine 21 Suppl 2: S89-95.

2070. Holmgren J, Svennerholm AM (1982) Pathogenic mechanisms and new perspectives in the treatment and prevention of enteric infections. Scand J Gastroenterol Suppl 77: 47-59.

2071. Holtz TH, Nettleman MD (1990) Emporiatrics: diarrhea in travelers. Infect Control Hosp Epidemiol 11: 606-610.

2072. Holzel H, Cubitt DW, McSwiggan DA, Sanderson PJ, Church J (1980) An outbreak of rotavirus infection among adults in a cardiology ward. J Infect 2: 33-37.

2073. Homma A (2001) [Feasibility of a vaccine against rotavirus for developing countries]. Rev Panam Salud Publica 9: 57-59.

2074. Honeyman MC, Coulson BS, Stone NL, Gellert SA, Goldwater PN, et al. (2000) Association between rotavirus infection and pancreatic islet autoimmunity in children at risk of developing type 1 diabetes. Diabetes 49: 1319-1324.

2075. Hong SK, Lee SG, Lee SA, Kang JH, Lee JH, et al. (2007) Characterization of a G11,P[4] strain of human rotavirus isolated in South Korea. J Clin Microbiol 45: 3759-3761.

2076. Hong T (1996) Human group B rotavirus: adult diarrhea rotavirus. Chin Med J (Engl) 109: 11-12.

2077. Hongou K, Konishi T, Yagi S, Araki K, Miyawaki T (1998) Rotavirus encephalitis mimicking afebrile benign convulsions in infants. Pediatr Neurol 18: 354-357.

2078. Honma H, Ushijimma H, Takagi M, Kitamiura T (1990) Evaluation of a new enzyme immunoassay (TESTPACK ROTAVIRUS) for diagnosis of viral gastroenteritis. Kansenshogaku Zasshi 64: 174-178.

2079. Honma S, Nakata S, Numata K, Kogawa K, Yamashita T, et al. (1998) Epidemiological study of prevalence of genogroup II human calicivirus (Mexico virus) infections in Japan and Southeast Asia as determined by enzyme-linked immunosorbent assays. J Clin Microbiol 36: 2481-2484.

2080. Hopkins RS, Gaspard GB, Williams FP, Jr., Karlin RJ, Cukor G, et al. (1984) A community waterborne gastroenteritis outbreak: evidence for rotavirus as the agent. Am J Public Health 74: 263-265.

2081. Hoppen T, Keller KM, Stolte M, Rister M (1993) [Clostridium difficile in early childhood ulcerative pancolitis]. Monatsschr Kinderheilkd 141: 474-477.

2082. Hoque SS, Faruque AS, Mahalanabis D, Hasnat A (1994) Infectious agents causing acute watery diarrhoea in infants and young children in Bangladesh and their public health implications. J Trop Pediatr 40: 351-354.

2083. Horacek J, Kubes V (1983) [Epidemic acute gastroenteritis in newborn infants with the immunoelectronoptic detection of virus]. Arch Exp Veterinarmed 37: 87-89.

2084. Horacek J, Kubes V, Kapla J, Stepanova V, Brychova E, et al. (1983) [Immunoelectron microscopic detection of viruses in newborn infants with acute gastroenteritis]. Cesk Epidemiol Mikrobiol Imunol 32: 134-137.

2085. Hori H, Akpedonu P, Armah G, Aryeetey M, Yartey J, et al. (1996) Enteric pathogens in severe forms of acute gastroenteritis in Ghanaian children. Acta Paediatr Jpn 38: 672-676.

2086. Horie Y, Masamune O, Nakagomi O (1997) Three major alleles of rotavirus NSP4 proteins identified by sequence analysis. J Gen Virol 78 ( Pt 9): 2341-2346.

2087. Horie Y, Nakagomi O, Koshimura Y, Nakagomi T, Suzuki Y, et al. (1999) Diarrhea induction by rotavirus NSP4 in the homologous mouse model system. Virology 262: 398-407.

2088. Horst H, Kohlhase B (1986) [Asymptomatic excretors of rotavirus]. Infection 14: 163-166.

2089. Horton S, Sanghvi T, Phillips M, Fiedler J, Perez-Escamilla R, et al. (1996) Breastfeeding promotion and priority setting in health. Health Policy Plan 11: 156-168.

2090. Hoshino T, Hosokawa N, Kumasaka K, Kawano K (2001) [The relationship of serum mitochondrial creatine kinase and rotavirus gastroenteritis in pediatric patients]. Rinsho Byori 49: 597-602.

2091. Hoshino T, Hosokawa N, Yanai M, Kumasaka K, Kawano K (2001) [A study of serum mitochondrial enzymes(mCK, mAST, mMDH) in rotavirus and adenovirus gastroenteritis in pediatric patients]. Rinsho Byori 49: 1157-1161.

2092. Hoshino Y, Gorziglia M, Valdesuso J, Askaa J, Glass RI, et al. (1987) An equine rotavirus (FI-14 strain) which bears both subgroup I and subgroup II specificities on its VP6. Virology 157: 488-496.

2093. Hoshino Y, Honma S, Jones RW, Ross J, Santos N, et al. (2005) A porcine G9 rotavirus strain shares neutralization and VP7 phylogenetic sequence lineage 3 characteristics with contemporary human G9 rotavirus strains. Virology 332: 177-188.

2094. Hoshino Y, Honma S, Jones RW, Santos N, Nakagomi O, et al. (2006) A rotavirus strain isolated from pig-tailed macaque (Macaca nemestrina) with diarrhea bears a P6[1]:G8 specificity. Virology 345: 1-12.

2095. Hoshino Y, Jones RW, Chanock RM, Kapikian AZ (2002) Generation and characterization of six single VP4 gene substitution reassortant rotavirus vaccine candidates: each bears a single human rotavirus VP4 gene encoding P serotype 1A[8] or 1B[4] and the remaining 10 genes of rhesus monkey rotavirus MMU18006 or bovine rotavirus UK. Vaccine 20: 3576-3584.

2096. Hoshino Y, Jones RW, Ross J, Honma S, Santos N, et al. (2004) Rotavirus serotype G9 strains belonging to VP7 gene phylogenetic sequence lineage 1 may be more suitable for serotype G9 vaccine candidates than those belonging to lineage 2 or 3. J Virol 78: 7795-7802.

2097. Hoshino Y, Jones RW, Ross J, Kapikian AZ (2003) Construction and characterization of rhesus monkey rotavirus (MMU18006)- or bovine rotavirus (UK)-based serotype G5, G8, G9 or G10 single VP7 gene substitution reassortant candidate vaccines. Vaccine 21: 3003-3010.

2098. Hoshino Y, Jones RW, Ross J, Kapikian AZ (2005) Porcine rotavirus strain Gottfried-based human rotavirus candidate vaccines: construction and characterization. Vaccine 23: 3791-3799.

2099. Hoshino Y, Jones RW, Ross J, Santos N, Kapikian AZ (2003) Human rotavirus strains bearing VP4 gene P[6] allele recovered from asymptomatic or symptomatic infections share similar, if not identical, VP4 neutralization specificities. Virology 316: 1-8.

2100. Hoshino Y, Kapikian AZ (1994) Rotavirus vaccine development for the prevention of severe diarrhea in infants and young children. Trends Microbiol 2: 242-249.

2101. Hoshino Y, Kapikian AZ (1996) Classification of rotavirus VP4 and VP7 serotypes. Arch Virol Suppl 12: 99-111.

2102. Hoshino Y, Kapikian AZ (2000) Rotavirus serotypes: classification and importance in epidemiology, immunity, and vaccine development. J Health Popul Nutr 18: 5-14.

2103. Hoshino Y, Kapikian AZ, Chanock RM (1994) Selection of cold-adapted mutants of human rotaviruses that exhibit various degrees of growth restriction in vitro. J Virol 68: 7598-7602.

2104. Hoshino Y, Saif LJ, Kang SY, Sereno MM, Chen WK, et al. (1995) Identification of group A rotavirus genes associated with virulence of a porcine rotavirus and host range restriction of a human rotavirus in the gnotobiotic piglet model. Virology 209: 274-280.

2105. Hoshino Y, Saif LJ, Sereno MM, Chanock RM, Kapikian AZ (1988) Infection immunity of piglets to either VP3 or VP7 outer capsid protein confers resistance to challenge with a virulent rotavirus bearing the corresponding antigen. J Virol 62: 744-748.

2106. Hoshino Y, Sereno MM, Midthun K, Flores J, Chanock RM, et al. (1987) Analysis by plaque reduction neutralization assay of intertypic rotaviruses suggests that gene reassortment occurs in vivo. J Clin Microbiol 25: 290-294.

2107. Hoshino Y, Wagner M, Yan XY, Perez-Schael I, Kapikian AZ (2003) Horizontal transmission of rhesus monkey rotavirus-based quadrivalent vaccine during a phase 3 clinical trial in Caracas, Venezuela. J Infect Dis 187: 791-800.

2108. Hoshino Y, Wyatt RG, Greenberg HB, Kalica AR, Flores J, et al. (1983) Isolation and characterization of an equine rotavirus. J Clin Microbiol 18: 585-591.

2109. Hoshino Y, Wyatt RG, Greenberg HB, Kalica AR, Flores J, et al. (1983) Isolation, propagation, and characterization of a second equine rotavirus serotype. Infect Immun 41: 1031-1037.

2110. Hou Z, Huang Y, Huan Y, Pang W, Meng M, et al. (2008) Anti-NSP4 antibody can block rotavirus-induced diarrhea in mice. J Pediatr Gastroenterol Nutr 46: 376-385.

2111. Houly C, Grunert B, Werchau H, Mietens C, Brussow H, et al. (1984) Epidemiology of rotavirus gastroenteritis in infants from the area of Bochum as revealed by electrophoresis of genome RNA. Eur J Pediatr 143: 128-132.

2112. Houly CA, Uchoa MM, Zaidan AM, Gomes-Neto A, de-Oliveira FM, et al. (1986) Electrophoretic study of the genome of human rotavirus from Maceio, Brazil. Braz J Med Biol Res 19: 33-37.

2113. House JA (1978) Economic impact of rotavirus and other neonatal disease agents of animals. J Am Vet Med Assoc 173: 573-576.

2114. Househam KC, Bowie DC, Mann MD, Bowie MD (1990) Factors influencing the duration of acute diarrheal disease in infancy. J Pediatr Gastroenterol Nutr 10: 37-40.

2115. Househam KC, Bowie MD (1988) Clinical features associated with enteropathogens causing acute infectious infantile diarrhoea. J Trop Pediatr 34: 94-96.

2116. Househam KC, Mann MD, Bowie MD (1988) Enteropathogens associated with acute infantile diarrhoea in Cape Town. S Afr Med J 73: 83-87.

2117. Howard P, Alexander ND, Atkinson A, Clegg AO, Gerega G, et al. (2000) Bacterial, viral and parasitic aetiology of paediatric diarrhoea in the highlands of Papua New Guinea. J Trop Pediatr 46: 10-14.

2118. Howe L, Sugiarto H, Squires RA (2008) Use of polymerase chain reaction for the differentiation of Group A bovine rotavirus G6, G8, and G10 genotypes in the North Island of New Zealand. N Z Vet J 56: 218-221.

2119. Hrdy DB (1982) Rotavirus antibodies in hanuman langurs (Presbytis entellus). J Med Primatol 11: 35-38.

2120. Hrdy DB (1987) Epidemiology of rotaviral infection in adults. Rev Infect Dis 9: 461-469.

2121. Hruska JF, Notter MF, Menegus MA, Steinhoff MC (1978) RNA polymerase associated with human rotaviruses in diarrhea stools. J Virol 26: 544-546.

2122. Hsu VP, Abdul Rahman HB, Wong SL, Ibrahim LH, Yusoff AF, et al. (2005) Estimates of the burden of rotavirus disease in Malaysia. J Infect Dis 192 Suppl 1: S80-86.

2123. Hsu VP, Staat MA, Roberts N, Thieman C, Bernstein DI, et al. (2005) Use of active surveillance to validate international classification of diseases code estimates of rotavirus hospitalizations in children. Pediatrics 115: 78-82.

2124. Hu CW (1985) [Rotavirus enteritis in infants and children in five child care centers in Hefei City--a one-year longitudinal survey]. Zhonghua Liu Xing Bing Xue Za Zhi 6: 333-335.

2125. Hu CW (1988) [Discovery of a new rotavirus in the feces of patients with acute diarrhea]. Zhonghua Yi Xue Za Zhi 68: 141-143, 112.

2126. Huang H, Schroeder F, Estes MK, McPherson T, Ball JM (2004) Interaction(s) of rotavirus non-structural protein 4 (NSP4) C-terminal peptides with model membranes. Biochem J 380: 723-733.

2127. Huang JA, Nagesha HS, Snodgrass DR, Holmes IH (1992) Molecular and serological analyses of two bovine rotaviruses (B-11 and B-60) causing calf scours in Australia. J Clin Microbiol 30: 85-92.

2128. Huang XL, Chen J, Yu YP, Chen LQ, Li ZY, et al. (2006) [Viraemia and extraintestinal involvement after rotavirus infection]. Zhejiang Da Xue Xue Bao Yi Xue Ban 35: 69-75.

2129. Huang YK, Qi Q, Hou ZL, Li HL, Wen GS, et al. (2005) [Analysis on molecular characteristic of VP7 and NSP4]. Zhonghua Liu Xing Bing Xue Za Zhi 26: 980-983.

2130. Huber AC, Yolken RH, Mader LC, Strandberg JD, Vonderfecht SL (1989) Pathology of infectious diarrhea of infant rats (IDIR) induced by an antigenically distinct rotavirus. Vet Pathol 26: 376-385.

2131. Hudson D (1981) Rota-coronavirus vaccination of pregnant cows. Mod Vet Pract 62: 626-628.

2132. Huet F, Allaert FA, Trancart A, Miadi-Fargier H, Trichard M, et al. (2008) [Economic evaluation of acute paediatric rotavirus gastroenteritis in France]. Arch Pediatr 15: 1159-1166.

2133. Huet F, Chouchane M, Cremillieux C, Aubert M, Caulin E, et al. (2008) [Prospective epidemiological study of rotavirus gastroenteritis in Europe (REVEAL study). Results in the French area of the study]. Arch Pediatr 15: 362-374.

2134. Huet F, Largeron N, Trichard M, Miadi-Fargier H, Jasso-Mosqueda G (2007) Burden of paediatric rotavirus gastroenteritis and potential benefits of a universal rotavirus vaccination programme with RotaTeq in France. Vaccine 25: 6348-6358.

2135. Huetink RE, van der Giessen JW, Noordhuizen JP, Ploeger HW (2001) Epidemiology of Cryptosporidium spp. and Giardia duodenalis on a dairy farm. Vet Parasitol 102: 53-67.

2136. Hughes JH, Tuomari AV, Mann DR, Hamparian VV (1984) Latex immunoassay for rapid detection of rotavirus. J Clin Microbiol 20: 441-447.

2137. Hughes JM, Rouse JD, Barada FA, Guerrant RL (1980) Etiology of summer diarrhea among the Navajo. Am J Trop Med Hyg 29: 613-619.

2138. Huh JW, Kim WH, Moon SG, Lee JB, Lim YH (2009) Viral etiology and incidence associated with acute gastroenteritis in a 5-year survey in Gyeonggi province, South Korea. J Clin Virol.

2139. Huh JW, Kim WH, Yoon MH, Lim YH (2009) Genotypic distribution of rotavirus strains causing severe gastroenteritis in Gyeonggi province, South Korea, from 2003 to 2005. Arch Virol 154: 167-170.

2140. Huicho L, Sanchez D, Contreras M, Paredes M, Murga H, et al. (1993) Occult blood and fecal leukocytes as screening tests in childhood infectious diarrhea: an old problem revisited. Pediatr Infect Dis J 12: 474-477.

2141. Huilan S, Zhen LG, Mathan MM, Mathew MM, Olarte J, et al. (1991) Etiology of acute diarrhoea among children in developing countries: a multicentre study in five countries. Bull World Health Organ 69: 549-555.

2142. Hull BP, Spence L, Bassett D, Swanston WH, Tikasingh ES (1982) The relative importance of rotavirus and other pathogens in the etiology of gastroenteritis in Trinidadian children. Am J Trop Med Hyg 31: 142-148.

2143. Hulten C, Demmers S (2002) Serum amyloid A (SAA) as an aid in the management of infectious disease in the foal: comparison with total leucocyte count, neutrophil count and fibrinogen. Equine Vet J 34: 693-698.

2144. Hung JJ, Wen HY, Yen MH, Chen HW, Yan DC, et al. (2003) Rotavirus gastroenteritis associated with afebrile convulsion in children: clinical analysis of 40 cases. Chang Gung Med J 26: 654-659.

2145. Hung LC, Wong SL, Chan LG, Rosli R, Ng AN, et al. (2006) Epidemiology and strain characterization of rotavirus diarrhea in Malaysia. Int J Infect Dis 10: 470-474.

2146. Hung T, Chen GM, Wang CG, Chou ZY, Chao TX, et al. (1983) Rotavirus-like agent in adult non-bacterial diarrhoea in China. Lancet 2: 1078-1079.

2147. Hung T, Chen GM, Wang CG, Fan RL, Yong RJ, et al. (1987) Seroepidemiology and molecular epidemiology of the Chinese rotavirus. Ciba Found Symp 128: 49-62.

2148. Hung T, Chen GM, Wang CG, Yao HL, Fang ZY, et al. (1984) Waterborne outbreak of rotavirus diarrhoea in adults in China caused by a novel rotavirus. Lancet 1: 1139-1142.

2149. Hung T, Fan RL, Wang CA, Chen GM, Chou DN, et al. (1985) Seroepidemiology of adult rotavirus. Lancet 2: 325-326.

2150. Hung TY, Liu MC, Hsu CF, Lin YC (2008) Rotavirus infection increases the risk of bacteremia in children with nontyphoid Salmonella gastroenteritis. Eur J Clin Microbiol Infect Dis.

2151. Hunt JB, Thillainayagam AV, Salim AF, Carnaby S, Elliott EJ, et al. (1992) Water and solute absorption from a new hypotonic oral rehydration solution: evaluation in human and animal perfusion models. Gut 33: 1652-1659.

2152. Huppertz HI, Soriano-Gabarro M, Grimprel E, Franco E, Mezner Z, et al. (2006) Intussusception among young children in Europe. Pediatr Infect Dis J 25: S22-29.

2153. Huq MI, Al Swailem AR, Fares S, Alim AR (1985) Studies on the etiologic agents of infantile diarrhea in Riyadh. Indian J Pediatr 52: 293-298.

2154. Huq MI, al-Ghamdi MA, Sibaii MS, al-Harfi R (1988) Incidence of asymptomatic rotavirus infection in neonates in the nursery of a children hospital in Damman, Saudi Arabia. East Afr Med J 65: 478-482.

2155. Huq MI, Rahman AS, Al-Sadiq A, Al-Shahri A, Alim AR (1987) Rotavirus as an important cause of diarrhoea in a hospital for children in Dammam, Saudi Arabia. Ann Trop Paediatr 7: 173-176.

2156. Hurtado O, Parwani AV, Tineo A, Lucchelli A, Saif LJ (1995) Serotypic analysis of group A bovine rotavirus field specimens using G1-, G2-, and G3-specific monoclonal antibodies in ELISA. J Vet Diagn Invest 7: 251-254.

2157. Husain M, Seth P, Broor S (1995) Detection of group A rotavirus by reverse transcriptase and polymerase chain reaction in feces from children with acute gastroenteritis. Arch Virol 140: 1225-1233.

2158. Husain M, Seth P, Dar L, Broor S (1996) Classification of rotavirus into G and P types with specimens from children with acute diarrhea in New Delhi, India. J Clin Microbiol 34: 1592-1594.

2159. Hussein AH, Cornaglia E, Saber MS, el-Azhary Y (1995) Prevalence of serotypes G6 and G10 group A rotaviruses in dairy calves in Quebec. Can J Vet Res 59: 235-237.

2160. Hussein HA, Parwani AV, Rosen BI, Lucchelli A, Saif LJ (1993) Detection of rotavirus serotypes G1, G2, G3, and G11 in feces of diarrheic calves by using polymerase chain reaction-derived cDNA probes. J Clin Microbiol 31: 2491-2496.

2161. Hyams JS, Krause PJ, Gleason PA (1981) Lactose malabsorption following rotavirus infection in young children. J Pediatr 99: 916-918.

2162. Hyser JM, Estes MK (2009) Rotavirus vaccines and pathogenesis: 2008. Curr Opin Gastroenterol 25: 36-43.

2163. Hyser JM, Zeng CQ, Beharry Z, Palzkill T, Estes MK (2007) Epitope mapping and use of epitope-specific antisera to characterize the VP5() binding site in rotavirus SA11 NSP4. Virology.

2164. Hyser JM, Zeng CQ, Beharry Z, Palzkill T, Estes MK (2008) Epitope mapping and use of epitope-specific antisera to characterize the VP5* binding site in rotavirus SA11 NSP4. Virology 373: 211-228.

2165. Ibrahim OS, Sunderland D, Hart CA (1990) Comparison of four methods for detection of rotavirus in faeces. Trop Doct 20: 30-32.

2166. Iglesias Escalera G, Usano Carrasco AI, Cueto Calvo E, Martinez Badas I, Guardia Nieto L, et al. (2005) [Benign afebrile convulsions due to rotavirus gastroenteritis]. An Pediatr (Barc) 63: 82-83.

2167. Ignatov G, Belchev L, Ignatova M, Tunkara A, Arnaudov D (1987) [Pathomorphological changes in the small intestine of pigs with rotavirus diarrhea]. Vet Med Nauki 24: 22-30.

2168. Ignatov G, Ignatova M, Tunkara A, Popov G (1987) [Etiological study of viral gastroenteritis in swine]. Vet Med Nauki 24: 10-18.

2169. Iijima Y, Iwamoto T, Nukuzuma S, Ohishi H, Hayashi K, et al. (2006) An outbreak of rotavirus infection among adults in an institution for rehabilitation: long-term residence in a closed community as a risk factor for rotavirus illness. Scand J Infect Dis 38: 490-496.

2170. Ijaz MK, Alharbi S, Uduman SA, Cheema Y, Sheek-Hussen MM, et al. (1994) Seasonality and prevalence of rotavirus in Al-Ain, United Arab Emirates. Clin Diagn Virol 2: 323-329.

2171. Ijaz MK, Attah-Poku SK, Redmond MJ, Parker MD, Sabara MI, et al. (1991) Heterotypic passive protection induced by synthetic peptides corresponding to VP7 and VP4 of bovine rotavirus. J Virol 65: 3106-3113.

2172. Ijaz MK, Dent D, Haines D, Babiuk LA (1989) Development of a murine model to study the pathogenesis of rotavirus infection. Exp Mol Pathol 51: 186-204.

2173. Ijaz MK, Sattar SA, Alkarmi T, Dar FK, Bhatti AR, et al. (1994) Studies on the survival of aerosolized bovine rotavirus (UK) and a murine rotavirus. Comp Immunol Microbiol Infect Dis 17: 91-98.

2174. Ijaz MK, Sattar SA, Johnson-Lussenburg CM, Springthorpe VS, Nair RC (1985) Effect of relative humidity, atmospheric temperature, and suspending medium on the airborne survival of human rotavirus. Can J Microbiol 31: 681-685.

2175. Ikegami N, Akatani K (1984) [Viral diarrhea]. Rinsho Byori 32: 715-725.

2176. Ikegami N, Akatani K (1993) [Present status of viral diarrhea in developing countries and the countermeasures]. Uirusu 43: 35-44.

2177. Imagawa H, Sekiguchi K, Anzai T, Fukunaga Y, Kanemaru T, et al. (1991) Epidemiology of equine rotavirus infection among foals in the breeding region. J Vet Med Sci 53: 1079-1080.

2178. Imagawa H, Tanaka T, Sekiguchi K, Fukunaga Y, Anzai T, et al. (1993) Electropherotypes, serotypes, and subgroups of equine rotaviruses isolated in Japan. Arch Virol 131: 169-176.

2179. Imagawa H, Wada R, Hirasawa K, Akiyama Y, Oda T (1984) Isolation of equine rotavirus in cell cultures from foals with diarrhea. Nippon Juigaku Zasshi 46: 1-9.

2180. Imai K, Otani K, Yanagihara K, Li Z, Futagi Y, et al. (1999) Ictal video-EEG recording of three partial seizures in a patient with the benign infantile convulsions associated with mild gastroenteritis. Epilepsia 40: 1455-1458.

2181. Imamura A, Puri P, O'Briain DS, Reen DJ (1992) Mucosal immune defence mechanisms in enterocolitis complicating Hirschsprung's disease. Gut 33: 801-806.

2182. Imamura Y, Hamada N, Nagai T, Shingu M (1992) [Detection and serotyping of HRVs collected from children with acute gastroenteritis in winter of 1986 to 1991]. Kansenshogaku Zasshi 66: 1404-1410.

2183. Imamura Y, Hamada N, Nagai T, Shingu M (1994) Detection and typing of human rotavirus in reference to repeated acute gastroenteritis in infants. Microbiol Immunol 38: 673-676.

2184. Ing D, Glass RI, LeBaron CW, Lew JF (1992) Laboratory-based surveillance for rotavirus United States, January 1989-May 1991. MMWR CDC Surveill Summ 41: 47-56.

2185. Inglis E, Kesson A, Newall AT, Macartney K, Macintyre CR (2008) The burden of rotavirus gastroenteritis in children presenting to a paediatric hospital. Epidemiol Infect: 1-7.

2186. Inoue Y, Imanishi Y, Kitahori Y (2008) An outbreak of group A rotavirus G1P[8] in an elementary school, Nara prefecture. Jpn J Infect Dis 61: 426.

2187. Inouye S, Hasegawa A, Matsuno S, Katow S (1984) Changes in antibody avidity after virus infections: detection by an immunosorbent assay in which a mild protein-denaturing agent is employed. J Clin Microbiol 20: 525-529.

2188. Inouye S, Matsuno S, Kono R (1981) Difference in antibody reactivity between complement fixation and immune adherence hemagglutination tests with virus antigens. J Clin Microbiol 14: 241-246.

2189. Inouye S, Matsuno S, Yamaguchi H (1984) Efficient coating of the solid phase with rotavirus antigens for enzyme-linked immunosorbent assay of immunoglobulin A antibody in feces. J Clin Microbiol 19: 259-263.

2190. Inouye S, Yamashita K, Yamadera S, Yoshikawa M, Kato N, et al. (2000) Surveillance of viral gastroenteritis in Japan: pediatric cases and outbreak incidents. J Infect Dis 181 Suppl 2: S270-274.

2191. Intusoma U, Sornsrivichai V, Jiraphongsa C, Varavithaya W (2008) Epidemiology, clinical presentations and burden of rotavirus diarrhea in children under five seen at Ramathibodi Hospital, Thailand. J Med Assoc Thai 91: 1350-1355.

2192. Ioi H, Kawashima H, Nishimata S, Watanabe Y, Yamanaka G, et al. (2006) A case of Reye syndrome with rotavirus infection accompanied with high cytokines. J Infect 52: e124-128.

2193. Iosef C, Chang KO, Azevedo MS, Saif LJ (2002) Systemic and intestinal antibody responses to NSP4 enterotoxin of Wa human rotavirus in a gnotobiotic pig model of human rotavirus disease. J Med Virol 68: 119-128.

2194. Isa P, Arias CF, Lopez S (2006) Role of sialic acids in rotavirus infection. Glycoconj J 23: 27-37.

2195. Isa P, Wood AR, Netherwood T, Ciarlet M, Imagawa H, et al. (1996) Survey of equine rotaviruses shows conservation of one P genotype in background of two G genotypes. Arch Virol 141: 1601-1612.

2196. Isaacs D, Day D, Crook S (1986) Childhood gastroenteritis: a population study. Br Med J (Clin Res Ed) 293: 545-546.

2197. Ishida S, Yoshizumi S, Ikeda T, Miyoshi M, Okano M, et al. (2008) Sensitive and rapid detection of norovirus using duplex TaqMan reverse transcription-polymerase chain reaction. J Med Virol 80: 913-920.

2198. Ishimaru Y, Nakano H, Oseto M, Yamashita Y, Kobayashi N, et al. (1990) Group C rotavirus infection and infiltration. Acta Paediatr Jpn 32: 523-529.

2199. Ishimaru Y, Nakano S, Nakano H, Oseto M, Yamashita Y (1991) Epidemiology of group C rotavirus gastroenteritis in Matsuyama, Japan. Acta Paediatr Jpn 33: 50-56.

2200. Ishino M, Mise K, Takemura H, Ahmed MU, Alam MM, et al. (2006) Comparison of NSP4 protein between group A and B human rotaviruses: detection of novel diarrhea-causing sequences in group B NSP4. Arch Virol 151: 173-182.

2201. Ishizaki H, Ohta C, Shirahata T, Goto H, Taniguchi K, et al. (1995) Persistence of a single electropherotype and serotype (G6P5) of bovine rotavirus in calves on a closed dairy farm from 1990 to 1993. Am J Vet Res 56: 1019-1024.

2202. Ishizaki H, Sakai T, Shirahata T, Taniguchi K, Urasawa T, et al. (1996) The distribution of G and P types within isolates of bovine rotavirus in Japan. Vet Microbiol 48: 367-372.

2203. Isik U, Caliskan M (2008) Reversible EEG changes during rotavirus gastroenteritis. Brain Dev 30: 73-76.

2204. Isik U, Caliskan M (2008) Reversible EEG changes during rotavirus gastroenteritis. Brain Dev 30: 73-76.

2205. Islam MN, Hossain MA, Rahman M, Yasmin M, Alam AN, et al. (1995) Development and evaluation of co-agglutination test to detect rotavirus antigens in stools of patients with diarrhoea. Bangladesh Med Res Counc Bull 21: 11-17.

2206. Ismaeel AY, Jamsheer AE, Yousif AQ, Al-Otaibi MA, Botta GA (2002) Causative pathogens of severe diarrhea in children. Saudi Med J 23: 1064-1069.

2207. Isolauri E (2000) The use of probiotics in paediatrics. Hosp Med 61: 6-7.

2208. Isolauri E, Jalonen T, Maki M (1989) Acute gastroenteritis. Changing pattern of clinical features and management. Acta Paediatr Scand 78: 685-691.

2209. Isolauri E, Joensuu J, Suomalainen H, Luomala M, Vesikari T (1995) Improved immunogenicity of oral D x RRV reassortant rotavirus vaccine by Lactobacillus casei GG. Vaccine 13: 310-312.

2210. Isolauri E, Juntunen M, Rautanen T, Sillanaukee P, Koivula T (1991) A human Lactobacillus strain (Lactobacillus casei sp strain GG) promotes recovery from acute diarrhea in children. Pediatrics 88: 90-97.

2211. Isolauri E, Juntunen M, Wiren S, Vuorinen P, Koivula T (1989) Intestinal permeability changes in acute gastroenteritis: effects of clinical factors and nutritional management. J Pediatr Gastroenterol Nutr 8: 466-473.

2212. Isolauri E, Kaila M, Arvola T, Majamaa H, Rantala I, et al. (1993) Diet during rotavirus enteritis affects jejunal permeability to macromolecules in suckling rats. Pediatr Res 33: 548-553.

2213. Isolauri E, Kaila M, Mykkanen H, Ling WH, Salminen S (1994) Oral bacteriotherapy for viral gastroenteritis. Dig Dis Sci 39: 2595-2600.

2214. Iturriza Gomara M, Kang G, Mammen A, Jana AK, Abraham M, et al. (2004) Characterization of G10P[11] rotaviruses causing acute gastroenteritis in neonates and infants in Vellore, India. J Clin Microbiol 42: 2541-2547.

2215. Iturriza Gomara M, Simpson R, Perault AM, Redpath C, Lorgelly P, et al. (2008) Structured surveillance of infantile gastroenteritis in East Anglia, UK: incidence of infection with common viral gastroenteric pathogens. Epidemiol Infect 136: 23-33.

2216. Iturriza-Gomara M, Auchterlonie IA, Zaw W, Molyneaux P, Desselberger U, et al. (2002) Rotavirus gastroenteritis and central nervous system (CNS) infection: characterization of the VP7 and VP4 genes of rotavirus strains isolated from paired fecal and cerebrospinal fluid samples from a child with CNS disease. J Clin Microbiol 40: 4797-4799.

2217. Iturriza-Gomara M, Cubitt D, Steele D, Green J, Brown D, et al. (2000) Characterisation of rotavirus G9 strains isolated in the UK between 1995 and 1998. J Med Virol 61: 510-517.

2218. Iturriza-Gomara M, Elliot AJ, Dockery C, Fleming DM, Gray JJ (2008) Structured surveillance of infectious intestinal disease in pre-school children in the community: 'The Nappy Study'. Epidemiol Infect: 1-10.

2219. Iturriza-Gomara M, Green J, Brown DW, Desselberger U, Gray JJ (1999) Comparison of specific and random priming in the reverse transcriptase polymerase chain reaction for genotyping group A rotaviruses. J Virol Methods 78: 93-103.

2220. Ivannikova TA, Korolev MB, Shekoian LA (1984) [Immunofluorescent study of the reproduction of human rotavirus in cell culture]. Vopr Virusol 29: 589-592.

2221. Ivanoff B (1998) [Traveller's diarrhea: which vaccines?]. Bull Soc Pathol Exot 91: 456-460.

2222. Ivanoff B, Glass R (1999) [Vaccines for rotavirus infections: efficacy and eventual role of vaccination programs]. Arch Pediatr 6 Suppl 2: 330s-331s.

2223. Ivanoff B, Glass RI (2001) [Vaccines against rotavirus infections]. Med Trop (Mars) 61: 262-263.

2224. Ivanoff B, Neira M (1998) [Vaccination against diarrheal diseases and typhoid fever. Current status and prospects]. Ann Med Interne (Paris) 149: 340-350.

2225. Iwasa T, Matsubayashi N (2008) Protein-loosing enteropathy associated with rotavirus infection in an infant. World J Gastroenterol 14: 1630-1632.

2226. Iyadurai S, Troester M, Harmala J, Bodensteiner J (2007) Benign afebrile seizures in acute gastroenteritis: is rotavirus the culprit? J Child Neurol 22: 887-890.

2227. Jacobs P, Shane L, Fassbender K, Wang E, Moineddin R, et al. (2002) Economic analysis of rotavirus-associated diarrhea in the metropolitan Toronto and Peel regions of Ontario. Can J Infect Dis 13: 167-174.

2228. Jacobson J, Bohn D (1993) Severe hypernatremic dehydration and hyperkalemia in an infant with gastroenteritis secondary to rotavirus. Ann Emerg Med 22: 1630-1632.

2229. Jacobson RM (1999) The current status of the rotavirus vaccine. Vaccine 17: 1690-1699.

2230. Jacobsson PA, Johansson ME, Wadell G (1979) Identification of an enteric adenovirus by immunoelectroosmophoresis (IEOP) technique. J Med Virol 3: 307-312.

2231. Jagannath MR, Kesavulu MM, Deepa R, Sastri PN, Kumar SS, et al. (2006) N- and C-terminal cooperation in rotavirus enterotoxin: novel mechanism of modulation of the properties of a multifunctional protein by a structurally and functionally overlapping conformational domain. J Virol 80: 412-425.

2232. Jagannath MR, Vethanayagam RR, Reddy BS, Raman S, Rao CD (2000) Characterization of human symptomatic rotavirus isolates MP409 and MP480 having 'long' RNA electropherotype and subgroup I specificity, highly related to the P6[1],G8 type bovine rotavirus A5, from Mysore, India. Arch Virol 145: 1339-1357.

2233. Jaimes MC, Rojas OL, Gonzalez AM, Cajiao I, Charpilienne A, et al. (2002) Frequencies of virus-specific CD4(+) and CD8(+) T lymphocytes secreting gamma interferon after acute natural rotavirus infection in children and adults. J Virol 76: 4741-4749.

2234. Jain V, Das BK, Bhan MK, Glass RI, Gentsch JR (2001) Great diversity of group A rotavirus strains and high prevalence of mixed rotavirus infections in India. J Clin Microbiol 39: 3524-3529.

2235. Jain V, Parashar UD, Glass RI, Bhan MK (2001) Epidemiology of rotavirus in India. Indian J Pediatr 68: 855-862.

2236. Jakab F, Peterfai J, Meleg E, Banyai K, Mitchell DK, et al. (2005) Comparison of clinical characteristics between astrovirus and rotavirus infections diagnosed in 1997 to 2002 in Hungary. Acta Paediatr 94: 667-671.

2237. Jalonen T, Isolauri E, Heyman M, Crain-Denoyelle AM, Sillanaukee P, et al. (1991) Increased beta-lactoglobulin absorption during rotavirus enteritis in infants: relationship to sugar permeability. Pediatr Res 30: 290-293.

2238. James VL, Lambden PR, Caul EO, Clarke IN (1998) Enzyme-linked immunosorbent assay based on recombinant human group C rotavirus inner capsid protein (VP6) To detect human group C rotaviruses in fecal samples. J Clin Microbiol 36: 3178-3181.
[truncated: 550,396 more chars]
